# Transcriptomes of bovine ovarian follicular and luteal cells

**DOI:** 10.1016/j.dib.2016.11.093

**Published:** 2016-12-10

**Authors:** Sarah M. Romereim, Adam F. Summers, William E. Pohlmeier, Pan Zhang, Xiaoying Hou, Heather A. Talbott, Robert A. Cushman, Jennifer R. Wood, John S. Davis, Andrea S. Cupp

**Affiliations:** a University of Nebraska–Lincoln; Animal Science, P.O. Box 830908, C203 ANSC, Lincoln, NE 68583-0908, USA; b University of Nebraska Medical Center; 983255 Nebraska Medical Center, Omaha, NE 68198-3255, USA; c USDA, ARS, US Meat Animal Research Center, Nutrition and Environmental Management Research, Spur 18D, Clay Center, NE 68933, USA; d VA Nebraska-Western Iowa Health Care System, Omaha, NE 68105, USA

## Abstract

Affymetrix Bovine GeneChip® Gene 1.0 ST Array RNA expression analysis was performed on four somatic ovarian cell types: the granulosa cells (GCs) and theca cells (TCs) of the dominant follicle and the large luteal cells (LLCs) and small luteal cells (SLCs) of the corpus luteum. The normalized linear microarray data was deposited to the NCBI GEO repository (GSE83524). Subsequent ANOVA determined genes that were enriched (≥2 fold more) or decreased (≤−2 fold less) in one cell type compared to all three other cell types, and these analyzed and filtered datasets are presented as tables. Genes that were shared in enriched expression in both follicular cell types (GCs and TCs) or in both luteal cells types (LLCs and SLCs) are also reported in tables. The standard deviation of the analyzed array data in relation to the log of the expression values is shown as a figure. These data have been further analyzed and interpreted in the companion article “Gene expression profiling of ovarian follicular and luteal cells provides insight into cellular identities and functions” (Romereim et al., 2017) [1].

**Specifications Table**

**Table:** 

Subject area	*Biology*
More specific subject area	*Reproductive Physiology*
Type of data	*Tables, figure*
How data was acquired	*RNA Microarray*
Data format	*Normalized, analyzed, filtered*
Experimental factors	*Isolation of four ovarian somatic cell types*
Experimental features	*A comparative transcriptome analysis of the granulosa cells and theca cells of the ovarian follicle versus the large and small luteal cells of the corpus luteum provides cell type markers, functional insight, and support for the differentiation lineage model describing where the luteal cells originate.*
Data source location	*Lincoln and Omaha, NE, USA*
Data accessibility	*Filtered dataset is present as tables in this article, raw data is in the public NCBI repository GEO*GSE83524. http://www.ncbi.nlm.nih.gov/geo/query/acc.cgi?acc=GSE83524

**Value of the data**

• The follicular GC and TC transcriptome data can be compared to previously published bovine gene expression analyses for corroboration [2], [3] and are valuable in metadata analyses investigating GC and TC transcriptomes at different stages or in different species.
• There are no previously published transcriptomes available for large luteal cells (LLC) and small luteal cells (SLC). Therefore, the luteal cell gene expression data allow novel insight into these two cell types.
• Lists of identified genes that are specifically enriched in each somatic ovarian cell type can inform future physiological research on the functions of the ovarian somatic cells.

## 1 Data

**Fig. 1:**
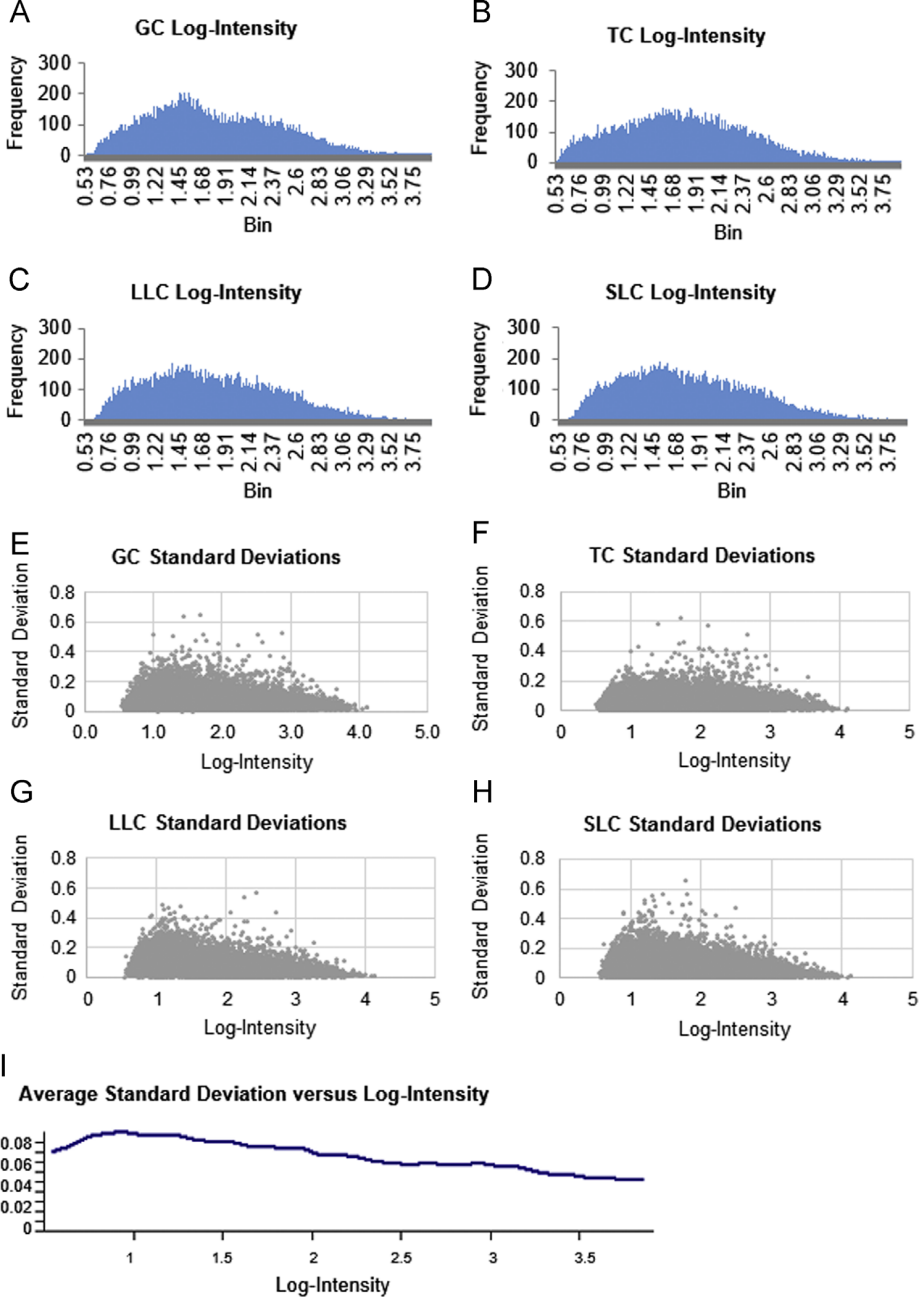
Microarray data log-intensities and standard deviations. A histogram of the frequency of microarray targets at a given average log-intensity for each cell type is provided for GC (A), TC (B), LLC (C), and SLC (D). The standard deviations versus each log-intensity are graphed as scatter plots for GC (E), TC (F), LLC (G), and SLC (H). The average standard deviation for all cell types and microarray replicates versus log-intensity is plotted in (I). RNA targets detected at a lower intensity (suggesting low levels of expression) have a slightly higher standard deviation and a poor signal to noise ratio. The overall average standard deviation, however, is low.

• The normalized linear microarray data are available at the NCBI GEO repository: GSE83524 at http://www.ncbi.nlm.nih.gov/geo/query/acc.cgi?acc=GSE83524
• Fig. 1: A graph depicting the frequency of log-intensities (A–D) and standard deviation versus log-intensity (E–H) of the microarray data for each cell type as well as the average standard deviation versus log-intensity for all of the microarray data (I).

All tables listed below contain the Affymetrix probeset ID, gene symbol, description of the gene, fold changes, and relevant normalized linear microarray data. There are four microarray replicates for the GC cell type and three replicates for the TCs, LLCs, and SLCs.

• Table 1: The filtered set of genes that are enriched or decreased in the GCs compared to the TCs, LLCs, and SLCs.
• Table 2: The filtered set of genes that are enriched or decreased in the TCs compared to the GCs, LLCs, and SLCs.
• Table 3: The filtered set of genes that are enriched or decreased in the LLCs compared to the GCs, TCs, and SLCs.
• Table 4: The filtered set of genes that are enriched or decreased in the SLCs compared to the GCs, TCs, and LLCs.
• Table 5: The filtered data showing genes that are enriched in both GCs and TCs compared to the LLCs and SLCs.
• Table 6: The filtered data showing genes that are enriched in both LLCs and SLCs compared to the GCs and TCs.

## 2 Experimental design, materials and methods

Follicular granulosa (*n*=4) and theca cells (*n*=3) were isolated from estrogen active dominant follicles in ovaries of beef cows (75% Red Angus, 25% MARC III) from the physiology herd located at the University of Nebraska Agricultural Research and Development Center. The University of Nebraska–Lincoln Institutional Animal Care and Use Committee approved all procedures and facilities used in this experiment. Estrous cycles of cows were synchronized a modified Co-Synch protocol using gonadotropin releasing hormone (GnRH) and a controlled internal drug release device (CIDR; 1.38 g progesterone, Zoetis) for 7 days with a PGF2α (25 mg/mL; Lutalyse, Pfizer Animal Health) injection at CIDR removal [4]. Ovariectomy was performed approximately 36 h after CIDR removal. Upon ovariectomy, each dominant antral follicle was aspirated/dissected and the granulosa cells, theca cells, and follicular fluid were isolated as described previously [4]. Both granulosa and theca cells were homogenized in Tri-reagent (Sigma-Aldrich) for RNA isolation.

Luteal cells were isolated by elutriation from the corpora lutea of ovaries collected at a local abattoir (JBSSA, Omaha, NE) as described previously [5], [6]. Each corpus luteum (*n*=3 for both LLC and SLC) was digested with collagenase to dissociate the cells, which were then suspended in a solution of DMEM (calcium free, 3.0 g/L glucose, antibiotics, 25 mM HEPES, 3.8 g/L sodium bicarbonate; 0.1% BSA, 0.02 mg/mL deoxyribonuclease, pH 7.2) to a total volume of 30 mL. Elutriation was then performed with a Beckman Coulter Avanti-J20X centrifuge with a JE 5.0 rotor. The cells were applied to a Sanderson (Beckman) elutriation chamber and the eluates were collected with continuous flow with each fraction comprising 100 mL of eluate. The first cell fraction was collected at a flow rate of 16 mL/min with a centrifuge speed of 1800 rpm and contained predominantly red blood cells. The second collected fraction was collected at a flow rate of 16 mL/min and a rotor speed of 1400 rpm and contained mostly (SLCs). The third fraction was collected at 24 mL/min at a rotor speed of 1200 rpm and contained predominantly SLCs. The fourth fraction was collected at a flow rate of 30 mL/min and a speed of 680 rpm and contained a majority of large luteal cells (≥80%) along with some SLC and endothelial cells. SLCs and LLCs were concentrated with additional centrifugation of the relevant fractions, and the cells were homogenized in Tri-reagent (Sigma-Aldrich) for RNA isolation.

After RNA extraction, 200 ng RNA for each sample were submitted to the University of Nebraska Microarray Core facility where the Affymetrix Bovine GeneChip® Gene 1.0 ST Array RNA expression analysis was performed. The microarray data were normalized with Robust Multi-Array Averaging. Array analysis was then performed using the National Institute of Aging tool (http://lgsun.grc.nia.nih.gov/ANOVA/) for Analysis of Variance (ANOVA).

## Support

This research was supported by USDA Hatch Grant NEB26-202/W2112 to AC, Hatch –NEB ANHL 26-213 to AC and JW, NEB 26-206 to AC and JW, NIFA 2013-67015-20965 to AC, JW and JD, NIFA Grant 2011-67015-20076 to JD and AC, Postdoctoral Fellowship 2016-67012-24697 to SR, and Predoctoral Fellowship 2014-67011-22280 to HT. USDA is an equal opportunity provider and employer. Names are necessary to report factually on available data; however, the USDA neither guarantees nor warrants the standard of the product, and the use of names by the USDA implies no approval of the product to the exclusion of others that may also be suitable. The research was also supported by funds from the VA Research and Development service.
