# Supplementary material for: Transcriptomes of bovine ovarian follicular and luteal cells
Source: Data Brief. 2016 Dec 10;10:335–9. doi: 10.1016/j.dib.2016.11.093 (PMC5157705; doi:10.1016/j.dib.2016.11.093)
Supplement: Supplementary file 1 — Supplementary material [file mmc1.docx]

Conflicts of interest: The authors have no conflicts of interest to disclose.
