# Supplementary material for: Transcriptomes of bovine ovarian follicular and luteal cells
Source: Data Brief. 2016 Dec 10;10:335–9. doi: 10.1016/j.dib.2016.11.093 (PMC5157705; doi:10.1016/j.dib.2016.11.093)
Supplement: Supplementary file 2 — Supplementary material [file mmc2.docx]

| **Table 1. Genes Enriched (≥ 2-fold greater expression than all other cells types) or Decreased (≤ -2-fold) in Granulosa Cells** | | | |  |  |  | **Linear Microarray Results (arbitrary units)** | | | | | | | | | | | | |
| --- | --- | --- | --- | --- | --- | --- | --- | --- | --- | --- | --- | --- | --- | --- | --- | --- | --- | --- | --- |
| **Probeset ID** | **Gene Symbol** | **Description** | **Functional Category** | **Fold Change (GC vs LLC)** | **Fold Change (GC vs SLC)** | **Fold Change (GC vs TC)** | **GC1** | **GC2** | **GC3** | **GC4** | **TC1** | **TC2** | **TC3** | **LLC1** | **LLC2** | **LLC3** | **SLC1** | **SLC2** | **SLC3** |
| 12749545 | BCAM | Bos taurus basal cell adhesion molecule (Lutheran blood group) | adhesion | -12.519 | -10.167 | -2.704 | 103 | 100 | 141 | 89 | 257 | 353 | 265 | 1384 | 1422 | 1212 | 1104 | 1186 | 975 |
| 12780102 | FN1 | Bos taurus fibronectin 1 | adhesion | -10.901 | -8.354 | -19.244 | 142 | 169 | 325 | 111 | 2986 | 4053 | 2978 | 2149 | 2054 | 1484 | 1773 | 1325 | 1254 |
| 12867984 | TENC1 | PREDICTED: Bos taurus tensin like C1 domain containing phosphatase (tensin 2) | adhesion | -7.652 | -3.793 | -3.561 | 114 | 128 | 121 | 63 | 348 | 443 | 317 | 844 | 777 | 739 | 392 | 444 | 340 |
| 12793983 | PEAK1 | Pseudopodium-Enriched Atypical Kinase 1 | adhesion | -4.348 | -5.78 | -4.343 | 138 | 159 | 184 | 111 | 693 | 682 | 535 | 667 | 749 | 507 | 816 | 935 | 782 |
| 12726044 | SDC2 | Syndecan 2 | adhesion | -4.21 | -2.502 | -9.276 | 109 | 97 | 122 | 116 | 742 | 1327 | 1094 | 412 | 541 | 452 | 334 | 189 | 335 |
| 12683338 | CXADR | Bos taurus coxsackie virus and adenovirus receptor | adhesion | -4.162 | -8.992 | -2.709 | 110 | 98 | 97 | 82 | 216 | 238 | 345 | 475 | 380 | 358 | 1017 | 924 | 693 |
| 12685065 | LPP | Bos taurus LIM domain containing preferred translocation partner in lipoma | adhesion | -3.634 | -4.092 | -3.714 | 297 | 309 | 356 | 235 | 1057 | 1364 | 923 | 1038 | 1150 | 1045 | 1143 | 1269 | 1228 |
| 12730659 | PARVA | Parvin, Alpha | adhesion | -2.625 | -2.584 | -2.495 | 317 | 389 | 485 | 405 | 918 | 1154 | 900 | 1075 | 1083 | 955 | 1081 | 1025 | 957 |
| 12791542 | BTBD7 | PREDICTED: Bos taurus BTB (POZ) domain containing 7 | adhesion | 2.186 | 2.175 | 2.003 | 608 | 611 | 741 | 583 | 334 | 325 | 291 | 340 | 290 | 246 | 285 | 312 | 276 |
| 12732724 | PVRL1 | Bos taurus poliovirus receptor-related 1 (herpesvirus entry mediator C) | adhesion | 2.646 | 2.992 | 2.092 | 168 | 170 | 200 | 126 | 103 | 73 | 64 | 64 | 44 | 84 | 59 | 48 | 58 |
| 12699857 | ITGB1BP1 | Integrin Beta 1 Binding Protein 1 | adhesion | 2.682 | 2.78 | 2.035 | 397 | 399 | 419 | 520 | 223 | 180 | 236 | 191 | 153 | 142 | 179 | 147 | 141 |
| 12757828 | CDH8 | Bos taurus cadherin 8, type 2 | adhesion | 3.341 | 5.974 | 4.841 | 148 | 113 | 57 | 78 | 15 | 21 | 22 | 36 | 21 | 29 | 15 | 15 | 16 |
| 12786958 | ITGA2 | Bos taurus integrin, alpha 2 (CD49B, alpha 2 subunit of VLA-2 receptor) | adhesion | 3.919 | 9.675 | 6.182 | 341 | 388 | 142 | 720 | 57 | 69 | 43 | 77 | 100 | 85 | 35 | 35 | 35 |
| 12686432 | IGSF11 | Bos taurus immunoglobulin superfamily, member 11 | adhesion | 4.998 | 5.064 | 2.234 | 2772 | 2771 | 2462 | 2890 | 1356 | 1041 | 1277 | 577 | 599 | 466 | 601 | 609 | 423 |
| 12768287 | TMIGD1 | Bos taurus transmembrane and immunoglobulin domain containing 1 | adhesion | 7.735 | 6.015 | 4.455 | 142 | 135 | 148 | 116 | 27 | 25 | 41 | 14 | 18 | 20 | 24 | 23 | 20 |
| 12887066 | EDIL3 | EGF-Like Repeats And Discoidin I-Like Domains 3 | adhesion | 13.07 | 33.151 | 2.778 | 553 | 735 | 384 | 1287 | 289 | 211 | 229 | 78 | 28 | 61 | 19 | 27 | 16 |
| 12720176 | FLRT3 | Bos taurus fibronectin leucine rich transmembrane protein 3 | adhesion | 13.648 | 21.998 | 2.408 | 455 | 516 | 368 | 527 | 174 | 159 | 255 | 42 | 33 | 28 | 19 | 21 | 23 |
| 12786403 | CDH12 | Bos taurus cadherin 12, type 2 (N-cadherin 2) | adhesion | 107.919 | 166.916 | 2.746 | 1464 | 1508 | 1597 | 1217 | 526 | 540 | 507 | 27 | 10 | 9 | 11 | 7 | 8 |
| 12727146 | CD82 | Bos taurus CD82 molecule | apoptosis | -14.2 | -13.899 | -7.321 | 170 | 166 | 191 | 202 | 1777 | 1040 | 1270 | 2699 | 2484 | 2555 | 2975 | 2353 | 2294 |
| 12787924 | FAM105A | Bos taurus family with sequence similarity 105, member A | apoptosis | -2.299 | -5.88 | -6.146 | 211 | 196 | 227 | 153 | 1225 | 1255 | 1112 | 648 | 418 | 330 | 1254 | 1475 | 809 |
| 12720625 | FAM188A | Bos taurus family with sequence similarity 188, member A | apoptosis | 3.794 | 4.813 | 2.484 | 1792 | 1916 | 1497 | 1891 | 713 | 664 | 758 | 462 | 446 | 488 | 394 | 383 | 327 |
| 12810714 | IER3IP1 | Bos taurus immediate early response 3 interacting protein 1 | apoptosis | 5.046 | 4.322 | 2.082 | 911 | 1087 | 1008 | 1140 | 556 | 450 | 487 | 211 | 185 | 219 | 269 | 199 | 255 |
| 12755826 | PSMB10 | Bos taurus proteasome (prosome, macropain) subunit, beta type, 10 | catabolism | -4.116 | -3.645 | -3.896 | 107 | 106 | 134 | 98 | 474 | 311 | 540 | 449 | 414 | 505 | 436 | 358 | 417 |
| 12734667 | PRDX6 | Bos taurus peroxiredoxin 6 | catabolism | -2.54 | -3.576 | -2.771 | 192 | 218 | 156 | 144 | 448 | 503 | 509 | 463 | 372 | 513 | 677 | 519 | 703 |
| 12683191 | LMLN | PREDICTED: Bos taurus leishmanolysin-like (metallopeptidase M8 family) | catabolism | 2.301 | 2.47 | 2.265 | 150 | 128 | 132 | 157 | 63 | 55 | 69 | 68 | 48 | 70 | 51 | 58 | 63 |
| 12709400 | RNASEH2B | Bos taurus ribonuclease H2, subunit B | catabolism | 3.1 | 3.085 | 2.359 | 1158 | 1111 | 1012 | 986 | 454 | 471 | 430 | 302 | 377 | 355 | 354 | 306 | 379 |
| 12763375 | CDC6 | Bos taurus cell division cycle 6 homolog (S. cerevisiae) | cell cycle | 7.382 | 6.495 | 2.668 | 210 | 205 | 157 | 140 | 81 | 52 | 67 | 16 | 36 | 23 | 32 | 21 | 29 |
| 12830605 | CHEK1 | Bos taurus CHK1 checkpoint homolog (S. pombe) | cell cycle | 8.643 | 9.011 | 3.117 | 219 | 217 | 188 | 160 | 63 | 50 | 77 | 21 | 22 | 24 | 36 | 18 | 15 |
| 12843787 | CDCA8 | Bos taurus cell division cycle associated 8 | cell cycle | 9.442 | 10.791 | 2.72 | 143 | 150 | 165 | 132 | 56 | 55 | 51 | 17 | 13 | 18 | 15 | 13 | 13 |
| 12825838 | CDK1 | Cyclin-Dependent Kinase 1 | cell cycle | 10.896 | 13.708 | 3.461 | 231 | 277 | 226 | 215 | 92 | 60 | 57 | 29 | 22 | 16 | 15 | 17 | 21 |
| 12844886 | CDC20 | Bos taurus cell division cycle 20 homolog (S. cerevisiae) | cell cycle | 13.617 | 16.572 | 2.532 | 468 | 471 | 513 | 471 | 210 | 165 | 198 | 37 | 34 | 36 | 31 | 31 | 25 |
| 12691926 | CDKN3 | Bos taurus cyclin-dependent kinase inhibitor 3 | cell cycle | 14.896 | 14.157 | 3.502 | 399 | 461 | 384 | 329 | 113 | 104 | 117 | 27 | 26 | 25 | 26 | 26 | 32 |
| 12869943 | CCNA2 | Bos taurus cyclin A2 | cell cycle | 15.977 | 19.815 | 3.497 | 619 | 620 | 533 | 576 | 204 | 139 | 165 | 60 | 35 | 23 | 26 | 28 | 35 |
| 12767844 | BIRC5 | Baculoviral IAP Repeat Containing 5 | cell survival | 8.258 | 8.892 | 2.722 | 518 | 513 | 547 | 365 | 183 | 156 | 191 | 73 | 60 | 45 | 59 | 50 | 53 |
| 12881065 | HIST3H2A | Bos taurus histone cluster 3, H2a | chromosome structure | -4.498 | -4.605 | 2.451 | 100 | 114 | 118 | 81 | 51 | 35 | 42 | 280 | 461 | 760 | 376 | 346 | 810 |
| 12841208 | HIST2H2BF | Histone Cluster 2, H2bf | chromosome structure | 2.476 | 2.34 | 2.035 | 264 | 232 | 316 | 292 | 185 | 111 | 119 | 95 | 170 | 83 | 100 | 133 | 121 |
| 12825587 | MLF1IP | Bos taurus MLF1 interacting protein, mRNA (cDNA clone IMAGE:8014917), partial cds. (also known as Centromere Protein U) | chromosome structure | 2.607 | 2.056 | 2.697 | 300 | 329 | 204 | 261 | 117 | 69 | 124 | 99 | 108 | 103 | 117 | 123 | 157 |
| 12679896 | CHAF1B | Bos taurus chromatin assembly factor 1, subunit B (p60) | chromosome structure | 2.649 | 2.395 | 2.325 | 151 | 131 | 113 | 96 | 63 | 49 | 45 | 44 | 50 | 43 | 62 | 34 | 61 |
| 12742180 | CETN4 | Bos taurus centrin 4 | chromosome structure | 2.687 | 2.811 | 2.768 | 188 | 187 | 157 | 248 | 84 | 67 | 60 | 88 | 50 | 82 | 76 | 59 | 71 |
| 12801577 | HIST1H1D | Bos taurus histone cluster 1, H1d | chromosome structure | 2.695 | 3.347 | 2.723 | 154 | 169 | 193 | 104 | 84 | 51 | 40 | 68 | 50 | 52 | 57 | 26 | 63 |
| 12903405 | PHF16 | PREDICTED: Bos taurus PHD finger protein 16 | chromosome structure | 4.423 | 4.255 | 2.894 | 352 | 361 | 401 | 425 | 119 | 138 | 142 | 97 | 77 | 88 | 88 | 68 | 123 |
| 12716539 | SUV39H2 | Bos taurus suppressor of variegation 3-9 homolog 2 (Drosophila) | chromosome structure | 4.757 | 3.714 | 2.339 | 114 | 126 | 93 | 134 | 59 | 47 | 43 | 25 | 30 | 19 | 34 | 27 | 33 |
| 12890056 | CEP44 | Bos taurus centrosomal protein 44kDa | chromosome structure | 4.814 | 4.007 | 2.572 | 101 | 119 | 110 | 115 | 46 | 40 | 44 | 25 | 24 | 20 | 27 | 32 | 25 |
| 12787132 | CENPH | Bos taurus centromere protein H | chromosome structure | 4.846 | 6.406 | 3.723 | 247 | 322 | 244 | 269 | 81 | 54 | 87 | 55 | 54 | 57 | 51 | 42 | 35 |
| 12805834 | XM_002697510 | PREDICTED: Bos taurus histone cluster 1, H4i-like (LOC518961) | chromosome structure | 5.346 | 3.158 | 3.033 | 272 | 334 | 270 | 228 | 159 | 73 | 63 | 56 | 63 | 38 | 77 | 103 | 82 |
| 12889307 | CENPP | Centromere Protein P | chromosome structure | 5.795 | 6.972 | 2.861 | 162 | 162 | 130 | 138 | 58 | 49 | 48 | 29 | 33 | 17 | 24 | 15 | 28 |
| 12799135 | FANCD2 | Bos taurus Fanconi anemia, complementation group D2 | chromosome structure | 5.796 | 3.964 | 2.738 | 179 | 180 | 168 | 145 | 62 | 59 | 63 | 30 | 36 | 22 | 41 | 44 | 42 |
| 12899402 | CENPW | Bos taurus centromere protein W | chromosome structure | 6.266 | 5.45 | 2.73 | 296 | 258 | 269 | 196 | 128 | 78 | 78 | 41 | 42 | 38 | 62 | 42 | 38 |
| 12803586 | CENPQ | Bos taurus centromere protein Q | chromosome structure | 6.453 | 5.504 | 3.705 | 125 | 153 | 101 | 95 | 33 | 25 | 37 | 22 | 17 | 16 | 24 | 19 | 20 |
| 12751437 | CENPN | Bos taurus centromere protein N | chromosome structure | 7.131 | 4.743 | 2.772 | 444 | 402 | 405 | 384 | 157 | 115 | 176 | 52 | 50 | 72 | 98 | 57 | 114 |
| 12891472 | SMC2 | Structural Maintenance Of Chromosomes 2 | chromosome structure | 8.192 | 7.601 | 2.757 | 778 | 830 | 657 | 597 | 252 | 235 | 288 | 90 | 103 | 70 | 119 | 77 | 88 |
| 12805811 | XM_002697529 | PREDICTED: Bos taurus histone cluster 1, H3a-like (LOC517139) | chromosome structure | 8.98 | 7.906 | 2.031 | 487 | 487 | 408 | 691 | 357 | 174 | 253 | 68 | 59 | 46 | 65 | 67 | 61 |
| 12802575 | HIST1H4F | PREDICTED: Bos taurus histone cluster 1, H4i-like (LOC527388) | chromosome structure | 9.379 | 7.968 | 2.552 | 455 | 366 | 324 | 350 | 229 | 98 | 136 | 29 | 55 | 39 | 45 | 39 | 56 |
| 12847661 | HJURP | Holliday Junction Recognition Protein | chromosome structure | 9.673 | 7.081 | 2.997 | 234 | 246 | 190 | 157 | 60 | 73 | 72 | 20 | 24 | 19 | 31 | 28 | 28 |
| 12820806 | HELLS | PREDICTED: Bos taurus helicase, lymphoid-specific | chromosome structure | 10.451 | 11.972 | 3.187 | 377 | 420 | 305 | 364 | 132 | 103 | 110 | 35 | 35 | 35 | 38 | 26 | 29 |
| 12802519 | HIST1H2BB | PREDICTED: Bos taurus histone cluster 1, H2bb | chromosome structure | 15.999 | 12.387 | 3.389 | 1266 | 1226 | 971 | 823 | 494 | 216 | 283 | 69 | 58 | 72 | 83 | 89 | 83 |
| 12819603 | CEP55 | Bos taurus centrosomal protein 55kDa | chromosome structure | 18.578 | 3.277 | 2.338 | 551 | 590 | 549 | 473 | 274 | 237 | 188 | 48 | 35 | 15 | 141 | 324 | 97 |
| 12785235 | CENPK | Bos taurus centromere protein K | chromosome structure | 18.68 | 20.502 | 4.304 | 202 | 194 | 204 | 167 | 59 | 40 | 37 | 13 | 9 | 9 | 11 | 10 | 8 |
| 12740194 | CENPF | Bos taurus centromere protein F, 350/400kDa (mitosin) | chromosome structure | 22.834 | 27.802 | 3.074 | 390 | 388 | 391 | 377 | 117 | 122 | 139 | 20 | 17 | 15 | 15 | 15 | 12 |
| 12869954 | CENPE | Centromere Protein E | chromosome structure | 28.248 | 23.79 | 3.662 | 419 | 462 | 427 | 430 | 129 | 101 | 127 | 17 | 19 | 11 | 16 | 21 | 18 |
| 12707854 | CENPA | Bos taurus centromere protein A | chromosome structure | 43.501 | 46.762 | 3.958 | 511 | 479 | 601 | 505 | 181 | 110 | 115 | 8 | 17 | 12 | 15 | 7 | 12 |
| 12773515 | TOP2A | Topoisomerase (DNA) II Alpha 170kDa | chromosome structure | 51.676 | 71.779 | 3.48 | 1294 | 1489 | 1267 | 1295 | 361 | 370 | 420 | 42 | 23 | 18 | 15 | 28 | 15 |
| 12800906 | HIST1H2AD | PREDICTED: Bos taurus histone cluster 1, H2 | chromosome structure | 88.145 | 58.304 | 2.163 | 2923 | 3421 | 3026 | 2601 | 2321 | 1012 | 1111 | 47 | 45 | 19 | 48 | 52 | 53 |
| 12694111 | SQRDL | Bos taurus sulfide quinone reductase-like (yeast), nuclear gene encoding mitochondrial protein | compound biosynthesis | -10.582 | -8.45 | -2.834 | 224 | 243 | 135 | 150 | 646 | 475 | 450 | 1903 | 1736 | 2175 | 1576 | 1554 | 1494 |
| 12863929 | TST | Bos taurus thiosulfate sulfurtransferase (rhodanese), nuclear gene encoding mitochondrial protein | compound biosynthesis | -3.437 | -3.367 | -3.003 | 111 | 95 | 114 | 150 | 347 | 305 | 398 | 364 | 425 | 408 | 454 | 355 | 368 |
| 12739927 | B3GALT6 | PREDICTED: Bos taurus UDP-Gal:betaGal beta 1,3-galactosyltransferase polypeptide 6 | compound biosynthesis | 2.145 | 2.142 | 2.004 | 177 | 183 | 215 | 163 | 89 | 94 | 91 | 99 | 86 | 74 | 107 | 80 | 73 |
| 12694883 | PIGH | Bos taurus phosphatidylinositol glycan anchor biosynthesis, class H, transcript variant 1 | compound biosynthesis | 2.201 | 2.55 | 2.502 | 195 | 193 | 234 | 278 | 106 | 72 | 93 | 114 | 75 | 121 | 88 | 74 | 102 |
| 12804549 | MOCS1 | Bos taurus molybdenum cofactor synthesis 1, transcript variant a | compound biosynthesis | 2.203 | 2.059 | 2.017 | 309 | 334 | 282 | 306 | 191 | 148 | 125 | 121 | 150 | 149 | 130 | 152 | 167 |
| 12767532 | NOS2 | Bos taurus nitric oxide synthase 2, inducible | compound biosynthesis | 12.319 | 18.81 | 3.495 | 755 | 764 | 580 | 583 | 377 | 147 | 124 | 54 | 59 | 49 | 32 | 41 | 34 |
| 12900935 | MTHFD1L | Bos taurus methylenetetrahydrofolate dehydrogenase (NADP+ dependent) 1-like, nuclear gene encoding mitochondrial protein | compound biosynthesis | 12.401 | 7.522 | 2.707 | 678 | 702 | 467 | 887 | 266 | 217 | 259 | 57 | 54 | 50 | 78 | 124 | 72 |
| 12848949 | CALD1 | Bos taurus caldesmon 1 | cytoskeletal dynamics | -17.546 | -16.477 | -15.98 | 203 | 187 | 221 | 86 | 2342 | 2955 | 2602 | 2771 | 2996 | 2873 | 2748 | 2585 | 2779 |
| 12822288 | ACTA2 | Bos taurus actin, alpha 2, smooth muscle, aorta | cytoskeletal dynamics | -9.49 | -2.223 | -22.019 | 241 | 188 | 151 | 123 | 3484 | 5113 | 2968 | 1332 | 1513 | 2101 | 314 | 497 | 349 |
| 12688156 | ANXA2 | Bos taurus annexin A2 | cytoskeletal dynamics | -4.651 | -4.167 | -2.922 | 271 | 309 | 155 | 303 | 741 | 802 | 660 | 1209 | 1091 | 1200 | 1214 | 925 | 1014 |
| 12903657 | MPP1 | Bos taurus membrane protein, palmitoylated 1, 55kDa | cytoskeletal dynamics | -4.601 | -8.978 | -2.132 | 169 | 173 | 148 | 205 | 363 | 422 | 327 | 886 | 886 | 640 | 1790 | 1406 | 1483 |
| 12864334 | MYH9 | Bos taurus myosin, heavy chain 9, non-muscle | cytoskeletal dynamics | -4.516 | -3.328 | -2.436 | 253 | 225 | 251 | 205 | 494 | 741 | 496 | 1187 | 969 | 1004 | 767 | 917 | 657 |
| 12714968 | DSTN | Bos taurus destrin (actin depolymerizing factor) | cytoskeletal dynamics | -3.445 | -3.416 | -2.036 | 357 | 411 | 384 | 342 | 769 | 871 | 652 | 1244 | 1159 | 1468 | 1159 | 1344 | 1325 |
| 12692275 | IQGAP2 | PREDICTED: Bos taurus IQ motif containing GTPase activating protein 2 | cytoskeletal dynamics | -3.22 | -4.605 | -2.659 | 209 | 236 | 221 | 209 | 468 | 583 | 722 | 577 | 841 | 720 | 885 | 977 | 1183 |
| 12682652 | TMSB4 | Bos taurus thymosin beta 4, X-linked | cytoskeletal dynamics | -2.827 | -2.71 | -2.296 | 130 | 126 | 136 | 188 | 414 | 279 | 306 | 441 | 306 | 488 | 345 | 404 | 417 |
| 12787470 | MAP1B | Bos taurus microtubule-associated protein 1B | cytoskeletal dynamics | -2.506 | -2.39 | -2.67 | 214 | 251 | 418 | 270 | 803 | 791 | 652 | 687 | 733 | 680 | 728 | 581 | 702 |
| 12764377 | LASP1 | Bos taurus LIM and SH3 protein 1 | cytoskeletal dynamics | -2.376 | -2.374 | -2.621 | 205 | 196 | 184 | 184 | 554 | 480 | 481 | 456 | 491 | 426 | 449 | 539 | 393 |
| 12866116 | MYL6B | Bos taurus myosin, light chain 6B, alkali, smooth muscle and non-muscle | cytoskeletal dynamics | 2.292 | 8.753 | 2.255 | 210 | 272 | 298 | 222 | 144 | 96 | 96 | 123 | 90 | 115 | 32 | 39 | 18 |
| 12697659 | PLEK2 | Bos taurus pleckstrin 2 | cytoskeletal dynamics | 3.327 | 3.092 | 2.122 | 119 | 146 | 109 | 131 | 68 | 47 | 64 | 32 | 47 | 37 | 39 | 45 | 37 |
| 12710363 | SLAIN1 | SLAIN Motif Family, Member 1 | cytoskeletal dynamics | 3.65 | 3.121 | 3.654 | 268 | 260 | 254 | 326 | 96 | 56 | 80 | 71 | 90 | 67 | 92 | 88 | 84 |
| 12778245 | MYO1B | Bos taurus myosin IB | cytoskeletal dynamics | 3.754 | 5.436 | 2.286 | 1069 | 1125 | 1008 | 1189 | 489 | 466 | 482 | 313 | 270 | 294 | 171 | 192 | 248 |
| 12862938 | GAS2L3 | PREDICTED: Bos taurus growth arrest-specific 2 like 3 | cytoskeletal dynamics | 7.55 | 8.544 | 2.301 | 331 | 404 | 375 | 280 | 160 | 141 | 148 | 46 | 51 | 40 | 46 | 36 | 39 |
| 12711817 | CKAP2 | Bos taurus cytoskeleton associated protein 2 | cytoskeletal dynamics | 8.165 | 10.018 | 2.237 | 851 | 902 | 952 | 702 | 313 | 407 | 425 | 112 | 119 | 83 | 84 | 101 | 71 |
| 12709849 | DIAPH3 | Bos taurus diaphanous homolog 3 (Drosophila) | cytoskeletal dynamics | 8.298 | 7.903 | 2.6 | 151 | 164 | 152 | 150 | 72 | 54 | 54 | 18 | 18 | 20 | 22 | 20 | 17 |
| 12714352 | TPX2 | TPX2, Microtubule-Associated | cytoskeletal dynamics | 9.132 | 14.541 | 2.33 | 437 | 447 | 420 | 412 | 164 | 201 | 188 | 56 | 42 | 44 | 27 | 36 | 26 |
| 12781258 | OBSL1 | Bos taurus obscurin-like 1 | cytoskeletal dynamics | 9.958 | 9.019 | 2.131 | 447 | 399 | 488 | 347 | 251 | 170 | 175 | 39 | 50 | 38 | 48 | 52 | 40 |
| 12836295 | TPM3 | Bos taurus tropomyosin 3 | cytoskeletal dynamics | 13.664 | 7.812 | 2.285 | 437 | 402 | 439 | 406 | 157 | 234 | 169 | 42 | 41 | 17 | 71 | 25 | 87 |
| 12868434 | CCND2 | Bos taurus cyclin D2 | cytoskeleton | 9.126 | 28.661 | 3.091 | 1892 | 1935 | 2096 | 2781 | 796 | 616 | 685 | 256 | 227 | 225 | 77 | 78 | 70 |
| 12888488 | RAD50 | Bos taurus RAD50 homolog (S. cerevisiae) | DNA repair | 3.331 | 2.763 | 2.203 | 540 | 537 | 413 | 562 | 218 | 248 | 228 | 150 | 177 | 135 | 197 | 185 | 171 |
| 12809623 | RBBP8 | Bos taurus retinoblastoma binding protein 8 | DNA repair | 3.73 | 4.051 | 2.344 | 498 | 621 | 472 | 687 | 233 | 247 | 240 | 165 | 135 | 154 | 126 | 142 | 150 |
| 12795525 | RAD18 | Bos taurus RAD18 homolog (S. cerevisiae) | DNA repair | 4.047 | 3.562 | 3.35 | 547 | 614 | 466 | 481 | 157 | 141 | 173 | 121 | 139 | 128 | 161 | 139 | 143 |
| 12793233 | BLM | PREDICTED: Bos taurus Bloom syndrome, RecQ helicase-like | DNA repair | 4.295 | 3.581 | 2.51 | 255 | 263 | 201 | 206 | 89 | 86 | 100 | 45 | 62 | 54 | 66 | 51 | 78 |
| 12897842 | MMS22L | Bos taurus MMS22-like, DNA repair protein | DNA repair | 4.338 | 3.925 | 2.301 | 209 | 185 | 165 | 159 | 64 | 75 | 97 | 55 | 42 | 30 | 48 | 34 | 58 |
| 12860884 | RECQL | Bos taurus RecQ protein-like (DNA helicase Q1-like) | DNA repair | 4.537 | 3.474 | 2.181 | 279 | 332 | 242 | 271 | 119 | 123 | 143 | 62 | 71 | 53 | 61 | 90 | 94 |
| 12863730 | RAD51AP1 | Bos taurus RAD51 associated protein 1 | DNA repair | 4.801 | 4.699 | 2.357 | 150 | 155 | 121 | 142 | 67 | 59 | 54 | 32 | 32 | 25 | 24 | 39 | 29 |
| 12842304 | RAD54L | Bos taurus RAD54-like (S. cerevisiae) | DNA repair | 5.013 | 5.082 | 2.34 | 193 | 186 | 153 | 133 | 79 | 64 | 68 | 32 | 41 | 27 | 29 | 31 | 38 |
| 12765876 | BRIP1 | BRCA1 Interacting Protein C-Terminal Helicase 1 | DNA repair | 5.388 | 6.969 | 2.431 | 164 | 142 | 119 | 111 | 61 | 46 | 58 | 21 | 25 | 28 | 18 | 19 | 19 |
| 12689358 | RAD51 | Bos taurus RAD51 homolog (S. cerevisiae) | DNA repair | 5.981 | 5.527 | 2.576 | 293 | 282 | 212 | 167 | 100 | 68 | 109 | 42 | 44 | 32 | 50 | 40 | 37 |
| 12738902 | EXO1 | PREDICTED: Bos taurus exonuclease 1 | DNA repair | 6.175 | 7.606 | 3.212 | 123 | 118 | 88 | 103 | 42 | 24 | 37 | 19 | 17 | 16 | 11 | 15 | 17 |
| 12790238 | FANCI | Bos taurus Fanconi anemia, complementation group I | DNA repair | 8.07 | 10.619 | 2.885 | 411 | 370 | 378 | 296 | 123 | 118 | 134 | 57 | 46 | 34 | 35 | 38 | 30 |
| 12862370 | PARPBP | PARP1 Binding Protein | DNA repair | 8.916 | 9.285 | 3.88 | 157 | 160 | 142 | 150 | 44 | 33 | 42 | 15 | 20 | 16 | 18 | 20 | 12 |
| 12691481 | KIAA0101 | Bos taurus KIAA0101 ortholog | DNA repair | 11.727 | 9.835 | 2.087 | 356 | 309 | 262 | 218 | 190 | 101 | 128 | 24 | 23 | 26 | 29 | 37 | 22 |
| 12708565 | GEN1 | Bos taurus Gen homolog 1, endonuclease (Drosophila) | DNA repair | 12.6 | 8.574 | 2.676 | 233 | 234 | 170 | 157 | 87 | 63 | 71 | 14 | 21 | 13 | 24 | 21 | 23 |
| 12729324 | POLD3 | Bos taurus polymerase (DNA-directed), delta 3, accessory subunit | DNA replication | 2.207 | 2.62 | 2.027 | 202 | 204 | 176 | 173 | 88 | 96 | 95 | 79 | 90 | 87 | 75 | 60 | 82 |
| 12805984 | GMNN | Bos taurus geminin, DNA replication inhibitor | DNA replication | 2.241 | 2.015 | 2.819 | 130 | 148 | 102 | 113 | 39 | 37 | 56 | 52 | 50 | 62 | 52 | 78 | 55 |
| 12711883 | RFC3 | Bos taurus replication factor C (activator 1) 3, 38kDa | DNA replication | 2.409 | 2.142 | 2.446 | 252 | 259 | 263 | 337 | 118 | 106 | 115 | 106 | 121 | 117 | 131 | 116 | 141 |
| 12755361 | GINS2 | PREDICTED: Bos taurus GINS complex subunit 2 (Psf2 homolog), transcript variant 1 | DNA replication | 2.702 | 2.607 | 2.314 | 219 | 225 | 153 | 157 | 111 | 64 | 73 | 58 | 80 | 70 | 70 | 75 | 69 |
| 12724704 | DSCC1 | Bos taurus defective in sister chromatid cohesion 1 homolog (S. cerevisiae) | DNA replication | 3.298 | 2.167 | 2.405 | 104 | 107 | 101 | 110 | 46 | 42 | 44 | 37 | 29 | 31 | 51 | 59 | 39 |
| 12715895 | MCM10 | Bos taurus minichromosome maintenance complex component 10, mRNA (cDNA clone IMAGE:8183176), partial cds. | DNA replication | 3.533 | 3.957 | 2.325 | 300 | 325 | 270 | 242 | 124 | 97 | 149 | 93 | 77 | 72 | 115 | 36 | 87 |
| 12682028 | TOPBP1 | Bos taurus topoisomerase (DNA) II binding protein 1 | DNA replication | 3.539 | 2.728 | 2.032 | 431 | 423 | 301 | 352 | 177 | 195 | 179 | 100 | 131 | 89 | 146 | 134 | 130 |
| 12853992 | RPA3 | Bos taurus replication protein A3, 14kDa | DNA replication | 4.556 | 4.867 | 2.383 | 987 | 1005 | 654 | 1095 | 595 | 240 | 401 | 223 | 170 | 216 | 211 | 170 | 187 |
| 12858052 | PRIM1 | Bos taurus primase, DNA, polypeptide 1 (49kDa) | DNA replication | 5.448 | 4.643 | 2.645 | 475 | 515 | 350 | 354 | 157 | 166 | 150 | 77 | 75 | 77 | 70 | 97 | 107 |
| 12853829 | DBF4 | Bos taurus DBF4 homolog (S. cerevisiae) | DNA replication | 5.67 | 6.13 | 2.691 | 212 | 207 | 193 | 200 | 82 | 63 | 83 | 41 | 35 | 31 | 31 | 30 | 39 |
| 12790500 | TICRR | TOPBP1-interacting checkpoint and replication regulator (TICRR, C21H15orf42 ) | DNA replication | 5.727 | 6.649 | 2.737 | 116 | 133 | 110 | 91 | 44 | 37 | 41 | 22 | 19 | 18 | 16 | 16 | 18 |
| 12744811 | POLE | Bos taurus polymerase (DNA directed), epsilon, catalytic subunit | DNA replication | 5.832 | 5.183 | 2.205 | 205 | 191 | 142 | 102 | 81 | 57 | 74 | 31 | 27 | 22 | 28 | 25 | 39 |
| 12678992 | RFC4 | Bos taurus replication factor C (activator 1) 4, 37kDa | DNA replication | 5.926 | 4.956 | 2.778 | 532 | 456 | 333 | 391 | 172 | 124 | 164 | 74 | 77 | 63 | 86 | 91 | 78 |
| 12715654 | GINS1 | Bos taurus GINS complex subunit 1 (Psf1 homolog) | DNA replication | 6.208 | 5.889 | 2.4 | 416 | 431 | 380 | 316 | 176 | 138 | 167 | 71 | 68 | 49 | 70 | 66 | 60 |
| 12828817 | DNA2 | Bos taurus DNA replication helicase 2 homolog (yeast) | DNA replication | 6.751 | 7.666 | 3.181 | 137 | 148 | 106 | 105 | 39 | 38 | 39 | 17 | 19 | 18 | 18 | 13 | 18 |
| 12696877 | WDHD1 | Bos taurus WD repeat and HMG-box DNA binding protein 1 | DNA replication | 8.371 | 8.433 | 2.747 | 285 | 247 | 175 | 199 | 78 | 78 | 87 | 29 | 29 | 23 | 28 | 24 | 27 |
| 12685836 | POLQ | PREDICTED: Bos taurus polymerase (DNA directed), theta | DNA replication | 12.047 | 11.441 | 3.492 | 277 | 282 | 234 | 259 | 81 | 69 | 76 | 22 | 18 | 25 | 19 | 28 | 23 |
| 12761510 | TK1 | Bos taurus thymidine kinase 1, soluble | DNA replication | 16.764 | 20.076 | 5.076 | 813 | 842 | 966 | 595 | 204 | 136 | 137 | 61 | 39 | 44 | 50 | 42 | 29 |
| 12678716 | SYNJ1 | Bos taurus synaptojanin 1 | endocytosis | 2.504 | 2.39 | 2.243 | 264 | 316 | 357 | 376 | 160 | 152 | 125 | 127 | 130 | 132 | 123 | 150 | 137 |
| 12717971 | CST3 | Cystatin C | enzyme inhibitor | -2.637 | -4.142 | -4.037 | 327 | 373 | 595 | 241 | 1366 | 1761 | 1317 | 1023 | 999 | 864 | 1300 | 1793 | 1469 |
| 12759234 | PPP1R14A | Bos taurus protein phosphatase 1, regulatory (inhibitor) subunit 14A | enzyme inhibitor | 4.744 | 6.065 | 2.302 | 233 | 244 | 233 | 250 | 108 | 95 | 109 | 60 | 38 | 56 | 46 | 32 | 42 |
| 12769405 | VAT1 | Bos taurus vesicle amine transport protein 1 homolog (T. californica) | exocytosis | -2.713 | -2.866 | -3.244 | 111 | 105 | 153 | 102 | 374 | 433 | 331 | 342 | 328 | 279 | 378 | 351 | 279 |
| 12716247 | OPTN | Bos taurus optineurin | exocytosis | 2.701 | 3.126 | 2.592 | 895 | 1126 | 953 | 893 | 360 | 266 | 534 | 370 | 326 | 375 | 288 | 309 | 328 |
| 12822991 | RAB11FIP2 | Bos taurus RAB11 family interacting protein 2 (class I) | exocytosis | 3.074 | 3.375 | 2.55 | 312 | 319 | 302 | 410 | 154 | 133 | 109 | 109 | 111 | 105 | 91 | 113 | 94 |
| 12807534 | RAB27B | Bos taurus RAB27B, member RAS oncogene family | exocytosis | 6.367 | 8.689 | 2.866 | 319 | 430 | 255 | 373 | 128 | 116 | 111 | 62 | 56 | 43 | 40 | 24 | 63 |
| 12732926 | SYT9 | PREDICTED: Bos taurus synaptotagmin IX | exocytosis | 8.342 | 9.293 | 2.342 | 545 | 514 | 707 | 334 | 265 | 132 | 290 | 48 | 79 | 59 | 56 | 45 | 64 |
| 12854492 | LAMB1 | Bos taurus laminin, beta 1 | extracellular matrix | -8.147 | -10.536 | -10.514 | 164 | 152 | 180 | 86 | 1191 | 1782 | 1507 | 1140 | 1323 | 987 | 1210 | 1669 | 1594 |
| 12863901 | DCN | Bos taurus decorin | extracellular matrix | -7.717 | -6.056 | -22.552 | 148 | 162 | 181 | 94 | 2554 | 3522 | 3676 | 1327 | 920 | 1085 | 924 | 976 | 710 |
| 12679824 | COL6A1 | Bos taurus collagen, type VI, alpha 1 | extracellular matrix | -6.826 | -20.09 | -8.737 | 140 | 138 | 172 | 109 | 1232 | 1544 | 924 | 1182 | 1054 | 673 | 2461 | 3636 | 2389 |
| 12863917 | LUM | Bos taurus lumican | extracellular matrix | -5.233 | -5.174 | -6.416 | 1905 | 1633 | 523 | 247 | 4730 | 5332 | 5279 | 6373 | 5975 | 6121 | 6232 | 5859 | 6172 |
| 12883285 | SPARC | Bos taurus secreted protein, acidic, cysteine-rich (osteonectin) | extracellular matrix | -3.206 | -3.339 | -4.556 | 1203 | 1431 | 1434 | 1557 | 6055 | 6976 | 6146 | 5096 | 4448 | 3992 | 5140 | 4951 | 4017 |
| 12838041 | CTSK | Bos taurus cathepsin K | extracellular matrix | -2.532 | -3.666 | -2.677 | 253 | 245 | 321 | 197 | 655 | 656 | 701 | 636 | 723 | 554 | 945 | 915 | 893 |
| 12702441 | LOXL3 | Bos taurus lysyl oxidase-like 3 | extracellular matrix | 2.614 | 2.911 | 2.12 | 181 | 166 | 174 | 203 | 96 | 81 | 79 | 77 | 56 | 77 | 62 | 59 | 65 |
| 12763683 | NTN1 | Bos taurus netrin 1 | extracellular matrix | 3.747 | 5.543 | 2.072 | 381 | 360 | 250 | 354 | 162 | 158 | 161 | 93 | 89 | 84 | 57 | 75 | 50 |
| 12821682 | TLL2 | PREDICTED: Bos taurus tolloid-like 2, transcript variant 2 | extracellular matrix | 12.384 | 10.055 | 3.324 | 1673 | 1688 | 1714 | 1788 | 598 | 451 | 509 | 137 | 154 | 126 | 204 | 89 | 275 |
| 12873186 | HPSE | Heparanase | extracellular matrix | 36.719 | 34.166 | 7.921 | 412 | 386 | 267 | 455 | 41 | 47 | 54 | 13 | 9 | 9 | 9 | 11 | 13 |
| 12826119 | SRGN | Bos taurus serglycin | granule formation | 5.663 | 18.668 | 2.715 | 5427 | 6222 | 4866 | 7173 | 2934 | 1858 | 1841 | 1233 | 815 | 1101 | 354 | 346 | 252 |
| 12687885 | B2M | Bos taurus beta-2-microglobulin | immune response | -9.74 | -7.148 | -8.625 | 106 | 122 | 149 | 198 | 1622 | 773 | 1397 | 1265 | 1106 | 1803 | 806 | 1052 | 1175 |
| 12801783 | NM_001076841 | Bos taurus major histocompatibility complex, class I (LOC512672), mRNA. | immune response | -6.14 | -5.925 | -3.079 | 189 | 196 | 155 | 214 | 431 | 707 | 627 | 1122 | 1159 | 1166 | 1003 | 1247 | 1089 |
| 12749627 | FCGRT | Bos taurus Fc fragment of IgG, receptor, transporter, alpha | immune response | -3.115 | -2.36 | -3.539 | 189 | 171 | 205 | 129 | 697 | 533 | 596 | 557 | 498 | 544 | 439 | 367 | 408 |
| 12741927 | MIF | Bos taurus macrophage migration inhibitory factor (glycosylation-inhibiting factor) | immune response | 3.404 | 4.507 | 2.25 | 424 | 333 | 498 | 182 | 204 | 121 | 136 | 115 | 76 | 110 | 84 | 71 | 71 |
| 12681991 | TRIM59 | Bos taurus tripartite motif containing 59 | immune response | 3.767 | 3.936 | 3.151 | 243 | 316 | 263 | 259 | 88 | 79 | 90 | 66 | 75 | 74 | 78 | 54 | 76 |
| 12875578 | TLR1 | Bos taurus toll-like receptor 1 | immune response | 5.978 | 3.169 | 2.064 | 603 | 652 | 467 | 540 | 307 | 278 | 236 | 96 | 82 | 105 | 159 | 172 | 204 |
| 12739111 | SUSD4 | Bos taurus sushi domain containing 4 | immune response | 21.168 | 40.504 | 2.624 | 1137 | 1351 | 1078 | 1098 | 636 | 318 | 428 | 101 | 52 | 31 | 28 | 28 | 31 |
| 12782940 | CYBRD1 | Bos taurus cytochrome b reductase 1 | ion conversion | -2.404 | -2.402 | -4.81 | 115 | 150 | 183 | 167 | 575 | 941 | 719 | 353 | 401 | 343 | 377 | 359 | 358 |
| 12724789 | GRINA | Bos taurus glutamate receptor, ionotropic, N-methyl D-aspartate-associated protein 1 (glutamate binding) | ion transport | -2.125 | -2.485 | -2.029 | 174 | 168 | 182 | 182 | 380 | 380 | 317 | 351 | 392 | 383 | 483 | 474 | 368 |
| 12864163 | ATP2B1 | ATPase, Ca++ Transporting, Plasma Membrane 1 | ion transport | 2.066 | 2.697 | 2.268 | 787 | 808 | 672 | 819 | 316 | 339 | 363 | 337 | 395 | 387 | 257 | 283 | 319 |
| 12777737 | SCN9A | Bos taurus sodium channel, voltage-gated, type IX, alpha subunit | ion transport | 2.074 | 7.726 | 4.615 | 103 | 114 | 171 | 113 | 29 | 17 | 38 | 79 | 37 | 72 | 17 | 13 | 18 |
| 12908736 | SLC9A7 | PREDICTED: Bos taurus solute carrier family 9 (sodium/hydrogen exchanger), member 7 | ion transport | 2.383 | 6.686 | 2.176 | 186 | 211 | 346 | 278 | 123 | 108 | 111 | 124 | 74 | 122 | 35 | 34 | 42 |
| 12803127 | SLC25A27 | Bos taurus solute carrier family 25, member 27, nuclear gene encoding mitochondrial protein | ion transport | 2.405 | 2.291 | 2.089 | 147 | 173 | 174 | 221 | 89 | 76 | 90 | 69 | 81 | 71 | 86 | 69 | 78 |
| 12754114 | GRIN2D | Glutamate Receptor, Ionotropic, N-Methyl D-Aspartate 2D | ion transport | 2.511 | 2.237 | 2.06 | 143 | 92 | 137 | 73 | 58 | 46 | 53 | 43 | 45 | 41 | 41 | 48 | 56 |
| 12891839 | TMEM38B | Bos taurus transmembrane protein 38B | ion transport | 3.273 | 3.915 | 3.086 | 243 | 229 | 294 | 279 | 90 | 86 | 77 | 88 | 67 | 84 | 70 | 65 | 64 |
| 12845080 | LRRC8B | PREDICTED: Bos taurus leucine rich repeat containing 8 family, member B | ion transport | 3.397 | 2.13 | 3.4 | 241 | 306 | 175 | 282 | 79 | 80 | 60 | 91 | 60 | 69 | 110 | 135 | 104 |
| 12683494 | SLC5A3 | Bos taurus solute carrier family 5 (sodium/myo-inositol cotransporter), member 3 | ion transport | 4.314 | 7.151 | 2.052 | 3559 | 3519 | 3845 | 3323 | 2175 | 1489 | 1609 | 780 | 802 | 897 | 458 | 528 | 509 |
| 12683750 | TFRC | Bos taurus transferrin receptor (p90, CD71) | ion transport | 5.102 | 3.459 | 2.078 | 1133 | 1119 | 1078 | 1046 | 486 | 494 | 606 | 264 | 252 | 148 | 362 | 324 | 269 |
| 12688260 | KCNN2 | Potassium Channel, Calcium Activated Intermediate/Small Conductance Subfamily N Alpha, Member 2 | ion transport | 8.29 | 6.093 | 2.431 | 169 | 198 | 124 | 160 | 65 | 82 | 54 | 19 | 19 | 20 | 16 | 39 | 30 |
| 12883582 | KCNN1 | Potassium Channel, Calcium Activated Intermediate/Small Conductance Subfamily N Alpha, Member 1 | ion transport | 8.725 | 5.748 | 2.854 | 305 | 302 | 173 | 416 | 140 | 92 | 78 | 32 | 27 | 41 | 62 | 49 | 40 |
| 12851661 | KCND2 | Bos taurus potassium voltage-gated channel, Shal-related subfamily, member 2 | ion transport | 9.232 | 9.698 | 5.069 | 139 | 188 | 35 | 293 | 30 | 27 | 20 | 11 | 18 | 14 | 13 | 16 | 11 |
| 12859907 | ANO4 | Bos taurus anoctamin 4 | ion transport | 9.24 | 35.85 | 2.604 | 1055 | 1188 | 1163 | 1453 | 455 | 527 | 414 | 179 | 90 | 138 | 23 | 28 | 59 |
| 12821015 | SLC35G1 | Bos taurus solute carrier family 35, member G1 | ion transport | 17.766 | 44.269 | 2.369 | 2046 | 2026 | 1756 | 1966 | 896 | 625 | 987 | 131 | 82 | 122 | 63 | 26 | 52 |
| 12762062 | NLK | Bos taurus nemo-like kinase | kinase | 3.011 | 3.155 | 2.05 | 493 | 487 | 406 | 612 | 217 | 259 | 250 | 151 | 186 | 158 | 161 | 148 | 161 |
| 12904595 | RPS6KA6 | Bos taurus ribosomal protein S6 kinase, 90kDa, polypeptide 6 | kinase | 6.014 | 4.264 | 2.243 | 393 | 472 | 393 | 515 | 200 | 185 | 204 | 83 | 71 | 66 | 79 | 128 | 109 |
| 12767740 | AURKB | Bos taurus aurora kinase B | kinase | 6.788 | 6.913 | 2.449 | 231 | 242 | 217 | 173 | 84 | 73 | 108 | 28 | 36 | 31 | 39 | 24 | 32 |
| 12890559 | MELK | Bos taurus maternal embryonic leucine zipper kinase | kinase | 10.449 | 11.078 | 3.791 | 347 | 383 | 331 | 289 | 99 | 67 | 104 | 35 | 35 | 27 | 31 | 29 | 31 |
| 12891134 | PBK | Bos taurus PDZ binding kinase | kinase | 21.399 | 17.151 | 2.918 | 413 | 380 | 387 | 381 | 149 | 124 | 130 | 17 | 21 | 17 | 24 | 20 | 23 |
| 12889017 | LPL | Lipoprotein Lipase | lipid metabolism | -18.535 | -15.021 | -4.888 | 107 | 132 | 106 | 137 | 574 | 470 | 742 | 2272 | 1807 | 2660 | 1921 | 1446 | 2091 |
| 12829453 | TM7SF2 | Bos taurus transmembrane 7 superfamily member 2 | lipid metabolism | -11.553 | -13.44 | -3.501 | 154 | 138 | 135 | 64 | 751 | 323 | 279 | 1199 | 1480 | 1367 | 1769 | 1092 | 1978 |
| 12833215 | CPT1A | Carnitine Palmitoyltransferase 1A (Liver) | lipid metabolism | -6.556 | -3.557 | -3.42 | 104 | 98 | 145 | 55 | 321 | 311 | 341 | 651 | 688 | 536 | 302 | 315 | 403 |
| 12786629 | PPAP2A | Bos taurus phosphatidic acid phosphatase type 2A | lipid metabolism | -6.194 | -3.67 | -3.791 | 315 | 304 | 360 | 425 | 984 | 1477 | 1577 | 2081 | 2308 | 2080 | 1278 | 1375 | 1182 |
| 12742026 | SCARB1 | Bos taurus scavenger receptor class B, member 1 | lipid metabolism | -3.758 | -3.056 | -2.119 | 2140 | 1779 | 1395 | 897 | 3355 | 2854 | 3207 | 5744 | 5577 | 5344 | 5013 | 3577 | 5136 |
| 12866155 | CYB5R3 | Bos taurus cytochrome b5 reductase 3 | lipid metabolism | -3.278 | -4.591 | -5.957 | 811 | 742 | 745 | 517 | 4463 | 4463 | 3549 | 2263 | 2504 | 2080 | 3064 | 3111 | 3396 |
| 12704087 | PTGDS | Prostaglandin D2 Synthase 21kDa (Brain) | lipid metabolism | -3.137 | -3.576 | -3.124 | 226 | 195 | 250 | 131 | 583 | 894 | 435 | 764 | 420 | 716 | 742 | 487 | 942 |
| 12790479 | DNAJA4 | Bos taurus DnaJ (Hsp40) homolog, subfamily A, member 4 | lipid metabolism | -2.838 | -2.072 | 2.319 | 158 | 157 | 157 | 144 | 80 | 55 | 66 | 413 | 494 | 408 | 268 | 317 | 382 |
| 12824231 | AGPAT5 | Bos taurus 1-acylglycerol-3-phosphate O-acyltransferase 5 (lysophosphatidic acid acyltransferase, epsilon) | lipid metabolism | 2.154 | 2.408 | 2.07 | 196 | 205 | 184 | 247 | 122 | 99 | 82 | 104 | 97 | 88 | 102 | 80 | 77 |
| 12833760 | CHKA | Choline Kinase Alpha | lipid metabolism | 2.223 | 3.064 | 2.718 | 266 | 324 | 292 | 354 | 121 | 101 | 117 | 150 | 114 | 154 | 117 | 70 | 124 |
| 12808023 | B4GALT6 | Bos taurus UDP-Gal:betaGlcNAc beta 1,4- galactosyltransferase, polypeptide 6 | lipid metabolism | 27.459 | 25.863 | 3.813 | 819 | 881 | 637 | 839 | 248 | 165 | 216 | 29 | 32 | 25 | 25 | 27 | 42 |
| 12841278 | TMEM48 | NDC1 Transmembrane Nucleoporin | membrane structure | 5.6 | 4.003 | 2.137 | 400 | 368 | 347 | 390 | 173 | 162 | 194 | 66 | 70 | 65 | 97 | 94 | 91 |
| 12907723 | SAT1 | Bos taurus spermidine/spermine N1-acetyltransferase 1 | metabolism | -14.537 | -14.005 | -3.629 | 215 | 215 | 312 | 264 | 1017 | 878 | 821 | 3411 | 3720 | 3714 | 3251 | 3331 | 3892 |
| 12705521 | CRAT | Bos taurus carnitine O-acetyltransferase, nuclear gene encoding mitochondrial protein | metabolism | -4.166 | -3.325 | -2.308 | 460 | 487 | 495 | 363 | 1253 | 882 | 1000 | 2036 | 1776 | 1797 | 1880 | 1341 | 1310 |
| 12702548 | RETSAT | Bos taurus retinol saturase (all-trans-retinol 13,14-reductase) | metabolism | -4.085 | -3.526 | -2.506 | 104 | 113 | 153 | 113 | 309 | 302 | 285 | 456 | 496 | 510 | 494 | 377 | 398 |
| 12891041 | PSAT1 | Bos taurus phosphoserine aminotransferase 1 | metabolism | -3.808 | -6.729 | -4.864 | 178 | 174 | 83 | 52 | 579 | 548 | 449 | 442 | 441 | 351 | 853 | 663 | 667 |
| 12799840 | ALAS1 | 5'-Aminolevulinate Synthase 1 | metabolism | -3.716 | -3.168 | -3.703 | 141 | 134 | 165 | 180 | 542 | 419 | 814 | 601 | 586 | 531 | 642 | 434 | 415 |
| 12893648 | FBP1 | Bos taurus fructose-1,6-bisphosphatase 1 | metabolism | -3.321 | 3.535 | 2.455 | 109 | 122 | 121 | 149 | 51 | 34 | 75 | 641 | 203 | 539 | 36 | 23 | 52 |
| 12882320 | GALNT10 | PREDICTED: Bos taurus UDP-N-acetyl-alpha-D-galactosamine:polypeptide N-acetylgalactosaminyltransferase 10 (GalNAc-T10) | metabolism | -2.92 | -2.693 | -2.973 | 167 | 155 | 189 | 102 | 507 | 457 | 378 | 440 | 483 | 391 | 469 | 355 | 391 |
| 12908534 | HS6ST2 | Bos taurus heparan sulfate 6-O-sulfotransferase 2 | metabolism | -2.168 | 3.822 | 3.047 | 221 | 244 | 195 | 167 | 90 | 45 | 74 | 517 | 322 | 524 | 67 | 41 | 55 |
| 12748964 | PDP2 | PREDICTED: Bos taurus pyruvate dehyrogenase phosphatase catalytic subunit 2 | metabolism | 2.323 | 2.349 | 3.228 | 289 | 307 | 303 | 305 | 93 | 80 | 109 | 157 | 104 | 133 | 158 | 105 | 127 |
| 12845397 | HS2ST1 | Heparan Sulfate 2-O-Sulfotransferase 1 | metabolism | 2.357 | 2.553 | 2.571 | 984 | 985 | 1164 | 1065 | 422 | 416 | 384 | 496 | 441 | 400 | 461 | 399 | 375 |
| 12838558 | B4GALT3 | UDP-Gal:BetaGlcNAc Beta 1,4- Galactosyltransferase, Polypeptide 3 | metabolism | 2.517 | 2.434 | 2.031 | 331 | 340 | 273 | 280 | 185 | 121 | 150 | 129 | 116 | 118 | 131 | 126 | 119 |
| 12725482 | GGH | Bos taurus gamma-glutamyl hydrolase (conjugase, folylpolygammaglutamyl hydrolase) | metabolism | 2.73 | 4.316 | 2.861 | 174 | 180 | 149 | 221 | 63 | 57 | 68 | 80 | 49 | 72 | 40 | 37 | 48 |
| 12871503 | DCK | Bos taurus deoxycytidine kinase | metabolism | 2.848 | 3.136 | 2.713 | 391 | 398 | 346 | 319 | 123 | 143 | 135 | 112 | 137 | 134 | 123 | 103 | 121 |
| 12722987 | AZIN1 | Bos taurus antizyme inhibitor 1 | metabolism | 2.901 | 2.302 | 2.374 | 1164 | 1199 | 1117 | 1287 | 528 | 474 | 503 | 334 | 483 | 428 | 446 | 536 | 578 |
| 12905259 | PRPS2 | Bos taurus phosphoribosyl pyrophosphate synthetase 2 | metabolism | 2.926 | 2.656 | -2.35 | 354 | 334 | 271 | 681 | 985 | 915 | 819 | 117 | 149 | 130 | 131 | 138 | 168 |
| 12787041 | NDUFS4 | Bos taurus NADH dehydrogenase (ubiquinone) Fe-S protein 4, 18kDa (NADH-coenzyme Q reductase), nuclear gene encoding mitochondrial protein | metabolism | 3.03 | 3.804 | 2.104 | 2369 | 2570 | 2267 | 3223 | 1513 | 1019 | 1198 | 864 | 764 | 938 | 784 | 632 | 632 |
| 12867044 | OSBPL8 | Oxysterol Binding Protein-Like 8 | metabolism | 3.089 | 2.966 | 2.07 | 1254 | 1312 | 759 | 1051 | 495 | 515 | 542 | 340 | 352 | 348 | 377 | 367 | 340 |
| 12786382 | AMACR | Bos taurus alpha-methylacyl-CoA racemase, mRNA (cDNA clone IMAGE:8284762), partial cds. | metabolism | 3.463 | 4.468 | 3.083 | 300 | 313 | 490 | 401 | 111 | 117 | 131 | 99 | 95 | 128 | 80 | 85 | 82 |
| 12891282 | FKTN | PREDICTED: Bos taurus fukutin | metabolism | 3.488 | 2.949 | 2.252 | 453 | 501 | 429 | 480 | 181 | 195 | 250 | 145 | 126 | 129 | 152 | 143 | 180 |
| 12770824 | AFMID | Bos taurus arylformamidase | metabolism | 4.537 | 4.003 | 3.264 | 175 | 172 | 232 | 135 | 62 | 58 | 42 | 38 | 31 | 48 | 39 | 43 | 49 |
| 12789104 | IDH3A | Isocitrate Dehydrogenase 3 (NAD+) Alpha | metabolism | 4.809 | 6.947 | 2.568 | 1338 | 1867 | 2181 | 1958 | 730 | 594 | 804 | 377 | 328 | 429 | 322 | 217 | 251 |
| 12801746 | GCLC | Bos taurus glutamate-cysteine ligase, catalytic subunit | metabolism | 5.442 | 4.5 | 2.211 | 3814 | 3940 | 3768 | 3945 | 2026 | 1774 | 1486 | 658 | 614 | 887 | 732 | 1068 | 810 |
| 12785671 | CMBL | Bos taurus carboxymethylenebutenolidase homolog (Pseudomonas) | metabolism | 6.04 | 4.562 | 2.425 | 576 | 617 | 457 | 664 | 361 | 197 | 186 | 90 | 94 | 101 | 150 | 106 | 125 |
| 12820687 | STAMBPL1 | Bos taurus STAM binding protein-like 1 | metabolism | 6.137 | 7.089 | 4.39 | 509 | 496 | 334 | 389 | 133 | 83 | 83 | 63 | 71 | 74 | 56 | 73 | 52 |
| 12889669 | GLDC | Bos taurus glycine dehydrogenase (decarboxylating), nuclear gene encoding mitochondrial protein | metabolism | 6.681 | 12.178 | 4.79 | 318 | 336 | 340 | 340 | 73 | 60 | 76 | 63 | 39 | 50 | 23 | 28 | 32 |
| 12725857 | GPT | Bos taurus glutamic-pyruvate transaminase (alanine aminotransferase) | metabolism | 7.54 | 5.754 | 2.757 | 225 | 213 | 211 | 270 | 112 | 63 | 81 | 32 | 28 | 31 | 43 | 34 | 42 |
| 12718907 | PDSS1 | Bos taurus prenyl (decaprenyl) diphosphate synthase, subunit 1 | metabolism | 18.997 | 19.085 | 3.608 | 778 | 890 | 1120 | 1197 | 309 | 237 | 275 | 58 | 57 | 42 | 47 | 52 | 56 |
| 12908260 | CA5B | Bos taurus carbonic anhydrase VB, mitochondrial, nuclear gene encoding mitochondrial protein | mitochondrial function | 4.976 | 4.986 | 2.566 | 183 | 189 | 318 | 184 | 95 | 73 | 82 | 55 | 39 | 37 | 50 | 39 | 39 |
| 12686832 | MRPS6 | Bos taurus mitochondrial ribosomal protein S6, nuclear gene encoding mitochondrial protein | mitochondrial function | 5.314 | 6.342 | 2.264 | 1602 | 1729 | 1724 | 1856 | 854 | 719 | 721 | 316 | 309 | 351 | 280 | 254 | 283 |
| 12900926 | FAM54A | Mitochondrial Fission Regulator 2 (MTFR2). Formerly Bos taurus family with sequence similarity 54, member A | mitochondrial function | 8.955 | 9.607 | 3.142 | 500 | 480 | 410 | 324 | 178 | 120 | 114 | 39 | 52 | 51 | 42 | 43 | 47 |
| 12806571 | CCND3 | Bos taurus cyclin D3 | mitosis | -3.215 | -4.709 | -2.209 | 260 | 220 | 245 | 197 | 545 | 443 | 538 | 762 | 918 | 572 | 1325 | 1062 | 894 |
| 12868668 | CCDC41 | Bos taurus coiled-coil domain containing 41 | mitosis | 2.214 | 2.031 | 2.968 | 177 | 197 | 173 | 175 | 62 | 54 | 67 | 73 | 82 | 90 | 92 | 70 | 108 |
| 12685024 | CEP63 | Centrosomal Protein 63kDa | mitosis | 2.292 | 2.155 | 2.04 | 130 | 166 | 151 | 147 | 66 | 86 | 67 | 55 | 60 | 81 | 77 | 59 | 71 |
| 12894933 | DBC1 | Bone Morphogenetic Protein/Retinoic Acid Inducible Neural-Specific 1, or DBC1 | mitosis | 2.378 | 4.046 | 3.414 | 106 | 147 | 126 | 96 | 34 | 34 | 35 | 51 | 47 | 49 | 30 | 28 | 29 |
| 12870891 | CCNG2 | Bos taurus cDNA clone IMAGE:8475492. | mitosis | 2.391 | 2.399 | 2.029 | 157 | 138 | 124 | 203 | 86 | 79 | 63 | 62 | 64 | 65 | 61 | 64 | 67 |
| 12733781 | C2CD3 | Bos taurus C2 calcium-dependent domain containing 3 | mitosis | 2.46 | 2.483 | 2.075 | 354 | 400 | 344 | 381 | 180 | 163 | 192 | 170 | 149 | 134 | 144 | 150 | 153 |
| 12891363 | CEP78 | PREDICTED: Bos taurus centrosomal protein 78kDa | mitosis | 2.542 | 2.615 | 2.177 | 110 | 113 | 91 | 105 | 51 | 43 | 51 | 51 | 38 | 36 | 47 | 39 | 35 |
| 12795899 | CDC25A | Bos taurus cell division cycle 25 homolog A (S. pombe) | mitosis | 2.545 | 3.004 | 2.488 | 149 | 181 | 180 | 158 | 71 | 62 | 68 | 79 | 62 | 57 | 68 | 45 | 55 |
| 12858199 | LRIG3 | Bos taurus leucine-rich repeats and immunoglobulin-like domains 3 | mitosis | 2.703 | 11.724 | 2.274 | 274 | 289 | 420 | 350 | 164 | 133 | 138 | 129 | 87 | 159 | 18 | 33 | 38 |
| 12843388 | CDKN2C | Bos taurus cyclin-dependent kinase inhibitor 2C (p18, inhibits CDK4) | mitosis | 2.706 | 2.293 | 2.247 | 333 | 338 | 365 | 308 | 169 | 144 | 136 | 107 | 158 | 113 | 107 | 164 | 177 |
| 12682007 | CEP97 | Bos taurus centrosomal protein 97kDa | mitosis | 2.793 | 2.594 | 2.041 | 199 | 211 | 237 | 209 | 94 | 104 | 119 | 68 | 74 | 89 | 74 | 86 | 88 |
| 12859994 | NEDD1 | Bos taurus neural precursor cell expressed, developmentally down-regulated 1 | mitosis | 2.823 | 3.488 | 2.256 | 238 | 256 | 250 | 261 | 109 | 102 | 125 | 108 | 72 | 90 | 68 | 73 | 75 |
| 12735189 | NSL1 | Bos taurus NSL1, MIND kinetochore complex component, homolog (S. cerevisiae) | mitosis | 2.834 | 2.738 | 2.152 | 152 | 161 | 144 | 152 | 80 | 64 | 69 | 54 | 61 | 47 | 70 | 46 | 53 |
| 12853903 | CDK6 | Bos taurus cyclin-dependent kinase 6 | mitosis | 3.01 | 4.147 | 2.126 | 380 | 414 | 516 | 385 | 233 | 175 | 190 | 130 | 143 | 147 | 101 | 113 | 92 |
| 12836403 | SASS6 | Bos taurus spindle assembly 6 homolog (C. elegans) | mitosis | 3.215 | 2.858 | 2.188 | 206 | 254 | 234 | 231 | 104 | 105 | 108 | 57 | 77 | 84 | 79 | 79 | 84 |
| 12866416 | CDK2 | Bos taurus cyclin-dependent kinase 2 | mitosis | 3.31 | 4.305 | 2.008 | 316 | 273 | 256 | 182 | 126 | 121 | 129 | 76 | 77 | 75 | 55 | 60 | 61 |
| 12819682 | ZWINT | Bos taurus ZW10 interactor | mitosis | 3.325 | 3.381 | 2.153 | 288 | 218 | 153 | 157 | 110 | 79 | 89 | 53 | 72 | 55 | 55 | 77 | 47 |
| 12725792 | CCNE2 | Bos taurus cyclin E2 | mitosis | 3.348 | 6.207 | 2.728 | 556 | 488 | 478 | 453 | 190 | 158 | 197 | 180 | 108 | 164 | 75 | 65 | 103 |
| 12847375 | CDC14A | Cell Division Cycle 14A | mitosis | 3.452 | 4.189 | 2.33 | 764 | 733 | 673 | 760 | 348 | 305 | 292 | 193 | 224 | 220 | 171 | 188 | 166 |
| 12777652 | SGOL2 | Bos taurus shugoshin-like 2 (S. pombe) | mitosis | 3.468 | 4.995 | 2.206 | 244 | 332 | 263 | 317 | 125 | 132 | 133 | 74 | 73 | 104 | 43 | 71 | 61 |
| 12894072 | REEP4 | Bos taurus receptor accessory protein 4 | mitosis | 3.675 | 3.641 | 2.186 | 202 | 210 | 206 | 191 | 107 | 85 | 87 | 57 | 67 | 44 | 58 | 56 | 52 |
| 12903846 | CENPI | Bos taurus centromere protein I | mitosis | 3.68 | 3.396 | 2.114 | 161 | 160 | 155 | 154 | 77 | 65 | 82 | 40 | 49 | 40 | 46 | 50 | 44 |
| 12718797 | DSN1 | Bos taurus DSN1, MIND kinetochore complex component, homolog (S. cerevisiae) | mitosis | 3.978 | 4.529 | 2.81 | 147 | 159 | 157 | 145 | 60 | 46 | 57 | 31 | 42 | 43 | 42 | 30 | 30 |
| 12733232 | WEE1 | Bos taurus WEE1 homolog (S. pombe) | mitosis | 3.986 | 2.985 | 2.14 | 694 | 773 | 656 | 557 | 284 | 332 | 318 | 164 | 205 | 138 | 228 | 228 | 213 |
| 12683595 | SMC4 | Bos taurus structural maintenance of chromosomes 4, mRNA (cDNA clone MGC:140334 IMAGE:8187023), complete cds. | mitosis | 4.028 | 3.985 | 2.69 | 609 | 753 | 533 | 660 | 234 | 217 | 258 | 149 | 164 | 159 | 152 | 156 | 170 |
| 12870366 | MAD2L1 | Bos taurus MAD2 mitotic arrest deficient-like 1 (yeast) | mitosis | 4.183 | 5.003 | 2.101 | 118 | 133 | 171 | 176 | 82 | 63 | 67 | 19 | 50 | 46 | 23 | 38 | 29 |
| 12709672 | BORA | Bos taurus bora, aurora kinase A activator | mitosis | 4.303 | 3.339 | 2.568 | 201 | 208 | 210 | 262 | 70 | 96 | 93 | 58 | 50 | 45 | 56 | 63 | 80 |
| 12744211 | ANAPC5 | Bos taurus anaphase promoting complex subunit 5 | mitosis | 4.333 | 2.758 | 2.19 | 1080 | 1107 | 1163 | 976 | 524 | 495 | 462 | 291 | 283 | 187 | 404 | 445 | 334 |
| 12772448 | FAM64A | Bos taurus family with sequence similarity 64, member A | mitosis | 4.658 | 4.774 | 2.06 | 155 | 152 | 186 | 95 | 78 | 67 | 63 | 24 | 33 | 36 | 24 | 32 | 34 |
| 12708398 | CENPO | Bos taurus centromere protein O | mitosis | 4.696 | 4.599 | 2.359 | 347 | 334 | 337 | 299 | 172 | 97 | 162 | 78 | 68 | 65 | 79 | 68 | 68 |
| 12854230 | NCAPG2 | Bos taurus non-SMC condensin II complex, subunit G2 | mitosis | 4.702 | 4.947 | 2.205 | 267 | 271 | 270 | 193 | 129 | 98 | 112 | 54 | 61 | 44 | 52 | 48 | 50 |
| 12906051 | ERCC6L | Bos taurus excision repair cross-complementing rodent repair deficiency, complementation group 6-like | mitosis | 4.717 | 4.624 | 2.432 | 164 | 176 | 154 | 124 | 71 | 51 | 69 | 46 | 28 | 27 | 36 | 26 | 39 |
| 12858506 | GTSE1 | Bos taurus G-2 and S-phase expressed 1 | mitosis | 5.116 | 4.601 | 2.214 | 121 | 140 | 115 | 81 | 59 | 46 | 48 | 29 | 17 | 22 | 29 | 21 | 23 |
| 12895589 | CKS2 | CDC28 Protein Kinase Regulatory Subunit 2 | mitosis | 5.142 | 4.114 | 2.122 | 321 | 324 | 321 | 308 | 161 | 120 | 176 | 63 | 64 | 59 | 83 | 85 | 65 |
| 12837338 | CLSPN | Bos taurus claspin | mitosis | 5.325 | 4.504 | 3.154 | 110 | 114 | 80 | 64 | 31 | 31 | 25 | 15 | 19 | 16 | 16 | 23 | 21 |
| 12791266 | VRK1 | Bos taurus vaccinia related kinase 1 | mitosis | 5.367 | 5.877 | 2.959 | 467 | 446 | 386 | 437 | 181 | 107 | 162 | 92 | 80 | 71 | 84 | 60 | 80 |
| 12736304 | NEK2 | Bos taurus NIMA (never in mitosis gene a)-related kinase 2 | mitosis | 5.375 | 4.946 | 2.771 | 101 | 109 | 106 | 96 | 43 | 29 | 40 | 26 | 14 | 19 | 21 | 16 | 26 |
| 12870714 | CEP135 | Bos taurus centrosomal protein 135kDa | mitosis | 5.671 | 4.301 | 3.585 | 142 | 165 | 153 | 150 | 51 | 35 | 43 | 25 | 33 | 24 | 37 | 42 | 29 |
| 12718493 | CDC25B | Bos taurus cell division cycle 25 homolog B (S. pombe) | mitosis | 5.735 | 6.418 | 2.473 | 320 | 345 | 355 | 262 | 130 | 132 | 124 | 52 | 75 | 44 | 55 | 61 | 36 |
| 12815755 | KIF22 | Bos taurus kinesin family member 22 | mitosis | 5.755 | 5.761 | 2.42 | 151 | 161 | 153 | 117 | 66 | 55 | 59 | 26 | 24 | 25 | 32 | 17 | 28 |
| 12846146 | KIF2C | Bos taurus kinesin family member 2C | mitosis | 5.807 | 7.126 | 2.271 | 121 | 130 | 129 | 98 | 54 | 51 | 52 | 17 | 28 | 18 | 19 | 15 | 16 |
| 12709472 | SKA3 | Bos taurus spindle and kinetochore associated complex subunit 3 | mitosis | 6.629 | 5.26 | 2.222 | 331 | 334 | 335 | 269 | 132 | 149 | 146 | 37 | 54 | 54 | 66 | 55 | 60 |
| 12689529 | KIF23 | Bos taurus kinesin family member 23 | mitosis | 6.998 | 5.558 | 2.636 | 432 | 463 | 453 | 338 | 159 | 160 | 156 | 64 | 71 | 47 | 82 | 84 | 62 |
| 12785624 | CCDC99 | Spindle Apparatus Coiled-Coil Protein 1 | mitosis | 8.147 | 9.933 | 3.1 | 134 | 118 | 147 | 139 | 44 | 35 | 53 | 15 | 17 | 17 | 11 | 17 | 13 |
| 12692009 | NUSAP1 | Bos taurus nucleolar and spindle associated protein 1 | mitosis | 8.169 | 5.995 | 3.574 | 165 | 137 | 155 | 137 | 55 | 31 | 41 | 14 | 26 | 16 | 17 | 29 | 32 |
| 12819693 | KIF20B | Bos taurus kinesin family member 20B | mitosis | 8.224 | 5.111 | 2.894 | 107 | 146 | 115 | 131 | 48 | 39 | 42 | 12 | 17 | 18 | 42 | 22 | 15 |
| 12686668 | SGOL1 | Bos taurus shugoshin-like 1 (S. pombe) | mitosis | 9.725 | 9.436 | 3.068 | 390 | 415 | 329 | 377 | 130 | 124 | 115 | 38 | 37 | 42 | 42 | 24 | 62 |
| 12775328 | SPC25 | Bos taurus SPC25, NDC80 kinetochore complex component, homolog (S. cerevisiae) | mitosis | 10.039 | 10.315 | 3.723 | 158 | 235 | 138 | 177 | 60 | 37 | 45 | 21 | 17 | 14 | 17 | 14 | 20 |
| 12714015 | AURKA | Bos taurus aurora kinase A | mitosis | 10.727 | 9.019 | 2.925 | 433 | 459 | 417 | 420 | 154 | 119 | 175 | 40 | 40 | 40 | 55 | 41 | 48 |
| 12792052 | MIS18BP1 | Bos taurus MIS18 binding protein 1 | mitosis | 11.318 | 10.162 | 4.016 | 441 | 470 | 395 | 467 | 128 | 103 | 102 | 36 | 37 | 44 | 54 | 32 | 48 |
| 12698951 | NCAPH | Bos taurus non-SMC condensin I complex, subunit H | mitosis | 11.331 | 12.117 | 2.735 | 512 | 513 | 494 | 307 | 191 | 143 | 159 | 43 | 34 | 42 | 36 | 36 | 38 |
| 12894921 | ESCO2 | Bos taurus establishment of cohesion 1 homolog 2 (S. cerevisiae) | mitosis | 12.212 | 14.787 | 4.394 | 501 | 565 | 430 | 390 | 124 | 84 | 115 | 44 | 35 | 36 | 37 | 26 | 33 |
| 12700024 | BUB1 | Bos taurus budding uninhibited by benzimidazoles 1 homolog (yeast) | mitosis | 12.246 | 9.418 | 2.681 | 285 | 311 | 277 | 216 | 105 | 84 | 116 | 22 | 28 | 17 | 31 | 23 | 33 |
| 12740427 | ASPM | Bos taurus asp (abnormal spindle) homolog, microcephaly associated (Drosophila) | mitosis | 13.292 | 10.774 | 2.592 | 362 | 383 | 374 | 353 | 144 | 141 | 140 | 24 | 29 | 31 | 23 | 32 | 55 |
| 12746604 | PLK4 | Bos taurus polo-like kinase 4 | mitosis | 14.089 | 14.527 | 3.583 | 662 | 696 | 612 | 639 | 198 | 141 | 215 | 47 | 52 | 40 | 45 | 46 | 44 |
| 12716738 | MASTL | Bos taurus microtubule associated serine/threonine kinase-like | mitosis | 14.295 | 7.462 | 2.61 | 195 | 215 | 160 | 177 | 70 | 63 | 82 | 12 | 10 | 18 | 21 | 24 | 30 |
| 12791280 | PRC1 | Bos taurus protein regulator of cytokinesis 1 | mitosis | 14.668 | 15.006 | 3.451 | 467 | 529 | 595 | 475 | 161 | 132 | 155 | 44 | 36 | 28 | 39 | 36 | 28 |
| 12796835 | KIF15 | Bos taurus kinesin family member 15 | mitosis | 15.779 | 11.816 | 3.526 | 291 | 285 | 215 | 236 | 77 | 62 | 78 | 17 | 18 | 14 | 23 | 23 | 19 |
| 12704554 | CKAP2L | Bos taurus cytoskeleton associated protein 2-like | mitosis | 16.375 | 13.889 | 3.623 | 521 | 505 | 500 | 461 | 134 | 129 | 149 | 27 | 29 | 35 | 33 | 42 | 33 |
| 12692627 | CASC5 | PREDICTED: Bos taurus cancer susceptibility candidate 5 | mitosis | 16.466 | 18.685 | 3.45 | 339 | 432 | 304 | 387 | 119 | 103 | 94 | 18 | 26 | 23 | 18 | 23 | 18 |
| 12909607 | KIF4A | Bos taurus kinesin family member 4A | mitosis | 17.025 | 20.859 | 3.425 | 296 | 308 | 277 | 304 | 84 | 83 | 92 | 18 | 21 | 14 | 15 | 13 | 14 |
| 12808472 | NDC80 | Bos taurus NDC80 homolog, kinetochore complex component (S. cerevisiae) | mitosis | 18.001 | 22.866 | 4.465 | 354 | 373 | 354 | 357 | 84 | 77 | 81 | 15 | 21 | 25 | 18 | 15 | 14 |
| 12871525 | NCAPG | Non-SMC Condensin I Complex, Subunit G | mitosis | 18.625 | 21.477 | 3.464 | 647 | 656 | 441 | 599 | 173 | 153 | 177 | 39 | 28 | 27 | 31 | 24 | 26 |
| 12845106 | NUF2 | Bos taurus NUF2, NDC80 kinetochore complex component, homolog (S. cerevisiae) | mitosis | 18.698 | 14.494 | 3.931 | 346 | 342 | 327 | 346 | 110 | 72 | 82 | 21 | 19 | 16 | 24 | 23 | 23 |
| 12690910 | BUB1B | Bos taurus budding uninhibited by benzimidazoles 1 homolog beta (yeast) | mitosis | 19.301 | 18.331 | 2.612 | 334 | 418 | 440 | 388 | 158 | 134 | 161 | 23 | 20 | 19 | 19 | 26 | 20 |
| 12745867 | KNTC1 | Bos taurus kinetochore associated 1 | mitosis | 19.647 | 19.328 | 3.738 | 387 | 400 | 292 | 261 | 99 | 75 | 93 | 18 | 19 | 14 | 19 | 16 | 16 |
| 12822057 | MKI67 | Marker Of Proliferation Ki-67 | mitosis | 21.567 | 18.552 | 2.419 | 613 | 595 | 605 | 452 | 274 | 209 | 218 | 26 | 29 | 23 | 28 | 35 | 28 |
| 12732959 | KIF18A | Bos taurus kinesin family member 18A | mitosis | 23.555 | 18.267 | 5.023 | 478 | 463 | 314 | 367 | 86 | 65 | 90 | 21 | 17 | 14 | 26 | 22 | 19 |
| 12819342 | KIF11 | Bos taurus kinesin family member 11 | mitosis | 26.424 | 25.881 | 3.557 | 590 | 683 | 579 | 532 | 174 | 168 | 160 | 26 | 18 | 24 | 20 | 25 | 24 |
| 12822921 | SLC16A12 | Solute Carrier Family 16, Member 12 | molecular transport | -9.6 | -5.555 | 3.841 | 122 | 115 | 80 | 117 | 29 | 20 | 36 | 1100 | 912 | 1083 | 834 | 459 | 549 |
| 12874187 | APBB2 | Bos taurus amyloid beta (A4) precursor protein-binding, family B, member 2 | molecular transport | -6.883 | -5.053 | -2.677 | 191 | 219 | 230 | 156 | 425 | 643 | 534 | 1477 | 1252 | 1339 | 1020 | 939 | 1024 |
| 12825466 | SLC7A2 | PREDICTED: Bos taurus solute carrier family 7 (cationic amino acid transporter, y+ system), member 2 | molecular transport | -3.853 | -3.743 | -2.483 | 107 | 112 | 202 | 155 | 339 | 367 | 330 | 585 | 485 | 540 | 463 | 426 | 711 |
| 12903879 | NXT2 | Bos taurus nuclear transport factor 2-like export factor 2 | molecular transport | 2.624 | 3.601 | 2 | 224 | 214 | 149 | 324 | 137 | 82 | 117 | 83 | 80 | 88 | 58 | 58 | 68 |
| 12761534 | NUP88 | Bos taurus nucleoporin 88kDa | molecular transport | 2.945 | 2.106 | 2.078 | 287 | 325 | 288 | 272 | 126 | 155 | 142 | 88 | 95 | 118 | 128 | 138 | 152 |
| 12904075 | ABCD1 | Bos taurus ATP-binding cassette, sub-family D (ALD), member 1 | molecular transport | 3.036 | 4.528 | 2.247 | 367 | 356 | 742 | 388 | 235 | 170 | 188 | 161 | 126 | 151 | 122 | 87 | 87 |
| 12800256 | SYNPR | Synaptoporin | molecular transport | 4.111 | 3.818 | 2.335 | 191 | 230 | 179 | 296 | 115 | 89 | 81 | 56 | 58 | 47 | 64 | 55 | 54 |
| 12791856 | SLCO3A1 | Bos taurus solute carrier organic anion transporter family, member 3A1 | molecular transport | 5.013 | 10.665 | 3.225 | 1110 | 1003 | 960 | 735 | 328 | 329 | 230 | 178 | 208 | 179 | 91 | 65 | 116 |
| 12790703 | UNC79 | Unc-79 Homolog | molecular transport | 5.296 | 6.417 | 2.562 | 107 | 100 | 165 | 110 | 41 | 40 | 60 | 27 | 15 | 29 | 23 | 17 | 16 |
| 12769290 | SLC16A3 | Bos taurus solute carrier family 16, member 3 (monocarboxylic acid transporter 4) | molecular transport | 5.604 | 4.48 | 3.661 | 169 | 154 | 201 | 164 | 39 | 49 | 53 | 28 | 37 | 28 | 34 | 50 | 33 |
| 12909023 | ATP11C | PREDICTED: Bos taurus ATPase, class VI, type 11C | molecular transport | 6.303 | 6.212 | 2.216 | 894 | 1072 | 879 | 1251 | 487 | 500 | 392 | 179 | 137 | 169 | 156 | 180 | 155 |
| 12860556 | SLC38A1 | Solute Carrier Family 38, Member 1 | molecular transport | 6.671 | 10.992 | 2.883 | 1771 | 1825 | 1345 | 1692 | 641 | 507 | 572 | 264 | 212 | 269 | 167 | 181 | 111 |
| 12843700 | SLC44A3 | Bos taurus solute carrier family 44, member 3 | molecular transport | 7.758 | 4.188 | 2.725 | 480 | 491 | 458 | 529 | 209 | 163 | 169 | 72 | 58 | 60 | 124 | 137 | 93 |
| 12724305 | TTPA | Bos taurus tocopherol (alpha) transfer protein | molecular transport | 13.677 | 14.477 | 8.224 | 251 | 254 | 212 | 265 | 55 | 28 | 17 | 14 | 23 | 18 | 19 | 17 | 15 |
| 12844142 | SLC27A3 | PREDICTED: Bos taurus solute carrier family 27 (fatty acid transporter), member 3 | molecular transport | 16.892 | 14.344 | 2.58 | 347 | 406 | 358 | 534 | 162 | 144 | 166 | 23 | 24 | 25 | 21 | 35 | 30 |
| 12896203 | POPDC3 | Bos taurus popeye domain containing 3 | muscle development | 18.706 | 20.436 | 6.422 | 258 | 326 | 206 | 418 | 63 | 32 | 46 | 20 | 15 | 13 | 17 | 16 | 11 |
| 12894401 | NXNL2 | Bos taurus nucleoredoxin-like 2 | neuron function | 6.207 | 5.993 | 2.472 | 341 | 346 | 228 | 383 | 128 | 135 | 123 | 38 | 54 | 65 | 46 | 58 | 56 |
| 12842993 | LMNA | Bos taurus lamin A/C | nuclear structure | -4.198 | -3.348 | -2.062 | 112 | 127 | 143 | 131 | 275 | 288 | 232 | 460 | 555 | 606 | 438 | 443 | 404 |
| 12858852 | TMPO | Bos taurus thymopoietin | nuclear structure | 3.294 | 2.909 | 2.187 | 838 | 890 | 707 | 861 | 435 | 352 | 345 | 286 | 262 | 206 | 273 | 318 | 258 |
| 12887402 | LMNB1 | Bos taurus lamin B1 | nuclear structure | 7.812 | 6.935 | 2.055 | 164 | 174 | 126 | 146 | 73 | 75 | 72 | 17 | 21 | 20 | 21 | 24 | 21 |
| 12788100 | DUSP1 | Bos taurus dual specificity phosphatase 1 | phosphatase | -21.008 | -24.032 | -3.086 | 205 | 190 | 182 | 165 | 516 | 480 | 751 | 4021 | 3893 | 3744 | 4053 | 5164 | 4190 |
| 12899272 | PTPRK | Bos taurus protein tyrosine phosphatase, receptor type, K | phosphatase | 2.099 | 6.611 | 2.11 | 236 | 249 | 261 | 236 | 97 | 137 | 118 | 153 | 96 | 109 | 36 | 42 | 34 |
| 12869707 | PTPN13 | Protein Tyrosine Phosphatase, Non-Receptor Type 13 (APO-1/CD95 (Fas)-Associated Phosphatase) | phosphatase | 3.822 | 3.827 | 2.04 | 926 | 993 | 1236 | 1148 | 473 | 537 | 566 | 288 | 285 | 266 | 261 | 238 | 350 |
| 12891005 | PTPRD | Protein Tyrosine Phosphatase, Receptor Type, D | phosphatase | 5.067 | 9.468 | 2.601 | 509 | 521 | 468 | 762 | 196 | 213 | 232 | 110 | 92 | 129 | 43 | 41 | 115 |
| 12694290 | DLGAP5 | Bos taurus discs, large (Drosophila) homolog-associated protein 5 | phosphatase | 16.08 | 16.791 | 3.421 | 424 | 423 | 425 | 385 | 141 | 107 | 118 | 26 | 38 | 17 | 34 | 21 | 21 |
| 12700364 | PLEK | Bos taurus pleckstrin | platelet function | 6.309 | 4.641 | 3.286 | 112 | 163 | 101 | 166 | 54 | 32 | 38 | 24 | 16 | 24 | 26 | 40 | 23 |
| 12872049 | CPEB2 | Cytoplasmic Polyadenylation Element Binding Protein 2 | posttranscriptional modification | -4.217 | -5.47 | -2.503 | 168 | 187 | 251 | 250 | 440 | 528 | 631 | 748 | 1033 | 907 | 1271 | 1006 | 1195 |
| 12815814 | CARHSP1 | Bos taurus calcium regulated heat stable protein 1, 24kDa | posttranscriptional modification | -2.922 | -3.184 | -2.066 | 493 | 423 | 345 | 205 | 989 | 614 | 615 | 944 | 1012 | 1106 | 1115 | 898 | 1365 |
| 12704506 | C1D | Bos taurus C1D nuclear receptor corepressor | posttranscriptional modification | 2.182 | 2.315 | 2.07 | 781 | 1054 | 973 | 907 | 444 | 385 | 519 | 423 | 407 | 440 | 398 | 343 | 464 |
| 12754435 | FTSJD1 | PREDICTED: Bos taurus FtsJ methyltransferase domain containing 1 AKA Cap Methyltransferase 2. | posttranscriptional modification | 2.445 | 3.221 | 2.101 | 225 | 210 | 152 | 296 | 94 | 89 | 126 | 107 | 64 | 99 | 71 | 66 | 63 |
| 12761717 | MSI2 | Bos taurus musashi homolog 2 (Drosophila) | posttranscriptional modification | 2.829 | 2.578 | 2.083 | 1420 | 1615 | 1488 | 1640 | 785 | 701 | 732 | 480 | 559 | 598 | 639 | 446 | 746 |
| 12790569 | MPHOSPH10 | Bos taurus M-phase phosphoprotein 10 (U3 small nucleolar ribonucleoprotein) | posttranscriptional modification | 3.295 | 3.061 | 2.333 | 235 | 240 | 166 | 269 | 89 | 96 | 103 | 71 | 76 | 58 | 70 | 78 | 72 |
| 12836672 | MAGOH | Bos taurus mago-nashi homolog, proliferation-associated (Drosophila) | posttranscriptional modification | 3.355 | 3.526 | 2.04 | 244 | 231 | 387 | 272 | 137 | 89 | 206 | 66 | 90 | 96 | 88 | 67 | 82 |
| 12862077 | MAGOHB | Mago-Nashi Homolog B (Drosophila) | posttranscriptional modification | 4.074 | 3.178 | 2.174 | 163 | 193 | 125 | 246 | 106 | 65 | 77 | 56 | 49 | 30 | 58 | 54 | 54 |
| 12810060 | MEX3C | Mex-3 RNA Binding Family Member C | posttranscriptional modification | 4.793 | 4.266 | 2.794 | 1330 | 1428 | 1279 | 1504 | 471 | 420 | 613 | 336 | 301 | 237 | 330 | 343 | 301 |
| 12871619 | NSUN7 | Bos taurus NOL1/NOP2/Sun domain family, member 7, mRNA (cDNA clone IMAGE:8038878), partial cds. | posttranscriptional modification | 5.003 | 5.933 | 5.964 | 103 | 132 | 119 | 139 | 21 | 18 | 23 | 20 | 29 | 25 | 21 | 28 | 15 |
| 12782631 | BARD1 | Bos taurus BRCA1 associated RING domain 1 | posttranscriptional modification | 5.209 | 4.878 | 2.27 | 347 | 338 | 320 | 324 | 137 | 146 | 156 | 72 | 67 | 54 | 65 | 72 | 68 |
| 12833395 | CTSF | Bos taurus cathepsin F | posttranslational modification | -14.186 | -21.057 | -4.103 | 197 | 162 | 179 | 135 | 800 | 707 | 563 | 2291 | 2597 | 2215 | 3237 | 3669 | 3629 |
| 12836201 | CTSS | Bos taurus cathepsin S | posttranslational modification | -7.087 | -5.391 | -6.383 | 114 | 119 | 541 | 101 | 930 | 966 | 1295 | 1172 | 1215 | 1117 | 744 | 959 | 982 |
| 12857956 | ERP27 | Bos taurus endoplasmic reticulum protein 27 | posttranslational modification | -4.027 | -10.313 | -5.627 | 248 | 288 | 134 | 109 | 947 | 847 | 1290 | 690 | 1049 | 524 | 1470 | 2256 | 1922 |
| 12742671 | ISCU | Bos taurus iron-sulfur cluster scaffold homolog (E. coli), nuclear gene encoding mitochondrial protein | posttranslational modification | -3.919 | -4.049 | -2.155 | 781 | 812 | 886 | 683 | 1570 | 1629 | 1908 | 3412 | 2911 | 2954 | 3537 | 3281 | 2790 |
| 12847485 | GSTM2 | Bos taurus glutathione S-transferase M2, mRNA (cDNA clone IMAGE:7963385), partial cds. | posttranslational modification | -3.888 | -6.008 | -4.941 | 296 | 334 | 392 | 290 | 1414 | 1584 | 1859 | 1190 | 1499 | 1138 | 2005 | 2043 | 1828 |
| 12848033 | GSTM1 | Bos taurus glutathione S-transferase mu 1 | posttranslational modification | -3.468 | -5.166 | -2.72 | 170 | 196 | 231 | 158 | 556 | 421 | 558 | 582 | 798 | 582 | 928 | 995 | 968 |
| 12698798 | ST3GAL5 | ST3 Beta-Galactoside Alpha-2,3-Sialyltransferase 5 | posttranslational modification | -2.419 | -4.079 | -2.754 | 114 | 138 | 118 | 103 | 264 | 338 | 382 | 315 | 324 | 227 | 470 | 567 | 417 |
| 12818997 | HTRA1 | Bos taurus HtrA serine peptidase 1 | posttranslational modification | -2.364 | -3.527 | -2.355 | 862 | 897 | 1112 | 1038 | 2301 | 2507 | 2079 | 2398 | 2457 | 2058 | 3565 | 3337 | 3386 |
| 12889986 | BMP1 | Bone Morphogenetic Protein 1 | posttranslational modification | -2.009 | -2.774 | -2.271 | 390 | 397 | 420 | 351 | 1184 | 725 | 803 | 771 | 908 | 681 | 979 | 1098 | 1167 |
| 12809169 | MOCOS | Bos taurus molybdenum cofactor sulfurase | posttranslational modification | -2 | -2.354 | -2.36 | 103 | 129 | 126 | 175 | 288 | 367 | 279 | 238 | 314 | 240 | 289 | 320 | 317 |
| 12898548 | UBE2J1 | Bos taurus ubiquitin-conjugating enzyme E2, J1, U | posttranslational modification | 2.327 | 2.938 | 2.714 | 600 | 692 | 992 | 727 | 273 | 230 | 322 | 341 | 278 | 339 | 261 | 236 | 259 |
| 12723360 | RNF19A | Bos taurus ring finger protein 19A | posttranslational modification | 2.744 | 2.827 | 2.189 | 1434 | 1527 | 1140 | 1742 | 627 | 760 | 602 | 516 | 533 | 530 | 465 | 642 | 446 |
| 12743577 | KLHL2 | Bos taurus kelch-like 2, Mayven (Drosophila) | posttranslational modification | 2.898 | 2.733 | 2.343 | 297 | 331 | 366 | 374 | 148 | 152 | 137 | 123 | 134 | 98 | 104 | 115 | 161 |
| 12753982 | UBE2S | Bos taurus ubiquitin-conjugating enzyme E2S | posttranslational modification | 3.019 | 3.332 | 2.73 | 611 | 659 | 543 | 458 | 222 | 166 | 237 | 186 | 189 | 184 | 169 | 164 | 173 |
| 12678907 | ST3GAL6 | Bos taurus ST3 beta-galactoside alpha-2,3-sialyltransferase 6 | posttranslational modification | 4.318 | 13.708 | 2.479 | 173 | 190 | 204 | 223 | 106 | 71 | 66 | 33 | 36 | 79 | 13 | 13 | 18 |
| 12737098 | B3GALT2 | Bos taurus UDP-Gal:betaGlcNAc beta 1,3-galactosyltransferase, polypeptide 2 | posttranslational modification | 4.781 | 6.791 | 2.317 | 330 | 332 | 381 | 276 | 159 | 123 | 145 | 76 | 72 | 59 | 53 | 44 | 48 |
| 12697186 | PIGB | Bos taurus phosphatidylinositol glycan anchor biosynthesis, class B | posttranslational modification | 5.081 | 4.494 | 2.153 | 395 | 437 | 380 | 463 | 191 | 161 | 238 | 93 | 73 | 82 | 109 | 56 | 132 |
| 12711895 | UGGT2 | PREDICTED: Bos taurus UDP-glucose glycoprotein glucosyltransferase 2 | posttranslational modification | 5.866 | 4.956 | 2.464 | 339 | 343 | 373 | 497 | 155 | 151 | 160 | 69 | 66 | 61 | 86 | 77 | 70 |
| 12849070 | ASB15 | Ankyrin Repeat And SOCS Box Containing 15 | posttranslational modification | 8.883 | 9.013 | 4.572 | 126 | 150 | 159 | 113 | 46 | 26 | 22 | 17 | 13 | 16 | 18 | 12 | 16 |
| 12714343 | UBE2C | Ubiquitin-Conjugating Enzyme E2C | posttranslational modification | 10.025 | 14.431 | 2.121 | 1163 | 1028 | 1290 | 790 | 489 | 474 | 523 | 112 | 98 | 105 | 91 | 61 | 70 |
| 12740723 | DTL | Denticleless E3 Ubiquitin Protein Ligase Homolog (Drosophila) | posttranslational modification | 10.49 | 11.552 | 2.665 | 685 | 650 | 563 | 463 | 235 | 179 | 250 | 59 | 62 | 47 | 63 | 45 | 46 |
| 12860640 | CHST11 | Bos taurus carbohydrate (chondroitin 4) sulfotransferase 11 | posttranslational modification | 22.418 | 35.867 | 2.91 | 3065 | 3157 | 3078 | 4248 | 1508 | 1035 | 981 | 153 | 163 | 134 | 76 | 100 | 107 |
| 12900227 | FBXO5 | Bos taurus F-box protein 5 | posttranslational modification | 25.009 | 23.534 | 4.15 | 588 | 545 | 488 | 457 | 123 | 116 | 136 | 23 | 28 | 14 | 26 | 18 | 22 |
| 12748838 | CHST8 | Bos taurus carbohydrate (N-acetylgalactosamine 4-0) sulfotransferase 8 | posttranslational modification | 39.021 | 40.747 | 3.289 | 2172 | 2325 | 1812 | 1880 | 873 | 555 | 490 | 63 | 44 | 50 | 73 | 34 | 50 |
| 12686124 | KIAA1524 | Bos taurus KIAA1524 ortholog | proliferation | 9.308 | 6.367 | 2.237 | 375 | 439 | 396 | 413 | 223 | 153 | 174 | 45 | 54 | 34 | 60 | 72 | 59 |
| 12880336 | RHOBTB3 | PREDICTED: Bos taurus Rho-related BTB domain containing 3 | protein trafficking | -3.677 | -3.112 | -2.558 | 188 | 224 | 240 | 315 | 555 | 713 | 568 | 858 | 838 | 928 | 743 | 726 | 751 |
| 12848151 | HOOK1 | Bos taurus hook homolog 1 (Drosophila) | protein trafficking | 2.426 | 3.553 | 4.859 | 106 | 146 | 77 | 136 | 28 | 21 | 21 | 42 | 37 | 66 | 29 | 37 | 30 |
| 12887682 | SNX24 | Bos taurus sorting nexin 24 | protein trafficking | 2.506 | 2.111 | 2.095 | 578 | 607 | 453 | 701 | 298 | 265 | 266 | 260 | 230 | 205 | 332 | 250 | 247 |
| 12824307 | SNX25 | Bos taurus sorting nexin 25 | protein trafficking | 3.708 | 3.841 | 2.113 | 217 | 224 | 230 | 220 | 110 | 84 | 127 | 60 | 59 | 61 | 62 | 53 | 59 |
| 12788447 | TNPO1 | Bos taurus transportin 1 | protein trafficking | 4.018 | 4.1 | 2.111 | 2659 | 2847 | 2567 | 3504 | 1358 | 1328 | 1396 | 756 | 712 | 679 | 754 | 678 | 673 |
| 12731023 | CEP57 | Centrosomal Protein 57kDa | protein trafficking | 4.362 | 3.634 | 2.24 | 404 | 420 | 186 | 336 | 119 | 173 | 143 | 76 | 75 | 70 | 81 | 112 | 76 |
| 12742595 | MGARP | mitochondria-localized glutamic acid-rich protein (MGARP) | protein trafficking | 4.39 | 25.474 | 5.06 | 802 | 859 | 707 | 918 | 255 | 159 | 104 | 224 | 124 | 233 | 30 | 35 | 31 |
| 12871651 | WDR19 | Bos taurus WD repeat domain 19 | protein trafficking | 5.925 | 3.58 | 2.239 | 1589 | 1848 | 1438 | 1503 | 711 | 777 | 645 | 285 | 274 | 247 | 386 | 480 | 470 |
| 12866791 | LIN7A | Bos taurus lin-7 homolog A (C. elegans) | protein trafficking | 6.187 | 4.878 | 3.26 | 166 | 172 | 189 | 167 | 55 | 49 | 56 | 25 | 35 | 25 | 38 | 39 | 30 |
| 12889007 | CLU | Bos taurus clusterin | protein-protein binding | -13.592 | -8.739 | -4.828 | 213 | 178 | 162 | 99 | 655 | 784 | 851 | 2126 | 2035 | 2257 | 1283 | 1682 | 1202 |
| 12836065 | S100A10 | Bos taurus S100 calcium binding protein A10 | protein-protein binding | -7.481 | -4.484 | -6.663 | 138 | 170 | 185 | 221 | 1335 | 1289 | 933 | 1204 | 1265 | 1491 | 1133 | 654 | 661 |
| 12685757 | FSTL1 | Bos taurus follistatin-like 1 | protein-protein binding | -2.042 | -2.185 | -2.2 | 640 | 686 | 870 | 904 | 1538 | 1810 | 1724 | 1732 | 1664 | 1332 | 1855 | 1782 | 1424 |
| 12791451 | BTBD6 | Bos taurus BTB (POZ) domain containing 6 | protein-protein binding | 2.216 | 2.179 | 2.356 | 382 | 290 | 375 | 180 | 154 | 142 | 89 | 149 | 132 | 118 | 155 | 142 | 111 |
| 12790384 | BTBD1 | Bos taurus BTB (POZ) domain containing 1 | protein-protein binding | 2.692 | 2.727 | 2.109 | 618 | 630 | 609 | 704 | 363 | 292 | 262 | 216 | 231 | 268 | 217 | 238 | 249 |
| 12715127 | PFDN4 | Bos taurus prefoldin subunit 4 | protein-protein binding | 2.718 | 3.3 | 2.249 | 101 | 104 | 111 | 166 | 79 | 39 | 47 | 56 | 36 | 41 | 44 | 32 | 33 |
| 12839021 | EFHD1 | Bos taurus EF-hand domain family, member D1 | protein-protein binding | 2.805 | 8.501 | 3.835 | 532 | 507 | 584 | 490 | 153 | 116 | 147 | 206 | 178 | 181 | 93 | 55 | 46 |
| 12903130 | CASK | Bos taurus calcium/calmodulin-dependent serine protein kinase (MAGUK family) | protein-protein binding | 2.88 | 2.222 | 2.167 | 1284 | 1404 | 1488 | 1610 | 721 | 627 | 651 | 514 | 517 | 472 | 602 | 596 | 760 |
| 12736928 | FBXO28 | Bos taurus F-box protein 28 | protein-protein binding | 3.329 | 2.999 | 2.604 | 710 | 815 | 774 | 841 | 298 | 275 | 332 | 241 | 216 | 250 | 265 | 230 | 293 |
| 12745816 | HSPA4L | PREDICTED: Bos taurus heat shock 70kDa protein 4-like, transcript variant 3 | protein-protein binding | 4.702 | 6.209 | 5.508 | 219 | 234 | 197 | 208 | 45 | 31 | 41 | 51 | 42 | 44 | 27 | 30 | 51 |
| 12740526 | NPHS2 | Bos taurus nephrosis 2, idiopathic, steroid-resistant (podocin) | protein-protein binding | 4.952 | 4.401 | 3.322 | 138 | 162 | 126 | 91 | 35 | 36 | 44 | 27 | 22 | 28 | 26 | 38 | 25 |
| 12742144 | CLGN | Bos taurus calmegin | protein-protein binding | 5.221 | 31.354 | 3.955 | 830 | 978 | 1066 | 1206 | 287 | 268 | 217 | 258 | 94 | 300 | 32 | 35 | 30 |
| 12891218 | DNAJC25 | Bos taurus DnaJ (Hsp40) homolog, subfamily C , member 25 | protein-protein binding | 6.857 | 7.95 | 3.499 | 913 | 953 | 837 | 1141 | 318 | 216 | 296 | 151 | 135 | 133 | 126 | 110 | 124 |
| 12837565 | STIL | Bos taurus SCL/TAL1 interrupting locus | protein-protein binding | 6.877 | 10.517 | 3.268 | 301 | 311 | 271 | 256 | 83 | 81 | 97 | 44 | 51 | 31 | 25 | 33 | 24 |
| 12706464 | CTNNA2 | Bos taurus catenin (cadherin-associated protein), alpha 2 | protein-protein binding | 7.084 | 7.99 | 2.845 | 104 | 159 | 98 | 183 | 64 | 40 | 38 | 26 | 12 | 21 | 15 | 16 | 19 |
| 12767708 | BRCA1 | Bos taurus breast cancer 1, early onset | protein-protein binding | 7.625 | 5.637 | 3.004 | 251 | 268 | 205 | 233 | 86 | 65 | 88 | 35 | 34 | 25 | 46 | 36 | 45 |
| 12796560 | ERC2 | ELKS/RAB6-Interacting/CAST Family Member 2 | protein-protein binding | 8.443 | 13.886 | 4.04 | 188 | 235 | 231 | 233 | 75 | 59 | 37 | 28 | 21 | 30 | 13 | 20 | 15 |
| 12722747 | ATAD2 | Bos taurus ATPase family, AAA domain containing 2 | protein-protein binding | 8.582 | 7.872 | 2.722 | 1246 | 1470 | 1298 | 1266 | 461 | 449 | 546 | 139 | 169 | 155 | 149 | 158 | 198 |
| 12906742 | ODZ1 | Bos taurus odz, odd Oz/ten-m homolog 1 (Drosophila) Teneurin Transmembrane Protein 1. | protein-protein binding | 8.914 | 7.983 | 2.768 | 179 | 195 | 258 | 266 | 86 | 86 | 69 | 24 | 19 | 32 | 27 | 26 | 30 |
| 12790079 | STRA6 | Stimulated By Retinoic Acid 6 | retinol metabolism | 57.279 | 53.296 | 4.096 | 1771 | 1713 | 1211 | 1613 | 562 | 339 | 290 | 25 | 39 | 21 | 31 | 32 | 25 |
| 12780541 | IGFBP5 | Bos taurus insulin-like growth factor binding protein 5 | signaling | -12.793 | -25.31 | -2.189 | 132 | 102 | 167 | 77 | 236 | 321 | 210 | 1548 | 1620 | 1267 | 2916 | 3408 | 2475 |
| 12723272 | GEM | Bos taurus GTP binding protein overexpressed in skeletal muscle | signaling | -11.302 | -12.755 | -2.213 | 132 | 106 | 130 | 123 | 310 | 247 | 260 | 1331 | 1497 | 1325 | 1576 | 1623 | 1484 |
| 12843892 | CYR61 | Bos taurus cysteine-rich, angiogenic inducer, 61 | signaling | -10.752 | -16.672 | -5.926 | 181 | 150 | 230 | 182 | 1090 | 1116 | 1059 | 1981 | 2125 | 1829 | 2690 | 3345 | 3188 |
| 12678498 | APP | Bos taurus amyloid beta (A4) precursor protein | signaling | -7.509 | -4.66 | -4.442 | 266 | 323 | 435 | 321 | 1283 | 1737 | 1428 | 2629 | 2566 | 2280 | 1635 | 1608 | 1398 |
| 12857319 | PTHLH | Parathyroid Hormone-Like Hormone | signaling | -6.703 | 3.527 | 2.289 | 283 | 216 | 512 | 187 | 107 | 92 | 180 | 2132 | 1259 | 2382 | 92 | 49 | 107 |
| 12686004 | ROBO1 | Bos taurus roundabout, axon guidance receptor, homolog 1 (Drosophila) | signaling | -6.112 | -11.465 | -4.259 | 159 | 174 | 346 | 136 | 674 | 971 | 811 | 1222 | 1175 | 1091 | 1829 | 2589 | 2186 |
| 12839347 | WLS | Bos taurus wntless homolog (Drosophila) | signaling | -5.908 | -5.78 | -6.559 | 141 | 125 | 142 | 104 | 795 | 878 | 827 | 748 | 738 | 765 | 680 | 668 | 869 |
| 12886534 | TRIP10 | Thyroid Hormone Receptor Interactor 10 | signaling | -5.22 | -5.352 | -3.129 | 121 | 128 | 115 | 135 | 375 | 431 | 368 | 593 | 704 | 660 | 533 | 783 | 713 |
| 12699180 | CRIM1 | Bos taurus cysteine rich transmembrane BMP regulator 1 (chordin-like) | signaling | -4.887 | -2.837 | -2.189 | 133 | 131 | 184 | 141 | 288 | 417 | 271 | 753 | 677 | 712 | 402 | 453 | 390 |
| 12804076 | JSP.1 | Bos taurus MHC Class I JSP.1 | signaling | -4.75 | -4.768 | -3.544 | 164 | 549 | 300 | 269 | 1093 | 1482 | 685 | 1451 | 1295 | 1419 | 1287 | 1577 | 1330 |
| 12789530 | IFI27 | Interferon, Alpha-Inducible Protein 27 | signaling | -4.579 | -6.137 | -6.959 | 128 | 107 | 82 | 236 | 671 | 375 | 2772 | 405 | 675 | 726 | 642 | 653 | 1140 |
| 12883457 | CD97 | Bos taurus CD97 molecule. Adhesion G Protein-Coupled Receptor E5 | signaling | -4.437 | -2.759 | -2.268 | 103 | 116 | 112 | 88 | 258 | 247 | 208 | 438 | 441 | 511 | 288 | 297 | 277 |
| 12857267 | TNFRSF1A | Bos taurus tumor necrosis factor receptor superfamily, member 1A | signaling | -3.948 | -4.604 | -2.521 | 250 | 304 | 272 | 196 | 792 | 600 | 541 | 1085 | 1055 | 861 | 1442 | 1131 | 958 |
| 12735710 | ERRFI1 | Bos taurus ERBB receptor feedback inhibitor 1 | signaling | -3.794 | -5.181 | -2.217 | 391 | 396 | 380 | 284 | 1021 | 669 | 742 | 1472 | 1209 | 1428 | 1695 | 2144 | 1779 |
| 12713867 | PLCB1 | Phospholipase C, Beta 1 (Phosphoinositide-Specific) | signaling | -3.621 | -2.764 | -2.138 | 154 | 168 | 147 | 166 | 278 | 376 | 372 | 562 | 591 | 568 | 355 | 506 | 469 |
| 12680487 | ARHGAP31 | Bos taurus Rho GTPase activating protein 31 | signaling | -3.535 | -3.042 | -2.46 | 342 | 384 | 282 | 308 | 776 | 1085 | 619 | 1258 | 1041 | 1180 | 1133 | 1010 | 861 |
| 12798073 | ITPR1 | Bos taurus inositol 1,4,5-trisphosphate receptor, type 1 | signaling | -3.461 | -4.042 | -2.804 | 147 | 138 | 206 | 139 | 386 | 454 | 471 | 464 | 615 | 545 | 435 | 479 | 1190 |
| 12895145 | SMARCA2 | Bos taurus SWI/SNF related, matrix associated, actin dependent regulator of chromatin, subfamily a, member 2 | signaling | -2.867 | -2.995 | -2.153 | 183 | 206 | 239 | 168 | 388 | 538 | 368 | 545 | 552 | 603 | 604 | 602 | 568 |
| 12744361 | PDGFC | PREDICTED: Bos taurus platelet derived growth factor C | signaling | -2.765 | -3.155 | -2.327 | 124 | 132 | 138 | 128 | 302 | 384 | 243 | 374 | 382 | 330 | 399 | 497 | 353 |
| 12828291 | NCOA4 | Bos taurus nuclear receptor coactivator 4 | signaling | -2.719 | -2.939 | -2.068 | 273 | 263 | 272 | 374 | 645 | 617 | 556 | 755 | 772 | 862 | 832 | 847 | 900 |
| 12841743 | TGFBR3 | PREDICTED: Bos taurus transforming growth factor, beta receptor III | signaling | -2.312 | -4.927 | -3.558 | 258 | 275 | 260 | 432 | 969 | 1216 | 1022 | 912 | 706 | 513 | 1416 | 1626 | 1387 |
| 12907364 | IL13RA1 | Interleukin 13 Receptor, Alpha 1 | signaling | -2.242 | -2.339 | -2.224 | 207 | 235 | 391 | 373 | 505 | 870 | 611 | 722 | 624 | 610 | 673 | 774 | 599 |
| 12731936 | ARHGAP1 | Bos taurus Rho GTPase activating protein 1 | signaling | -2.061 | -2.677 | -2.017 | 439 | 399 | 389 | 257 | 918 | 855 | 503 | 857 | 821 | 599 | 1036 | 1074 | 830 |
| 12897583 | PLEKHG1 | Bos taurus pleckstrin homology domain containing, family G (with RhoGef domain) member 1 | signaling | 2.065 | 5.821 | 2.838 | 554 | 503 | 580 | 440 | 185 | 184 | 177 | 286 | 256 | 214 | 82 | 100 | 85 |
| 12751697 | NDRG4 | Bos taurus NDRG family member 4 | signaling | 2.129 | 5.213 | 2.29 | 279 | 291 | 364 | 329 | 135 | 117 | 163 | 162 | 114 | 174 | 48 | 58 | 78 |
| 12680597 | PIK3CB | Bos taurus phosphoinositide-3-kinase, catalytic, beta polypeptide | signaling | 2.163 | 3.486 | 3.335 | 202 | 233 | 275 | 332 | 71 | 76 | 84 | 112 | 112 | 132 | 78 | 79 | 64 |
| 12760345 | CYTH1 | Cytohesin 1 | signaling | 2.195 | 2.585 | 2.324 | 482 | 535 | 365 | 583 | 245 | 185 | 200 | 217 | 211 | 234 | 159 | 226 | 182 |
| 12781968 | EPHA4 | EPH Receptor A4 | signaling | 2.207 | 4.601 | 2.47 | 367 | 315 | 485 | 252 | 129 | 124 | 170 | 151 | 161 | 157 | 73 | 81 | 71 |
| 12684420 | HEG1 | PREDICTED: Bos taurus HEG homolog 1 (zebrafish) | signaling | 2.334 | 5.217 | 3.501 | 1679 | 1932 | 1186 | 2147 | 517 | 502 | 437 | 760 | 786 | 641 | 310 | 388 | 285 |
| 12729339 | PIK3C2A | PREDICTED: Bos taurus phosphoinositide-3-kinase, class 2, alpha polypeptide | signaling | 2.417 | 2.756 | 2.126 | 765 | 753 | 727 | 890 | 350 | 347 | 408 | 325 | 310 | 335 | 233 | 310 | 315 |
| 12790617 | CSPG4 | Bos taurus chondroitin sulfate proteoglycan 4 | signaling | 2.511 | 5.133 | 2.031 | 170 | 168 | 150 | 189 | 102 | 71 | 79 | 64 | 61 | 78 | 41 | 26 | 33 |
| 12743174 | KREMEN1 | Kringle Containing Transmembrane Protein 1 | signaling | 2.582 | 2.543 | 2.05 | 590 | 569 | 583 | 507 | 294 | 236 | 296 | 258 | 193 | 206 | 303 | 153 | 230 |
| 12909865 | CHRDL1 | Chordin-Like 1 | signaling | 2.595 | 6.648 | 2.201 | 295 | 304 | 381 | 393 | 148 | 142 | 177 | 145 | 94 | 165 | 37 | 37 | 100 |
| 12728473 | MAPK8IP1 | Bos taurus mitogen-activated protein kinase 8 interacting protein 1 | signaling | 2.701 | 2.674 | 2.016 | 152 | 214 | 146 | 181 | 96 | 85 | 75 | 67 | 61 | 62 | 56 | 71 | 66 |
| 12709777 | RAP2A | Bos taurus RAP2A, member of RAS oncogene family | signaling | 2.78 | 2.038 | 2.029 | 731 | 725 | 773 | 958 | 469 | 357 | 354 | 309 | 297 | 252 | 484 | 390 | 310 |
| 12879220 | SEMA6A | Bos taurus sema domain, transmembrane domain (TM), and cytoplasmic domain, (semaphorin) 6A | signaling | 2.871 | 5.72 | 2.83 | 1825 | 1773 | 1362 | 1293 | 483 | 547 | 616 | 517 | 629 | 479 | 263 | 297 | 251 |
| 12724180 | ARFGEF1 | Bos taurus ADP-ribosylation factor guanine nucleotide-exchange factor 1 (brefeldin A-inhibited) | signaling | 3.114 | 3.08 | 2.104 | 955 | 1038 | 671 | 1270 | 485 | 467 | 417 | 306 | 306 | 311 | 283 | 348 | 306 |
| 12848320 | BCL9 | PREDICTED: Bos taurus B-cell CLL/lymphoma 9 | signaling | 3.2 | 2.765 | 2.467 | 1204 | 1268 | 791 | 1190 | 611 | 423 | 338 | 354 | 346 | 327 | 374 | 439 | 379 |
| 12867814 | SRGAP1 | Bos taurus SLIT-ROBO Rho GTPase activating protein 1 | signaling | 3.208 | 2.768 | 2.388 | 457 | 563 | 497 | 511 | 226 | 211 | 199 | 167 | 160 | 147 | 175 | 159 | 219 |
| 12870215 | FAM13A | Bos taurus family with sequence similarity 13, member A | signaling | 3.222 | 4.015 | 2.56 | 801 | 795 | 410 | 635 | 309 | 246 | 204 | 196 | 189 | 209 | 140 | 151 | 190 |
| 12786931 | NPR3 | Bos taurus natriuretic peptide receptor C/guanylate cyclase C (atrionatriuretic peptide receptor C) | signaling | 3.512 | 25.142 | 3.116 | 1577 | 1334 | 909 | 2315 | 441 | 492 | 465 | 405 | 360 | 483 | 70 | 34 | 80 |
| 12690056 | F2R | Bos taurus coagulation factor II (thrombin) receptor | signaling | 3.53 | 2.629 | 2.195 | 448 | 407 | 315 | 313 | 181 | 166 | 155 | 110 | 102 | 100 | 150 | 141 | 128 |
| 12711992 | CAB39L | Calcium Binding Protein 39-Like | signaling | 3.727 | 3.784 | 2.979 | 977 | 1054 | 1000 | 901 | 370 | 318 | 304 | 342 | 215 | 248 | 318 | 230 | 238 |
| 12789065 | IGF1R | Bos taurus insulin-like growth factor 1 receptor | signaling | 3.761 | 3.014 | 2.336 | 1678 | 1666 | 1688 | 1524 | 912 | 671 | 562 | 393 | 497 | 422 | 494 | 564 | 575 |
| 12697094 | RAB15 | Bos taurus RAB15, member RAS onocogene family | signaling | 3.861 | 4.162 | 2.281 | 394 | 358 | 336 | 487 | 221 | 162 | 139 | 118 | 84 | 104 | 99 | 103 | 80 |
| 12706420 | TBC1D8 | Bos taurus TBC1 domain family, member 8 (with GRAM domain) | signaling | 3.94 | 5.941 | 2.407 | 548 | 546 | 463 | 526 | 296 | 201 | 169 | 136 | 130 | 130 | 83 | 99 | 81 |
| 12844648 | GPSM2 | Bos taurus G-protein signaling modulator 2 | signaling | 3.943 | 2.621 | 2.022 | 258 | 236 | 243 | 287 | 129 | 105 | 147 | 63 | 78 | 55 | 94 | 104 | 94 |
| 12898719 | RRAGD | Bos taurus Ras-related GTP binding D | signaling | 4.058 | 4.739 | 3.878 | 443 | 470 | 873 | 593 | 182 | 129 | 138 | 130 | 115 | 188 | 130 | 141 | 96 |
| 12780755 | RCAN3 | Bos taurus RCAN family member 3 | signaling | 4.227 | 8.757 | 4.25 | 277 | 250 | 280 | 231 | 79 | 47 | 60 | 76 | 58 | 52 | 28 | 29 | 32 |
| 12689622 | RGS6 | Regulator Of G-Protein Signaling 6 | signaling | 4.538 | 7.351 | 3.015 | 961 | 1033 | 1011 | 1180 | 362 | 339 | 337 | 261 | 200 | 233 | 229 | 119 | 105 |
| 12836813 | IQGAP3 | Bos taurus IQ motif containing GTPase activating protein 3 | signaling | 4.58 | 5.53 | 2.087 | 163 | 146 | 141 | 99 | 69 | 49 | 79 | 36 | 31 | 22 | 25 | 21 | 28 |
| 12809770 | SMAD2 | Bos taurus SMAD family member 2 | signaling | 4.791 | 4.588 | 2.759 | 2198 | 2416 | 2048 | 2629 | 948 | 765 | 811 | 524 | 454 | 473 | 541 | 463 | 511 |
| 12898500 | EPHA7 | Bos taurus EPH receptor A7 | signaling | 4.972 | 17.009 | 4.424 | 386 | 390 | 300 | 266 | 81 | 63 | 81 | 88 | 48 | 70 | 20 | 20 | 18 |
| 12895648 | ARHGEF39 | Rho guanine nucleotide exchange factor (GEF) 39 | signaling | 5.007 | 4.946 | 2.276 | 174 | 159 | 143 | 135 | 72 | 60 | 70 | 34 | 29 | 29 | 32 | 35 | 26 |
| 12856932 | TAC3 | Bos taurus tachykinin 3 | signaling | 5.172 | 4.156 | 2.54 | 133 | 170 | 96 | 110 | 51 | 42 | 54 | 25 | 26 | 21 | 28 | 32 | 30 |
| 12690050 | F2RL1 | Bos taurus coagulation factor II (thrombin) receptor-like 1 | signaling | 5.279 | 2.872 | 3.754 | 233 | 229 | 128 | 205 | 35 | 33 | 118 | 33 | 41 | 36 | 48 | 56 | 114 |
| 12678379 | EPHA6 | EPH Receptor A6 | signaling | 5.822 | 10.776 | 4.261 | 244 | 191 | 302 | 214 | 58 | 49 | 58 | 57 | 24 | 47 | 19 | 21 | 27 |
| 12695131 | SEMA6D | Bos taurus sema domain, transmembrane domain (TM), and cytoplasmic domain, (semaphorin) 6D | signaling | 6.163 | 6.045 | 2.878 | 2225 | 2417 | 2403 | 2559 | 823 | 871 | 807 | 470 | 348 | 360 | 417 | 430 | 348 |
| 12889361 | DOCK5 | PREDICTED: Bos taurus dedicator of cytokinesis 5 | signaling | 7.017 | 5.824 | 2.71 | 598 | 705 | 557 | 541 | 273 | 199 | 196 | 98 | 70 | 90 | 100 | 125 | 86 |
| 12901045 | MAP3K5 | Bos taurus mitogen-activated protein kinase kinase kinase 5 | signaling | 7.032 | 5.591 | 3.172 | 788 | 806 | 603 | 747 | 261 | 199 | 236 | 95 | 120 | 98 | 82 | 164 | 166 |
| 12853629 | CHN2 | Bos taurus chimerin (chimaerin) 2 | signaling | 7.156 | 11.547 | 2.257 | 548 | 625 | 576 | 387 | 268 | 239 | 197 | 61 | 81 | 80 | 28 | 61 | 55 |
| 12843689 | IL6R | Bos taurus interleukin 6 receptor | signaling | 7.234 | 18.573 | 3.322 | 681 | 813 | 734 | 1098 | 308 | 222 | 218 | 113 | 106 | 120 | 34 | 49 | 51 |
| 12817058 | TMEM120A | Bos taurus transmembrane protein 120A | signaling | 7.884 | 8.653 | 2.558 | 2631 | 2917 | 2526 | 3200 | 1299 | 1073 | 947 | 396 | 334 | 341 | 385 | 243 | 365 |
| 12683782 | ECT2 | Bos taurus epithelial cell transforming sequence 2 oncogene | signaling | 8.04 | 5.145 | 3.318 | 609 | 635 | 600 | 581 | 211 | 156 | 185 | 78 | 78 | 70 | 113 | 120 | 120 |
| 12888738 | TMEFF1 | Bos taurus transmembrane protein with EGF-like and two follistatin-like domains 1 | signaling | 8.475 | 10.901 | 2.334 | 223 | 193 | 196 | 218 | 83 | 99 | 85 | 31 | 20 | 24 | 18 | 18 | 21 |
| 12859556 | RACGAP1 | PREDICTED: Bos taurus Rac GTPase activating protein 1, transcript variant 1 | signaling | 8.578 | 7.414 | 2.611 | 728 | 692 | 635 | 466 | 232 | 219 | 266 | 67 | 84 | 68 | 106 | 80 | 69 |
| 12876902 | HMMR | Bos taurus hyaluronan-mediated motility receptor (RHAMM) | signaling | 8.665 | 9.431 | 3.994 | 337 | 379 | 336 | 290 | 92 | 69 | 92 | 38 | 40 | 38 | 34 | 31 | 42 |
| 12873095 | EPHA5 | PREDICTED: Bos taurus ePH receptor A5-like (LOC100337226) | signaling | 9.525 | 16.73 | 2.877 | 873 | 851 | 818 | 1003 | 316 | 327 | 280 | 104 | 70 | 110 | 63 | 55 | 43 |
| 12786454 | DEPDC1B | Bos taurus DEP domain containing 1B | signaling | 9.765 | 9.492 | 3.288 | 124 | 120 | 143 | 106 | 37 | 36 | 40 | 10 | 12 | 16 | 11 | 11 | 18 |
| 12788381 | RGNEF | Bos taurus 190 kDa guanine nucleotide exchange factor | signaling | 10.487 | 21.527 | 2.895 | 1326 | 1376 | 1156 | 1278 | 547 | 398 | 398 | 143 | 127 | 101 | 68 | 62 | 50 |
| 12887876 | EFNA5 | Bos taurus ephrin-A5 | signaling | 11.931 | 25.066 | 5.413 | 1086 | 1034 | 1352 | 1424 | 255 | 261 | 169 | 90 | 98 | 119 | 43 | 67 | 39 |
| 12892173 | ROR2 | Bos taurus receptor tyrosine kinase-like orphan receptor 2 | signaling | 13.899 | 18.025 | 3.291 | 588 | 644 | 388 | 704 | 194 | 161 | 164 | 34 | 43 | 47 | 32 | 34 | 29 |
| 12777446 | INHBB | Bos taurus inhibin, beta B | signaling | 14.736 | 19.778 | 4.249 | 1445 | 1339 | 1084 | 1980 | 365 | 348 | 298 | 101 | 80 | 113 | 70 | 81 | 67 |
| 12897969 | BAI3 | Adhesion G Protein-Coupled Receptor B3 (ADGRB3, BAI3) | signaling | 15.768 | 14.096 | 6.555 | 259 | 225 | 420 | 271 | 35 | 30 | 78 | 25 | 16 | 15 | 15 | 25 | 22 |
| 12719872 | PTPRT | PREDICTED: Bos taurus protein tyrosine phosphatase, receptor type, T | signaling | 20.758 | 23.334 | 12.479 | 799 | 650 | 996 | 112 | 56 | 58 | 82 | 52 | 41 | 27 | 36 | 31 | 36 |
| 12849517 | GPR85 | Bos taurus G protein-coupled receptor 85 | signaling | 22.299 | 18.36 | 5.428 | 197 | 227 | 102 | 482 | 38 | 22 | 75 | 12 | 10 | 8 | 10 | 15 | 10 |
| 12697409 | ARHGAP11A | PREDICTED: Bos taurus Rho GTPase activating protein 11A | signaling | 27.441 | 34.546 | 4.257 | 451 | 457 | 449 | 475 | 112 | 98 | 114 | 25 | 16 | 11 | 9 | 18 | 15 |
| 12899705 | RGS17 | Bos taurus regulator of G-protein signaling 17 | signaling | 30.269 | 28.047 | 5.602 | 406 | 506 | 288 | 512 | 68 | 74 | 82 | 13 | 16 | 12 | 17 | 14 | 14 |
| 12693406 | ESR2 | Bos taurus estrogen receptor 2 (ER beta) | signaling | 33.166 | 27.874 | 3.899 | 713 | 757 | 380 | 625 | 214 | 130 | 130 | 21 | 15 | 18 | 20 | 27 | 19 |
| 12703820 | FSHR | Bos taurus follicle stimulating hormone receptor | signaling | 36.19 | 93.864 | 3.302 | 1327 | 1800 | 1175 | 2205 | 497 | 513 | 427 | 68 | 25 | 49 | 19 | 15 | 16 |
| 12890327 | LPPR1 | Bos taurus lipid phosphate phosphatase-related protein type 1 | signaling | 36.845 | 37.487 | 3.155 | 844 | 1108 | 798 | 978 | 173 | 342 | 425 | 23 | 27 | 26 | 29 | 26 | 20 |
| 12726690 | FDX1 | Bos taurus ferredoxin 1, nuclear gene encoding mitochondrial protein | steroidogenesis | -8.965 | -5.918 | -3.035 | 481 | 448 | 389 | 542 | 1609 | 1151 | 1488 | 4157 | 3794 | 4504 | 2881 | 2348 | 3019 |
| 12761316 | HSD17B1 | Bos taurus hydroxysteroid (17-beta) dehydrogenase 1 | steroidogenesis | 7.972 | 8.156 | 2.352 | 328 | 295 | 442 | 261 | 257 | 124 | 83 | 29 | 50 | 46 | 31 | 41 | 51 |
| 12900868 | CYB5R4 | Bos taurus cytochrome b5 reductase 4 | stress response | 2.756 | 2.826 | 2.571 | 236 | 277 | 230 | 237 | 108 | 88 | 90 | 87 | 86 | 93 | 81 | 90 | 89 |
| 12705036 | FOSL2 | Bos taurus FOS-like antigen 2 | transcription | -12.257 | -15.714 | -2.122 | 144 | 130 | 71 | 83 | 213 | 269 | 181 | 1130 | 1376 | 1288 | 1506 | 1674 | 1674 |
| 12697103 | ZFP36L1 | Bos taurus zinc finger protein 36, C3H type-like 1 | transcription | -11.483 | -14.318 | -5.121 | 155 | 158 | 136 | 97 | 662 | 718 | 681 | 1709 | 1534 | 1391 | 1964 | 2026 | 1777 |
| 12902223 | TSC22D3 | TSC22 Domain Family, Member 3 | transcription | -8.452 | -6.248 | -3.354 | 118 | 93 | 174 | 72 | 323 | 381 | 390 | 831 | 800 | 1154 | 725 | 589 | 726 |
| 12827244 | ARID5B | PREDICTED: Bos taurus AT rich interactive domain 5B (MRF1-like) | transcription | -7.578 | -10.653 | -2.63 | 128 | 130 | 98 | 141 | 357 | 377 | 253 | 902 | 1009 | 897 | 1126 | 1450 | 1389 |
| 12721602 | CEBPD | Bos taurus CCAAT/enhancer binding protein (C/EBP), delta | transcription | -4.628 | -7.045 | -2.063 | 262 | 254 | 441 | 344 | 685 | 555 | 737 | 1451 | 1449 | 1503 | 2050 | 2396 | 2270 |
| 12791694 | NR2F2 | Bos taurus nuclear receptor subfamily 2, group F, member 2 | transcription | -4.11 | -6.675 | -5.668 | 191 | 205 | 143 | 153 | 1000 | 1069 | 852 | 672 | 893 | 579 | 1081 | 1269 | 1085 |
| 12767359 | MYO1C | Bos taurus myosin IC | transcription | -3.512 | -2.497 | -2.525 | 166 | 168 | 167 | 140 | 447 | 460 | 319 | 609 | 573 | 505 | 444 | 383 | 372 |
| 12847452 | NFIA | Bos taurus nuclear factor I/A | transcription | -2.633 | -3.345 | -3.797 | 116 | 145 | 168 | 284 | 645 | 654 | 620 | 449 | 522 | 372 | 483 | 606 | 611 |
| 12819755 | TAF5 | Bos taurus TAF5 RNA polymerase II, TATA box binding protein (TBP)-associated factor, 100kDa | transcription | 2.09 | 2.368 | 2.296 | 100 | 131 | 93 | 132 | 42 | 50 | 57 | 58 | 48 | 57 | 43 | 45 | 56 |
| 12754960 | ZNF829 | Bos taurus cDNA clone IMAGE:8190822. | transcription | 2.431 | 2.256 | 2.254 | 111 | 149 | 94 | 110 | 58 | 42 | 54 | 49 | 54 | 40 | 55 | 45 | 52 |
| 12889145 | PSIP1 | Bos taurus PC4 and SFRS1 interacting protein 1 | transcription | 2.436 | 2.971 | 2.294 | 955 | 1091 | 816 | 1061 | 428 | 387 | 463 | 405 | 380 | 416 | 300 | 375 | 313 |
| 12715011 | TCFL5 | Bos taurus transcription factor-like 5 (basic helix-loop-helix), mRNA (cDNA clone MGC:137754 IMAGE:8168585), complete cds. | transcription | 2.66 | 2.871 | 2.07 | 134 | 112 | 103 | 141 | 60 | 57 | 59 | 59 | 48 | 34 | 43 | 39 | 45 |
| 12737211 | NR5A2 | Bos taurus nuclear receptor subfamily 5, group A, member 2 | transcription | 2.722 | 54.777 | 2.766 | 2640 | 2929 | 2832 | 2366 | 1105 | 764 | 1080 | 1161 | 606 | 1361 | 54 | 44 | 49 |
| 12877814 | ZNF358 | PREDICTED: Bos taurus zinc finger protein 358, transcript variant 1 | transcription | 2.763 | 2.111 | 2.039 | 334 | 328 | 509 | 265 | 228 | 150 | 146 | 140 | 116 | 124 | 193 | 160 | 146 |
| 12841158 | ITGB3BP | Bos taurus integrin beta 3 binding protein (beta3-endonexin) | transcription | 2.789 | 3.328 | 2.119 | 127 | 192 | 133 | 155 | 75 | 78 | 60 | 56 | 54 | 52 | 48 | 35 | 53 |
| 12902925 | CITED1 | Bos taurus Cbp/p300-interacting transactivator, with Glu/Asp-rich carboxy-terminal domain, 1 | transcription | 2.823 | 4.109 | 3.77 | 706 | 689 | 921 | 563 | 216 | 195 | 158 | 242 | 196 | 333 | 182 | 156 | 180 |
| 12784696 | IKZF2 | Bos taurus IKAROS family zinc finger 2 (Helios), mRNA (cDNA clone MGC:142349 IMAGE:8190718), complete cds. | transcription | 2.936 | 3.156 | 3.602 | 146 | 161 | 134 | 192 | 41 | 40 | 51 | 55 | 49 | 57 | 43 | 44 | 64 |
| 12868415 | YEATS4 | Bos taurus YEATS domain containing 4 | transcription | 2.97 | 2.799 | 2.553 | 461 | 538 | 422 | 806 | 244 | 224 | 172 | 198 | 151 | 199 | 205 | 179 | 195 |
| 12752672 | ZNF415 | Zinc Finger Protein 415 | transcription | 3.269 | 2.549 | 2.076 | 179 | 197 | 218 | 213 | 97 | 92 | 102 | 59 | 64 | 61 | 80 | 103 | 60 |
| 12720944 | C13H20orf20 | MRG/MORF4L Binding Protein (MRGBP, C20orf20) | transcription | 3.326 | 2.834 | 2.362 | 187 | 174 | 168 | 219 | 91 | 62 | 87 | 62 | 60 | 48 | 77 | 62 | 59 |
| 12709252 | FOXO1 | PREDICTED: Bos taurus forkhead box O1 | transcription | 3.351 | 2.028 | 2.42 | 2056 | 1924 | 1371 | 1546 | 700 | 726 | 684 | 566 | 554 | 418 | 915 | 931 | 693 |
| 12883086 | DNMT1 | Bos taurus DNA (cytosine-5-)-methyltransferase 1 | transcription | 3.426 | 3.281 | 2.131 | 511 | 540 | 431 | 349 | 220 | 201 | 214 | 132 | 144 | 120 | 123 | 147 | 145 |
| 12707529 | ZBTB6 | Bos taurus zinc finger and BTB domain containing 6 | transcription | 3.544 | 2.827 | 2.15 | 150 | 155 | 135 | 185 | 70 | 65 | 82 | 40 | 48 | 43 | 56 | 50 | 59 |
| 12858942 | RPAP3 | Bos taurus RNA polymerase II associated protein 3 | transcription | 3.926 | 4.215 | 2.814 | 500 | 605 | 588 | 634 | 219 | 199 | 200 | 151 | 142 | 150 | 164 | 144 | 110 |
| 12853746 | EZH2 | Bos taurus enhancer of zeste homolog 2 (Drosophila) | transcription | 3.941 | 5.751 | 2.691 | 548 | 562 | 410 | 397 | 169 | 183 | 176 | 92 | 135 | 140 | 78 | 78 | 92 |
| 12852052 | JAZF1 | Bos taurus JAZF zinc finger 1 | transcription | 4.309 | 3.565 | 2.352 | 716 | 792 | 673 | 721 | 336 | 292 | 297 | 162 | 169 | 174 | 245 | 198 | 173 |
| 12901117 | HEY2 | Bos taurus hairy/enhancer-of-split related with YRPW motif 2 | transcription | 4.505 | 21.517 | 4.584 | 1121 | 1090 | 845 | 687 | 248 | 145 | 223 | 231 | 180 | 204 | 38 | 40 | 51 |
| 12820083 | ZNF518A | PREDICTED: Bos taurus zinc finger protein 518A | transcription | 4.765 | 3.339 | 2.281 | 169 | 207 | 166 | 297 | 87 | 84 | 97 | 48 | 43 | 38 | 58 | 56 | 70 |
| 12712565 | DACH1 | Dachshund Family Transcription Factor 1 | transcription | 4.836 | 13.819 | 3.139 | 665 | 765 | 627 | 650 | 242 | 178 | 230 | 143 | 127 | 150 | 45 | 66 | 39 |
| 12720549 | E2F1 | Bos taurus E2F transcription factor 1 | transcription | 4.884 | 3.753 | 2.595 | 341 | 298 | 260 | 152 | 121 | 73 | 104 | 56 | 49 | 49 | 66 | 56 | 82 |
| 12813350 | ATF7IP2 | Bos taurus activating transcription factor 7 interacting protein 2 | transcription | 5.008 | 3.291 | 2.79 | 174 | 225 | 116 | 326 | 67 | 84 | 62 | 52 | 34 | 34 | 67 | 65 | 48 |
| 12832191 | E2F8 | Bos taurus E2F transcription factor 8 | transcription | 6.437 | 6.086 | 3.411 | 193 | 180 | 142 | 164 | 48 | 45 | 56 | 28 | 22 | 29 | 37 | 23 | 25 |
| 12715490 | MKX | Bos taurus mohawk homeobox | transcription | 7.481 | 20.053 | 2.696 | 521 | 533 | 532 | 659 | 227 | 192 | 204 | 88 | 55 | 86 | 26 | 25 | 34 |
| 12724318 | MYBL1 | Bos taurus v-myb myeloblastosis viral oncogene homolog (avian)-like 1 | transcription | 8.197 | 6.641 | 4.315 | 342 | 340 | 336 | 250 | 77 | 62 | 81 | 44 | 36 | 37 | 49 | 41 | 54 |
| 12707318 | BCL11A | Bos taurus B-cell CLL/lymphoma 11A (zinc finger protein) | transcription | 8.412 | 7.461 | 3.075 | 144 | 187 | 115 | 256 | 68 | 43 | 56 | 16 | 22 | 22 | 23 | 20 | 26 |
| 12689969 | MYEF2 | Bos taurus myelin expression factor 2 | transcription | 8.939 | 6.872 | 2.492 | 1110 | 1281 | 1139 | 1506 | 528 | 489 | 489 | 144 | 135 | 141 | 162 | 172 | 216 |
| 12783910 | CSRNP3 | Bos taurus cysteine-serine-rich nuclear protein 3 | transcription | 9.179 | 11.505 | 2.121 | 362 | 371 | 510 | 565 | 250 | 194 | 189 | 54 | 45 | 47 | 40 | 60 | 24 |
| 12752064 | ZNF536 | Zinc Finger Protein 536 | transcription | 11.292 | 16.652 | 2.772 | 365 | 356 | 471 | 403 | 160 | 126 | 145 | 28 | 29 | 55 | 25 | 19 | 29 |
| 12797985 | PPARG | Bos taurus peroxisome proliferator-activated receptor gamma | transcription | 14.537 | 21.837 | 4.533 | 560 | 599 | 284 | 724 | 127 | 107 | 107 | 29 | 32 | 47 | 36 | 17 | 21 |
| 12725510 | TOX | Bos taurus thymocyte selection-associated high mobility group box | transcription | 26.224 | 30.584 | 2.789 | 1054 | 1040 | 1508 | 982 | 401 | 429 | 385 | 38 | 49 | 42 | 36 | 32 | 43 |
| 12839301 | DEPDC1 | PREDICTED: Bos taurus DEP domain containing 1 | transcription | 34.474 | 29.348 | 4.567 | 442 | 458 | 461 | 445 | 126 | 75 | 102 | 16 | 13 | 10 | 24 | 15 | 10 |
| 12861319 | CAPRIN2 | Caprin Family Member 2 | translation | 2.969 | 3.657 | 3.028 | 1471 | 1888 | 1596 | 2070 | 562 | 589 | 573 | 604 | 476 | 700 | 490 | 401 | 549 |
| 12805748 | BOLA | Bos taurus MHC class I heavy chain, transcript variant 1 | unknown | -11.773 | -11.579 | -8.44 | 164 | 145 | 160 | 130 | 2754 | 591 | 1226 | 2217 | 825 | 2960 | 1504 | 1180 | 2905 |
| 12720500 | FAM171A1 | Bos taurus family with sequence similarity 171, member A1 | unknown | -10.604 | -6.385 | -3.544 | 134 | 127 | 99 | 50 | 259 | 441 | 346 | 1094 | 1067 | 907 | 683 | 612 | 554 |
| 12836058 | TAGLN2 | Bos taurus transgelin 2 | unknown | -7.302 | -4.254 | -4.6 | 227 | 204 | 263 | 78 | 844 | 873 | 714 | 1293 | 1351 | 1205 | 923 | 673 | 670 |
| 12728164 | GRAMD1B | Bos taurus GRAM domain containing 1B | unknown | -6.16 | -5.328 | -3.375 | 203 | 226 | 620 | 280 | 1089 | 1049 | 897 | 1710 | 1825 | 1996 | 1815 | 1065 | 2086 |
| 12721502 | ENSBTAT00000054742 | ENSBTAT00000054742 | unknown | -2.99 | -2.687 | -3.094 | 484 | 921 | 670 | 866 | 2156 | 2257 | 2208 | 2226 | 1908 | 2282 | 1769 | 2127 | 1872 |
| 12891206 | C8H9orf91 | Bos taurus chromosome 8 open reading frame, human C9orf91 | unknown | -2.967 | -4.011 | -2.076 | 164 | 156 | 173 | 138 | 343 | 298 | 339 | 493 | 421 | 487 | 758 | 493 | 669 |
| 12853801 | TMEM209 | PREDICTED: Bos taurus transmembrane protein 209 | unknown | 2.242 | 2.412 | 2.07 | 588 | 578 | 515 | 576 | 286 | 257 | 274 | 282 | 234 | 240 | 245 | 215 | 242 |
| 12871454 | KLHL5 | Kelch-Like Family Member 5 | unknown | 2.27 | 2.011 | 2.275 | 778 | 807 | 637 | 794 | 345 | 310 | 337 | 317 | 373 | 306 | 429 | 307 | 395 |
| 12890299 | KIAA0020 | Bos taurus KIAA0020 | unknown | 2.365 | 2.153 | 2.033 | 315 | 339 | 275 | 399 | 148 | 164 | 174 | 126 | 142 | 151 | 128 | 157 | 178 |
| 12865547 | KIAA1467 | PREDICTED: Bos taurus KIAA1467 ortholog | unknown | 2.385 | 4.322 | 2.016 | 404 | 406 | 448 | 419 | 242 | 193 | 191 | 214 | 127 | 199 | 106 | 80 | 108 |
| 12690936 | KLHDC1 | Bos taurus kelch domain containing 1 | unknown | 2.425 | 2.443 | 2.728 | 252 | 332 | 285 | 354 | 109 | 110 | 115 | 123 | 130 | 122 | 142 | 106 | 127 |
| 12849324 | BZW2 | Bos taurus basic leucine zipper and W2 domains 2 | unknown | 2.492 | 2.184 | 2.281 | 914 | 1017 | 749 | 1373 | 582 | 390 | 359 | 377 | 389 | 426 | 385 | 476 | 506 |
| 12826173 | C28H10orf107 | Bos taurus chromosome 28 open reading frame, human C10orf107 (C28H10orf107), transcript variant 2, mRNA. | unknown | 2.612 | 2.901 | 2.601 | 156 | 191 | 212 | 159 | 73 | 72 | 61 | 68 | 75 | 62 | 58 | 56 | 70 |
| 12770183 | SYNGR2 | Bos taurus synaptogyrin 2 | unknown | 2.711 | 3.113 | 2.479 | 1170 | 1170 | 1314 | 849 | 603 | 400 | 375 | 458 | 399 | 377 | 387 | 347 | 338 |
| 12747213 | C17H4orf29 | Bos taurus chromosome 17 open reading frame, human C4orf29 | unknown | 2.831 | 2.34 | 2.412 | 213 | 237 | 384 | 347 | 114 | 125 | 117 | 101 | 99 | 103 | 127 | 117 | 122 |
| 12725324 | CA8 | Carbonic Anhydrase VIII | unknown | 2.972 | 4.113 | 4.435 | 636 | 669 | 483 | 677 | 224 | 105 | 111 | 195 | 257 | 173 | 198 | 118 | 141 |
| 12793033 | XM_002696660 | PREDICTED: Bos taurus relaxin/insulin-like family peptide receptor 3-like (LOC530472), mRNA. | unknown | 2.985 | 3.325 | 2.037 | 163 | 153 | 185 | 124 | 102 | 60 | 71 | 46 | 58 | 52 | 52 | 37 | 52 |
| 12770098 | RDM1 | Bos taurus RAD52 motif 1 | unknown | 2.991 | 2.756 | 2.386 | 103 | 121 | 100 | 91 | 50 | 44 | 37 | 32 | 38 | 34 | 34 | 42 | 37 |
| 12899142 | NT5DC1 | Bos taurus 5'-nucleotidase domain containing 1 | unknown | 2.992 | 5.588 | 3.396 | 272 | 303 | 222 | 303 | 87 | 78 | 76 | 88 | 84 | 102 | 56 | 41 | 51 |
| 12680915 | DONSON | Bos taurus downstream neighbor of SON | unknown | 3.019 | 2.53 | 2.55 | 408 | 411 | 355 | 363 | 145 | 144 | 161 | 126 | 156 | 104 | 141 | 151 | 164 |
| 12689043 | LRRC49 | Leucine Rich Repeat Containing 49 | unknown | 3.09 | 3.71 | 2.556 | 126 | 106 | 132 | 140 | 49 | 51 | 48 | 42 | 42 | 38 | 32 | 40 | 30 |
| 12728295 | FAM76B | Bos taurus family with sequence similarity 76, member B | unknown | 3.146 | 2.681 | 2.062 | 190 | 194 | 124 | 184 | 68 | 112 | 74 | 59 | 65 | 42 | 59 | 69 | 63 |
| 12791247 | C21H14orf132 | Bos taurus chromosome 21 open reading frame, human C14orf132 | unknown | 3.172 | 3.226 | 2.198 | 320 | 292 | 168 | 149 | 75 | 106 | 126 | 54 | 74 | 83 | 64 | 63 | 78 |
| 12859746 | CCDC134 | Bos taurus coiled-coil domain containing 134 | unknown | 3.237 | 2.066 | 2.851 | 265 | 252 | 136 | 226 | 79 | 71 | 74 | 60 | 67 | 70 | 97 | 97 | 116 |
| 12905729 | MAP7D2 | MAP7 Domain Containing 2 | unknown | 3.416 | 3.045 | 2.621 | 106 | 137 | 104 | 276 | 57 | 49 | 58 | 43 | 38 | 45 | 45 | 44 | 52 |
| 12679281 | C1H3orf33 | Chromosome 3 Open Reading Frame 33 | unknown | 3.422 | 2.496 | 2.295 | 458 | 492 | 512 | 512 | 214 | 256 | 181 | 151 | 132 | 150 | 229 | 187 | 180 |
| 12851464 | C4H7orf60 | Chromosome 7 Open Reading Frame 60 | unknown | 3.429 | 3.201 | 2.8 | 855 | 852 | 679 | 1252 | 347 | 266 | 343 | 266 | 245 | 266 | 278 | 294 | 260 |
| 12725347 | RALYL | Bos taurus RALY RNA binding protein-like | unknown | 3.539 | 10.073 | 2.336 | 211 | 233 | 243 | 203 | 134 | 60 | 106 | 59 | 54 | 78 | 26 | 20 | 21 |
| 12823052 | MORN4 | Bos taurus MORN repeat containing 4 | unknown | 3.629 | 2.793 | 3.619 | 443 | 410 | 609 | 363 | 209 | 89 | 102 | 146 | 112 | 115 | 177 | 175 | 133 |
| 12679305 | LNP1 | Bos taurus leukemia NUP98 fusion partner 1 | unknown | 3.717 | 2.683 | 2.041 | 210 | 205 | 248 | 249 | 116 | 112 | 106 | 74 | 55 | 56 | 91 | 73 | 90 |
| 12689471 | PLEKHH1 | Pleckstrin Homology Domain Containing, Family H (With MyTH4 Domain) Member 1 | unknown | 3.851 | 4.169 | 2.876 | 202 | 196 | 181 | 187 | 75 | 60 | 66 | 60 | 38 | 54 | 34 | 46 | 62 |
| 12759859 | BC148976 | Bos taurus cDNA clone IMAGE:8228065. | unknown | 3.92 | 2.464 | 3.029 | 106 | 172 | 143 | 159 | 59 | 41 | 44 | 33 | 34 | 44 | 36 | 69 | 78 |
| 12867751 | BC148984 | Bos taurus cDNA clone IMAGE:8310512. | unknown | 3.988 | 3.773 | 2.01 | 159 | 161 | 107 | 165 | 96 | 72 | 55 | 40 | 38 | 32 | 37 | 28 | 55 |
| 12872103 | C4orf21 | Zinc Finger, GRF-Type Containing 1 | unknown | 4.02 | 3.676 | 2.16 | 144 | 154 | 118 | 121 | 66 | 62 | 58 | 33 | 33 | 34 | 30 | 46 | 35 |
| 12906512 | BC126800 | Bos taurus cDNA clone MGC:148868 IMAGE:8258061, complete cds. | unknown | 4.243 | 11.649 | 2.078 | 246 | 315 | 306 | 364 | 164 | 137 | 140 | 84 | 51 | 86 | 20 | 17 | 51 |
| 12816534 | STYXL1 | Bos taurus serine/threonine/tyrosine interacting-like 1 | unknown | 4.318 | 3.331 | 2.698 | 387 | 469 | 487 | 549 | 241 | 168 | 130 | 79 | 142 | 116 | 123 | 139 | 163 |
| 12852506 | XM_002684117 | PREDICTED: Bos taurus uncharacterized LOC786657 | unknown | 4.508 | 4.389 | 2.268 | 177 | 217 | 216 | 183 | 87 | 87 | 87 | 52 | 41 | 40 | 35 | 52 | 50 |
| 12861056 | TMEM194A | Bos taurus transmembrane protein 194A | unknown | 4.603 | 4.873 | 2.233 | 575 | 503 | 389 | 452 | 241 | 206 | 193 | 101 | 99 | 109 | 105 | 97 | 91 |
| 12905441 | SPIN4 | PREDICTED: Bos taurus spindlin family, member 4 | unknown | 4.838 | 3.313 | 2.275 | 159 | 157 | 179 | 144 | 61 | 79 | 71 | 30 | 32 | 36 | 42 | 62 | 43 |
| 12821102 | ENSBTAT00000039939 | ENSBTAT00000039939 | unknown | 4.949 | 7.726 | 3.072 | 387 | 388 | 348 | 321 | 139 | 104 | 111 | 79 | 67 | 72 | 55 | 37 | 50 |
| 12731291 | CCDC34 | Coiled-Coil Domain Containing 34 | unknown | 4.963 | 4.469 | 3.781 | 156 | 177 | 107 | 156 | 45 | 28 | 46 | 44 | 20 | 30 | 29 | 31 | 39 |
| 12808600 | C24H18orf54 | Bos taurus chromosome 24 open reading frame, human C18orf54 | unknown | 5.438 | 4.3 | 2.563 | 119 | 147 | 140 | 160 | 70 | 52 | 46 | 29 | 28 | 21 | 37 | 28 | 34 |
| 12878194 | BC151570 | Bos taurus cDNA clone IMAGE:8314962, partial cds. | unknown | 5.733 | 8.558 | 3.99 | 262 | 246 | 141 | 314 | 65 | 46 | 65 | 53 | 46 | 27 | 32 | 29 | 22 |
| 12827978 | DNAJC9 | Bos taurus DnaJ (Hsp40) homolog, subfamily C, member 9 | unknown | 5.857 | 4.939 | 2.852 | 325 | 342 | 292 | 331 | 117 | 106 | 116 | 50 | 55 | 61 | 64 | 58 | 75 |
| 12872704 | XM_002688173 | PREDICTED: Bos taurus histone cluster 2, H2be-like (LOC782350) | unknown | 6.105 | 6.146 | 2.12 | 164 | 252 | 167 | 152 | 118 | 65 | 80 | 38 | 22 | 31 | 38 | 25 | 26 |
| 12855425 | PRR15 | Bos taurus proline rich 15 | unknown | 6.271 | 5.288 | 2.916 | 239 | 239 | 291 | 235 | 95 | 86 | 77 | 43 | 42 | 35 | 44 | 53 | 46 |
| 12737711 | C16H1orf112 | C16H1orf112 | unknown | 6.552 | 5.471 | 2.238 | 147 | 134 | 91 | 86 | 47 | 41 | 64 | 14 | 21 | 17 | 24 | 21 | 17 |
| 12847098 | CCDC18 | PREDICTED: Bos taurus coiled-coil domain containing 18 | unknown | 7.196 | 5.446 | 2.766 | 194 | 255 | 178 | 175 | 79 | 62 | 75 | 25 | 32 | 25 | 37 | 32 | 40 |
| 12898727 | FAM184A | PREDICTED: Bos taurus family with sequence similarity 184, member A, transcript variant 2 | unknown | 7.739 | 5.506 | 4.074 | 153 | 174 | 167 | 155 | 40 | 35 | 45 | 20 | 16 | 28 | 23 | 34 | 33 |
| 12723771 | SAMD12 | Bos taurus sterile alpha motif domain containing 12 | unknown | 7.828 | 6.774 | 4.286 | 196 | 178 | 162 | 127 | 46 | 25 | 47 | 22 | 22 | 19 | 31 | 18 | 25 |
| 12906608 | ENSBTAT00000040183 | ENSBTAT00000040183 | unknown | 9.09 | 7.707 | 3.728 | 151 | 142 | 148 | 187 | 45 | 40 | 40 | 19 | 19 | 14 | 20 | 26 | 16 |
| 12765398 | PRR11 | Bos taurus proline rich 11 | unknown | 9.136 | 11.566 | 3.571 | 251 | 295 | 317 | 237 | 91 | 68 | 73 | 27 | 31 | 31 | 27 | 16 | 30 |
| 12804914 | XM_002697515 | PREDICTED: Bos taurus histone H2B type 1-like (LOC505183) | unknown | 9.206 | 8.296 | 2.424 | 2044 | 2066 | 1512 | 1183 | 874 | 578 | 632 | 223 | 164 | 159 | 212 | 244 | 155 |
| 12726392 | C15H11orf93 | PREDICTED: Bos taurus uncharacterized LOC100294918 (LOC100294918) | unknown | 9.495 | 11.83 | 3.827 | 205 | 239 | 206 | 252 | 73 | 63 | 43 | 19 | 23 | 31 | 15 | 23 | 20 |
| 12805856 | XM_002697507 | PREDICTED: Bos taurus histone H2B type 1-like (LOC521580) | unknown | 9.781 | 13.359 | 3.184 | 202 | 136 | 174 | 176 | 78 | 43 | 45 | 16 | 20 | 16 | 15 | 15 | 9 |
| 12807147 | XM_002697517 | PREDICTED: Bos taurus histone H2B type 1-like (LOC787465) | unknown | 10.064 | 8.655 | 2.048 | 2977 | 2611 | 2912 | 2325 | 1771 | 1100 | 1167 | 288 | 281 | 236 | 367 | 286 | 287 |
| 12910004 | CXHXorf30 | PREDICTED: Bos taurus chromosome X open reading frame, human CXorf30 | unknown | 10.886 | 6.508 | 3.307 | 142 | 138 | 162 | 141 | 36 | 52 | 45 | 13 | 14 | 12 | 25 | 25 | 18 |
| 12784152 | FAM171B | Bos taurus family with sequence similarity 171, member B | unknown | 11.994 | 42.403 | 2.97 | 922 | 925 | 826 | 1105 | 324 | 281 | 347 | 91 | 59 | 90 | 20 | 22 | 26 |
| 12804021 | XM_003583905 | PREDICTED: Bos taurus histone cluster 1, H3a-like (LOC788250) | unknown | 12.103 | 18.94 | 2.559 | 1477 | 1313 | 1445 | 1207 | 662 | 497 | 453 | 124 | 74 | 154 | 79 | 58 | 80 |
| 12796824 | LRRC2 | Bos taurus leucine rich repeat containing 2 | unknown | 12.353 | 2.928 | 3.019 | 1016 | 1134 | 940 | 1022 | 398 | 316 | 311 | 129 | 82 | 54 | 352 | 569 | 214 |
| 12794225 | MIR1284 | Bos taurus microRNA mir-1284, microRNA. | unknown | 12.365 | 7.73 | 2.966 | 102 | 115 | 115 | 178 | 44 | 44 | 38 | 9 | 13 | 8 | 13 | 17 | 19 |
| 12891544 | LINGO2 | PREDICTED: Bos taurus leucine rich repeat and Ig domain containing 2, transcript variant 1 | unknown | 12.855 | 19.106 | 3.536 | 259 | 315 | 300 | 406 | 102 | 81 | 85 | 26 | 30 | 19 | 13 | 23 | 15 |
| 12800563 | PPARG-TSEN2 | Bos taurus PPARG-TSEN2, transcript variant 1, non-coding RNA. | unknown | 14.079 | 14.557 | 4.699 | 465 | 479 | 198 | 464 | 96 | 85 | 63 | 30 | 22 | 29 | 34 | 32 | 16 |
| 12910294 | CXHXorf22 | PREDICTED: Bos taurus chromosome X open reading frame, human CXorf22 | unknown | 14.157 | 9.956 | 3.305 | 224 | 248 | 333 | 266 | 61 | 86 | 97 | 17 | 17 | 22 | 26 | 31 | 23 |
| 12850069 | ENSBTAT00000025667 | cdna:pseudogene chromosome:UMD3.1:4:33507617:33508901:1 | unknown | 17.025 | 16.492 | 3.564 | 169 | 189 | 183 | 219 | 58 | 43 | 59 | 11 | 11 | 12 | 9 | 17 | 11 |
| 12896448 | XM_002690146 | PREDICTED: Bos taurus uncharacterized LOC100336854 (LOC100336854) | unknown | 20.351 | 20.049 | 5.821 | 344 | 380 | 226 | 440 | 85 | 48 | 48 | 15 | 21 | 14 | 20 | 11 | 22 |
| 12704512 | FAM78A | Bos taurus chromosome 9 open reading frame 59 (C9orf59), mRNA, complete cds. | unknown | 21.572 | 16.865 | 2.529 | 2152 | 2036 | 1663 | 2470 | 1063 | 789 | 644 | 85 | 144 | 71 | 141 | 119 | 109 |
| 12829082 | H19 | Bos taurus H19, imprinted maternally expressed transcript, non-coding RNA. | untranslated RNA | -13.67 | -5.889 | -27.714 | 108 | 112 | 99 | 78 | 2355 | 3363 | 2551 | 1724 | 1174 | 1198 | 551 | 576 | 611 |
