# Supplementary material for: Transcriptomes of bovine ovarian follicular and luteal cells
Source: Data Brief. 2016 Dec 10;10:335–9. doi: 10.1016/j.dib.2016.11.093 (PMC5157705; doi:10.1016/j.dib.2016.11.093)
Supplement: Supplementary file 3 — Supplementary material [file mmc3.docx]

| **Table 2. Genes Enriched (≥ 2-fold greater expression than all other cells types) or Decreased (≤ -2-fold) in Theca Cells** | | | | |  |  | **Linear Microarray Results (arbitrary units)** | | | | | | | | | | | | |
| --- | --- | --- | --- | --- | --- | --- | --- | --- | --- | --- | --- | --- | --- | --- | --- | --- | --- | --- | --- |
| **Probeset ID** | **Gene Symbol** | **Description** | **Functional Category** | **Fold Change (TC vs LLC)** | **Fold Change (TC vs SLC)** | **Fold Change (TC vs GC)** | **GC1** | **GC2** | **GC3** | **GC4** | **TC1** | **TC2** | **TC3** | **LLC1** | **LLC2** | **LLC3** | **SLC1** | **SLC2** | **SLC3** |
| 12726044 | SDC2 | Syndecan 2 | adhesion | 2.203 | 3.707 | 9.276 | 109 | 97 | 122 | 116 | 742 | 1327 | 1094 | 412 | 541 | 452 | 334 | 189 | 335 |
| 12719021 | ITGA8 | PREDICTED: Bos taurus integrin, alpha 8 | adhesion | 2.538 | 5.552 | 13.264 | 23 | 19 | 15 | 35 | 279 | 408 | 225 | 134 | 125 | 94 | 50 | 71 | 42 |
| 12700159 | CERCAM | Bos taurus cerebral endothelial cell adhesion molecule | adhesion | 2.553 | 2.244 | 2.309 | 65 | 73 | 83 | 68 | 210 | 159 | 138 | 74 | 74 | 50 | 86 | 67 | 70 |
| 12709784 | GPC5 | Bos taurus glypican 5 | adhesion | 2.77 | 2.518 | 11.873 | 13 | 14 | 11 | 24 | 143 | 182 | 212 | 73 | 70 | 51 | 78 | 52 | 84 |
| 12733466 | THY1 | Bos taurus Thy-1 cell surface antigen | adhesion | 2.791 | 4.965 | 16.611 | 31 | 23 | 18 | 21 | 345 | 508 | 305 | 176 | 133 | 105 | 73 | 109 | 55 |
| 12749287 | CDH3 | Bos taurus cadherin 3, type 1, P-cadherin (placental) | adhesion | 3.218 | 2.199 | 3.717 | 57 | 57 | 59 | 58 | 219 | 345 | 131 | 63 | 68 | 69 | 101 | 107 | 86 |
| 12828494 | NID1 | Bos taurus nidogen 1 | adhesion | 3.693 | 5.172 | 17.672 | 44 | 42 | 54 | 52 | 696 | 1007 | 863 | 295 | 245 | 166 | 162 | 198 | 136 |
| 12845414 | GJB5 | Bos taurus gap junction protein, beta 5, 31.1kDa | adhesion | 4.827 | 3.536 | 9.763 | 41 | 27 | 34 | 36 | 451 | 366 | 227 | 89 | 78 | 48 | 144 | 76 | 78 |
| 12834537 | CDON | Bos taurus Cdon homolog (mouse) | adhesion | 7.39 | 4.213 | 5.534 | 62 | 63 | 58 | 62 | 300 | 431 | 302 | 45 | 50 | 43 | 60 | 100 | 88 |
| 12851548 | TSPAN33 | Tetraspanin 33 | adhesion | 7.979 | 4.383 | 4.304 | 166 | 166 | 213 | 134 | 903 | 731 | 566 | 84 | 100 | 87 | 186 | 178 | 134 |
| 12834558 | FAT3 | Bos taurus FAT tumor suppressor homolog 3 (Drosophila) | adhesion | 8.064 | 5.38 | 6.043 | 28 | 27 | 31 | 29 | 163 | 275 | 117 | 18 | 29 | 19 | 23 | 47 | 32 |
| 12684080 | CLDN11 | Bos taurus claudin 11 | adhesion | 20.488 | 18.267 | 18.56 | 62 | 66 | 65 | 58 | 904 | 1060 | 1647 | 61 | 53 | 57 | 54 | 58 | 81 |
| 12851996 | DFNA5 | Bos taurus deafness, autosomal dominant 5 | apoptosis | 4.898 | 25.316 | 2.323 | 162 | 172 | 270 | 134 | 425 | 363 | 461 | 133 | 34 | 133 | 16 | 21 | 13 |
| 12809257 | COLEC12 | Bos taurus collectin sub-family member 12 | carbohydrate-protein binding | 3.212 | 3.658 | 8.143 | 38 | 38 | 65 | 30 | 266 | 314 | 445 | 122 | 91 | 100 | 70 | 83 | 131 |
| 12841545 | REG4 | Bos taurus regenerating islet-derived family, member 4 | carbohydrate-protein binding | 10.265 | 6.133 | 2.945 | 49 | 48 | 63 | 20 | 157 | 70 | 163 | 14 | 12 | 10 | 18 | 20 | 21 |
| 12686659 | CHODL | Bos taurus chondrolectin | carbohydrate-protein binding | 12.519 | 11.328 | 6.752 | 38 | 32 | 26 | 24 | 130 | 187 | 324 | 15 | 15 | 18 | 14 | 21 | 18 |
| 12765572 | H3F3B | Bos taurus H3 histone, family 3B | chromosome structure | -4.858 | -3.631 | -2.428 | 281 | 268 | 321 | 572 | 204 | 115 | 121 | 664 | 601 | 809 | 549 | 392 | 626 |
| 12691034 | DNAL1 | Dynein, Axonemal, Light Chain 1 | cilia | -2.51 | -2.181 | -2.243 | 389 | 411 | 328 | 353 | 178 | 135 | 184 | 379 | 384 | 484 | 324 | 370 | 385 |
| 12822288 | ACTA2 | Bos taurus actin, alpha 2, smooth muscle, aorta | cytoskeletal dynamics | 2.32 | 9.901 | 22.019 | 241 | 188 | 151 | 123 | 3484 | 5113 | 2968 | 1332 | 1513 | 2101 | 314 | 497 | 349 |
| 12786332 | STMN1 | Stathmin 1 (ENSBTAT00000037688) | cytoskeletal dynamics | 2.436 | 2.128 | 2.906 | 223 | 174 | 197 | 175 | 538 | 592 | 538 | 211 | 297 | 189 | 288 | 237 | 260 |
| 12727781 | TAGLN | Bos taurus transgelin | cytoskeletal dynamics | 2.718 | 10.048 | 27.409 | 57 | 50 | 48 | 29 | 1281 | 1519 | 953 | 462 | 395 | 506 | 87 | 177 | 119 |
| 12892875 | TPM2 | Bos taurus tropomyosin 2 (beta) | cytoskeletal dynamics | 2.763 | 5.377 | 3.189 | 191 | 213 | 329 | 447 | 906 | 1132 | 682 | 280 | 298 | 398 | 89 | 209 | 240 |
| 12825451 | PDLIM3 | Bos taurus PDZ and LIM domain 3 | cytoskeletal dynamics | 2.959 | 18.724 | 30.683 | 50 | 54 | 47 | 39 | 1339 | 1652 | 1377 | 413 | 518 | 550 | 84 | 91 | 60 |
| 12683570 | MYLK | Bos taurus myosin light chain kinase | cytoskeletal dynamics | 3.127 | 3.234 | 22.818 | 40 | 48 | 64 | 39 | 1035 | 1241 | 955 | 352 | 338 | 337 | 241 | 459 | 328 |
| 12881080 | CNN2 | Bos taurus calponin 2 | cytoskeletal dynamics | 3.174 | 5.148 | 9.512 | 73 | 75 | 85 | 70 | 715 | 877 | 596 | 241 | 215 | 226 | 117 | 162 | 145 |
| 12774318 | DES | Bos taurus desmin | cytoskeletal dynamics | 3.325 | 4.989 | 4.49 | 31 | 38 | 40 | 33 | 126 | 367 | 87 | 42 | 52 | 50 | 29 | 36 | 31 |
| 12824980 | GPM6A | Bos taurus glycoprotein M6A | cytoskeletal dynamics | 3.355 | 4.396 | 20.734 | 52 | 43 | 27 | 30 | 544 | 1060 | 775 | 262 | 299 | 151 | 173 | 198 | 154 |
| 12886881 | TPM4 | Bos taurus tropomyosin 4 | cytoskeletal dynamics | 5.385 | 4.916 | 6.1 | 98 | 120 | 111 | 141 | 725 | 982 | 507 | 125 | 142 | 131 | 126 | 165 | 146 |
| 12804435 | AIF1 | Allograft Inflammatory Factor 1 | cytoskeletal dynamics | 5.622 | 3.423 | 3.303 | 24 | 33 | 59 | 23 | 119 | 78 | 131 | 15 | 19 | 25 | 24 | 36 | 36 |
| 12698109 | PLEKHH2 | Pleckstrin Homology Domain Containing, Family H (With MyTH4 Domain) Member 2 | cytoskeletal dynamics | 7.128 | 5.105 | 3.076 | 124 | 142 | 109 | 149 | 344 | 527 | 353 | 58 | 54 | 56 | 75 | 70 | 91 |
| 12703851 | ACTG2 | Bos taurus actin, gamma 2, smooth muscle, enteric | cytoskeletal dynamics | 10.218 | 12.673 | 12.359 | 159 | 122 | 101 | 244 | 1755 | 2364 | 1479 | 186 | 190 | 163 | 90 | 148 | 226 |
| 12681397 | PLOD2 | Bos taurus procollagen-lysine, 2-oxoglutarate 5-dioxygenase 2 | extracellular matrix | -5.482 | -4.446 | -3.665 | 568 | 634 | 528 | 674 | 140 | 157 | 197 | 937 | 934 | 819 | 899 | 508 | 836 |
| 12711158 | COL4A1 | Bos taurus collagen, type IV, alpha 1 | extracellular matrix | 2.13 | 2.736 | 3.405 | 180 | 152 | 197 | 139 | 583 | 610 | 503 | 314 | 251 | 235 | 191 | 213 | 214 |
| 12734598 | PRELP | Proline/Arginine-Rich End Leucine-Rich Repeat Protein | extracellular matrix | 2.442 | 4.262 | 3.265 | 178 | 217 | 124 | 184 | 748 | 650 | 366 | 300 | 234 | 174 | 104 | 142 | 155 |
| 12707220 | COL5A1 | Collagen, Type V, Alpha 1 | extracellular matrix | 2.525 | 2.388 | 3.849 | 229 | 177 | 139 | 126 | 707 | 857 | 409 | 292 | 228 | 231 | 245 | 282 | 264 |
| 12815112 | ELN | Bos taurus elastin | extracellular matrix | 2.636 | 4.398 | 6.821 | 49 | 56 | 51 | 44 | 609 | 253 | 259 | 157 | 130 | 107 | 67 | 93 | 75 |
| 12863901 | DCN | Bos taurus decorin | extracellular matrix | 2.922 | 3.723 | 22.552 | 148 | 162 | 181 | 94 | 2554 | 3522 | 3676 | 1327 | 920 | 1085 | 924 | 976 | 710 |
| 12749561 | MMP2 | Matrix Metallopeptidase 2 | extracellular matrix | 3.063 | 2.46 | 32.011 | 36 | 60 | 38 | 30 | 1057 | 1582 | 1233 | 468 | 463 | 331 | 430 | 680 | 473 |
| 12791675 | LOXL1 | Bos taurus lysyl oxidase-like 1 | extracellular matrix | 3.095 | 3.205 | 12.962 | 24 | 49 | 56 | 17 | 388 | 578 | 327 | 128 | 124 | 156 | 132 | 99 | 171 |
| 12786794 | EGFLAM | EGF-Like, Fibronectin Type III And Laminin G Domains | extracellular matrix | 3.935 | 11.898 | 25.479 | 27 | 24 | 24 | 31 | 623 | 851 | 572 | 220 | 128 | 177 | 73 | 59 | 42 |
| 12693498 | LTBP2 | Latent Transforming Growth Factor Beta Binding Protein 2 | extracellular matrix | 3.948 | 3.775 | 4.805 | 32 | 26 | 29 | 27 | 180 | 137 | 104 | 37 | 41 | 27 | 35 | 43 | 32 |
| 12834503 | EFEMP2 | Bos taurus EGF containing fibulin-like extracellular matrix protein 2 | extracellular matrix | 4.564 | 5.441 | 7.564 | 53 | 57 | 78 | 101 | 554 | 680 | 392 | 101 | 128 | 120 | 108 | 94 | 90 |
| 12761228 | MFAP4 | Bos taurus microfibrillar-associated protein 4 | extracellular matrix | 4.58 | 7.703 | 12.322 | 43 | 29 | 35 | 29 | 557 | 401 | 321 | 77 | 99 | 97 | 53 | 55 | 53 |
| 12781192 | COL3A1 | Bos taurus collagen, type III, alpha 1 | extracellular matrix | 5.252 | 4.552 | 33.611 | 43 | 49 | 34 | 35 | 1175 | 1461 | 1401 | 266 | 259 | 241 | 364 | 291 | 241 |
| 12723659 | COL14A1 | Collagen, Type XIV, Alpha 1 | extracellular matrix | 7.074 | 12.705 | 21.438 | 52 | 63 | 71 | 52 | 1118 | 1710 | 1057 | 187 | 173 | 176 | 89 | 120 | 92 |
| 12774541 | MFAP2 | Bos taurus microfibrillar-associated protein 2 | extracellular matrix | 9.638 | 10.604 | 3.722 | 112 | 92 | 86 | 93 | 403 | 402 | 277 | 29 | 42 | 41 | 36 | 44 | 24 |
| 12881226 | FBN2 | Fibrillin 2 | extracellular matrix | 10.089 | 11.217 | 11.015 | 20 | 17 | 21 | 20 | 226 | 239 | 176 | 18 | 28 | 18 | 21 | 17 | 18 |
| 12845908 | COL6A3 | Collagen, Type VI, Alpha 3 | extracellular matrix | 10.641 | 10.15 | 11.936 | 30 | 30 | 38 | 35 | 368 | 513 | 319 | 41 | 28 | 43 | 45 | 32 | 40 |
| 12848984 | COL1A2 | Bos taurus collagen, type I, alpha 2 | extracellular matrix | 10.916 | 6.144 | 32.771 | 63 | 54 | 56 | 42 | 1590 | 1947 | 1713 | 184 | 183 | 122 | 260 | 318 | 276 |
| 12899625 | COL12A1 | Bos taurus collagen, type XII, alpha 1 | extracellular matrix | 11.63 | 27.057 | 39.363 | 65 | 57 | 46 | 34 | 1995 | 2311 | 1568 | 192 | 158 | 151 | 67 | 90 | 60 |
| 12760992 | COL1A1 | Bos taurus collagen, type I, alpha 1 | extracellular matrix | 13.762 | 13.378 | 22.335 | 117 | 118 | 133 | 81 | 2644 | 2866 | 1980 | 239 | 165 | 146 | 128 | 287 | 171 |
| 12876752 | LOX | Bos taurus lysyl oxidase | extracellular matrix | 24.797 | 13.161 | 54.3 | 32 | 35 | 46 | 38 | 2112 | 2091 | 1904 | 71 | 100 | 78 | 105 | 172 | 204 |
| 12892568 | OMD | Osteomodulin | extracellular matrix | 26.211 | 43.521 | 47.818 | 16 | 22 | 16 | 18 | 839 | 859 | 898 | 39 | 28 | 33 | 16 | 23 | 21 |
| 12877752 | CFD | Complement Factor D (Adipsin) | immune response | 2.517 | 2.449 | 3.661 | 39 | 61 | 105 | 36 | 311 | 179 | 148 | 84 | 80 | 76 | 58 | 122 | 79 |
| 12881782 | CCL25 | Bos taurus chemokine (C-C motif) ligand 25 | immune response | 2.95 | 2.476 | 2.046 | 114 | 116 | 106 | 118 | 231 | 200 | 270 | 70 | 83 | 85 | 82 | 101 | 99 |
| 12781080 | C1QC | Bos taurus complement component 1, q subcomponent, C chain | immune response | 4.81 | 3.117 | 3.417 | 53 | 57 | 206 | 43 | 372 | 169 | 237 | 61 | 46 | 47 | 47 | 112 | 93 |
| 12835956 | FCGR3A | Bos taurus Fc fragment of IgG, low affinity IIIa, receptor (CD16a), transcript variant 1 | immune response | 5.442 | 5.953 | 4.893 | 34 | 33 | 50 | 32 | 195 | 114 | 254 | 39 | 29 | 31 | 24 | 37 | 30 |
| 12783013 | C1QA | Bos taurus complement component 1, q subcomponent, A chain | immune response | 9.399 | 5.36 | 6.307 | 40 | 27 | 201 | 22 | 581 | 175 | 250 | 28 | 25 | 43 | 44 | 74 | 51 |
| 12784435 | C1QB | Bos taurus complement component 1, q subcomponent, B chain | immune response | 13.743 | 7.531 | 14.507 | 124 | 114 | 1001 | 66 | 1935 | 1138 | 1301 | 92 | 95 | 126 | 101 | 406 | 164 |
| 12782940 | CYBRD1 | Bos taurus cytochrome b reductase 1 | ion conversion | 2 | 2.002 | 4.81 | 115 | 150 | 183 | 167 | 575 | 941 | 719 | 353 | 401 | 343 | 377 | 359 | 358 |
| 12704110 | SLC8A1 | Bos taurus solute carrier family 8 (sodium/calcium exchanger), member 1 | ion transport | 2.308 | 3.888 | 4.663 | 34 | 31 | 31 | 38 | 124 | 180 | 165 | 56 | 70 | 75 | 40 | 40 | 39 |
| 12844852 | ATP1A2 | Bos taurus ATPase, Na+/K+ transporting, alpha 2 polypeptide | ion transport | 5.187 | 4.668 | 12.223 | 58 | 47 | 70 | 43 | 925 | 676 | 448 | 124 | 120 | 135 | 105 | 198 | 133 |
| 12706341 | KCNG3 | PREDICTED: Bos taurus potassium voltage-gated channel, subfamily G, member 3 | ion transport | 8.269 | 4.283 | 3.134 | 47 | 59 | 85 | 125 | 224 | 209 | 262 | 23 | 26 | 36 | 46 | 36 | 93 |
| 12786517 | KCNIP1 | Bos taurus Kv channel interacting protein 1 | ion transport | 10.5 | 6.796 | 7.725 | 22 | 20 | 22 | 20 | 199 | 143 | 148 | 22 | 12 | 14 | 25 | 29 | 18 |
| 12791755 | CHRNA3 | Cholinergic Receptor, Nicotinic, Alpha 3 (Neuronal) | ion transport | 22.646 | 23.9 | 9.901 | 22 | 25 | 48 | 29 | 244 | 376 | 273 | 11 | 17 | 12 | 13 | 11 | 13 |
| 12902661 | NRK | Nik Related Kinase | kinase | 17.413 | 14.154 | 14.233 | 25 | 35 | 22 | 32 | 304 | 453 | 450 | 25 | 20 | 23 | 19 | 46 | 25 |
| 12871633 | ELOVL6 | Bos taurus ELOVL fatty acid elongase 6 | lipid metabolism | -2.416 | -3.181 | -2.985 | 1053 | 1053 | 892 | 1058 | 318 | 281 | 435 | 806 | 772 | 882 | 1173 | 749 | 1424 |
| 12872205 | ARSJ | PREDICTED: Bos taurus arylsulfatase family, member J | lipid metabolism | 2.93 | 3.95 | 8.201 | 31 | 28 | 31 | 21 | 221 | 220 | 236 | 99 | 61 | 76 | 53 | 44 | 81 |
| 12886857 | CYP4F3 | Bos taurus cytochrome P450, family 4, subfamily F, polypeptide 3 | lipid metabolism | 17.721 | 17.084 | 4.778 | 88 | 119 | 65 | 123 | 552 | 279 | 618 | 36 | 21 | 23 | 27 | 29 | 24 |
| 12896617 | NT5E | 5'-Nucleotidase, Ecto (CD73) | metabolism | -2.382 | -3.917 | -6.496 | 1576 | 1433 | 1538 | 93 | 525 | 63 | 381 | 1311 | 841 | 1099 | 1680 | 1817 | 1765 |
| 12694398 | PSME1 | Bos taurus proteasome (prosome, macropain) activator subunit 1 (PA28 alpha) | metabolism | 2.139 | 2.253 | 2.194 | 1265 | 1289 | 1129 | 1134 | 2565 | 2324 | 3079 | 1184 | 1201 | 1318 | 1239 | 1023 | 1267 |
| 12790303 | PCSK6 | Proprotein Convertase Subtilisin/Kexin Type 6 | metabolism | 2.336 | 2.386 | 3.357 | 39 | 34 | 40 | 25 | 111 | 128 | 104 | 48 | 53 | 45 | 51 | 51 | 42 |
| 12742874 | GSTT1 | Bos taurus glutathione S-transferase theta 1 | metabolism | 2.515 | 2.438 | 2.568 | 136 | 157 | 193 | 87 | 370 | 319 | 373 | 138 | 137 | 145 | 144 | 143 | 147 |
| 12869680 | HTRA3 | HtrA Serine Peptidase 3 | metabolism | 2.8 | 2.394 | 2.607 | 70 | 66 | 85 | 62 | 201 | 226 | 135 | 62 | 74 | 61 | 90 | 86 | 57 |
| 12873369 | ATP5I | Bos taurus ATP synthase, H+ transporting, mitochondrial Fo complex, subunit E, nuclear gene encoding mitochondrial protein | metabolism | 2.971 | 2.96 | 2.462 | 171 | 144 | 156 | 160 | 456 | 324 | 397 | 148 | 138 | 109 | 161 | 100 | 141 |
| 12688778 | PSME2 | Bos taurus proteasome (prosome, macropain) activator subunit 2 (PA28 beta) | metabolism | 3.073 | 2.998 | 2.676 | 406 | 437 | 311 | 335 | 825 | 873 | 1333 | 291 | 323 | 352 | 315 | 313 | 361 |
| 12897737 | ENPP3 | Bos taurus ectonucleotide pyrophosphatase/phosphodiesterase 3 | metabolism | 3.372 | 2.415 | 6.51 | 18 | 22 | 22 | 32 | 108 | 173 | 180 | 37 | 43 | 55 | 56 | 52 | 83 |
| 12825066 | MBOAT4 | Bos taurus membrane bound O-acyltransferase domain containing 4 | metabolism | 3.547 | 3.984 | 3.641 | 61 | 41 | 48 | 43 | 179 | 219 | 134 | 57 | 39 | 53 | 42 | 36 | 54 |
| 12723002 | PI15 | Bos taurus peptidase inhibitor 15 | metabolism | 5.443 | 3.65 | 35.53 | 22 | 21 | 18 | 19 | 524 | 772 | 851 | 120 | 139 | 128 | 180 | 193 | 203 |
| 12819205 | AS3MT | Bos taurus arsenic (+3 oxidation state) methyltransferase | metabolism | 6.129 | 21.453 | 6.709 | 40 | 60 | 109 | 66 | 417 | 422 | 461 | 62 | 65 | 87 | 20 | 25 | 17 |
| 12905259 | PRPS2 | Bos taurus phosphoribosyl pyrophosphate synthetase 2 | metabolism | 6.878 | 6.244 | 2.35 | 354 | 334 | 271 | 681 | 985 | 915 | 819 | 117 | 149 | 130 | 131 | 138 | 168 |
| 12781370 | PRKAG3 | Bos taurus protein kinase, AMP-activated, gamma 3 non-catalytic subunit, transcript variant 1 | metabolism | 10.979 | 12.938 | 5.665 | 141 | 104 | 87 | 100 | 635 | 726 | 473 | 65 | 58 | 44 | 43 | 53 | 45 |
| 12718222 | PTI | Bos taurus pancreatic trypsin inhibitor AKA Serpin Peptidase Inhibitor, Clade B (Ovalbumin), Member 6 | metabolism | 11.342 | 9.021 | 7.632 | 28 | 18 | 62 | 20 | 103 | 603 | 161 | 19 | 21 | 17 | 17 | 31 | 26 |
| 12775207 | PLA2G2D3 | Phospholipase A2, Group IID | metabolism | 13.505 | 10.37 | 16.139 | 15 | 28 | 263 | 24 | 1112 | 307 | 126 | 18 | 27 | 35 | 17 | 292 | 67 |
| 12825443 | ENPP6 | Ectonucleotide Pyrophosphatase/Phosphodiesterase 6 | metabolism | 13.97 | 4.006 | 24.411 | 22 | 16 | 13 | 17 | 366 | 492 | 379 | 53 | 24 | 19 | 100 | 131 | 82 |
| 12893495 | HPGD | Bos taurus hydroxyprostaglandin dehydrogenase 15-(NAD) | metabolism | 24.603 | 12.147 | 22.085 | 21 | 22 | 29 | 36 | 835 | 620 | 378 | 26 | 23 | 22 | 54 | 49 | 41 |
| 12731703 | FXYD6 | Bos taurus FXYD domain containing ion transport regulator 6 | molecular transport | 2.382 | 3.846 | 2.873 | 273 | 318 | 269 | 256 | 1008 | 933 | 543 | 346 | 415 | 262 | 319 | 204 | 138 |
| 12739086 | SLC30A10 | Bos taurus solute carrier family 30, member 10 | molecular transport | 2.944 | 2.988 | 2.307 | 52 | 33 | 57 | 59 | 132 | 77 | 143 | 38 | 37 | 40 | 64 | 24 | 35 |
| 12727223 | FOLR2 | Bos taurus folate receptor 2 (fetal) | molecular transport | 4.218 | 3.207 | 2.912 | 99 | 76 | 318 | 80 | 453 | 347 | 256 | 71 | 91 | 83 | 73 | 167 | 100 |
| 12819589 | SFXN2 | Bos taurus sideroflexin 2 | molecular transport | 4.313 | 2.735 | 2.793 | 120 | 128 | 155 | 125 | 314 | 420 | 374 | 97 | 78 | 81 | 121 | 135 | 147 |
| 12787010 | SLC1A3 | Solute Carrier Family 1 (Glial High Affinity Glutamate Transporter), Member 3 | molecular transport | 8.284 | 9.032 | 7.521 | 41 | 38 | 55 | 33 | 318 | 423 | 215 | 34 | 44 | 34 | 36 | 33 | 33 |
| 12717468 | RPS13 | Ribosomal Protein S13 | organelle structure | 2.712 | 2.201 | 2.139 | 527 | 503 | 354 | 594 | 1091 | 1261 | 815 | 420 | 372 | 360 | 483 | 509 | 427 |
| 12896730 | RPS12 | Bos taurus ribosomal protein S12 | organelle structure | 2.802 | 2.378 | 2.07 | 467 | 555 | 363 | 684 | 1157 | 1040 | 942 | 399 | 372 | 347 | 422 | 458 | 436 |
| 12705845 | RPL35 | Bos taurus ribosomal protein L35 | organelle structure | 3.206 | 3 | 2.421 | 93 | 137 | 99 | 132 | 369 | 250 | 225 | 102 | 77 | 80 | 84 | 103 | 89 |
| 12794373 | RPSA | Ribosomal Protein SA | organelle structure | 3.259 | 2.433 | 2.24 | 943 | 881 | 694 | 1104 | 1988 | 2045 | 1971 | 660 | 556 | 630 | 791 | 977 | 719 |
| 12910359 | RPL39 | Bos taurus ribosomal protein L39 | organelle structure | 3.282 | 2.361 | 2.003 | 1079 | 947 | 970 | 1451 | 2481 | 2209 | 1925 | 652 | 729 | 628 | 998 | 1025 | 783 |
| 12786649 | RPL37 | Ribosomal Protein L37 | organelle structure | 3.545 | 2.788 | 2.942 | 501 | 479 | 447 | 750 | 1896 | 1435 | 1413 | 417 | 472 | 438 | 536 | 616 | 537 |
| 12720857 | RPS21 | Ribosomal Protein S21 | organelle structure | 3.83 | 3.348 | 2.179 | 427 | 416 | 324 | 468 | 1135 | 842 | 720 | 250 | 226 | 217 | 248 | 290 | 255 |
| 12899167 | RPL37 | Bos taurus ribosomal protein L37 | organelle structure | 3.838 | 3.162 | 2.794 | 405 | 465 | 375 | 639 | 1506 | 1166 | 1217 | 350 | 347 | 311 | 390 | 434 | 399 |
| 12750150 | RPS5 | Bos taurus ribosomal protein S5 | organelle structure | 7.302 | 5.054 | 3.329 | 651 | 627 | 480 | 735 | 1943 | 2223 | 1996 | 299 | 283 | 262 | 346 | 436 | 443 |
| 12687880 | BRB | Bos taurus brain ribonuclease | posttranscriptional modification | 30.549 | 4.534 | 25.066 | 33 | 39 | 396 | 17 | 625 | 816 | 670 | 12 | 32 | 30 | 11 | 252 | 94 |
| 12720317 | SULF2 | Bos taurus sulfatase 2 | posttranslational modification | 2.393 | 6.55 | 9.922 | 42 | 49 | 45 | 45 | 398 | 618 | 368 | 187 | 208 | 169 | 63 | 78 | 65 |
| 12694663 | FBXL22 | Bos taurus F-box and leucine-rich repeat protein 22 | posttranslational modification | 3.017 | 4.937 | 5.355 | 50 | 41 | 40 | 38 | 181 | 297 | 212 | 70 | 87 | 68 | 44 | 46 | 47 |
| 12728718 | DTX4 | Deltex 4, E3 Ubiquitin Ligase | posttranslational modification | 3.148 | 2.323 | 3.722 | 43 | 36 | 35 | 29 | 141 | 138 | 121 | 39 | 41 | 47 | 53 | 70 | 51 |
| 12830479 | METTL12 | Bos taurus methyltransferase like 12, nuclear gene encoding mitochondrial protein | posttranslational modification | 3.677 | 3.121 | 2.69 | 55 | 53 | 61 | 80 | 211 | 286 | 75 | 45 | 53 | 38 | 41 | 66 | 54 |
| 12764558 | FKBP10 | Bos taurus FK506 binding protein 10, 65 kDa | posttranslational modification | 3.797 | 2.777 | 3.893 | 123 | 127 | 142 | 198 | 597 | 551 | 547 | 188 | 140 | 125 | 207 | 231 | 175 |
| 12870876 | PRSS12 | Bos taurus protease, serine, 12 (neurotrypsin, motopsin) | posttranslational modification | 7.118 | 6.2 | 7.035 | 19 | 23 | 14 | 20 | 185 | 121 | 101 | 19 | 19 | 17 | 20 | 24 | 20 |
| 12889437 | ADAMDEC1 | Bos taurus ADAM-like, decysin 1 | posttranslational modification | 54.162 | 30.704 | 45.446 | 16 | 17 | 14 | 33 | 627 | 1303 | 807 | 30 | 11 | 12 | 12 | 39 | 47 |
| 12903267 | SSR4 | Bos taurus signal sequence receptor, delta | protein trafficking | 4.395 | 3.951 | 2.062 | 1853 | 1723 | 1488 | 1475 | 3247 | 3552 | 3274 | 890 | 621 | 804 | 847 | 904 | 800 |
| 12836660 | OLFML3 | Bos taurus olfactomedin-like 3 | protein-protein binding | 2.723 | 2.904 | 5.685 | 46 | 34 | 30 | 24 | 154 | 240 | 171 | 65 | 66 | 72 | 68 | 64 | 60 |
| 12906096 | ASB9 | Bos taurus ankyrin repeat and SOCS box containing 9 | protein-protein binding | 2.946 | 2.147 | 2.086 | 81 | 57 | 98 | 45 | 148 | 110 | 167 | 37 | 60 | 48 | 74 | 64 | 58 |
| 12705085 | FHL2 | Bos taurus four and a half LIM domains 2 | protein-protein binding | 2.979 | 3.172 | 3.437 | 86 | 73 | 51 | 78 | 240 | 313 | 192 | 75 | 89 | 82 | 74 | 79 | 77 |
| 12905178 | DRP2 | Bos taurus dystrophin related protein 2 | protein-protein binding | 6.131 | 4.743 | 3.424 | 38 | 31 | 62 | 27 | 127 | 130 | 127 | 22 | 20 | 20 | 28 | 30 | 23 |
| 12855131 | MPP6 | Bos taurus membrane protein, palmitoylated 6 (MAGUK p55 subfamily member 6) | protein-protein binding | 6.366 | 3.291 | 2.909 | 110 | 120 | 141 | 147 | 359 | 374 | 389 | 50 | 64 | 63 | 121 | 108 | 112 |
| 12738945 | TRAF5 | Bos taurus TNF receptor-associated factor 5 | protein-protein binding | 6.395 | 9.027 | 7.843 | 15 | 21 | 12 | 14 | 139 | 145 | 85 | 21 | 20 | 16 | 14 | 12 | 14 |
| 12745328 | EDNRA | Endothelin Receptor Type A | signaling | 2.135 | 6.161 | 2.164 | 118 | 115 | 91 | 111 | 216 | 292 | 201 | 103 | 113 | 112 | 43 | 29 | 44 |
| 12821633 | SORBS1 | Bos taurus sorbin and SH3 domain containing 1 | signaling | 2.291 | 4.758 | 2.74 | 100 | 106 | 115 | 77 | 288 | 400 | 171 | 98 | 130 | 129 | 48 | 64 | 60 |
| 12711207 | RASA3 | RAS P21 Protein Activator 3 | signaling | 2.32 | 3.862 | 6.17 | 52 | 56 | 69 | 35 | 348 | 365 | 254 | 143 | 154 | 117 | 78 | 100 | 72 |
| 12823127 | AFAP1L2 | Bos taurus actin filament associated protein 1-like 2 | signaling | 2.328 | 4.039 | 2.207 | 102 | 115 | 88 | 88 | 238 | 205 | 206 | 80 | 106 | 94 | 47 | 48 | 67 |
| 12726646 | CD3E | Bos taurus CD3e molecule, epsilon (CD3-TCR complex) | signaling | 2.361 | 2.18 | 2.27 | 52 | 52 | 59 | 52 | 139 | 133 | 97 | 56 | 54 | 44 | 61 | 51 | 55 |
| 12783078 | NBL1 | Neuroblastoma 1, DAN Family BMP Antagonist | signaling | 2.665 | 2.076 | 2.854 | 96 | 64 | 81 | 77 | 181 | 274 | 226 | 95 | 85 | 73 | 108 | 108 | 107 |
| 12871689 | SLIT2 | Bos taurus slit homolog 2 (Drosophila) | signaling | 2.898 | 2.218 | 29.16 | 22 | 16 | 12 | 18 | 453 | 542 | 451 | 160 | 210 | 136 | 203 | 198 | 252 |
| 12856116 | THSD7A | Bos taurus thrombospondin, type I, domain containing 7A | signaling | 3.014 | 4.945 | 2.935 | 43 | 56 | 68 | 82 | 233 | 186 | 129 | 57 | 60 | 59 | 41 | 33 | 34 |
| 12884534 | CSF1R | Bos taurus colony stimulating factor 1 receptor | signaling | 3.55 | 2.897 | 2.699 | 44 | 40 | 83 | 30 | 154 | 109 | 114 | 33 | 43 | 30 | 27 | 68 | 43 |
| 12746214 | ARHGAP10 | Bos taurus Rho GTPase activating protein 10 | signaling | 3.579 | 4.006 | 2.382 | 241 | 233 | 259 | 234 | 576 | 671 | 492 | 160 | 161 | 160 | 130 | 155 | 147 |
| 12889029 | OGN | Osteoglycin | signaling | 3.615 | 36.416 | 44.524 | 30 | 48 | 32 | 40 | 1193 | 2186 | 1689 | 439 | 245 | 866 | 56 | 38 | 42 |
| 12728764 | SCUBE2 | Signal Peptide, CUB Domain, EGF-Like 2 | signaling | 4.96 | 5.346 | 9.414 | 34 | 34 | 34 | 32 | 285 | 367 | 302 | 66 | 70 | 56 | 44 | 77 | 61 |
| 12679648 | RARRES1 | Bos taurus retinoic acid receptor responder (tazarotene induced) 1 | signaling | 5.35 | 2.868 | 50.57 | 16 | 15 | 50 | 18 | 1105 | 1388 | 850 | 230 | 230 | 161 | 464 | 339 | 351 |
| 12856231 | VIPR2 | Bos taurus vasoactive intestinal peptide receptor 2 | signaling | 6.804 | 6.9 | 7.113 | 35 | 27 | 32 | 32 | 182 | 285 | 214 | 39 | 22 | 41 | 30 | 47 | 24 |
| 12685559 | ARHGEF26 | Bos taurus Rho guanine nucleotide exchange factor (GEF) 26 | signaling | 7.022 | 7.292 | 2.816 | 38 | 52 | 50 | 88 | 115 | 200 | 155 | 19 | 33 | 17 | 14 | 25 | 26 |
| 12862788 | PPP1R1A | Protein Phosphatase 1, Regulatory (Inhibitor) Subunit 1A | signaling | 9.401 | 7.115 | 3.614 | 167 | 149 | 185 | 126 | 435 | 709 | 573 | 56 | 75 | 51 | 70 | 83 | 85 |
| 12871482 | QRFPR | Bos taurus pyroglutamylated RFamide peptide receptor | signaling | 9.569 | 26.018 | 4.635 | 396 | 495 | 82 | 100 | 1525 | 710 | 736 | 62 | 106 | 138 | 35 | 31 | 41 |
| 12876972 | INSL3 | Insulin-Like 3 (Leydig Cell) | signaling | 9.711 | 2.525 | 12.499 | 933 | 962 | 494 | 282 | 7386 | 7137 | 7781 | 836 | 920 | 582 | 1958 | 4972 | 2616 |
| 12710121 | ITGBL1 | Bos taurus integrin, beta-like 1 (with EGF-like repeat domains) | signaling | 14.773 | 15.995 | 14.285 | 33 | 29 | 37 | 33 | 641 | 397 | 414 | 41 | 28 | 28 | 32 | 32 | 25 |
| 12774390 | FRZB | Frizzled-Related Protein | signaling | 15.156 | 11.947 | 14.571 | 10 | 11 | 13 | 16 | 160 | 297 | 123 | 10 | 13 | 12 | 18 | 15 | 13 |
| 12870149 | PDGFRA | Bos taurus platelet-derived growth factor receptor, alpha polypeptide | signaling | 24.338 | 15.808 | 16.006 | 44 | 47 | 60 | 76 | 717 | 1207 | 809 | 33 | 32 | 46 | 45 | 61 | 65 |
| 12821429 | CYP17A1 | Bos taurus cytochrome P450, subfamily XVII | steroidogenesis | 11.595 | 129.714 | 37.024 | 99 | 116 | 178 | 56 | 4085 | 3329 | 4163 | 169 | 474 | 453 | 39 | 31 | 21 |
| 12728123 | SESN3 | Sestrin 3 | stress response | 3.598 | 3.639 | 2.479 | 123 | 159 | 180 | 213 | 413 | 441 | 379 | 112 | 128 | 104 | 103 | 134 | 104 |
| 12704320 | ID2 | Bos taurus inhibitor of DNA binding 2, dominant negative helix-loop-helix protein | transcription | -4.489 | -3.124 | -2.867 | 1706 | 1934 | 2155 | 1397 | 731 | 497 | 653 | 2917 | 2269 | 3243 | 2190 | 1876 | 1762 |
| 12890072 | POLR1E | Bos taurus polymerase (RNA) I polypeptide E, 53kDa | transcription | -3.692 | -2.718 | -2.033 | 686 | 666 | 1052 | 437 | 319 | 293 | 394 | 1217 | 1002 | 1525 | 1131 | 902 | 727 |
| 12825680 | POLR2A | Polymerase (RNA) II (DNA Directed) Polypeptide A, 220kDa | transcription | -2.237 | -2.137 | -2.208 | 2079 | 2022 | 2230 | 1725 | 854 | 853 | 1028 | 1938 | 2112 | 2047 | 1925 | 2028 | 1874 |
| 12708137 | TCF23 | Bos taurus transcription factor 23 | transcription | 2.063 | 3.049 | 3.162 | 52 | 54 | 38 | 36 | 134 | 153 | 133 | 77 | 58 | 69 | 45 | 36 | 58 |
| 12884070 | OLFM2 | Bos taurus olfactomedin 2 | transcription | 2.686 | 2.699 | 2.522 | 40 | 44 | 42 | 38 | 109 | 111 | 89 | 41 | 28 | 48 | 43 | 34 | 38 |
| 12765009 | MYOCD | PREDICTED: Bos taurus myocardin | transcription | 2.995 | 4.186 | 2.005 | 92 | 122 | 92 | 153 | 266 | 266 | 161 | 66 | 80 | 80 | 40 | 64 | 60 |
| 12831952 | PKNOX2 | Bos taurus PBX/knotted 1 homeobox 2 | transcription | 4.31 | 5.724 | 6.787 | 65 | 58 | 51 | 47 | 449 | 369 | 311 | 74 | 112 | 78 | 67 | 79 | 52 |
| 12897055 | TCF21 | Bos taurus transcription factor 21 | transcription | 4.696 | 6.134 | 3.934 | 75 | 66 | 56 | 58 | 163 | 272 | 351 | 59 | 42 | 60 | 36 | 51 | 37 |
| 12832705 | EEF1G | Bos taurus eukaryotic translation elongation factor 1 gamma | translation | 2.494 | 2.195 | 2.333 | 872 | 817 | 746 | 820 | 2371 | 1800 | 1597 | 794 | 712 | 776 | 882 | 914 | 799 |
| 12801086 | ENSBTAT00000007026 | cdna:known chromosome:UMD3.1:23:17255947:17269998:1 gene:ENSBTAG00000005339 | unknown | -8.826 | -2.448 | -3.11 | 678 | 739 | 708 | 422 | 177 | 192 | 236 | 1793 | 1278 | 2399 | 433 | 352 | 770 |
| 12793718 | TM6SF1 | Bos taurus transmembrane 6 superfamily member 1 | unknown | -2.094 | 2.405 | -2.085 | 931 | 844 | 657 | 598 | 412 | 379 | 292 | 917 | 452 | 1011 | 143 | 159 | 144 |
| 12702743 | MORN2 | MORN Repeat Containing 2 | unknown | -2.084 | -2.495 | -2.22 | 189 | 241 | 214 | 297 | 103 | 99 | 112 | 222 | 207 | 225 | 280 | 245 | 259 |
| 12685923 | SCHIP1 | Bos taurus schwannomin interacting protein 1 (SCHIP1), mRNA. | unknown | 2.048 | 2.971 | 2.912 | 46 | 63 | 57 | 78 | 141 | 235 | 158 | 91 | 83 | 81 | 56 | 60 | 59 |
| 12732659 | OLFML1 | Bos taurus olfactomedin-like 1 | unknown | 2.28 | 7.055 | 19.81 | 28 | 25 | 21 | 19 | 287 | 626 | 525 | 234 | 189 | 180 | 57 | 72 | 66 |
| 12902417 | XM_002700200 | PREDICTED: Bos taurus dystrophin-like (LOC100297621), partial mRNA. | unknown | 3.254 | 2.769 | 3.986 | 14 | 25 | 23 | 70 | 122 | 178 | 59 | 39 | 29 | 34 | 42 | 45 | 32 |
| 12793818 | ISLR2 | PREDICTED: Bos taurus immunoglobulin superfamily containing leucine-rich repeat 2 | unknown | 3.774 | 3.332 | 4.665 | 38 | 34 | 28 | 28 | 187 | 181 | 93 | 43 | 35 | 39 | 37 | 57 | 40 |
| 12905207 | XM_002700245 | PREDICTED: Bos taurus dystrophin-like (LOC537655), partial mRNA. | unknown | 4.224 | 2.799 | 3.693 | 60 | 69 | 55 | 161 | 269 | 488 | 182 | 70 | 57 | 80 | 66 | 167 | 99 |
| 12791556 | ENSBTAT00000063737 | cdna:known chromosome:UMD3.1:21:69533538:69534140:-1 gene:ENSBTAG00000047319 | unknown | 4.499 | 2.364 | 2.968 | 109 | 97 | 105 | 103 | 366 | 323 | 244 | 79 | 57 | 71 | 127 | 170 | 101 |
| 12729400 | NRIP3 | Bos taurus nuclear receptor interacting protein 3 | unknown | 4.981 | 6.218 | 8.092 | 46 | 45 | 59 | 38 | 278 | 386 | 490 | 78 | 67 | 81 | 48 | 59 | 77 |
| 12725493 | SBSPON | Somatomedin B And Thrombospondin, Type 1 Domain Containing | unknown | 5.126 | 6.286 | 7.792 | 36 | 28 | 36 | 37 | 195 | 457 | 209 | 52 | 48 | 56 | 36 | 52 | 40 |
| 12728761 | TSKU | Bos taurus tsukushi small leucine rich proteoglycan homolog (Xenopus laevis) | unknown | 6.378 | 5.166 | 5.496 | 123 | 123 | 116 | 108 | 891 | 523 | 576 | 101 | 102 | 101 | 148 | 104 | 126 |
| 12723845 | NKAIN3 | PREDICTED: Bos taurus Na+/K+ transporting ATPase interacting 3 | unknown | 6.878 | 2.65 | 3.787 | 62 | 57 | 85 | 42 | 256 | 303 | 146 | 45 | 23 | 34 | 95 | 62 | 105 |
| 12847645 | OTOS | Otospiralin | unknown | 9.676 | 2.545 | 77.339 | 38 | 50 | 40 | 45 | 3314 | 3104 | 3596 | 460 | 260 | 341 | 1130 | 1355 | 1466 |
| 12829082 | H19 | Bos taurus H19, imprinted maternally expressed transcript, non-coding RNA. | untranslated RNA | 2.027 | 4.705 | 27.714 | 108 | 112 | 99 | 78 | 2355 | 3363 | 2551 | 1724 | 1174 | 1198 | 551 | 576 | 611 |
| 12831806 | SYTL2 | Synaptotagmin-Like 2 | vesicle transport | 3.335 | 3.489 | 10.437 | 41 | 32 | 25 | 28 | 270 | 427 | 291 | 94 | 113 | 85 | 62 | 147 | 86 |
| 12831416 | RAB38 | RAB38, Member RAS Oncogene Family | vesicle transport | 4.804 | 6.435 | 3.836 | 28 | 22 | 59 | 357 | 160 | 114 | 114 | 34 | 26 | 21 | 19 | 25 | 17 |
