# Supplementary material for: Transcriptomes of bovine ovarian follicular and luteal cells
Source: Data Brief. 2016 Dec 10;10:335–9. doi: 10.1016/j.dib.2016.11.093 (PMC5157705; doi:10.1016/j.dib.2016.11.093)
Supplement: Supplementary file 4 — Supplementary material [file mmc4.docx]

| **Table 3. Genes Enriched (≥ 2-fold greater expression than all other cells types) or Decreased (≤ -2-fold) in Large Luteal Cells** | | | | |  |  | **Linear Microarray Results (arbitrary units)** | | | | | | | | | | | | |
| --- | --- | --- | --- | --- | --- | --- | --- | --- | --- | --- | --- | --- | --- | --- | --- | --- | --- | --- | --- |
| **Probeset ID** | **Gene Symbol** | **Description** | **Functional Category** | **Fold Change (LLC vs GC)** | **Fold Change (LLC vs TC)** | **Fold Change (LLC vs SLC)** | **GC1** | **GC2** | **GC3** | **GC4** | **TC1** | **TC2** | **TC3** | **LLC1** | **LLC2** | **LLC3** | **SLC1** | **SLC2** | **SLC3** |
| 12884529 | PCDH1 | protocadherin 1 | adhesion | 2.811 | 2.091 | 2.307 | 47 | 51 | 52 | 48 | 57 | 74 | 71 | 174 | 107 | 145 | 63 | 69 | 51 |
| 12697589 | GLDN | PREDICTED: gliomedin | adhesion | 2.924 | 6 | 3.553 | 687 | 846 | 1057 | 640 | 421 | 267 | 512 | 2721 | 1855 | 2462 | 661 | 419 | 999 |
| 12749523 | CDH1 | Cadherin 1, Type 1, E-Cadherin (Epithelial) | adhesion | 3.741 | 4.887 | 2.196 | 30 | 31 | 45 | 24 | 25 | 24 | 24 | 134 | 86 | 142 | 37 | 57 | 75 |
| 12701179 | SDC1 | syndecan 1 | adhesion | 4.421 | 2.411 | 2.039 | 44 | 30 | 29 | 24 | 55 | 72 | 45 | 134 | 119 | 157 | 84 | 45 | 79 |
| 12835846 | F11R | F11 receptor | adhesion | 4.528 | 2.896 | 2.533 | 158 | 184 | 277 | 152 | 226 | 373 | 298 | 846 | 851 | 847 | 314 | 339 | 352 |
| 12858363 | ITGA7 | Integrin, Alpha 7 | adhesion | 4.856 | 3.392 | 2.833 | 28 | 23 | 27 | 27 | 34 | 41 | 38 | 112 | 132 | 137 | 54 | 50 | 33 |
| 12711726 | PCDH17 | protocadherin 17 | adhesion | 6.591 | 3.281 | 2.298 | 32 | 26 | 30 | 26 | 47 | 63 | 62 | 207 | 204 | 154 | 94 | 80 | 71 |
| 12834992 | ESAM | endothelial cell adhesion molecule (ESAM), mRNA. | adhesion | 6.688 | 3.153 | 2.665 | 33 | 43 | 37 | 40 | 69 | 89 | 88 | 278 | 263 | 231 | 103 | 115 | 75 |
| 12748152 | CLDN5 | claudin 5 | adhesion | 6.926 | 3.971 | 2.222 | 49 | 36 | 41 | 38 | 74 | 75 | 64 | 272 | 269 | 307 | 106 | 199 | 97 |
| 12867984 | TENC1 | PREDICTED: tensin like C1 domain containing phosphatase (tensin 2) | adhesion | 7.652 | 2.148 | 2.017 | 114 | 128 | 121 | 63 | 348 | 443 | 317 | 844 | 777 | 739 | 392 | 444 | 340 |
| 12752457 | PVR | PREDICTED: poliovirus receptor | adhesion | 7.917 | 4.03 | 2.336 | 70 | 53 | 81 | 57 | 114 | 133 | 131 | 495 | 506 | 523 | 210 | 213 | 230 |
| 12846830 | GJA4 | Gap Junction Protein, Alpha 4, 37kDa | adhesion | 9.134 | 4.359 | 2.702 | 54 | 59 | 83 | 60 | 105 | 149 | 149 | 781 | 502 | 493 | 218 | 223 | 202 |
| 12735818 | TNN | tenascin N | adhesion | 9.185 | 8.786 | 4.31 | 21 | 20 | 21 | 20 | 19 | 22 | 23 | 190 | 213 | 158 | 62 | 41 | 32 |
| 12734422 | CD34 | CD34 molecule | adhesion | 10.697 | 2.434 | 2.418 | 40 | 34 | 32 | 25 | 99 | 179 | 164 | 376 | 395 | 283 | 137 | 190 | 114 |
| 12871741 | LIMCH1 | LIM and calponin homology domains 1 | adhesion | 12.311 | 3.221 | 2.979 | 38 | 28 | 30 | 26 | 88 | 156 | 111 | 377 | 331 | 408 | 129 | 125 | 119 |
| 12792206 | CLEC14A | C-type lectin domain family 14, member A | adhesion | 13.819 | 3.776 | 2.706 | 18 | 13 | 16 | 11 | 50 | 57 | 48 | 199 | 184 | 201 | 81 | 85 | 54 |
| 12814004 | TMEM204 | transmembrane protein 204 | adhesion | 15.448 | 3.484 | 2.502 | 72 | 54 | 72 | 72 | 259 | 353 | 288 | 1258 | 1051 | 841 | 474 | 506 | 296 |
| 12720703 | CD93 | CD93 Molecule (ENSBTAT00000005511) | adhesion | 16.21 | 4.758 | 3.139 | 28 | 28 | 25 | 24 | 61 | 125 | 92 | 443 | 371 | 461 | 165 | 154 | 96 |
| 12871222 | NDNF | neuron-derived neurotrophic factor | adhesion | 16.477 | 10.568 | 6.334 | 51 | 37 | 57 | 54 | 88 | 68 | 75 | 944 | 688 | 821 | 118 | 120 | 149 |
| 12855916 | PODXL | Podocalyxin-Like | adhesion | 24.389 | 7.13 | 3.398 | 45 | 30 | 32 | 28 | 80 | 145 | 128 | 846 | 869 | 733 | 211 | 304 | 214 |
| 12767437 | PECAM1 | platelet/endothelial cell adhesion molecule | adhesion | 34.489 | 3.927 | 3.201 | 31 | 27 | 20 | 17 | 145 | 280 | 208 | 912 | 798 | 707 | 251 | 305 | 205 |
| 12869336 | EMP1 | PREDICTED: epithelial membrane protein 1, transcript variant 1 | adhesion | 41.151 | 11.004 | 2.034 | 26 | 23 | 43 | 31 | 76 | 148 | 121 | 977 | 1223 | 1522 | 653 | 573 | 577 |
| 12749712 | CDH5 | Cadherin 5, Type 2 (Vascular Endothelium) | adhesion | 43.501 | 4.864 | 2.973 | 31 | 33 | 27 | 24 | 152 | 410 | 265 | 1319 | 1279 | 1127 | 396 | 560 | 326 |
| 12738559 | SELP | Selectin P (Granule Membrane Protein 140kDa, Antigen CD62) | adhesion | 44.533 | 18.997 | 4.818 | 26 | 21 | 27 | 17 | 37 | 65 | 60 | 933 | 1015 | 1043 | 210 | 189 | 223 |
| 12840667 | GJA5 | gap junction protein, alpha 5, 40kDa | adhesion | 79.836 | 10.473 | 8.475 | 21 | 18 | 33 | 36 | 124 | 266 | 235 | 2503 | 1796 | 1972 | 237 | 222 | 277 |
| 12872493 | EMCN | Endomucin | adhesion | 89.804 | 3.745 | 2.618 | 11 | 18 | 12 | 16 | 130 | 559 | 523 | 1345 | 1214 | 1219 | 463 | 606 | 395 |
| 12691569 | LGALS3 | lectin, galactoside-binding, soluble, 3 | carbohydrate-protein binding | 6.197 | 5.325 | 2.201 | 24 | 22 | 27 | 25 | 35 | 25 | 26 | 152 | 109 | 202 | 59 | 75 | 71 |
| 12686973 | TFF2 | trefoil factor 2 | carbohydrate-protein binding | 10.769 | 7.12 | 8.826 | 38 | 43 | 44 | 34 | 49 | 87 | 50 | 210 | 474 | 779 | 44 | 49 | 52 |
| 12678862 | TERC | telomerase RNA component | chromosome structure | -3.077 | -3.756 | -2.358 | 844 | 689 | 552 | 428 | 1016 | 657 | 615 | 167 | 205 | 226 | 401 | 554 | 457 |
| 12851829 | AMPH | amphiphysin | cytoskeletal dynamics | 2.337 | 3.855 | 3.224 | 45 | 42 | 40 | 49 | 25 | 25 | 29 | 110 | 90 | 108 | 28 | 35 | 32 |
| 12880668 | CNN1 | calponin 1, basic, smooth muscle | cytoskeletal dynamics | 2.819 | -3.425 | -3.086 | 74 | 66 | 48 | 46 | 569 | 698 | 424 | 131 | 191 | 168 | 271 | 924 | 493 |
| 12900196 | FILIP1 | filamin A interacting protein 1 | cytoskeletal dynamics | 2.983 | 3.389 | 2.209 | 85 | 87 | 130 | 113 | 80 | 96 | 94 | 300 | 250 | 377 | 115 | 123 | 186 |
| 12861889 | FMNL3 | formin-like 3 | cytoskeletal dynamics | 3.943 | 2.404 | 2.077 | 52 | 43 | 34 | 43 | 59 | 75 | 76 | 147 | 191 | 167 | 83 | 72 | 87 |
| 12689890 | MYO1E | Myosin IE | cytoskeletal dynamics | 5.375 | 2.059 | 2.773 | 39 | 41 | 62 | 47 | 110 | 141 | 114 | 255 | 252 | 240 | 87 | 96 | 86 |
| 12849825 | ELMO1 | engulfment and cell motility 1, transcript variant 1 | cytoskeletal dynamics | 6.743 | 4.206 | 3.047 | 38 | 40 | 44 | 23 | 52 | 64 | 55 | 218 | 245 | 252 | 68 | 97 | 72 |
| 12822288 | ACTA2 | actin, alpha 2, smooth muscle, aorta | cytoskeletal dynamics | 9.49 | -2.32 | 4.267 | 241 | 188 | 151 | 123 | 3484 | 5113 | 2968 | 1332 | 1513 | 2101 | 314 | 497 | 349 |
| 12884951 | ECSCR | endothelial cell-specific chemotaxis regulator, transcript variant 1 | cytoskeletal dynamics | 10.859 | 2.656 | 2.812 | 29 | 26 | 28 | 29 | 70 | 176 | 122 | 290 | 351 | 275 | 100 | 131 | 96 |
| 12759184 | TPPP3 | tubulin polymerization-promoting protein family member 3 | cytoskeletal dynamics | 13.921 | 8.635 | 2.272 | 49 | 65 | 57 | 37 | 79 | 121 | 58 | 715 | 578 | 867 | 490 | 226 | 276 |
| 12702928 | AIF1L | allograft inflammatory factor 1-like | cytoskeletal dynamics | 22.714 | 19.833 | 3.713 | 85 | 99 | 101 | 61 | 88 | 90 | 115 | 2022 | 1522 | 2312 | 772 | 413 | 436 |
| 12699608 | SMC6 | PREDICTED: structural maintenance of chromosomes 6 | DNA repair | -3.514 | -2.24 | -2.026 | 1291 | 1367 | 1178 | 1247 | 833 | 816 | 778 | 389 | 383 | 316 | 671 | 916 | 637 |
| 12853913 | EEPD1 | endonuclease/exonuclease/phosphatase family domain containing 1 | DNA repair | 2.017 | 4.42 | 3.368 | 175 | 199 | 144 | 214 | 95 | 82 | 72 | 420 | 267 | 431 | 113 | 83 | 135 |
| 12853718 | SNX10 | sorting nexin 10 | endocytosis | 3.463 | 3.952 | 2.476 | 45 | 35 | 50 | 68 | 49 | 31 | 49 | 165 | 142 | 199 | 52 | 68 | 85 |
| 12705093 | EHD3 | EH-domain containing 3 | endocytosis | 7.133 | 3.136 | 2.828 | 39 | 66 | 48 | 42 | 88 | 137 | 107 | 363 | 303 | 364 | 105 | 154 | 109 |
| 12693873 | EHD4 | EH-domain containing 4 | endocytosis | 21.567 | 5.955 | 2.291 | 52 | 30 | 53 | 38 | 122 | 160 | 183 | 978 | 878 | 880 | 382 | 419 | 393 |
| 12859527 | FBLN1 | fibulin 1 | extracellular matrix | 4.215 | 3.579 | 2.403 | 345 | 342 | 429 | 114 | 219 | 488 | 320 | 1254 | 911 | 1376 | 449 | 383 | 660 |
| 12763562 | MMP28 | Matrix Metallopeptidase 28 | extracellular matrix | 8.027 | 2.337 | 2.79 | 14 | 23 | 24 | 17 | 61 | 76 | 60 | 150 | 170 | 138 | 52 | 61 | 52 |
| 12828038 | MMRN2 | PREDICTED: multimerin 2 | extracellular matrix | 8.922 | 3.33 | 3.011 | 56 | 55 | 48 | 50 | 99 | 158 | 174 | 422 | 489 | 488 | 163 | 172 | 131 |
| 12876394 | SPINK9 | PREDICTED: serine peptidase inhibitor, Kazal type 9 | extracellular matrix | 9.64 | 8.776 | 6.338 | 19 | 20 | 19 | 18 | 20 | 23 | 20 | 217 | 94 | 319 | 31 | 33 | 25 |
| 12887830 | ADAMTSL5 | ADAMTS-Like 5 | extracellular matrix | 9.96 | 4.379 | 4.042 | 33 | 29 | 31 | 26 | 55 | 91 | 62 | 277 | 314 | 300 | 59 | 90 | 74 |
| 12825859 | PLAU | plasminogen activator, urokinase | extracellular matrix | 10.19 | 5.895 | 2.611 | 44 | 33 | 43 | 46 | 58 | 68 | 91 | 454 | 410 | 394 | 185 | 187 | 119 |
| 12836080 | ADAMTS4 | ADAM metallopeptidase with thrombospondin type 1 motif, 4 | extracellular matrix | 14.083 | 11.526 | 2.273 | 87 | 69 | 70 | 35 | 70 | 62 | 99 | 850 | 782 | 995 | 345 | 389 | 419 |
| 12681808 | COL8A1 | collagen, type VIII, alpha 1 | extracellular matrix | 14.804 | 4.205 | 13.079 | 28 | 28 | 33 | 28 | 142 | 89 | 86 | 573 | 286 | 490 | 39 | 32 | 28 |
| 12894406 | ADAMTSL1 | PREDICTED: ADAMTS-like 1 | extracellular matrix | 24.586 | 15.779 | 12.624 | 30 | 25 | 19 | 19 | 30 | 38 | 40 | 568 | 441 | 718 | 38 | 62 | 38 |
| 12680034 | ADAMTS1 | ADAM metallopeptidase with thrombospondin type 1 motif, 1 | extracellular matrix | 39.93 | 4.218 | 3.331 | 33 | 26 | 33 | 34 | 310 | 283 | 296 | 1301 | 1244 | 1206 | 382 | 395 | 349 |
| 12741910 | FGG | fibrinogen gamma chain | extracellular matrix | 293.629 | 337.823 | 2.47 | 10 | 10 | 8 | 10 | 7 | 10 | 8 | 3275 | 2044 | 3408 | 1154 | 1136 | 1154 |
| 12893064 | IL33 | interleukin 33 | immune response | 3.433 | 3.471 | 3.988 | 42 | 61 | 94 | 73 | 41 | 107 | 60 | 215 | 194 | 265 | 44 | 71 | 55 |
| 12765990 | CCL1 | PREDICTED: chemokine (C-C motif) ligand 1 | immune response | 3.45 | 2.891 | 2.798 | 39 | 34 | 41 | 38 | 44 | 48 | 42 | 113 | 130 | 148 | 44 | 48 | 47 |
| 12765527 | CCL16 | PREDICTED: chemokine (C-C motif) ligand 16 | immune response | 5.156 | 3.31 | 2.09 | 32 | 32 | 60 | 36 | 39 | 78 | 74 | 204 | 245 | 161 | 95 | 113 | 83 |
| 12733526 | LRRC32 | leucine rich repeat containing 32 | immune response | 7.35 | 3.868 | 2.495 | 29 | 21 | 28 | 21 | 41 | 54 | 46 | 191 | 204 | 152 | 65 | 85 | 69 |
| 12765521 | CCL14 | Chemokine (C-C Motif) Ligand 14 | immune response | 11.767 | 3.822 | 3.01 | 20 | 11 | 22 | 19 | 31 | 89 | 55 | 213 | 184 | 214 | 55 | 103 | 54 |
| 12751936 | CX3CL1 | Chemokine (C-X3-C Motif) Ligand 1 | immune response | 12.696 | 5.513 | 3.148 | 11 | 16 | 13 | 12 | 24 | 34 | 31 | 180 | 151 | 158 | 58 | 52 | 45 |
| 12767757 | CCL11 | chemokine (C-C motif) ligand 11 | immune response | 13.737 | 14.223 | 7.698 | 17 | 10 | 16 | 10 | 10 | 12 | 17 | 181 | 116 | 271 | 29 | 19 | 23 |
| 12766769 | ICAM2 | PREDICTED: intercellular adhesion molecule 2-like, transcript variant 1 (LOC506088) | immune response | 14.594 | 5.437 | 2.575 | 29 | 13 | 15 | 25 | 45 | 56 | 57 | 293 | 279 | 283 | 80 | 173 | 98 |
| 12869909 | CXCL2 | Chemokine (C-X-C Motif) Ligand 2 | immune response | 19.556 | 17.897 | 2.942 | 10 | 10 | 6 | 8 | 9 | 8 | 11 | 157 | 167 | 171 | 60 | 48 | 62 |
| 12872284 | CXCL3 | chemokine (C-X-C motif) ligand 3 | immune response | 77.821 | 19.611 | 2.742 | 10 | 7 | 9 | 6 | 13 | 52 | 46 | 601 | 579 | 668 | 201 | 221 | 254 |
| 12858014 | SLC26A10 | solute carrier family 26, member 10 | ion transport | 3.405 | 2.424 | 2.267 | 35 | 33 | 43 | 35 | 44 | 53 | 55 | 97 | 151 | 127 | 49 | 63 | 52 |
| 12767314 | KCNJ2 | potassium inwardly-rectifying channel, subfamily J, member 2 | ion transport | 4.532 | 3.405 | 2.922 | 22 | 19 | 27 | 26 | 27 | 34 | 32 | 109 | 91 | 118 | 34 | 34 | 41 |
| 12767675 | P2RX5 | Purinergic Receptor P2X, Ligand Gated Ion Channel, 5 | ion transport | 6.105 | 4.228 | 3.175 | 22 | 22 | 26 | 18 | 28 | 37 | 31 | 162 | 103 | 143 | 46 | 42 | 39 |
| 12732540 | KCNE3 | PREDICTED: potassium voltage-gated channel, Isk-related family, member 3 | ion transport | 7.516 | 5.046 | 5.398 | 38 | 31 | 39 | 33 | 48 | 50 | 60 | 301 | 269 | 227 | 57 | 44 | 46 |
| 12831337 | ANO1 | anoctamin 1, calcium activated chloride channel | ion transport | 7.93 | 6.513 | 4.721 | 14 | 13 | 15 | 13 | 14 | 20 | 16 | 108 | 105 | 114 | 31 | 23 | 17 |
| 12839963 | KCNN3 | PREDICTED: potassium intermediate/small conductance calcium-activated channel, subfamily N, member 3, transcript variant 2 | ion transport | 11.418 | 10.99 | 2.506 | 16 | 13 | 12 | 12 | 14 | 15 | 11 | 123 | 171 | 159 | 83 | 56 | 46 |
| 12905032 | GRIA3 | glutamate receptor, ionotrophic, AMPA 3 | ion transport | 13.896 | 14.424 | 8.511 | 39 | 32 | 44 | 37 | 36 | 41 | 33 | 689 | 429 | 490 | 93 | 46 | 55 |
| 12689668 | PRKCH | protein kinase C, eta | kinase | 14.417 | 3.796 | 2.916 | 26 | 23 | 22 | 28 | 76 | 118 | 90 | 386 | 354 | 320 | 133 | 148 | 90 |
| 12833208 | PLA2G16 | phospholipase A2, group XVI | lipid metabolism | 2.139 | 2.51 | 2.196 | 215 | 190 | 269 | 224 | 203 | 157 | 215 | 514 | 386 | 547 | 232 | 198 | 224 |
| 12903047 | PGRMC1 | progesterone receptor membrane component 1 | lipid metabolism | 2.872 | 2.811 | 2.874 | 844 | 845 | 1106 | 967 | 940 | 1126 | 823 | 2945 | 2091 | 3143 | 940 | 871 | 995 |
| 12776426 | SERINC2 | Serine Incorporator 2 | lipid metabolism | 3.513 | 3.728 | 3.863 | 47 | 45 | 36 | 35 | 41 | 37 | 36 | 190 | 102 | 148 | 35 | 32 | 45 |
| 12713456 | SPTLC3 | PREDICTED: serine palmitoyltransferase, long chain base subunit 3 | lipid metabolism | 4.269 | 3.699 | 6.894 | 44 | 39 | 56 | 46 | 48 | 72 | 42 | 266 | 152 | 184 | 34 | 23 | 29 |
| 12725595 | CYP7B1 | cDNA clone IMAGE:8161594. | lipid metabolism | 5.548 | 2.966 | 2.281 | 23 | 16 | 25 | 24 | 35 | 38 | 49 | 117 | 104 | 142 | 48 | 45 | 68 |
| 12909544 | ACSL4 | acyl-CoA synthetase long-chain family member 4 | lipid metabolism | 5.551 | 6.51 | 2.098 | 315 | 355 | 244 | 261 | 242 | 218 | 288 | 1485 | 1233 | 2284 | 676 | 670 | 1000 |
| 12833840 | FADS3 | Fatty Acid Desaturase 3 | lipid metabolism | 6.546 | 2.305 | 3.696 | 55 | 50 | 45 | 34 | 135 | 137 | 115 | 279 | 263 | 353 | 108 | 58 | 81 |
| 12694667 | DHRS7 | dehydrogenase/reductase (SDR family) member 7 | lipid metabolism | 9.734 | 2.624 | 2.347 | 24 | 28 | 32 | 36 | 169 | 104 | 77 | 295 | 205 | 404 | 147 | 102 | 126 |
| 12867232 | FAR2 | fatty acyl CoA reductase 2 | lipid metabolism | 13.545 | 2.793 | 2.251 | 28 | 27 | 18 | 30 | 69 | 174 | 150 | 355 | 361 | 306 | 139 | 201 | 123 |
| 12809232 | ST8SIA5 | ST8 alpha-N-acetyl-neuraminide alpha-2,8-sialyltransferase 5 | metabolism | 2.595 | 6.258 | 3.666 | 122 | 131 | 92 | 103 | 44 | 50 | 43 | 343 | 213 | 325 | 120 | 50 | 81 |
| 12721150 | ABHD12 | Abhydrolase Domain Containing 12 | metabolism | 2.963 | 3.48 | 2.295 | 683 | 678 | 687 | 644 | 702 | 507 | 528 | 2655 | 1495 | 1998 | 1130 | 729 | 796 |
| 12893648 | FBP1 | fructose-1,6-bisphosphatase 1 | metabolism | 3.321 | 8.156 | 11.743 | 109 | 122 | 121 | 149 | 51 | 34 | 75 | 641 | 203 | 539 | 36 | 23 | 52 |
| 12699819 | RBKS | ribokinase | metabolism | 3.712 | 6.469 | 2.175 | 155 | 158 | 144 | 150 | 91 | 76 | 96 | 575 | 464 | 674 | 322 | 200 | 271 |
| 12721746 | RDH10 | retinol dehydrogenase 10 (all-trans) | metabolism | 3.993 | 2.397 | 2.297 | 255 | 243 | 217 | 317 | 355 | 487 | 448 | 1138 | 762 | 1230 | 671 | 297 | 441 |
| 12691859 | CA12 | PREDICTED: carbonic anhydrase XII | metabolism | 4.754 | 3.769 | 3.947 | 28 | 26 | 21 | 18 | 29 | 26 | 33 | 55 | 114 | 213 | 25 | 25 | 35 |
| 12853844 | PON3 | paraoxonase 3 | metabolism | 4.786 | 4.632 | 4.827 | 19 | 32 | 46 | 96 | 34 | 50 | 44 | 151 | 149 | 326 | 33 | 40 | 49 |
| 12761176 | ATP1B2 | ATPase, Na+/K+ Transporting, Beta 2 Polypeptide | metabolism | 5.457 | 2.981 | 2.009 | 29 | 32 | 31 | 25 | 67 | 46 | 49 | 203 | 142 | 139 | 88 | 65 | 86 |
| 12743757 | INPP4B | PREDICTED: inositol polyphosphate-4-phosphatase, type II, 105kDa, transcript variant 1 | metabolism | 5.827 | 4.095 | 3.737 | 30 | 41 | 39 | 36 | 44 | 60 | 52 | 188 | 264 | 192 | 55 | 63 | 52 |
| 12781150 | PID1 | phosphotyrosine interaction domain containing 1 | metabolism | 9.03 | 6.103 | 8.867 | 26 | 37 | 28 | 33 | 39 | 49 | 48 | 356 | 170 | 352 | 29 | 33 | 31 |
| 12797483 | RBMS3 | RNA Binding Motif, Single Stranded Interacting Protein 3 | metabolism | 9.678 | 2.334 | 2.039 | 28 | 46 | 61 | 27 | 119 | 202 | 167 | 393 | 343 | 379 | 162 | 195 | 190 |
| 12760653 | AOC3 | Amine Oxidase, Copper Containing 3 | metabolism | 10.497 | 2.58 | 3.042 | 41 | 24 | 20 | 23 | 87 | 175 | 77 | 285 | 259 | 275 | 87 | 116 | 72 |
| 12736792 | EPHX1 | epoxide hydrolase 1, microsomal (xenobiotic) | metabolism | 12.534 | 3.778 | 9.906 | 67 | 80 | 63 | 96 | 234 | 317 | 211 | 1333 | 638 | 994 | 131 | 100 | 66 |
| 12694790 | CDO1 | Cysteine Dioxygenase Type 1 | metabolism | 13.617 | 3.792 | 3.078 | 96 | 125 | 108 | 102 | 396 | 403 | 360 | 1295 | 1267 | 1911 | 338 | 351 | 907 |
| 12795679 | ACOX2 | acyl-CoA oxidase 2, branched chain | metabolism | 15.498 | 12.682 | 8.937 | 45 | 39 | 41 | 34 | 44 | 68 | 37 | 785 | 360 | 803 | 78 | 63 | 65 |
| 12798391 | ABHD6 | abhydrolase domain containing 6 | metabolism | 16.258 | 9.145 | 2.565 | 126 | 154 | 238 | 134 | 217 | 225 | 454 | 2503 | 2550 | 2649 | 938 | 615 | 1737 |
| 12692815 | RNASE3 | Ribonuclease, RNase A Family, 3 | metabolism | 18.59 | 15.402 | 11.659 | 10 | 13 | 11 | 9 | 13 | 11 | 15 | 147 | 81 | 625 | 16 | 21 | 15 |
| 12724157 | CA2 | carbonic anhydrase II | metabolism | 25.344 | 8.852 | 3.112 | 29 | 23 | 40 | 24 | 95 | 63 | 90 | 593 | 1011 | 627 | 228 | 195 | 280 |
| 12727180 | BBOX1 | butyrobetaine (gamma), 2-oxoglutarate dioxygenase (gamma-butyrobetaine hydroxylase) 1 | metabolism | 26.484 | 14.937 | 2.69 | 12 | 14 | 8 | 14 | 21 | 21 | 21 | 311 | 234 | 410 | 123 | 99 | 126 |
| 12697204 | RNASE4 | ribonuclease, RNase A family, 4 | metabolism | 117.489 | 18.543 | 6.217 | 25 | 18 | 61 | 38 | 130 | 265 | 247 | 3284 | 3126 | 5275 | 997 | 304 | 745 |
| 12778346 | CCNYL1 | Cyclin Y-Like 1 | mitosis | 3.464 | 3.073 | 2.322 | 112 | 136 | 113 | 125 | 123 | 158 | 130 | 373 | 437 | 452 | 191 | 171 | 180 |
| 12831125 | CCND1 | cyclin D1 | mitosis | 5.058 | 2.665 | 2.283 | 36 | 54 | 37 | 46 | 72 | 79 | 94 | 236 | 191 | 221 | 124 | 84 | 80 |
| 12836290 | CRABP2 | Cellular Retinoic Acid Binding Protein 2 | molecular transport | 2.76 | 10.11 | 4.618 | 176 | 171 | 120 | 105 | 41 | 29 | 45 | 405 | 273 | 517 | 136 | 65 | 65 |
| 12681711 | RBP1 | retinol binding protein 1, cellular | molecular transport | 3.088 | 2.89 | 2.418 | 112 | 91 | 93 | 146 | 151 | 102 | 101 | 334 | 236 | 478 | 198 | 108 | 124 |
| 12690897 | SLC7A8 | solute carrier family 7 (amino acid transporter light chain, L system), member 8 | molecular transport | 3.153 | 2.38 | 2.862 | 29 | 31 | 37 | 31 | 38 | 47 | 41 | 168 | 68 | 87 | 38 | 37 | 29 |
| 12730785 | APOA1 | apolipoprotein A-I | molecular transport | 3.538 | 2.172 | 2.604 | 174 | 186 | 244 | 166 | 348 | 241 | 354 | 780 | 434 | 901 | 297 | 231 | 251 |
| 12792419 | SLC25A21 | solute carrier family 25 (mitochondrial oxodicarboxylate carrier), member 21, nuclear gene encoding mitochondrial protein | molecular transport | 5.246 | 5.402 | 3.743 | 40 | 44 | 37 | 41 | 36 | 47 | 35 | 259 | 92 | 399 | 65 | 55 | 50 |
| 12864151 | SLC2A3 | solute carrier family 2 (facilitated glucose transporter), member 3 | molecular transport | 5.632 | 15.167 | 2.154 | 100 | 109 | 189 | 201 | 53 | 59 | 48 | 801 | 633 | 1025 | 326 | 471 | 338 |
| 12853145 | AQP1 | aquaporin 1 (Colton blood group) | molecular transport | 22.33 | 8.544 | 4.055 | 31 | 27 | 35 | 32 | 83 | 76 | 83 | 519 | 872 | 720 | 139 | 273 | 128 |
| 12810552 | ATP8B1 | ATPase, aminophospholipid transporter, class I, type 8B, member 1 | molecular transport | 23.046 | 10.334 | 2.451 | 16 | 14 | 15 | 20 | 32 | 43 | 33 | 345 | 370 | 396 | 137 | 132 | 190 |
| 12678807 | SLCO2A1 | solute carrier organic anion transporter family, member 2A1 | molecular transport | 52.954 | 4.354 | 2.184 | 37 | 47 | 45 | 35 | 352 | 748 | 462 | 2404 | 2476 | 1687 | 953 | 1391 | 727 |
| 12895201 | SCARA3 | PREDICTED: scavenger receptor class A, member 3 | oxidative stress | 5.547 | 5.209 | 3.035 | 101 | 89 | 69 | 49 | 81 | 85 | 72 | 478 | 405 | 360 | 192 | 135 | 96 |
| 12679876 | PDIA5 | protein disulfide isomerase family A, member 5 | posttranslational modification | -2.301 | -2.918 | -2.009 | 392 | 460 | 471 | 537 | 807 | 434 | 574 | 229 | 227 | 155 | 370 | 397 | 446 |
| 12908534 | HS6ST2 | heparan sulfate 6-O-sulfotransferase 2 | posttranslational modification | 2.168 | 6.606 | 8.287 | 221 | 244 | 195 | 167 | 90 | 45 | 74 | 517 | 322 | 524 | 67 | 41 | 55 |
| 12841901 | TRABD2B | PREDICTED: UPF0632 protein A (LOC787081); TraB Domain Containing 2B | posttranslational modification | 3.97 | 2.555 | 2.528 | 31 | 39 | 37 | 33 | 49 | 55 | 58 | 135 | 165 | 116 | 52 | 72 | 43 |
| 12838481 | RHBDL2 | rhomboid, veinlet-like 2 (Drosophila) | posttranslational modification | 4.094 | 4.273 | 2.329 | 58 | 56 | 84 | 28 | 35 | 57 | 64 | 200 | 251 | 203 | 112 | 70 | 103 |
| 12883575 | ST8SIA4 | ST8 alpha-N-acetyl-neuraminide alpha-2,8-sialyltransferase 4 | posttranslational modification | 9.783 | 2.963 | 2.515 | 13 | 16 | 23 | 19 | 51 | 63 | 57 | 205 | 189 | 124 | 74 | 84 | 48 |
| 12816646 | WBSCR22 | Williams Beuren Syndrome Chromosome Region 22 | posttranslational modification | 11.091 | 8.822 | 3.205 | 301 | 282 | 387 | 208 | 371 | 304 | 416 | 3440 | 2579 | 3639 | 913 | 692 | 1551 |
| 12762867 | ST6GALNAC2 | ST6 (alpha-N-acetyl-neuraminyl-2,3-beta-galactosyl-1, 3)-N-acetylgalactosaminide alpha-2,6-sialyltransferase 2 | posttranslational modification | 13.424 | 2.96 | 2.249 | 44 | 28 | 43 | 25 | 104 | 240 | 149 | 512 | 532 | 353 | 192 | 287 | 153 |
| 12782662 | HECW2 | HECT, C2 And WW Domain Containing E3 Ubiquitin Protein Ligase 2 | posttranslational modification | 14.079 | 5.058 | 3.208 | 18 | 16 | 11 | 16 | 32 | 60 | 40 | 248 | 211 | 191 | 95 | 57 | 56 |
| 12859291 | CPM | carboxypeptidase M | posttranslational modification | 16.307 | 9.044 | 8.18 | 24 | 20 | 35 | 14 | 25 | 49 | 51 | 532 | 218 | 394 | 64 | 39 | 33 |
| 12699761 | MALL | mal, T-cell differentiation protein-like | protein trafficking | 5.837 | 2.016 | 3.515 | 47 | 39 | 42 | 40 | 78 | 155 | 145 | 230 | 266 | 234 | 77 | 62 | 68 |
| 12728805 | USH1C | Usher Syndrome 1C (Autosomal Recessive, Severe) | protein-protein binding | 2.735 | 2.462 | 2.201 | 49 | 31 | 43 | 36 | 42 | 47 | 43 | 105 | 104 | 114 | 42 | 41 | 68 |
| 12729863 | UVRAG | UV radiation resistance associated gene | protein-protein binding | 3.461 | 4.133 | 3.268 | 179 | 186 | 236 | 224 | 172 | 168 | 175 | 779 | 439 | 1045 | 215 | 213 | 224 |
| 12690012 | WDR72 | PREDICTED: WD repeat domain 72 | protein-protein binding | 3.604 | 4.406 | 5.375 | 31 | 30 | 23 | 42 | 21 | 23 | 32 | 143 | 51 | 181 | 19 | 25 | 19 |
| 12778639 | CYTIP | cytohesin 1 interacting protein | protein-protein binding | 8.194 | 5.171 | 5.644 | 11 | 11 | 13 | 11 | 20 | 18 | 17 | 133 | 48 | 131 | 20 | 18 | 13 |
| 12856735 | VWF | von Willebrand factor | protein-protein binding | 8.194 | 3.108 | 2.576 | 50 | 31 | 40 | 26 | 76 | 110 | 99 | 278 | 321 | 278 | 114 | 115 | 111 |
| 12814467 | SLC9A3R2 | solute carrier family 9 (sodium/hydrogen exchanger), member 3 regulator 2 | protein-protein binding | 8.871 | 3.308 | 2.698 | 92 | 77 | 91 | 91 | 206 | 287 | 218 | 781 | 849 | 705 | 301 | 354 | 223 |
| 12755625 | HP | Haptoglobin | protein-protein binding | 10.029 | 10.876 | 8.838 | 10 | 12 | 11 | 10 | 11 | 10 | 9 | 96 | 73 | 169 | 10 | 11 | 16 |
| 12747823 | HSPB8 | heat shock 22kDa protein 8 | protein-protein binding | 15.244 | 4.069 | 2.658 | 29 | 23 | 28 | 27 | 112 | 105 | 87 | 470 | 465 | 314 | 161 | 184 | 123 |
| 12781035 | TINAGL1 | PREDICTED: tubulointerstitial nephritis antigen-like 1 | protein-protein binding | 16.065 | 4.138 | 2.582 | 62 | 40 | 42 | 36 | 136 | 200 | 181 | 790 | 649 | 680 | 283 | 315 | 228 |
| 12758651 | HSPB6 | heat shock protein, alpha-crystallin-related, B6 | protein-protein binding | 23.55 | 2.861 | 8.38 | 82 | 67 | 73 | 63 | 963 | 498 | 418 | 2132 | 1407 | 1566 | 194 | 237 | 173 |
| 12875858 | LDB2 | LIM domain binding 2 | protein-protein binding | 28.138 | 3.967 | 2.797 | 21 | 22 | 15 | 27 | 95 | 202 | 165 | 638 | 617 | 504 | 201 | 267 | 169 |
| 12709043 | POSTN | periostin, osteoblast specific factor | protein-protein binding | 28.773 | 4.91 | 12.708 | 59 | 67 | 80 | 15 | 164 | 313 | 393 | 2127 | 762 | 1474 | 307 | 40 | 94 |
| 12857108 | TIMP3 | TIMP Metallopeptidase Inhibitor 3 | protein-protein binding | 49.112 | 4.161 | 2.698 | 45 | 50 | 53 | 32 | 405 | 769 | 455 | 2320 | 2127 | 2070 | 856 | 1052 | 577 |
| 12775222 | GRB14 | growth factor receptor-bound protein 14 | signaling | -10.08 | -7.123 | -2.433 | 1581 | 1589 | 1650 | 1791 | 1211 | 1103 | 1187 | 163 | 170 | 159 | 320 | 460 | 430 |
| 12849057 | PRKAR2B | Protein Kinase, CAMP-Dependent, Regulatory, Type II, Beta | signaling | -8.066 | -5.307 | -2.165 | 1684 | 1861 | 1769 | 2140 | 1317 | 1246 | 1110 | 259 | 223 | 211 | 513 | 620 | 389 |
| 12844249 | PTPRF | protein tyrosine phosphatase, receptor type, F | signaling | -3.053 | -2.905 | -2.429 | 509 | 453 | 458 | 362 | 465 | 393 | 408 | 146 | 152 | 137 | 309 | 405 | 348 |
| 12706345 | SEMA4C | sema domain, immunoglobulin domain (Ig), transmembrane domain (TM) and short cytoplasmic domain, (semaphorin) 4C | signaling | 2.373 | 2.316 | 2.412 | 154 | 151 | 158 | 146 | 118 | 193 | 165 | 360 | 360 | 363 | 134 | 196 | 128 |
| 12774585 | IGFBP2 | insulin-like growth factor binding protein 2, 36kDa | signaling | 2.383 | 2.517 | 4.256 | 116 | 129 | 102 | 84 | 117 | 93 | 94 | 302 | 212 | 2084 | 59 | 55 | 65 |
| 12705950 | ADRA2B | adrenergic, alpha-2B-, receptor | signaling | 2.666 | 2.215 | 2.258 | 59 | 49 | 40 | 40 | 57 | 53 | 59 | 146 | 115 | 113 | 58 | 64 | 45 |
| 12689684 | DLL4 | delta-like 4 (Drosophila) | signaling | 2.781 | 5.615 | 3.396 | 159 | 155 | 530 | 125 | 85 | 96 | 122 | 569 | 543 | 567 | 159 | 195 | 144 |
| 12819852 | PLCE1 | phospholipase C, epsilon 1 | signaling | 2.808 | 4.252 | 2.204 | 70 | 73 | 98 | 51 | 46 | 44 | 51 | 207 | 218 | 178 | 99 | 64 | 118 |
| 12801086 | VEGFA | Vascular Endothelial Growth Factor A (Isoform 201) | signaling | 2.837 | 8.826 | 3.604 | 678 | 739 | 708 | 422 | 177 | 192 | 236 | 1793 | 1278 | 2399 | 433 | 352 | 770 |
| 12759735 | PRKD2 | Protein Kinase D2 | signaling | 2.894 | 2.931 | 2.238 | 78 | 88 | 68 | 77 | 74 | 78 | 78 | 222 | 263 | 194 | 102 | 129 | 76 |
| 12864062 | KITLG | KIT ligand | signaling | 3 | 2.549 | 4.858 | 145 | 160 | 134 | 181 | 171 | 208 | 168 | 534 | 363 | 509 | 98 | 89 | 98 |
| 12786119 | ARL15 | ADP-ribosylation factor-like 15 | signaling | 3.066 | 2.976 | 2.663 | 303 | 314 | 304 | 319 | 259 | 332 | 378 | 1051 | 825 | 991 | 368 | 386 | 320 |
| 12734639 | ADORA1 | adenosine A1 receptor | signaling | 3.078 | 2.763 | 2.908 | 55 | 50 | 46 | 48 | 52 | 60 | 54 | 170 | 105 | 200 | 51 | 43 | 66 |
| 12862620 | DUSP16 | PREDICTED: dual specificity phosphatase 16 | signaling | 3.206 | 3.375 | 3.081 | 159 | 169 | 192 | 224 | 208 | 173 | 149 | 553 | 455 | 820 | 174 | 244 | 166 |
| 12780277 | INPP1 | Inositol Polyphosphate-1-Phosphatase | signaling | 3.256 | 2.677 | 2.607 | 53 | 50 | 60 | 56 | 55 | 80 | 68 | 178 | 213 | 149 | 67 | 79 | 61 |
| 12762096 | RASD1 | RAS, dexamethasone-induced 1 | signaling | 3.32 | 2.874 | 2.257 | 111 | 89 | 91 | 82 | 99 | 116 | 107 | 324 | 263 | 342 | 165 | 138 | 111 |
| 12729539 | P2RY6 | pyrimidinergic receptor P2Y, G-protein coupled, 6 | signaling | 3.373 | 2.281 | 2.066 | 37 | 29 | 34 | 28 | 43 | 42 | 58 | 103 | 94 | 128 | 49 | 49 | 59 |
| 12811857 | PDGFA | Platelet-Derived Growth Factor Alpha Polypeptide | signaling | 3.379 | 2.619 | 2.747 | 63 | 53 | 66 | 44 | 71 | 81 | 65 | 189 | 187 | 189 | 68 | 66 | 72 |
| 12754460 | CMIP | PREDICTED: c-Maf inducing protein | signaling | 3.419 | 2.548 | 2.121 | 64 | 69 | 78 | 58 | 85 | 100 | 83 | 218 | 245 | 221 | 88 | 101 | 139 |
| 12901622 | DLL1 | PREDICTED: delta-like 1 (Drosophila) | signaling | 3.473 | 2.527 | 2.011 | 37 | 32 | 33 | 40 | 41 | 54 | 53 | 131 | 129 | 111 | 59 | 75 | 52 |
| 12705580 | RALGDS | ral guanine nucleotide dissociation stimulator | signaling | 3.497 | 3.539 | 2.059 | 79 | 69 | 79 | 59 | 74 | 67 | 70 | 263 | 261 | 221 | 126 | 118 | 117 |
| 12688966 | SMAD6 | SMAD family member 6 | signaling | 3.552 | 2.672 | 3.182 | 61 | 59 | 58 | 33 | 52 | 81 | 75 | 193 | 192 | 163 | 60 | 62 | 50 |
| 12728447 | UBASH3B | ubiquitin associated and SH3 domain containing B | signaling | 3.681 | 3.793 | 2.6 | 34 | 34 | 33 | 37 | 36 | 32 | 32 | 132 | 93 | 164 | 52 | 41 | 55 |
| 12861208 | RAPGEF3 | Rap guanine nucleotide exchange factor (GEF) 3 | signaling | 3.683 | 2.531 | 2.539 | 55 | 40 | 60 | 49 | 69 | 81 | 72 | 193 | 175 | 190 | 75 | 85 | 62 |
| 12713975 | PKIG | Protein Kinase (CAMP-Dependent, Catalytic) Inhibitor Gamma | signaling | 3.749 | 2.514 | 3.513 | 117 | 96 | 91 | 70 | 133 | 154 | 126 | 375 | 327 | 334 | 116 | 113 | 72 |
| 12692043 | MCC | mutated in colorectal cancers | signaling | 3.915 | 2.33 | 2.081 | 51 | 35 | 50 | 44 | 67 | 85 | 74 | 180 | 180 | 166 | 83 | 97 | 74 |
| 12880033 | ARHGEF37 | Rho guanine nucleotide exchange factor (GEF) 37 | signaling | 3.917 | 3.193 | 2.675 | 33 | 29 | 39 | 36 | 40 | 41 | 45 | 94 | 151 | 170 | 70 | 34 | 53 |
| 12890785 | RGS3 | Regulator Of G-Protein Signaling 3 | signaling | 3.95 | 2.636 | 2.026 | 67 | 63 | 48 | 50 | 76 | 98 | 81 | 184 | 254 | 238 | 112 | 138 | 86 |
| 12692661 | ADCY4 | adenylate cyclase 4 | signaling | 4.039 | 2.216 | 2.163 | 45 | 25 | 27 | 30 | 46 | 78 | 50 | 117 | 136 | 122 | 55 | 61 | 56 |
| 12733368 | WNT11 | wingless-type MMTV integration site family, member 11 | signaling | 4.181 | 3.945 | 4.075 | 53 | 36 | 42 | 44 | 48 | 45 | 45 | 198 | 120 | 252 | 53 | 43 | 38 |
| 12864745 | C1QTNF6 | C1q and tumor necrosis factor related protein 6 | signaling | 4.451 | 2.695 | 3.796 | 53 | 46 | 56 | 34 | 66 | 81 | 86 | 322 | 190 | 147 | 65 | 60 | 42 |
| 12865573 | PTPN6 | protein tyrosine phosphatase, non-receptor type 6 | signaling | 4.554 | 2.572 | 2.796 | 32 | 30 | 40 | 24 | 58 | 49 | 57 | 145 | 86 | 225 | 45 | 51 | 55 |
| 12849263 | NOS3 | nitric oxide synthase 3 (endothelial cell) | signaling | 4.702 | 3.118 | 2.773 | 43 | 35 | 40 | 29 | 44 | 54 | 70 | 164 | 180 | 172 | 57 | 81 | 51 |
| 12751873 | AXL | AXL Receptor Tyrosine Kinase | signaling | 4.718 | 2.054 | 2.794 | 55 | 41 | 45 | 30 | 115 | 105 | 73 | 224 | 182 | 187 | 66 | 74 | 72 |
| 12783627 | DOCK10 | dedicator of cytokinesis 10 | signaling | 4.964 | 3.462 | 7.573 | 37 | 34 | 28 | 34 | 50 | 46 | 47 | 224 | 98 | 205 | 25 | 20 | 20 |
| 12805964 | RCAN2 | Regulator Of Calcineurin 2 | signaling | 5.061 | 5.414 | 3.99 | 34 | 27 | 42 | 31 | 31 | 35 | 28 | 204 | 137 | 167 | 35 | 43 | 48 |
| 12726941 | TRPC6 | transient receptor potential cation channel, subfamily C, member 6 | signaling | 5.135 | 2.476 | 4.042 | 20 | 20 | 14 | 26 | 43 | 37 | 41 | 82 | 96 | 126 | 26 | 23 | 24 |
| 12796510 | SNRK | PREDICTED: SNF related kinase | signaling | 5.21 | 3.194 | 2.156 | 121 | 112 | 120 | 139 | 152 | 229 | 229 | 748 | 589 | 594 | 297 | 368 | 238 |
| 12731162 | ZNF259 | zinc finger protein 259; ZPR1 Zinc Finger | signaling | 5.252 | 6.171 | 4.616 | 276 | 276 | 301 | 334 | 256 | 257 | 243 | 1713 | 1222 | 1790 | 385 | 332 | 297 |
| 12806404 | TNFRSF21 | tumor necrosis factor receptor superfamily, member 21 | signaling | 5.628 | 2.878 | 2.545 | 27 | 22 | 24 | 21 | 39 | 62 | 38 | 160 | 121 | 115 | 59 | 49 | 47 |
| 12698197 | NRARP | PREDICTED: NOTCH-regulated ankyrin repeat protein | signaling | 5.808 | 3.255 | 2.915 | 68 | 53 | 67 | 66 | 101 | 126 | 113 | 424 | 378 | 309 | 142 | 129 | 109 |
| 12885817 | HBEGF | heparin-binding EGF-like growth factor | signaling | 5.865 | 4.448 | 2.054 | 61 | 41 | 39 | 46 | 52 | 71 | 60 | 288 | 266 | 252 | 128 | 130 | 134 |
| 12682057 | KALRN | kalirin, RhoGEF kinase | signaling | 5.984 | 3.288 | 2.077 | 41 | 28 | 35 | 28 | 53 | 85 | 46 | 166 | 217 | 207 | 81 | 87 | 118 |
| 12864595 | MFNG | MFNG O-fucosylpeptide 3-beta-N-acetylglucosaminyltransferase | signaling | 6.057 | 2.993 | 2.344 | 33 | 35 | 33 | 39 | 70 | 89 | 56 | 249 | 218 | 174 | 100 | 99 | 74 |
| 12882470 | NOTCH3 | Notch 3 | signaling | 6.182 | 2.08 | 3.17 | 39 | 37 | 43 | 40 | 110 | 135 | 110 | 309 | 225 | 212 | 92 | 81 | 62 |
| 12713738 | PPP1R16B | protein phosphatase 1, regulatory subunit 16B | signaling | 6.233 | 3.6 | 2.872 | 39 | 34 | 26 | 32 | 54 | 62 | 54 | 215 | 247 | 159 | 61 | 94 | 62 |
| 12702269 | TGFA | Transforming Growth Factor, Alpha | signaling | 6.516 | 5.597 | 2.22 | 49 | 50 | 52 | 49 | 59 | 61 | 55 | 305 | 285 | 393 | 162 | 101 | 190 |
| 12857319 | PTHLH | Parathyroid Hormone-Like Hormone | signaling | 6.703 | 15.349 | 23.648 | 283 | 216 | 512 | 187 | 107 | 92 | 180 | 2132 | 1259 | 2382 | 92 | 49 | 107 |
| 12835181 | ROBO4 | roundabout homolog 4, magic roundabout (Drosophila) | signaling | 6.805 | 2.489 | 2.059 | 46 | 33 | 26 | 32 | 80 | 127 | 76 | 268 | 187 | 237 | 115 | 113 | 105 |
| 12843050 | WNT2B | wingless-type MMTV integration site family, member 2B | signaling | 7.229 | 4.84 | 5.023 | 44 | 33 | 43 | 25 | 61 | 56 | 43 | 253 | 149 | 446 | 41 | 43 | 74 |
| 12909088 | BMX | BMX non-receptor tyrosine kinase | signaling | 7.531 | 6.889 | 4.755 | 20 | 15 | 15 | 21 | 18 | 19 | 20 | 115 | 142 | 139 | 24 | 38 | 23 |
| 12788338 | SLIT3 | slit homolog 3 (Drosophila) | signaling | 7.657 | 2.527 | 4.585 | 35 | 35 | 42 | 31 | 158 | 106 | 73 | 265 | 273 | 274 | 51 | 67 | 60 |
| 12848232 | PEAR1 | platelet endothelial aggregation receptor 1 | signaling | 8.235 | 2.122 | 2.804 | 38 | 27 | 25 | 21 | 90 | 148 | 88 | 237 | 248 | 191 | 84 | 112 | 54 |
| 12850708 | TSPAN13 | tetraspanin 13 | signaling | 8.369 | 5.696 | 2.771 | 68 | 77 | 134 | 96 | 119 | 143 | 138 | 870 | 623 | 800 | 268 | 249 | 306 |
| 12867247 | ACVRL1 | activin A receptor type II-like 1 | signaling | 8.607 | 3.206 | 2.697 | 51 | 26 | 28 | 38 | 93 | 93 | 94 | 297 | 351 | 257 | 98 | 142 | 98 |
| 12732659 | OLFML1 | olfactomedin-like 1 | signaling | 8.685 | -2.28 | 3.093 | 28 | 25 | 21 | 19 | 287 | 626 | 525 | 234 | 189 | 180 | 57 | 72 | 66 |
| 12843108 | PLK3 | polo-like kinase 3 | signaling | 8.851 | 5.441 | 2.277 | 39 | 40 | 35 | 33 | 61 | 61 | 57 | 311 | 323 | 340 | 137 | 140 | 152 |
| 12861534 | EPS8 | epidermal growth factor receptor pathway substrate 8 | signaling | 8.933 | 6.116 | 3.722 | 23 | 23 | 25 | 19 | 29 | 41 | 31 | 198 | 191 | 216 | 64 | 48 | 51 |
| 12867502 | PDE3A | PREDICTED: phosphodiesterase 3A, cGMP-inhibited | signaling | 8.988 | 2.273 | 3.557 | 14 | 18 | 23 | 17 | 56 | 80 | 75 | 162 | 158 | 157 | 52 | 41 | 42 |
| 12826450 | UNC5B | unc-5 homolog B (C. elegans) | signaling | 9.217 | 6.684 | 2.386 | 56 | 38 | 58 | 51 | 63 | 75 | 68 | 553 | 505 | 351 | 207 | 212 | 164 |
| 12755764 | GNAO1 | Guanine Nucleotide Binding Protein (G Protein), Alpha Activating Activity Polypeptide O | signaling | 9.221 | 5.18 | 3.152 | 20 | 22 | 22 | 28 | 42 | 61 | 26 | 259 | 118 | 305 | 59 | 58 | 87 |
| 12862540 | CLEC2D | PREDICTED: C-type lectin domain family 2, member D, transcript variant 2 | signaling | 9.473 | 2.83 | 2.099 | 20 | 16 | 17 | 13 | 42 | 61 | 65 | 116 | 193 | 167 | 75 | 65 | 82 |
| 12850633 | RUNDC3B | RUN domain containing 3B | signaling | 9.56 | 3.638 | 2.614 | 24 | 15 | 20 | 13 | 30 | 63 | 53 | 162 | 173 | 171 | 56 | 69 | 69 |
| 12854454 | RAPGEF5 | PREDICTED: Rap guanine nucleotide exchange factor (GEF) 5 | signaling | 9.66 | 4.716 | 2.788 | 21 | 15 | 22 | 18 | 31 | 50 | 37 | 199 | 213 | 142 | 71 | 75 | 52 |
| 12762058 | TMEM88 | transmembrane protein 88 | signaling | 9.963 | 2.186 | 2.048 | 69 | 72 | 71 | 43 | 313 | 304 | 240 | 725 | 604 | 546 | 272 | 358 | 286 |
| 12861386 | CLEC1A | C-type lectin domain family 1, member A | signaling | 10.253 | 3.193 | 2.999 | 22 | 21 | 18 | 16 | 43 | 72 | 78 | 198 | 193 | 204 | 72 | 67 | 60 |
| 12842552 | TIE1 | tyrosine kinase with immunoglobulin-like and EGF-like domains 1 | signaling | 10.624 | 4.254 | 2.621 | 23 | 14 | 38 | 21 | 47 | 70 | 54 | 246 | 261 | 213 | 97 | 108 | 72 |
| 12738588 | RGS16 | regulator of G-protein signaling 16 | signaling | 10.801 | 8.258 | -2.337 | 36 | 25 | 25 | 24 | 32 | 35 | 40 | 261 | 408 | 239 | 653 | 874 | 571 |
| 12684815 | EVA1C | protein eva-1 homolog C (C1H21orf63 ) | signaling | 11.04 | 6.376 | 3.924 | 26 | 19 | 28 | 17 | 29 | 56 | 34 | 206 | 263 | 265 | 68 | 67 | 52 |
| 12799597 | SEMA3F | PREDICTED: sema domain, immunoglobulin domain (Ig), short basic domain, secreted, (semaphorin) 3F | signaling | 11.05 | 4.682 | 2.017 | 46 | 33 | 39 | 34 | 79 | 110 | 79 | 420 | 463 | 366 | 193 | 230 | 195 |
| 12796992 | MUSTN1 | musculoskeletal, embryonic nuclear protein 1 | signaling | 11.478 | 2.585 | 2.611 | 46 | 31 | 36 | 27 | 140 | 181 | 140 | 441 | 382 | 366 | 155 | 200 | 112 |
| 12898060 | GPR126 | Adhesion G Protein-Coupled Receptor G6 (ADGRG6), formerly GPR126 | signaling | 11.844 | 3.081 | 4.567 | 17 | 17 | 17 | 19 | 39 | 88 | 87 | 216 | 186 | 215 | 45 | 45 | 45 |
| 12867769 | APOLD1 | apolipoprotein L domain containing 1 | signaling | 12.133 | 6.41 | 3.234 | 46 | 31 | 45 | 34 | 51 | 91 | 83 | 400 | 445 | 567 | 135 | 156 | 142 |
| 12892571 | TEK | TEK tyrosine kinase, endothelial | signaling | 12.33 | 3.891 | 2.748 | 28 | 21 | 34 | 27 | 57 | 124 | 90 | 323 | 412 | 280 | 111 | 171 | 95 |
| 12732746 | SWAP70 | SWAP switching B-cell complex 70kDa subunit | signaling | 12.534 | 5.941 | 2.43 | 35 | 39 | 36 | 47 | 72 | 92 | 85 | 405 | 499 | 584 | 197 | 181 | 231 |
| 12711148 | EDNRB | Endothelin Receptor Type B | signaling | 12.696 | 5.888 | 4.124 | 20 | 21 | 23 | 19 | 36 | 57 | 43 | 266 | 274 | 246 | 67 | 83 | 46 |
| 12836019 | PTGER3 | prostaglandin E receptor 3 (subtype EP3) | signaling | 12.843 | 13.188 | 10.002 | 14 | 17 | 15 | 17 | 15 | 14 | 18 | 204 | 96 | 422 | 20 | 17 | 24 |
| 12722624 | PREX2 | phosphatidylinositol-3,4,5-trisphosphate-dependent Rac exchange factor 2 | signaling | 12.911 | 4.913 | 4.215 | 16 | 17 | 13 | 17 | 28 | 57 | 43 | 193 | 218 | 196 | 49 | 53 | 43 |
| 12685166 | S100B | S100 calcium binding protein B | signaling | 13.335 | 3.464 | 2.175 | 45 | 46 | 44 | 34 | 127 | 150 | 218 | 493 | 622 | 562 | 351 | 165 | 290 |
| 12874999 | PRKG2 | Protein Kinase, CGMP-Dependent, Type II | signaling | 13.412 | 11.892 | 10.471 | 9 | 9 | 8 | 11 | 10 | 13 | 9 | 152 | 70 | 185 | 11 | 13 | 12 |
| 12723906 | PTP4A3 | protein tyrosine phosphatase type IVA, member 3 | signaling | 13.536 | 4.907 | 2.86 | 34 | 25 | 35 | 29 | 73 | 97 | 85 | 478 | 392 | 378 | 170 | 161 | 110 |
| 12710948 | EFNB2 | PREDICTED: ephrin-B2 | signaling | 13.551 | 7.197 | 2.558 | 25 | 26 | 30 | 19 | 40 | 55 | 47 | 304 | 495 | 254 | 145 | 141 | 112 |
| 12837477 | CXCR7 | Atypical Chemokine Receptor 3 (ACKR3; CXCR7) | signaling | 13.727 | 3.925 | 2.633 | 34 | 34 | 40 | 28 | 83 | 119 | 164 | 465 | 409 | 511 | 225 | 113 | 209 |
| 12855665 | RAMP3 | receptor (G protein-coupled) activity modifying protein 3 | signaling | 14.424 | 6.357 | 2.492 | 27 | 37 | 28 | 37 | 61 | 79 | 77 | 472 | 516 | 393 | 199 | 238 | 130 |
| 12829004 | C28H10orf54 | chromosome 28 open reading frame, human C10orf54 | signaling | 14.621 | 3.79 | 3.693 | 31 | 25 | 29 | 30 | 104 | 154 | 84 | 504 | 374 | 392 | 136 | 110 | 98 |
| 12772627 | TMEM100 | transmembrane protein 100 | signaling | 15.111 | 4.717 | 4.577 | 28 | 39 | 30 | 22 | 69 | 104 | 110 | 492 | 386 | 436 | 91 | 95 | 100 |
| 12876951 | ADRB2 | adrenergic, beta-2-, receptor, surface | signaling | 15.215 | 8.419 | 3.251 | 15 | 19 | 26 | 20 | 29 | 48 | 31 | 247 | 358 | 295 | 75 | 107 | 94 |
| 12713091 | LPAR6 | lysophosphatidic acid receptor 6 | signaling | 15.233 | 2.539 | 2.145 | 14 | 23 | 20 | 29 | 74 | 155 | 169 | 343 | 292 | 317 | 151 | 163 | 130 |
| 12830373 | CDKN1C | cyclin-dependent kinase inhibitor 1C (p57, Kip2) | signaling | 15.244 | 5.528 | 7.159 | 68 | 51 | 78 | 58 | 166 | 150 | 212 | 860 | 584 | 1776 | 137 | 141 | 126 |
| 12886046 | PDGFRB | platelet-derived growth factor receptor, beta polypeptide | signaling | 15.381 | 2.508 | 3.24 | 42 | 38 | 35 | 33 | 183 | 318 | 198 | 652 | 500 | 558 | 241 | 161 | 138 |
| 12842705 | PTGFR | prostaglandin F receptor (FP) | signaling | 16.117 | 27.631 | 6.381 | 457 | 469 | 211 | 607 | 281 | 155 | 307 | 6932 | 5607 | 7278 | 1199 | 735 | 1235 |
| 12842602 | S1PR1 | sphingosine-1-phosphate receptor 1 | signaling | 18.121 | 3.898 | 2.476 | 35 | 20 | 32 | 26 | 103 | 170 | 122 | 513 | 525 | 467 | 200 | 254 | 163 |
| 12856728 | NTS | neurotensin | signaling | 18.883 | 21.09 | 17.127 | 14 | 15 | 15 | 16 | 13 | 12 | 15 | 391 | 120 | 475 | 15 | 18 | 17 |
| 12712638 | GPR183 | G protein-coupled receptor 183 | signaling | 19.217 | 22.186 | 25.888 | 53 | 63 | 80 | 65 | 56 | 48 | 65 | 1243 | 757 | 2022 | 31 | 60 | 60 |
| 12861362 | PTPRR | Protein Tyrosine Phosphatase, Receptor Type, R | signaling | 20.777 | 7.135 | 8.375 | 17 | 16 | 15 | 18 | 40 | 67 | 43 | 446 | 245 | 384 | 41 | 32 | 55 |
| 12691467 | RHOJ | ras homolog gene family, member J | signaling | 20.868 | 3.886 | 2.42 | 31 | 32 | 25 | 36 | 137 | 206 | 160 | 675 | 718 | 547 | 239 | 378 | 207 |
| 12703748 | ENG | Endoglin | signaling | 21.572 | 4.109 | 2.218 | 66 | 49 | 48 | 54 | 200 | 361 | 314 | 1293 | 1231 | 989 | 533 | 604 | 448 |
| 12721237 | JAG1 | jagged 1 | signaling | 22.713 | 2.637 | 2.19 | 59 | 43 | 52 | 31 | 344 | 454 | 367 | 1024 | 1044 | 985 | 493 | 566 | 359 |
| 12796464 | TGFBR2 | transforming growth factor, beta receptor II (70/80kDa) | signaling | 23.878 | 3.991 | 2.053 | 72 | 81 | 76 | 61 | 323 | 559 | 448 | 1953 | 2006 | 1316 | 950 | 955 | 656 |
| 12696035 | RASGRP1 | RAS guanyl releasing protein 1 (calcium and DAG-regulated) | signaling | 26.73 | 25.621 | 16.576 | 49 | 42 | 49 | 37 | 45 | 44 | 48 | 1361 | 760 | 1560 | 58 | 49 | 124 |
| 12840087 | ELTD1 | Adhesion G Protein-Coupled Receptor L4 (ADGRL4; ELTD1) | signaling | 27.264 | 3.07 | 2.842 | 25 | 26 | 28 | 26 | 134 | 338 | 271 | 742 | 622 | 772 | 311 | 292 | 171 |
| 12880284 | GRIA1 | ionotropic glutamate receptor AMPA 1, GRIA1-high allele | signaling | 30.974 | 29.716 | 27.611 | 15 | 13 | 16 | 12 | 13 | 16 | 14 | 515 | 263 | 593 | 18 | 13 | 16 |
| 12697629 | ANG2 | angiogenin 2 | signaling | 31.246 | 20.426 | 8.158 | 12 | 16 | 17 | 13 | 15 | 25 | 29 | 591 | 257 | 619 | 80 | 36 | 60 |
| 12848067 | KIAA1324 | PREDICTED: KIAA1324 ortholog, transcript variant 1 | signaling | 31.995 | 33.173 | 33.005 | 27 | 26 | 25 | 24 | 24 | 25 | 25 | 1026 | 495 | 1067 | 26 | 26 | 23 |
| 12871206 | PARM1 | prostate androgen-regulated mucin-like protein 1 | signaling | 35.042 | 19.633 | 9.64 | 32 | 29 | 29 | 29 | 49 | 57 | 54 | 1194 | 912 | 1041 | 118 | 127 | 85 |
| 12709110 | FLT1 | fms-related tyrosine kinase 1 (vascular endothelial growth factor/vascular permeability factor receptor) | signaling | 35.058 | 7.192 | 3.064 | 31 | 26 | 30 | 23 | 78 | 192 | 158 | 1030 | 1112 | 772 | 382 | 380 | 212 |
| 12899020 | MYCT1 | Myc Target 1 | signaling | 35.522 | 3.884 | 2.61 | 20 | 28 | 22 | 17 | 96 | 311 | 252 | 744 | 823 | 723 | 277 | 364 | 247 |
| 12857922 | PTPRB | protein tyrosine phosphatase, receptor type, B | signaling | 35.941 | 3.732 | 3.321 | 18 | 17 | 17 | 17 | 100 | 244 | 188 | 684 | 642 | 541 | 184 | 225 | 157 |
| 12786321 | ESM1 | Endothelial Cell-Specific Molecule 1 | signaling | 38.805 | 11.025 | 3.073 | 48 | 56 | 29 | 46 | 93 | 186 | 207 | 2013 | 1458 | 1635 | 758 | 579 | 377 |
| 12806131 | GPR116 | G protein-coupled receptor 116; Adhesion G Protein-Coupled Receptor F5 (ADGRF5) | signaling | 39.138 | 5.428 | 3.188 | 15 | 13 | 14 | 14 | 51 | 186 | 110 | 657 | 499 | 508 | 195 | 187 | 142 |
| 12811468 | PRKCB | protein kinase C, beta | signaling | 40.869 | 4.073 | 4.247 | 17 | 19 | 21 | 28 | 236 | 187 | 204 | 1008 | 578 | 1045 | 178 | 203 | 219 |
| 12900295 | SGK1 | serum/glucocorticoid regulated kinase 1 | signaling | 40.962 | 10.66 | 2.313 | 37 | 37 | 76 | 20 | 94 | 195 | 169 | 1485 | 1392 | 1812 | 675 | 660 | 679 |
| 12873413 | KDR | kinase insert domain receptor (a type III receptor tyrosine kinase) | signaling | 67.951 | 5.542 | 3.069 | 45 | 20 | 20 | 22 | 134 | 517 | 410 | 1994 | 1632 | 1484 | 618 | 689 | 392 |
| 12777248 | CALCRL | calcitonin receptor-like | signaling | 68.801 | 4.293 | 3.149 | 11 | 12 | 12 | 13 | 93 | 280 | 266 | 779 | 880 | 802 | 242 | 341 | 213 |
| 12681340 | TM4SF1 | transmembrane 4 L six family member 1 | signaling | 72.962 | 5.072 | 2.17 | 42 | 26 | 27 | 27 | 190 | 619 | 669 | 2130 | 2142 | 2256 | 980 | 1011 | 1016 |
| 12679297 | TNFSF10 | PREDICTED: tumor necrosis factor (ligand) superfamily, member 10 | signaling | 90.011 | 3.509 | 2.476 | 23 | 31 | 23 | 27 | 267 | 909 | 1208 | 2338 | 2210 | 2453 | 903 | 1025 | 902 |
| 12785083 | PRLR | Prolactin Receptor | signaling | 91.769 | 54.487 | 19.275 | 15 | 13 | 17 | 14 | 29 | 28 | 19 | 1735 | 860 | 1692 | 73 | 114 | 42 |
| 12697507 | ANG | angiogenin, ribonuclease, RNase A family, 5 | signaling | 118.604 | 49.113 | 11.857 | 8 | 7 | 18 | 13 | 17 | 22 | 47 | 1259 | 839 | 2002 | 230 | 58 | 95 |
| 12840861 | RGS5 | regulator of G-protein signaling 5 | signaling | 138.356 | 4.505 | 4.256 | 23 | 30 | 19 | 23 | 392 | 1159 | 812 | 3499 | 3204 | 3009 | 1203 | 828 | 438 |
| 12891585 | BNC2 | PREDICTED: basonuclin 2, transcript variant 2 | transcription | -2.35 | -2.58 | -2.082 | 287 | 332 | 268 | 295 | 322 | 346 | 303 | 114 | 151 | 114 | 212 | 282 | 297 |
| 12862306 | NRIP2 | PREDICTED: nuclear receptor interacting protein 2, transcript variant 3 | transcription | 2.745 | 2.19 | 2.082 | 65 | 60 | 58 | 55 | 78 | 80 | 66 | 175 | 142 | 174 | 83 | 58 | 99 |
| 12873208 | HOPX | HOP homeobox | transcription | 4.456 | 2.53 | 3.906 | 50 | 35 | 32 | 21 | 58 | 70 | 48 | 186 | 123 | 138 | 43 | 45 | 27 |
| 12786378 | MSX2 | Msh Homeobox 2 | transcription | 4.703 | 4.275 | 4.052 | 23 | 25 | 18 | 23 | 24 | 24 | 25 | 87 | 63 | 200 | 23 | 28 | 26 |
| 12714026 | SNAI1 | snail homolog 1 (Drosophila) | transcription | 6.021 | 4.561 | 2.307 | 28 | 22 | 25 | 24 | 36 | 31 | 30 | 165 | 168 | 115 | 63 | 83 | 49 |
| 12751361 | APLP1 | amyloid beta (A4) precursor-like protein 1 | transcription | 6.052 | 7.573 | 8.107 | 50 | 50 | 64 | 35 | 35 | 49 | 35 | 419 | 157 | 394 | 38 | 35 | 36 |
| 12720518 | FAM107B | family with sequence similarity 107, member B | transcription | 6.899 | 3.176 | 2.597 | 16 | 14 | 15 | 17 | 23 | 46 | 35 | 127 | 107 | 86 | 40 | 38 | 43 |
| 12781647 | STAT4 | signal transducer and activator of transcription 4 | transcription | 7.138 | 10.353 | 8.852 | 78 | 103 | 93 | 175 | 60 | 100 | 67 | 790 | 510 | 1104 | 85 | 54 | 141 |
| 12724359 | HEY1 | hairy/enhancer-of-split related with YRPW motif 1 | transcription | 7.187 | 4.412 | 4.086 | 22 | 12 | 16 | 15 | 27 | 32 | 21 | 113 | 126 | 110 | 35 | 27 | 24 |
| 12715424 | SOX18 | SRY (sex determining region Y)-box 18 | transcription | 8.485 | 4.606 | 2.422 | 42 | 29 | 40 | 36 | 57 | 74 | 73 | 271 | 459 | 238 | 155 | 129 | 104 |
| 12733478 | LMO2 | LIM Domain Only 2 (Rhombotin-Like 1) | transcription | 8.713 | 3.85 | 2.149 | 95 | 76 | 97 | 68 | 188 | 190 | 187 | 796 | 692 | 693 | 331 | 475 | 244 |
| 12890889 | NR4A3 | PREDICTED: nuclear receptor subfamily 4, group A, member 3 | transcription | 9.021 | 3.737 | 2.5 | 25 | 25 | 36 | 34 | 98 | 48 | 76 | 241 | 263 | 298 | 116 | 109 | 96 |
| 12699543 | REL | v-rel reticuloendotheliosis viral oncogene homolog (avian) | transcription | 9.315 | 3.747 | 2.145 | 16 | 17 | 26 | 19 | 40 | 56 | 46 | 160 | 194 | 178 | 68 | 84 | 98 |
| 12887005 | EBF1 | early B-cell factor 1 | transcription | 10.969 | 4.538 | 3.523 | 25 | 21 | 22 | 25 | 41 | 73 | 58 | 279 | 272 | 211 | 82 | 82 | 54 |
| 12862103 | ELK3 | ELK3, ETS-domain protein (SRF accessory protein 2) | transcription | 11.081 | 2.951 | 2.003 | 40 | 44 | 44 | 41 | 115 | 234 | 147 | 480 | 575 | 368 | 220 | 301 | 191 |
| 12685825 | ERG | V-Ets Avian Erythroblastosis Virus E26 Oncogene Homolog | transcription | 12.832 | 3.443 | 2.89 | 23 | 28 | 27 | 19 | 70 | 120 | 82 | 320 | 323 | 276 | 107 | 130 | 85 |
| 12798575 | BHLHE40 | basic helix-loop-helix family, member e40 | transcription | 13.109 | 16.08 | 2.218 | 89 | 142 | 287 | 148 | 151 | 113 | 112 | 1966 | 1695 | 2384 | 927 | 882 | 890 |
| 12831228 | FLI1 | Friend leukemia virus integration 1 | transcription | 13.645 | 3.035 | 2.641 | 48 | 40 | 47 | 28 | 137 | 241 | 177 | 579 | 516 | 549 | 203 | 251 | 174 |
| 12681285 | MECOM | PREDICTED: MDS1 and EVI1 complex locus, transcript variant 1 | transcription | 18.416 | 6.315 | 4.422 | 20 | 22 | 22 | 26 | 38 | 99 | 71 | 447 | 393 | 388 | 94 | 92 | 92 |
| 12686304 | HES1 | hairy and enhancer of split 1, (Drosophila) | transcription | 21.998 | 8.699 | 3.015 | 26 | 24 | 25 | 17 | 48 | 85 | 47 | 553 | 527 | 432 | 174 | 193 | 137 |
| 12891421 | NFIB | nuclear factor I/B | transcription | 24.819 | 2.171 | 2.145 | 28 | 28 | 21 | 24 | 267 | 376 | 237 | 677 | 688 | 523 | 270 | 335 | 273 |
| 12834727 | ETS1 | v-ets erythroblastosis virus E26 oncogene homolog 1 (avian) | transcription | 27.308 | 4.988 | 2.6 | 23 | 14 | 21 | 27 | 109 | 135 | 99 | 616 | 568 | 518 | 203 | 262 | 193 |
| 12698660 | EPAS1 | Endothelial PAS Domain Protein 1 | transcription | 37.835 | 6.522 | 2.628 | 83 | 90 | 88 | 83 | 348 | 681 | 526 | 3415 | 3278 | 3092 | 1347 | 1288 | 1098 |
| 12809952 | MRO | maestro | unknown | -14.581 | -8.928 | -2.589 | 2699 | 2753 | 3221 | 2088 | 1763 | 1817 | 1347 | 176 | 227 | 152 | 382 | 650 | 424 |
| 12870843 | FAM114A1 | PREDICTED: family with sequence similarity 114, member A1 | unknown | -3.869 | -3.295 | -2.051 | 1953 | 2057 | 2123 | 2141 | 1756 | 1721 | 1804 | 604 | 548 | 460 | 1153 | 1193 | 955 |
| 12865365 | C5H12orf35 | chromosome 5 open reading frame, human C12orf35; KIAA1551 | unknown | -2.68 | -3.147 | -2.133 | 1706 | 1915 | 1794 | 2179 | 2431 | 1871 | 2404 | 725 | 777 | 623 | 1270 | 1634 | 1642 |
| 12728090 | TP53I11 | tumor protein p53 inducible protein 11 | unknown | 2.042 | 2.958 | 3.067 | 137 | 135 | 106 | 157 | 102 | 92 | 82 | 385 | 215 | 240 | 98 | 94 | 75 |
| 12901444 | LRP11 | low density lipoprotein receptor-related protein 11 | unknown | 2.149 | 3.051 | 2.121 | 167 | 206 | 188 | 190 | 114 | 122 | 165 | 435 | 327 | 458 | 252 | 178 | 152 |
| 12844716 | ENSBTAT00000012885 | cdna:known chromosome:UMD3.1:3:84711664:84712071:-1 | unknown | 3.074 | 4.862 | 2.809 | 33 | 50 | 29 | 65 | 41 | 29 | 16 | 141 | 94 | 167 | 73 | 37 | 37 |
| 12679157 | LRRC3 | Leucine Rich Repeat Containing 3 | unknown | 3.563 | 2.398 | 2.156 | 37 | 49 | 36 | 42 | 60 | 60 | 63 | 185 | 122 | 136 | 56 | 60 | 93 |
| 12791153 | FAM174B | Family With Sequence Similarity 174, Member B | unknown | 3.794 | 4.659 | 2.85 | 43 | 38 | 110 | 49 | 37 | 50 | 47 | 249 | 175 | 201 | 81 | 64 | 73 |
| 12905382 | PRRG3 | PREDICTED: proline rich Gla (G-carboxyglutamic acid) 3 (transmembrane) | unknown | 3.98 | 3.96 | 3.624 | 52 | 50 | 79 | 37 | 53 | 62 | 45 | 187 | 181 | 269 | 61 | 48 | 66 |
| 12686542 | MAP3K7CL | MAP3K7 C-Terminal Like; C1H21orf7 | unknown | 4.383 | 3.46 | 3.764 | 29 | 25 | 33 | 23 | 31 | 34 | 41 | 127 | 132 | 104 | 33 | 30 | 33 |
| 12908599 | LANCL3 | LanC lantibiotic synthetase component C-like 3 (bacterial) | unknown | 4.757 | 3.04 | 2.382 | 23 | 29 | 24 | 28 | 37 | 49 | 36 | 139 | 120 | 112 | 47 | 57 | 52 |
| 12795038 | C22H3orf64 | chromosome 22 open reading frame, human C3orf64 | unknown | 4.876 | 2.624 | 2.223 | 75 | 89 | 101 | 70 | 136 | 167 | 160 | 446 | 339 | 437 | 169 | 176 | 202 |
| 12899089 | SH3BGRL2 | SH3 domain binding glutamic acid-rich protein like 2 | unknown | 4.985 | 2.006 | 2.242 | 43 | 49 | 35 | 59 | 85 | 124 | 139 | 265 | 233 | 192 | 103 | 133 | 77 |
| 12719555 | FAM65C | PREDICTED: family with sequence similarity 65, member C | unknown | 5.18 | 3.357 | -2.884 | 38 | 25 | 35 | 29 | 50 | 47 | 48 | 179 | 171 | 138 | 405 | 611 | 409 |
| 12774181 | ENSBTAT00000015387 | cdna:known chromosome:UMD3.1:2:122824336:122835401:1 | unknown | 5.496 | 7.368 | 6.343 | 90 | 113 | 105 | 82 | 82 | 73 | 63 | 665 | 340 | 665 | 86 | 77 | 89 |
| 12727897 | OAF | PREDICTED: OAF homolog (Drosophila) | unknown | 5.9 | 3.408 | 3.527 | 66 | 65 | 51 | 50 | 96 | 103 | 100 | 378 | 286 | 362 | 90 | 113 | 87 |
| 12786303 | ENSBTAT00000054642 | cdna:known chromosome:UMD3.1:20:20088695:20200206:1 | unknown | 6.98 | 3.984 | 2.001 | 48 | 41 | 107 | 40 | 67 | 141 | 90 | 337 | 344 | 462 | 147 | 166 | 274 |
| 12875828 | FAM190A | family with sequence similarity 190, member A; Coiled-Coil Serine-Rich Protein 1 (CCSER1) | unknown | 7.012 | 3.167 | 4.837 | 24 | 18 | 24 | 18 | 51 | 56 | 33 | 175 | 92 | 188 | 32 | 32 | 26 |
| 12768239 | XM_002696071 | PREDICTED: intercellular adhesion molecule 2-like (LOC506088) | unknown | 8.949 | 6.731 | 2.936 | 19 | 18 | 18 | 14 | 19 | 23 | 25 | 141 | 149 | 164 | 42 | 53 | 61 |
| 12738139 | ENSBTAT00000023528 | cdna:pseudogene chromosome:UMD3.1:16:57861756:57862145:-1 | unknown | 9.019 | 2.966 | 2.259 | 30 | 37 | 34 | 26 | 77 | 108 | 107 | 338 | 315 | 220 | 157 | 134 | 96 |
| 12697619 | XM_001251830 | PREDICTED: ribonuclease 4-like (LOC783195) | unknown | 10.28 | 6.283 | 4.066 | 89 | 87 | 106 | 101 | 124 | 174 | 176 | 682 | 865 | 1602 | 504 | 193 | 145 |
| 12837541 | XM_586975 | PREDICTED: solute carrier family 16, member 1 (monocarboxylic acid transporter 1)-like (LOC509911) | unknown | 10.754 | 10.609 | 11.005 | 8 | 7 | 7 | 7 | 7 | 7 | 8 | 150 | 22 | 134 | 8 | 7 | 6 |
| 12899070 | FAM162B | Family With Sequence Similarity 162, Member B | unknown | 10.779 | 6.099 | 4.415 | 21 | 23 | 20 | 28 | 38 | 49 | 36 | 371 | 189 | 216 | 94 | 43 | 43 |
| 12905894 | CXHXorf36 | chromosome X open reading frame 36 | unknown | 11.148 | 2.989 | 2.702 | 26 | 24 | 28 | 38 | 70 | 163 | 103 | 321 | 389 | 254 | 113 | 162 | 87 |
| 12863031 | XM_001788179 | PREDICTED: C-type lectin domain family 2 member D11-like (LOC784451) | unknown | 13.689 | 4.181 | 2.503 | 25 | 18 | 27 | 16 | 46 | 83 | 85 | 302 | 327 | 241 | 106 | 139 | 102 |
| 12831015 | VWA5A | Von Willebrand Factor A Domain Containing 5A | unknown | 13.896 | 3.682 | 4.188 | 38 | 58 | 80 | 45 | 111 | 344 | 208 | 857 | 544 | 853 | 207 | 186 | 141 |
| 12858844 | NM_001100333 | apolipoprotein L, 3-like (LOC510193) | unknown | 14.444 | 6.084 | 2.484 | 26 | 26 | 19 | 22 | 35 | 73 | 63 | 407 | 337 | 264 | 164 | 168 | 86 |
| 12702649 | ENSBTAT00000011846 | cdna:known chromosome:UMD3.1:11:98509474:98513781:1 | unknown | 25.769 | 3.472 | 2.358 | 59 | 98 | 77 | 73 | 372 | 782 | 607 | 2143 | 2037 | 1693 | 865 | 1072 | 608 |
| 12682628 | CYYR1 | cysteine/tyrosine-rich 1 | unknown | 55.258 | 4.533 | 3.027 | 16 | 15 | 17 | 20 | 113 | 283 | 267 | 959 | 1005 | 824 | 313 | 391 | 233 |
| 12866290 | BPIFC | BPI fold containing family C | unknown | 69.516 | 64.194 | 4.677 | 12 | 14 | 12 | 11 | 12 | 14 | 13 | 911 | 739 | 832 | 182 | 101 | 296 |
| 12704678 | DYSF | dysferlin, limb girdle muscular dystrophy 2B (autosomal recessive) | vesicles | 6.899 | 4.241 | 2.323 | 73 | 69 | 65 | 80 | 115 | 132 | 104 | 567 | 508 | 414 | 249 | 255 | 150 |
