# Supplementary material for: Transcriptomes of bovine ovarian follicular and luteal cells
Source: Data Brief. 2016 Dec 10;10:335–9. doi: 10.1016/j.dib.2016.11.093 (PMC5157705; doi:10.1016/j.dib.2016.11.093)
Supplement: Supplementary file 5 — Supplementary material [file mmc5.docx]

| **Table 4. Genes Enriched (≥ 2-fold greater expression than all other cells types) or Decreased (≤ -2-fold) in Small Luteal Cells** | | | |  |  |  | **Linear Microarray Results (arbitrary units)** | | | | | | | | | | | | |
| --- | --- | --- | --- | --- | --- | --- | --- | --- | --- | --- | --- | --- | --- | --- | --- | --- | --- | --- | --- |
| **Probeset ID** | **Gene Symbol** | **Description** | **Functional Category** | **Fold Change (SLC vs GC)** | **Fold Change (SLC vs TC)** | **Fold Change (SLC vs LLC)** | **GC1** | **GC2** | **GC3** | **GC4** | **TC1** | **TC2** | **TC3** | **LLC1** | **LLC2** | **LLC3** | **SLC1** | **SLC2** | **SLC3** |
| 12780587 | TNFAIP6 | tumor necrosis factor, alpha-induced protein 6 | adhesion | -5.134 | -2.459 | -2.949 | 443 | 520 | 963 | 1200 | 436 | 210 | 445 | 645 | 236 | 462 | 173 | 96 | 165 |
| 12852910 | CD36 | CD36 molecule (thrombospondin receptor) | adhesion | 4.246 | 3.503 | 2.148 | 103 | 119 | 130 | 143 | 118 | 183 | 152 | 286 | 236 | 211 | 443 | 755 | 421 |
| 12683338 | CXADR | coxsackie virus and adenovirus receptor | adhesion | 8.992 | 3.318 | 2.16 | 110 | 98 | 97 | 82 | 216 | 238 | 345 | 475 | 380 | 358 | 1017 | 924 | 693 |
| 12678900 | CLDN1 | claudin 1 | adhesion | 9.635 | 4.495 | 2.689 | 43 | 34 | 89 | 35 | 90 | 100 | 109 | 181 | 166 | 153 | 431 | 443 | 469 |
| 12899536 | THBS2 | thrombospondin 2 | adhesion | 19.998 | 6.265 | 2.557 | 75 | 65 | 81 | 42 | 158 | 219 | 242 | 636 | 460 | 421 | 1291 | 1296 | 1234 |
| 12849788 | GPNMB | Glycoprotein (Transmembrane) Nmb | adhesion | 55.093 | 34.213 | 2.87 | 51 | 39 | 225 | 29 | 95 | 68 | 38 | 735 | 983 | 579 | 1841 | 2472 | 2172 |
| 12815958 | EMP2 | Epithelial Membrane Protein 2 | cell membrane composition | -2.91 | -2.361 | -2.042 | 885 | 802 | 492 | 735 | 631 | 650 | 470 | 541 | 472 | 488 | 222 | 276 | 239 |
| 12835715 | F3 | coagulation factor III (thromboplastin, tissue factor) | coagulation | 3.599 | 5.437 | 2.292 | 115 | 115 | 172 | 194 | 129 | 82 | 84 | 231 | 186 | 277 | 383 | 588 | 636 |
| 12798763 | XIRP1 | xin actin-binding repeat containing 1 | cytoskeletal dynamics | 2.471 | 2.568 | 2.154 | 57 | 60 | 65 | 60 | 51 | 60 | 64 | 63 | 67 | 78 | 104 | 172 | 184 |
| 12809935 | PSTPIP2 | proline-serine-threonine phosphatase interacting protein 2 | cytoskeletal dynamics | 7.516 | 3.116 | 2.042 | 28 | 37 | 33 | 20 | 75 | 66 | 67 | 115 | 107 | 95 | 250 | 211 | 190 |
| 12726116 | SNTB1 | PREDICTED: syntrophin, beta 1 (dystrophin-associated protein A1, 59kDa, basic component 1) | cytoskeletal dynamics | 21.423 | 2.76 | 2.074 | 42 | 33 | 32 | 34 | 235 | 320 | 268 | 357 | 449 | 297 | 635 | 812 | 823 |
| 12726834 | MMP7 | matrix metallopeptidase 7 (matrilysin, uterine) | extracellular matrix | 11.381 | 13.136 | 2.722 | 10 | 9 | 8 | 9 | 7 | 9 | 8 | 42 | 39 | 32 | 116 | 87 | 101 |
| 12679824 | COL6A1 | collagen, type VI, alpha 1 | extracellular matrix | 20.09 | 2.299 | 2.942 | 140 | 138 | 172 | 109 | 1232 | 1544 | 924 | 1182 | 1054 | 673 | 2461 | 3636 | 2389 |
| 12810233 | CCBE1 | Collagen And Calcium Binding EGF Domains 1 | extracellular matrix | 27.126 | 14.2 | 3.749 | 54 | 23 | 43 | 29 | 80 | 78 | 50 | 294 | 339 | 170 | 802 | 1558 | 712 |
| 12894605 | CD274 | CD274 molecule | immune response | 57.068 | 13.436 | 3.116 | 19 | 24 | 24 | 28 | 114 | 62 | 143 | 327 | 486 | 511 | 878 | 1562 | 1793 |
| 12686574 | PTX3 | pentraxin 3, long | immune response | 57.996 | 52.226 | 2.28 | 33 | 23 | 30 | 28 | 27 | 41 | 28 | 557 | 968 | 695 | 1448 | 1579 | 1942 |
| 12748645 | RYR1 | ryanodine receptor 1 (skeletal) | ion transport | 4.54 | 3.764 | 2.013 | 29 | 29 | 26 | 23 | 30 | 32 | 33 | 54 | 66 | 60 | 114 | 90 | 169 |
| 12779281 | KCNE4 | potassium voltage-gated channel, Isk-related family, member 4 | ion transport | 42.082 | 3.181 | 2.102 | 33 | 31 | 34 | 27 | 472 | 300 | 508 | 627 | 855 | 465 | 1416 | 1338 | 1222 |
| 12864151 | SLC2A3 | solute carrier family 2 (facilitated glucose transporter), member 3 | molecular transport | 2.613 | 7.038 | -2.154 | 100 | 109 | 189 | 201 | 53 | 59 | 48 | 801 | 633 | 1025 | 326 | 471 | 338 |
| 12764677 | ABCA6 | PREDICTED: ATP-binding cassette, sub-family A (ABC1), member 6 | molecular transport | 9.51 | 2.017 | 2.486 | 26 | 18 | 22 | 17 | 111 | 106 | 78 | 80 | 102 | 59 | 154 | 257 | 189 |
| 12876894 | GPX3 | glutathione peroxidase 3 (plasma) | oxidative stress response | -4.098 | -2.384 | -2.616 | 2678 | 2791 | 2844 | 1981 | 1423 | 1303 | 1755 | 1692 | 1426 | 1784 | 578 | 522 | 796 |
| 12679629 | PHLDB2 | pleckstrin homology-like domain, family B, member 2 | phospholipid binding | 43.791 | 2.469 | 2.283 | 26 | 27 | 23 | 23 | 433 | 545 | 351 | 383 | 614 | 446 | 699 | 1466 | 1218 |
| 12711403 | NHLRC3 | NHL repeat containing 3 | post-translational modification | 4.408 | 4.071 | 2.069 | 80 | 95 | 89 | 132 | 95 | 113 | 108 | 256 | 219 | 158 | 451 | 464 | 375 |
| 12815458 | DCUN1D3 | DCN1, defective in cullin neddylation 1, domain containing 3 (S. cerevisiae) | proliferation regulation | 3.202 | 3.902 | 2.16 | 123 | 168 | 135 | 125 | 116 | 111 | 110 | 185 | 240 | 187 | 386 | 522 | 417 |
| 12780349 | SERPINE2 | serpin peptidase inhibitor, clade E (nexin, plasminogen activator inhibitor type 1), member 2 | proteolysis regulation | -49.704 | -37.059 | -30.415 | 5603 | 6267 | 5637 | 7062 | 4752 | 4035 | 4945 | 5128 | 2555 | 3999 | 217 | 66 | 130 |
| 12834667 | PRSS23 | protease, serine, 23 | proteolysis regulation | -3.705 | -2.778 | -3.037 | 566 | 501 | 1157 | 588 | 520 | 422 | 559 | 687 | 435 | 536 | 214 | 194 | 138 |
| 12876696 | EFNA5 | ephrin-A5 | signal transduction | -20.753 | -4.565 | -2.147 | 3468 | 2781 | 4651 | 4359 | 786 | 951 | 745 | 327 | 439 | 404 | 167 | 215 | 163 |
| 12775041 | BMPR2 | Bone Morphogenetic Protein Receptor, Type II (Serine/Threonine Kinase) | signal transduction | -6.856 | -3.135 | -4.067 | 1541 | 1496 | 1647 | 1527 | 579 | 768 | 804 | 929 | 848 | 990 | 243 | 240 | 198 |
| 12873401 | BMPR1B | bone morphogenetic protein receptor, type IB | signal transduction | -6.168 | -4.467 | -2.519 | 944 | 960 | 1263 | 942 | 710 | 772 | 733 | 495 | 264 | 551 | 211 | 145 | 148 |
| 12699636 | LTBP1 | latent transforming growth factor beta binding protein 1 | signal transduction | -2.177 | -5.327 | -3.756 | 250 | 307 | 383 | 264 | 690 | 836 | 664 | 547 | 563 | 436 | 134 | 172 | 110 |
| 12872081 | BMP3 | bone morphogenetic protein 3 | signal transduction | 4.35 | 3.996 | 2.512 | 49 | 55 | 56 | 51 | 59 | 58 | 55 | 101 | 85 | 89 | 250 | 167 | 290 |
| 12804472 | PIM1 | Pim-1 Proto-Oncogene, Serine/Threonine Kinase | signal transduction | 7.654 | 6.036 | 2.524 | 104 | 101 | 66 | 67 | 139 | 100 | 83 | 239 | 259 | 254 | 499 | 866 | 587 |
| 12780430 | PTPRN | protein tyrosine phosphatase, receptor type, N | signal transduction | 9.179 | 9.495 | 3.782 | 27 | 22 | 25 | 25 | 23 | 23 | 26 | 52 | 66 | 64 | 143 | 335 | 246 |
| 12877273 | NR2F1 | Nuclear Receptor Subfamily 2, Group F, Member 1 | signal transduction | 9.3 | 2.697 | 2.156 | 71 | 58 | 79 | 88 | 225 | 273 | 260 | 265 | 411 | 287 | 576 | 742 | 736 |
| 12786665 | PLCXD3 | phosphatidylinositol-specific phospholipase C, X domain containing 3 | signal transduction | 12.083 | 3.801 | 3.029 | 20 | 16 | 26 | 22 | 63 | 58 | 80 | 91 | 108 | 58 | 295 | 274 | 197 |
| 12738588 | RGS16 | regulator of G-protein signaling 16 | signal transduction | 25.252 | 19.306 | 2.337 | 36 | 25 | 25 | 24 | 32 | 35 | 40 | 261 | 408 | 239 | 653 | 874 | 571 |
| 12774753 | PLA2R1 | phospholipase A2 receptor 1, 180kDa | signal transduction | 31.03 | 3.149 | 2.078 | 23 | 21 | 24 | 18 | 232 | 196 | 204 | 331 | 342 | 285 | 679 | 553 | 774 |
| 12859128 | C1R | complement component 1, r subcomponent | signal transduction | 37.48 | 6.438 | 2.144 | 40 | 18 | 34 | 29 | 188 | 155 | 164 | 490 | 501 | 526 | 1090 | 947 | 1235 |
| 12869844 | KIT | v-kit Hardy-Zuckerman 4 feline sarcoma viral oncogene homolog | signal transduction | 37.548 | 6.953 | 2.72 | 20 | 28 | 24 | 39 | 88 | 131 | 268 | 325 | 600 | 264 | 841 | 1138 | 1083 |
| 12872256 | DKK2 | dickkopf homolog 2 (Xenopus laevis) | signal transduction | 54.865 | 24.054 | 2.561 | 39 | 38 | 48 | 41 | 80 | 104 | 102 | 1011 | 1157 | 597 | 2040 | 2741 | 2099 |
| 12868860 | C1S | complement component 1, s subcomponent | signal transduction | 93.046 | 13.674 | 2.502 | 28 | 21 | 20 | 18 | 181 | 117 | 150 | 914 | 781 | 725 | 1938 | 2145 | 1952 |
| 12723704 | SNX31 | PREDICTED: sorting nexin 31 | signal transduction (protein-protein binding) | 4.346 | 2.507 | 3.179 | 42 | 38 | 45 | 43 | 71 | 76 | 72 | 69 | 64 | 42 | 147 | 277 | 149 |
| 12903812 | GAB3 | GRB2-associated binding protein 3 | signal transduction (protein-protein binding) | 6.765 | 2.171 | 3.016 | 33 | 33 | 36 | 36 | 115 | 120 | 90 | 108 | 69 | 62 | 251 | 314 | 161 |
| 12719139 | CASS4 | Cas Scaffolding Protein Family Member 4 | signal transduction (protein-protein binding) | 7.774 | 6.969 | 2.624 | 44 | 37 | 43 | 39 | 42 | 46 | 48 | 191 | 116 | 80 | 294 | 433 | 252 |
| 12682546 | SH3BGR | SH3 domain binding glutamic acid-rich protein | signal transduction (protein-protein binding) | 18.892 | 2.48 | 2.105 | 35 | 36 | 32 | 31 | 215 | 288 | 270 | 345 | 339 | 233 | 701 | 659 | 550 |
| 12734319 | DHRS3 | Dehydrogenase/Reductase (SDR Family) Member 3 | steroid/lipid metabolism | -5.331 | -6.974 | -3.725 | 764 | 608 | 496 | 439 | 1018 | 748 | 528 | 450 | 341 | 399 | 124 | 129 | 74 |
| 12807778 | LIPG | PREDICTED: lipase, endothelial | steroid/lipid metabolism | 24.945 | 5.147 | 2.578 | 21 | 18 | 15 | 14 | 65 | 83 | 101 | 174 | 173 | 144 | 382 | 370 | 522 |
| 12718058 | PTGIS | Prostaglandin I2 (Prostacyclin) Synthase | steroid/lipid metabolism | 44.065 | 7.716 | 2.45 | 44 | 36 | 50 | 37 | 225 | 315 | 185 | 1001 | 937 | 438 | 2065 | 2002 | 1461 |
| 12724640 | OSR2 | odd-skipped related 2 (Drosophila) | transcription | 29.984 | 4.215 | 2.369 | 32 | 28 | 33 | 30 | 288 | 233 | 155 | 342 | 543 | 315 | 852 | 1021 | 895 |
| 12697831 | LARP6 | La ribonucleoprotein domain family, member 6 | translation | 8.053 | 2.014 | 2.129 | 29 | 22 | 29 | 32 | 104 | 131 | 101 | 107 | 121 | 89 | 195 | 304 | 189 |
| 12793718 | TM6SF1 | transmembrane 6 superfamily member 1 | unknown | -5.016 | -2.405 | -5.037 | 931 | 844 | 657 | 598 | 412 | 379 | 292 | 917 | 452 | 1011 | 143 | 159 | 144 |
| 12815028 | MIR2384-1 | microRNA mir-2384-1 | unknown | 2.688 | 3.981 | 2.037 | 118 | 111 | 110 | 136 | 85 | 67 | 90 | 160 | 180 | 132 | 293 | 368 | 299 |
| 12712983 | FAM155A | family with sequence similarity 155, member A | unknown | 7.024 | 7.042 | 2.006 | 21 | 20 | 26 | 17 | 18 | 19 | 26 | 67 | 75 | 78 | 110 | 150 | 193 |
| 12788935 | TNFAIP2 | PREDICTED: tumor necrosis factor alpha-induced protein 2 | unknown | 8.304 | 3.624 | 3.101 | 62 | 60 | 49 | 45 | 292 | 87 | 73 | 140 | 155 | 136 | 362 | 440 | 551 |
| 12719555 | FAM65C | PREDICTED: family with sequence similarity 65, member C | unknown | 14.944 | 9.685 | 2.884 | 38 | 25 | 35 | 29 | 50 | 47 | 48 | 179 | 171 | 138 | 405 | 611 | 409 |
| 12825548 | ZMAT4 | zinc finger, matrin-type 4 | unknown | 16.154 | 2.565 | 2.78 | 33 | 31 | 33 | 35 | 142 | 202 | 317 | 228 | 203 | 154 | 524 | 687 | 426 |
| 12732039 | PAMR1 | peptidase domain containing associated with muscle regeneration 1 | unknown | 24.968 | 23.297 | 3.085 | 29 | 26 | 27 | 23 | 31 | 27 | 28 | 166 | 322 | 184 | 545 | 655 | 806 |
| 12744445 | RNF150 | PREDICTED: ring finger protein 150 | unknown | 38.636 | 6.27 | 2.284 | 13 | 18 | 28 | 17 | 132 | 140 | 78 | 270 | 426 | 260 | 551 | 749 | 863 |
| 12826119 | SRGN | serglycin | vesicles | -18.668 | -6.873 | -3.296 | 5427 | 6222 | 4866 | 7173 | 2934 | 1858 | 1841 | 1233 | 815 | 1101 | 354 | 346 | 252 |
| 12898561 | STX11 | syntaxin 11 | vesicles | 11.592 | 13.762 | 2.685 | 55 | 64 | 44 | 44 | 39 | 45 | 46 | 216 | 252 | 199 | 405 | 772 | 670 |
