# Supplementary material for: Transcriptomes of bovine ovarian follicular and luteal cells
Source: Data Brief. 2016 Dec 10;10:335–9. doi: 10.1016/j.dib.2016.11.093 (PMC5157705; doi:10.1016/j.dib.2016.11.093)
Supplement: Supplementary file 6 — Supplementary material [file mmc6.docx]

| **Table 5. Genes Enriched (≥ 2-fold greater) in Follicular Cells Compared to Luteal Cells** | | |  | **Linear Microarray Results (arbitrary units)** | | | | | | | | | | | | |
| --- | --- | --- | --- | --- | --- | --- | --- | --- | --- | --- | --- | --- | --- | --- | --- | --- |
| **Probe Set ID** | **Gene Symbol** | **Description** | **Fold Change Follicle vs Luteal** | **GC1** | **GC2** | **GC3** | **GC4** | **TC1** | **TC2** | **TC3** | **LLC1** | **LLC2** | **LLC3** | **SLC1** | **SLC2** | **SLC3** |
| 12858180 | AAAS | achalasia, adrenocortical insufficiency, alacrimia (AAAS), mRNA. | 2.24620467 | 325 | 285 | 281 | 186 | 181 | 145 | 149 | 92 | 99 | 87 | 112 | 98 | 105 |
| 12904893 | ABCB7 | ATP-binding cassette, sub-family B (MDR/TAP), member 7 (ABCB7), nuclear gene encoding mitochondrial protein, mRNA. | 2.313379717 | 324 | 367 | 327 | 426 | 220 | 234 | 208 | 150 | 147 | 129 | 126 | 108 | 120 |
| 12896865 | ABRACL | ABRA C-terminal like (ABRACL), mRNA. | 2.968625509 | 146 | 176 | 116 | 134 | 96 | 63 | 83 | 56 | 42 | 36 | 41 | 33 | 27 |
| 12792573 | ACSBG1 | acyl-CoA synthetase bubblegum family member 1 (ACSBG1), mRNA. | 4.15534793 | 110 | 144 | 277 | 94 | 109 | 81 | 99 | 37 | 32 | 35 | 35 | 18 | 32 |
| 12858784 | ADCY6 | adenylate cyclase 6 (ADCY6), mRNA. | 2.245821146 | 270 | 256 | 262 | 220 | 209 | 152 | 182 | 120 | 108 | 87 | 106 | 88 | 84 |
| 12904382 | AFF2 | AF4/FMR2 family, member 2 (AFF2), mRNA. | 6.26948401 | 1289 | 1463 | 1478 | 1377 | 817 | 747 | 739 | 203 | 173 | 185 | 166 | 158 | 196 |
| 12903165 | AGTR2 | Angiotensin II Receptor, Type 2 | 24.82353554 | 27 | 27 | 41 | 45 | 89 | 940 | 1229 | 12 | 15 | 11 | 11 | 15 | 18 |
| 12853180 | AKR1B1 | Aldo-Keto Reductase Family 1, Member B1 (Aldose Reductase) | 2.373761628 | 940 | 863 | 875 | 982 | 978 | 1172 | 1268 | 497 | 467 | 392 | 404 | 396 | 399 |
| 12844014 | ALG6 | asparagine-linked glycosylation 6, alpha-1,3-glucosyltransferase homolog (S. cerevisiae) (ALG6), mRNA. | 2.083149621 | 384 | 383 | 323 | 448 | 219 | 210 | 281 | 162 | 124 | 167 | 169 | 150 | 153 |
| 12831552 | ALG8 | asparagine-linked glycosylation 8, alpha-1,3-glucosyltransferase homolog (S. cerevisiae) (ALG8), mRNA. | 2.16293818 | 521 | 578 | 603 | 602 | 328 | 341 | 449 | 283 | 229 | 234 | 249 | 156 | 206 |
| 12866047 | AMHR2 | anti-Mullerian hormone receptor, type II (AMHR2), mRNA. | 2.097917279 | 168 | 179 | 255 | 142 | 123 | 119 | 110 | 73 | 82 | 77 | 87 | 69 | 59 |
| 12859490 | AMIGO2 | adhesion molecule with Ig-like domain 2 (AMIGO2), mRNA. | 3.03778837 | 472 | 466 | 520 | 577 | 405 | 341 | 274 | 121 | 125 | 94 | 167 | 206 | 149 |
| 12870991 | ANAPC4 | anaphase promoting complex subunit 4 (ANAPC4), mRNA. | 3.201156892 | 274 | 307 | 246 | 372 | 189 | 160 | 212 | 62 | 77 | 62 | 85 | 86 | 99 |
| 12827996 | ANK3 | ankyrin 3, node of Ranvier (ankyrin G) (ANK3), mRNA. | 8.648474086 | 169 | 245 | 209 | 245 | 173 | 215 | 71 | 24 | 20 | 23 | 26 | 17 | 22 |
| 12886277 | ANKRD34B | ankyrin repeat domain 34B (ANKRD34B), mRNA. | 8.907200249 | 212 | 264 | 182 | 105 | 280 | 273 | 253 | 24 | 21 | 27 | 28 | 26 | 25 |
| 12729722 | ANKRD49 | ankyrin repeat domain 49 (ANKRD49), mRNA. | 2.316842331 | 115 | 155 | 162 | 168 | 72 | 95 | 93 | 57 | 58 | 52 | 55 | 43 | 54 |
| 12860119 | ANKRD52 | ankyrin repeat domain 52 (ANKRD52), mRNA. | 2.286971191 | 333 | 346 | 430 | 320 | 254 | 202 | 222 | 165 | 152 | 126 | 124 | 123 | 99 |
| 12900330 | ANKRD6 | ankyrin repeat domain 6 (ANKRD6), mRNA. | 2.425174848 | 199 | 211 | 182 | 184 | 132 | 104 | 101 | 76 | 53 | 53 | 68 | 74 | 70 |
| 12854287 | ANLN | PREDICTED: anillin, actin binding protein (ANLN), mRNA. | 8.782451042 | 351 | 348 | 276 | 343 | 162 | 216 | 177 | 38 | 39 | 31 | 30 | 20 | 25 |
| 12859907 | ANO4 | anoctamin 4 (ANO4), mRNA. | 10.37048996 | 1055 | 1188 | 1163 | 1453 | 455 | 527 | 414 | 179 | 90 | 138 | 23 | 28 | 59 |
| 12857775 | APOBEC3B | apolipoprotein B mRNA editing enzyme, catalytic polypeptide-like 3B (APOBEC3B), mRNA. | 4.088159389 | 178 | 169 | 180 | 136 | 85 | 93 | 95 | 41 | 31 | 42 | 34 | 26 | 23 |
| 12724180 | ARFGEF1 | ADP-ribosylation factor guanine nucleotide-exchange factor 1 (brefeldin A-inhibited) (ARFGEF1), mRNA. | 2.443156921 | 955 | 1038 | 671 | 1270 | 485 | 467 | 417 | 306 | 306 | 311 | 283 | 348 | 306 |
| 12746214 | ARHGAP10 | Rho GTPase activating protein 10 (ARHGAP10), mRNA. | 2.537540929 | 241 | 233 | 259 | 234 | 576 | 671 | 492 | 160 | 161 | 160 | 130 | 155 | 147 |
| 12697409 | ARHGAP11A | PREDICTED: Rho GTPase activating protein 11A (ARHGAP11A), mRNA. | 19.57555607 | 451 | 457 | 449 | 475 | 112 | 98 | 114 | 25 | 16 | 11 | 9 | 18 | 15 |
| 12716760 | ARHGAP12 | Rho GTPase activating protein 12 (ARHGAP12), mRNA. | 2.093956561 | 286 | 356 | 264 | 377 | 214 | 201 | 179 | 119 | 115 | 134 | 132 | 127 | 142 |
| 12900030 | ARHGAP18 | Rho GTPase activating protein 18 (ARHGAP18), mRNA. | 2.871782172 | 535 | 580 | 1191 | 649 | 426 | 404 | 605 | 280 | 213 | 254 | 181 | 162 | 219 |
| 12827931 | ARHGAP22 | Rho GTPase Activating Protein 22 | 2.373165983 | 97 | 104 | 171 | 129 | 108 | 128 | 134 | 48 | 45 | 58 | 57 | 49 | 57 |
| 12685559 | ARHGEF26 | Rho guanine nucleotide exchange factor (GEF) 26 (ARHGEF26), mRNA. | 4.494441228 | 38 | 52 | 50 | 88 | 115 | 200 | 155 | 19 | 33 | 17 | 14 | 25 | 26 |
| 12715823 | ARMC4 | armadillo repeat containing 4 (ARMC4), mRNA. | 3.655275887 | 69 | 93 | 108 | 134 | 50 | 57 | 53 | 24 | 26 | 20 | 22 | 19 | 22 |
| 12849070 | ASB15 | Ankyrin Repeat And SOCS Box Containing 15 | 5.984039082 | 126 | 150 | 159 | 113 | 46 | 26 | 22 | 17 | 13 | 16 | 18 | 12 | 16 |
| 12740427 | ASPM | asp (abnormal spindle) homolog, microcephaly associated (Drosophila) (ASPM), mRNA. | 8.425149771 | 362 | 383 | 374 | 353 | 144 | 141 | 140 | 24 | 29 | 31 | 23 | 32 | 55 |
| 12722747 | ATAD2 | ATPase family, AAA domain containing 2 (ATAD2), mRNA. | 5.966943872 | 1246 | 1470 | 1298 | 1266 | 461 | 449 | 546 | 139 | 169 | 155 | 149 | 158 | 198 |
| 12887659 | ATOX1 | ATX1 antioxidant protein 1 homolog (yeast) (ATOX1), mRNA. | 2.111332546 | 270 | 334 | 303 | 270 | 309 | 242 | 292 | 115 | 115 | 138 | 152 | 142 | 158 |
| 12909023 | ATP11C | PREDICTED: ATPase, class VI, type 11C (ATP11C), mRNA. | 4.809660165 | 894 | 1072 | 879 | 1251 | 487 | 500 | 392 | 179 | 137 | 169 | 156 | 180 | 155 |
| 12679011 | ATR | ATR Serine/Threonine Kinase | 2.427917934 | 149 | 162 | 133 | 136 | 86 | 74 | 85 | 43 | 46 | 41 | 50 | 51 | 61 |
| 12819106 | ATRNL1 | PREDICTED: attractin-like 1 (ATRNL1), mRNA. | 3.949227972 | 582 | 568 | 711 | 597 | 690 | 890 | 787 | 202 | 133 | 142 | 181 | 202 | 189 |
| 12714015 | AURKA | aurora kinase A (AURKA), mRNA. | 7.029611075 | 433 | 459 | 417 | 420 | 154 | 119 | 175 | 40 | 40 | 40 | 55 | 41 | 48 |
| 12767740 | AURKB | aurora kinase B (AURKB), mRNA. | 5.094885893 | 231 | 242 | 217 | 173 | 84 | 73 | 108 | 28 | 36 | 31 | 39 | 24 | 32 |
| 12815020 | AUTS2 | PREDICTED: autism susceptibility candidate 2 (AUTS2), mRNA. | 2.452795174 | 234 | 254 | 264 | 231 | 235 | 361 | 286 | 96 | 119 | 92 | 105 | 135 | 106 |
| 12737098 | B3GALT2 | UDP-Gal:betaGlcNAc beta 1,3-galactosyltransferase, polypeptide 2 (B3GALT2), mRNA. | 4.251557271 | 330 | 332 | 381 | 276 | 159 | 123 | 145 | 76 | 72 | 59 | 53 | 44 | 48 |
| 12808023 | B4GALT6 | UDP-Gal:betaGlcNAc beta 1,4- galactosyltransferase, polypeptide 6 (B4GALT6), mRNA. | 18.06991905 | 819 | 881 | 637 | 839 | 248 | 165 | 216 | 29 | 32 | 25 | 25 | 27 | 42 |
| 12761690 | B9D1 | B9 protein domain 1 (B9D1), mRNA. | 2.043627626 | 450 | 443 | 318 | 265 | 448 | 281 | 307 | 159 | 155 | 143 | 208 | 184 | 204 |
| 12775474 | BAI2 | Adhesion G Protein-Coupled Receptor B2 | 3.158282798 | 137 | 113 | 151 | 149 | 86 | 78 | 89 | 36 | 38 | 38 | 44 | 39 | 23 |
| 12897969 | BAI3 | Adhesion G Protein-Coupled Receptor B3 | 9.605998434 | 259 | 225 | 420 | 271 | 35 | 30 | 78 | 25 | 16 | 15 | 15 | 25 | 22 |
| 12782631 | BARD1 | BRCA1 associated RING domain 1 (BARD1), mRNA. | 3.817570007 | 347 | 338 | 320 | 324 | 137 | 146 | 156 | 72 | 67 | 54 | 65 | 72 | 68 |
| 12707318 | BCL11A | B-cell CLL/lymphoma 11A (zinc finger protein) (BCL11A), mRNA. | 5.800673467 | 144 | 187 | 115 | 256 | 68 | 43 | 56 | 16 | 22 | 22 | 23 | 20 | 26 |
| 12910364 | BEX2 | brain expressed X-linked 2 (BEX2), mRNA. | 13.81337167 | 70 | 134 | 294 | 214 | 294 | 125 | 267 | 45 | 10 | 5 | 6 | 12 | 9 |
| 12908485 | BEX5 | Brain Expressed, X-Linked 5 | 4.690877049 | 74 | 86 | 145 | 203 | 141 | 156 | 143 | 44 | 37 | 36 | 16 | 20 | 20 |
| 12767844 | BIRC5 | Baculoviral IAP Repeat Containing 5 | 6.233938032 | 518 | 513 | 547 | 365 | 183 | 156 | 191 | 73 | 60 | 45 | 59 | 50 | 53 |
| 12853419 | BLVRA | biliverdin reductase A (BLVRA), mRNA. | 2.412656203 | 1452 | 1387 | 1266 | 1771 | 1169 | 908 | 944 | 573 | 479 | 506 | 633 | 470 | 499 |
| 12755702 | BLVRB | biliverdin reductase B (flavin reductase (NADPH)) (BLVRB), mRNA. | 2.196871619 | 590 | 654 | 827 | 925 | 1207 | 981 | 969 | 450 | 349 | 543 | 364 | 317 | 377 |
| 12825944 | BMPR1A | Bone Morphogenetic Protein Receptor, Type IA | 2.178653858 | 1078 | 1141 | 1016 | 994 | 959 | 1034 | 1065 | 372 | 423 | 335 | 553 | 644 | 540 |
| 12873401 | BMPR1B | bone morphogenetic protein receptor, type IB (BMPR1B), mRNA. | 2.988739183 | 944 | 960 | 1263 | 942 | 710 | 772 | 733 | 495 | 264 | 551 | 211 | 145 | 148 |
| 12767708 | BRCA1 | breast cancer 1, early onset (BRCA1), mRNA. | 4.619943456 | 251 | 268 | 205 | 233 | 86 | 65 | 88 | 35 | 34 | 25 | 46 | 36 | 45 |
| 12711421 | BRCA2 | Breast Cancer 2, Early Onset | 3.934880985 | 256 | 264 | 199 | 237 | 134 | 127 | 160 | 45 | 45 | 31 | 62 | 67 | 50 |
| 12765876 | BRIP1 | BRCA1 Interacting Protein C-Terminal Helicase 1 | 4.576280398 | 164 | 142 | 119 | 111 | 61 | 46 | 58 | 21 | 25 | 28 | 18 | 19 | 19 |
| 12700024 | BUB1 | budding uninhibited by benzimidazoles 1 homolog (yeast) (BUB1), mRNA. | 7.732218419 | 285 | 311 | 277 | 216 | 105 | 84 | 116 | 22 | 28 | 17 | 31 | 23 | 33 |
| 12690910 | BUB1B | budding uninhibited by benzimidazoles 1 homolog beta (yeast) (BUB1B), mRNA. | 13.8058124 | 334 | 418 | 440 | 388 | 158 | 134 | 161 | 23 | 20 | 19 | 19 | 26 | 20 |
| 12688491 | C10H14orf93 | chromosome 14 open reading frame 93 ortholog, mRNA (cDNA clone MGC:134083 IMAGE:8067486), complete cds. | 2.381208252 | 123 | 130 | 113 | 81 | 82 | 53 | 55 | 42 | 39 | 33 | 36 | 36 | 43 |
| 12688633 | C10H15orf23 | chromosome 10 open reading frame, human C15orf23 (C10H15orf23), mRNA. | 2.722237971 | 262 | 276 | 260 | 263 | 128 | 148 | 151 | 78 | 77 | 54 | 88 | 97 | 74 |
| 12819402 | C10orf131 | cdna:known chromosome:UMD3.1:26:17161309:17266254:1 | 4.611744436 | 54 | 117 | 74 | 107 | 118 | 136 | 95 | 25 | 33 | 19 | 19 | 20 | 15 |
| 12726392 | C15H11orf93 | PREDICTED: uncharacterized LOC100294918 (LOC100294918), mRNA. | 7.114179392 | 205 | 239 | 206 | 252 | 73 | 63 | 43 | 19 | 23 | 31 | 15 | 23 | 20 |
| 12737711 | C16H1orf112 | cdna:known chromosome:UMD3.1:16:38250081:38299757:1 | 4.599411182 | 147 | 134 | 91 | 86 | 47 | 41 | 64 | 14 | 21 | 17 | 24 | 21 | 17 |
| 12744739 | C17H4orf46 | chromosome 17 open reading frame, human C4orf46 (C17H4orf46), mRNA. | 3.105622536 | 456 | 499 | 506 | 503 | 325 | 327 | 385 | 153 | 150 | 142 | 137 | 140 | 106 |
| 12763019 | C19H17orf49 | chromosome 19 open reading frame, human C17orf49 (C19H17orf49), mRNA. | 2.116482365 | 826 | 833 | 690 | 486 | 652 | 636 | 547 | 332 | 315 | 266 | 335 | 310 | 333 |
| 12786060 | C20H5orf42 | PREDICTED: chromosome 20 open reading frame, human C5orf42 (C20H5orf42), mRNA. | 2.17224991 | 302 | 308 | 261 | 284 | 223 | 267 | 240 | 116 | 122 | 120 | 104 | 138 | 143 |
| 12790500 | C21H15orf42 | cdna:known chromosome:UMD3.1:21:21436441:21478472:1 | 4.501699476 | 116 | 133 | 110 | 91 | 44 | 37 | 41 | 22 | 19 | 18 | 16 | 16 | 18 |
| 12808600 | C24H18orf54 | chromosome 24 open reading frame, human C18orf54 (C24H18orf54), mRNA. | 3.547510888 | 119 | 147 | 140 | 160 | 70 | 52 | 46 | 29 | 28 | 21 | 37 | 28 | 34 |
| 12818251 | C25H16orf93 | chromosome 25 open reading frame, human C16orf93 (C25H16orf93), mRNA. | 2.118795673 | 136 | 148 | 173 | 127 | 104 | 95 | 87 | 54 | 58 | 50 | 65 | 54 | 70 |
| 12820864 | C26H10orf32 | hypothetical protein LOC613274, mRNA (cDNA clone IMAGE:8167377), partial cds. | 2.304256832 | 162 | 174 | 251 | 206 | 231 | 218 | 277 | 99 | 127 | 163 | 64 | 55 | 57 |
| 12835151 | C29H11orf10 | chromosome 29 open reading frame, human C11orf10 (C29H11orf10), mRNA. | 2.234180795 | 1126 | 1036 | 1098 | 838 | 1346 | 1214 | 1424 | 657 | 554 | 672 | 408 | 356 | 453 |
| 12852258 | C4H7orf55 | chromosome 4 open reading frame, human C7orf55 (C4H7orf55), nuclear gene encoding mitochondrial protein, mRNA. | 2.266821012 | 262 | 262 | 359 | 323 | 257 | 215 | 230 | 115 | 120 | 123 | 133 | 106 | 125 |
| 12872103 | C4orf21 | cdna:known chromosome:UMD3.1:6:14200688:14227106:1 | 2.947781694 | 144 | 154 | 118 | 121 | 66 | 62 | 58 | 33 | 33 | 34 | 30 | 46 | 35 |
| 12865802 | C5H12orf32 | chromosome 5 open reading frame, human C12orf32 (C5H12orf32), mRNA. | 2.197607485 | 232 | 211 | 219 | 134 | 172 | 143 | 144 | 89 | 85 | 73 | 89 | 81 | 73 |
| 12856580 | C5H12orf4 | chromosome 12 open reading frame 4 ortholog, mRNA (cDNA clone MGC:139467 IMAGE:8214316), complete cds. | 2.419609441 | 341 | 373 | 371 | 336 | 225 | 246 | 254 | 135 | 138 | 141 | 114 | 123 | 109 |
| 12693316 | C5orf25 | cdna:novel chromosome:UMD3.1:10:4979152:5011240:-1 | 2.584744414 | 320 | 346 | 369 | 333 | 225 | 205 | 219 | 109 | 120 | 100 | 123 | 122 | 93 |
| 12895648 | C8H9orf100 | chromosome 8 open reading frame, human C9orf100 (C8H9orf100), mRNA. | 3.778882978 | 174 | 159 | 143 | 135 | 72 | 60 | 70 | 34 | 29 | 29 | 32 | 35 | 26 |
| 12908260 | CA5B | carbonic anhydrase VB, mitochondrial (CA5B), nuclear gene encoding mitochondrial protein, mRNA. | 3.725996316 | 183 | 189 | 318 | 184 | 95 | 73 | 82 | 55 | 39 | 37 | 50 | 39 | 39 |
| 12692627 | CASC5 | PREDICTED: cancer susceptibility candidate 5 (CASC5), mRNA. | 12.14045406 | 339 | 432 | 304 | 387 | 119 | 103 | 94 | 18 | 26 | 23 | 18 | 23 | 18 |
| 12805112 | CCDC167 | coiled-coil domain containing 167 (CCDC167), mRNA. | 2.344235715 | 237 | 241 | 243 | 295 | 202 | 158 | 181 | 102 | 98 | 132 | 75 | 62 | 100 |
| 12710996 | CCDC168 | Coiled-Coil Domain Containing 168 | 3.910082937 | 131 | 119 | 104 | 71 | 48 | 55 | 76 | 22 | 22 | 17 | 22 | 24 | 26 |
| 12847098 | CCDC18 | PREDICTED: coiled-coil domain containing 18 (CCDC18), mRNA. | 4.525052861 | 194 | 255 | 178 | 175 | 79 | 62 | 75 | 25 | 32 | 25 | 37 | 32 | 40 |
| 12714635 | CCDC3 | coiled-coil domain containing 3 (CCDC3), mRNA. | 3.147703691 | 95 | 137 | 95 | 119 | 81 | 76 | 52 | 27 | 33 | 33 | 31 | 33 | 22 |
| 12800467 | CCDC72 | Coiled-Coil Domain Containing 72 | 2.324400343 | 1543 | 1539 | 1340 | 1696 | 1620 | 1532 | 1807 | 637 | 649 | 654 | 738 | 684 | 724 |
| 12753878 | CCDC8 | coiled-coil domain containing 8 (CCDC8), mRNA. | 2.343712824 | 155 | 185 | 192 | 220 | 267 | 213 | 215 | 50 | 61 | 52 | 120 | 165 | 81 |
| 12785624 | CCDC99 | Coiled-Coil Domain Containing 99 | 6.340295398 | 134 | 118 | 147 | 139 | 44 | 35 | 53 | 15 | 17 | 17 | 11 | 17 | 13 |
| 12869943 | CCNA2 | cyclin A2 (CCNA2), mRNA. | 11.79034248 | 619 | 620 | 533 | 576 | 204 | 139 | 165 | 60 | 35 | 23 | 26 | 28 | 35 |
| 12787030 | CCNB1 | cyclin B1 (CCNB1), mRNA. | 4.306755007 | 286 | 258 | 289 | 182 | 124 | 131 | 150 | 28 | 49 | 53 | 44 | 69 | 39 |
| 12868434 | CCND2 | cyclin D2 (CCND2), mRNA. | 9.923127995 | 1892 | 1935 | 2096 | 2781 | 796 | 616 | 685 | 256 | 227 | 225 | 77 | 78 | 70 |
| 12811770 | CCNF | cyclin F (CCNF), mRNA. | 2.475679151 | 113 | 111 | 124 | 86 | 63 | 60 | 63 | 35 | 37 | 29 | 42 | 40 | 31 |
| 12847375 | CDC14A | Cell Division Cycle 14A | 2.859324851 | 764 | 733 | 673 | 760 | 348 | 305 | 292 | 193 | 224 | 220 | 171 | 188 | 166 |
| 12894083 | CDC14B | CDC14 cell division cycle 14 homolog B (S. cerevisiae) (CDC14B), mRNA. | 3.661943893 | 1004 | 1186 | 1293 | 1233 | 1057 | 889 | 1123 | 260 | 273 | 295 | 307 | 325 | 361 |
| 12844886 | CDC20 | cell division cycle 20 homolog (S. cerevisiae) (CDC20), mRNA. | 11.06070151 | 468 | 471 | 513 | 471 | 210 | 165 | 198 | 37 | 34 | 36 | 31 | 31 | 25 |
| 12718493 | CDC25B | cell division cycle 25 homolog B (S. pombe) (CDC25B), mRNA. | 4.424707052 | 320 | 345 | 355 | 262 | 130 | 132 | 124 | 52 | 75 | 44 | 55 | 61 | 36 |
| 12884149 | CDC25C | cell division cycle 25 homolog C (S. pombe) (CDC25C), mRNA. | 3.006696709 | 96 | 121 | 110 | 106 | 52 | 43 | 51 | 32 | 25 | 21 | 28 | 28 | 29 |
| 12763375 | CDC6 | cell division cycle 6 homolog (S. cerevisiae) (CDC6), mRNA. | 4.96484589 | 210 | 205 | 157 | 140 | 81 | 52 | 67 | 16 | 36 | 23 | 32 | 21 | 29 |
| 12844935 | CDC7 | cell division cycle 7 homolog (S. cerevisiae) (CDC7), mRNA. | 2.060301762 | 377 | 381 | 328 | 484 | 310 | 375 | 345 | 159 | 189 | 163 | 186 | 174 | 211 |
| 12862285 | CDCA3 | cell division cycle associated 3 (CDCA3), mRNA. | 2.759595913 | 162 | 168 | 138 | 118 | 98 | 77 | 81 | 42 | 33 | 54 | 43 | 40 | 49 |
| 12854135 | CDCA7L | Cell Division Cycle Associated 7-Like | 4.136287862 | 477 | 464 | 308 | 277 | 259 | 255 | 293 | 109 | 81 | 86 | 70 | 80 | 57 |
| 12843787 | CDCA8 | cell division cycle associated 8 (CDCA8), mRNA. | 7.31477293 | 143 | 150 | 165 | 132 | 56 | 55 | 51 | 17 | 13 | 18 | 15 | 13 | 13 |
| 12786403 | CDH12 | cadherin 12, type 2 (N-cadherin 2) (CDH12), mRNA. | 87.62871155 | 1464 | 1508 | 1597 | 1217 | 526 | 540 | 507 | 27 | 10 | 9 | 11 | 7 | 8 |
| 12690735 | CDH24 | Cadherin 24, Type 2 | 3.993379275 | 364 | 321 | 228 | 284 | 197 | 125 | 150 | 61 | 49 | 70 | 70 | 53 | 56 |
| 12825838 | CDK1 | Cyclin-Dependent Kinase 1 | 8.316230446 | 231 | 277 | 226 | 215 | 92 | 60 | 57 | 29 | 22 | 16 | 15 | 17 | 21 |
| 12866416 | CDK2 | cyclin-dependent kinase 2 (CDK2), mRNA. | 2.978459835 | 316 | 273 | 256 | 182 | 126 | 121 | 129 | 76 | 77 | 75 | 55 | 60 | 61 |
| 12744453 | CDK2AP1 | cyclin-dependent kinase 2 associated protein 1 (CDK2AP1), mRNA. | 2.014875928 | 375 | 376 | 316 | 380 | 464 | 413 | 360 | 222 | 206 | 200 | 193 | 190 | 131 |
| 12853903 | CDK6 | cyclin-dependent kinase 6 (CDK6), mRNA. | 2.711537861 | 380 | 414 | 516 | 385 | 233 | 175 | 190 | 130 | 143 | 147 | 101 | 113 | 92 |
| 12884993 | CDKN2AIPNL | CDKN2A interacting protein N-terminal like (CDKN2AIPNL), mRNA. | 2.177019977 | 275 | 319 | 288 | 469 | 303 | 302 | 282 | 139 | 142 | 154 | 150 | 135 | 160 |
| 12691926 | CDKN3 | cyclin-dependent kinase inhibitor 3 (CDKN3), mRNA. | 10.10509447 | 399 | 461 | 384 | 329 | 113 | 104 | 117 | 27 | 26 | 25 | 26 | 26 | 32 |
| 12707854 | CENPA | centromere protein A (CENPA), mRNA. | 29.43681882 | 511 | 479 | 601 | 505 | 181 | 110 | 115 | 8 | 17 | 12 | 15 | 7 | 12 |
| 12869954 | CENPE | Centromere Protein E, 312kDa | 17.51732297 | 419 | 462 | 427 | 430 | 129 | 101 | 127 | 17 | 19 | 11 | 16 | 21 | 18 |
| 12740194 | CENPF | centromere protein F, 350/400kDa (mitosin) (CENPF), mRNA. | 17.7315992 | 390 | 388 | 391 | 377 | 117 | 122 | 139 | 20 | 17 | 15 | 15 | 15 | 12 |
| 12903846 | CENPI | centromere protein I (CENPI), mRNA. | 2.729507307 | 161 | 160 | 155 | 154 | 77 | 65 | 82 | 40 | 49 | 40 | 46 | 50 | 44 |
| 12785235 | CENPK | centromere protein K (CENPK), mRNA. | 13.02136748 | 202 | 194 | 204 | 167 | 59 | 40 | 37 | 13 | 9 | 9 | 11 | 10 | 8 |
| 12741130 | CENPL | centromere protein L (CENPL), mRNA. | 2.377536869 | 231 | 212 | 198 | 207 | 122 | 94 | 121 | 72 | 71 | 61 | 80 | 72 | 72 |
| 12751437 | CENPN | centromere protein N (CENPN), mRNA. | 4.02942199 | 444 | 402 | 405 | 384 | 157 | 115 | 176 | 52 | 50 | 72 | 98 | 57 | 114 |
| 12708398 | CENPO | centromere protein O (CENPO), mRNA. | 3.520404613 | 347 | 334 | 337 | 299 | 172 | 97 | 162 | 78 | 68 | 65 | 79 | 68 | 68 |
| 12889307 | CENPP | Centromere Protein P | 4.408757636 | 162 | 162 | 130 | 138 | 58 | 49 | 48 | 29 | 33 | 17 | 24 | 15 | 28 |
| 12757103 | CENPT | Centromere Protein T | 2.993280099 | 200 | 179 | 165 | 118 | 110 | 93 | 81 | 37 | 33 | 48 | 46 | 54 | 53 |
| 12899402 | CENPW | centromere protein W (CENPW), mRNA. | 4.253534919 | 296 | 258 | 269 | 196 | 128 | 78 | 78 | 41 | 42 | 38 | 62 | 42 | 38 |
| 12890056 | CEP44 | centrosomal protein 44kDa (CEP44), mRNA. | 3.220282278 | 101 | 119 | 110 | 115 | 46 | 40 | 44 | 25 | 24 | 20 | 27 | 32 | 25 |
| 12830605 | CHEK1 | CHK1 checkpoint homolog (S. pombe) (CHEK1), mRNA. | 6.091467252 | 219 | 217 | 188 | 160 | 63 | 50 | 77 | 21 | 22 | 24 | 36 | 18 | 15 |
| 12853629 | CHN2 | chimerin (chimaerin) 2 (CHN2), mRNA. | 6.644057522 | 548 | 625 | 576 | 387 | 268 | 239 | 197 | 61 | 81 | 80 | 28 | 61 | 55 |
| 12783792 | CHPF | chondroitin polymerizing factor (CHPF), mRNA. | 3.686197278 | 696 | 680 | 583 | 653 | 547 | 388 | 464 | 145 | 124 | 142 | 182 | 166 | 173 |
| 12791755 | CHRNA3 | Cholinergic Receptor, Nicotinic, Alpha 3 (Neuronal) | 11.42462162 | 22 | 25 | 48 | 29 | 244 | 376 | 273 | 11 | 17 | 12 | 13 | 11 | 13 |
| 12705319 | CHST10 | carbohydrate sulfotransferase 10 (CHST10), mRNA. | 2.071209417 | 266 | 275 | 209 | 417 | 274 | 233 | 238 | 132 | 124 | 95 | 166 | 152 | 122 |
| 12860640 | CHST11 | carbohydrate (chondroitin 4) sulfotransferase 11 (CHST11), mRNA. | 19.94131872 | 3065 | 3157 | 3078 | 4248 | 1508 | 1035 | 981 | 153 | 163 | 134 | 76 | 100 | 107 |
| 12688963 | CHST14 | carbohydrate (N-acetylgalactosamine 4-0) sulfotransferase 14 (CHST14), mRNA. | 2.125691901 | 241 | 202 | 220 | 214 | 178 | 199 | 145 | 102 | 98 | 88 | 87 | 90 | 100 |
| 12748838 | CHST8 | carbohydrate (N-acetylgalactosamine 4-0) sulfotransferase 8 (CHST8), mRNA. | 27.45839228 | 2172 | 2325 | 1812 | 1880 | 873 | 555 | 490 | 63 | 44 | 50 | 73 | 34 | 50 |
| 12789026 | CHSY1 | chondroitin sulfate synthase 1 (CHSY1), mRNA. | 4.594220782 | 576 | 607 | 589 | 882 | 468 | 303 | 337 | 129 | 115 | 125 | 125 | 93 | 115 |
| 12744882 | CIT | citron (rho-interacting, serine/threonine kinase 21), mRNA (cDNA clone IMAGE:8280364), partial cds. | 9.324208933 | 460 | 443 | 449 | 345 | 230 | 198 | 246 | 42 | 35 | 35 | 34 | 29 | 43 |
| 12711817 | CKAP2 | cytoskeleton associated protein 2 (CKAP2), mRNA. | 6.838604243 | 851 | 902 | 952 | 702 | 313 | 407 | 425 | 112 | 119 | 83 | 84 | 101 | 71 |
| 12704554 | CKAP2L | cytoskeleton associated protein 2-like (CKAP2L), mRNA. | 10.32077964 | 521 | 505 | 500 | 461 | 134 | 129 | 149 | 27 | 29 | 35 | 33 | 42 | 33 |
| 12732760 | CKAP5 | cytoskeleton associated protein 5 (CKAP5), mRNA. | 2.413806563 | 662 | 700 | 761 | 621 | 381 | 393 | 404 | 229 | 251 | 233 | 242 | 219 | 218 |
| 12895589 | CKS2 | CDC28 Protein Kinase Regulatory Subunit 2 | 3.532566203 | 321 | 324 | 321 | 308 | 161 | 120 | 176 | 63 | 64 | 59 | 83 | 85 | 65 |
| 12802146 | CLIC1 | chloride intracellular channel 1 (CLIC1), mRNA. | 2.277190157 | 595 | 613 | 688 | 817 | 497 | 435 | 432 | 296 | 289 | 338 | 249 | 175 | 187 |
| 12757931 | CLIP3 | CAP-GLY domain containing linker protein 3 (CLIP3), mRNA. | 2.135344036 | 232 | 222 | 260 | 203 | 174 | 199 | 139 | 125 | 114 | 90 | 73 | 92 | 79 |
| 12785671 | CMBL | carboxymethylenebutenolidase homolog (Pseudomonas) (CMBL), mRNA. | 3.938017455 | 576 | 617 | 457 | 664 | 361 | 197 | 186 | 90 | 94 | 101 | 150 | 106 | 125 |
| 12754736 | CMTM3 | CKLF-like MARVEL transmembrane domain containing 3 (CMTM3), mRNA. | 2.259215731 | 552 | 556 | 652 | 479 | 822 | 677 | 583 | 322 | 278 | 178 | 288 | 321 | 253 |
| 12737300 | CNIH3 | cornichon homolog 3 (Drosophila) (CNIH3), mRNA. | 35.5023849 | 692 | 748 | 828 | 973 | 630 | 501 | 500 | 23 | 15 | 23 | 21 | 21 | 15 |
| 12796803 | CNOT10 | CCR4-NOT transcription complex, subunit 10 (CNOT10), mRNA. | 2.107569225 | 148 | 153 | 167 | 211 | 120 | 115 | 122 | 53 | 71 | 54 | 65 | 85 | 94 |
| 12886832 | CNOT6 | CCR4-NOT transcription complex, subunit 6 (CNOT6), mRNA. | 2.575611464 | 687 | 802 | 858 | 1098 | 533 | 429 | 460 | 247 | 255 | 234 | 288 | 275 | 320 |
| 12707497 | CNRIP1 | cannabinoid receptor interacting protein 1 (CNRIP1), mRNA. | 2.839898333 | 414 | 449 | 322 | 540 | 367 | 417 | 389 | 146 | 159 | 115 | 142 | 177 | 137 |
| 12802836 | COL21A1 | collagen, type XXI, alpha 1, mRNA (cDNA clone MGC:155235 IMAGE:8142934), complete cds. | 16.56475082 | 498 | 593 | 389 | 781 | 313 | 332 | 260 | 22 | 18 | 14 | 25 | 50 | 34 |
| 12774697 | COL4A3 | collagen, type IV, alpha 3 (Goodpasture antigen) (COL4A3), mRNA. | 2.770561138 | 87 | 95 | 107 | 100 | 189 | 160 | 116 | 43 | 30 | 31 | 33 | 87 | 40 |
| 12873520 | COQ2 | Coenzyme Q2 4-Hydroxybenzoate Polyprenyltransferase | 2.002277227 | 692 | 655 | 862 | 616 | 640 | 660 | 948 | 345 | 262 | 362 | 421 | 367 | 414 |
| 12703862 | CPSF3 | Cleavage And Polyadenylation Specific Factor 3, 73kDa | 2.066587896 | 426 | 434 | 437 | 503 | 258 | 277 | 308 | 148 | 167 | 175 | 221 | 173 | 212 |
| 12815150 | CPSF4 | cleavage and polyadenylation specific factor 4, 30kDa (CPSF4), mRNA. | 2.173505158 | 351 | 325 | 306 | 372 | 298 | 223 | 259 | 106 | 139 | 125 | 152 | 161 | 159 |
| 12868913 | CPSF6 | cleavage and polyadenylation specific factor 6, 68kDa (CPSF6), mRNA. | 2.0673307 | 650 | 643 | 651 | 601 | 500 | 535 | 442 | 220 | 255 | 239 | 303 | 346 | 305 |
| 12783910 | CSRNP3 | cysteine-serine-rich nuclear protein 3 (CSRNP3), mRNA. | 7.76962111 | 362 | 371 | 510 | 565 | 250 | 194 | 189 | 54 | 45 | 47 | 40 | 60 | 24 |
| 12769014 | CTDNEP1 | CTD nuclear envelope phosphatase 1 (CTDNEP1), mRNA. | 2.000542863 | 605 | 547 | 604 | 609 | 570 | 625 | 535 | 262 | 270 | 247 | 358 | 316 | 302 |
| 12691258 | CTDSPL2 | CTD (carboxy-terminal domain, RNA polymerase II, polypeptide A) small phosphatase like 2 (CTDSPL2), mRNA. | 2.097006359 | 635 | 683 | 696 | 847 | 387 | 414 | 413 | 287 | 271 | 278 | 261 | 272 | 295 |
| 12706464 | CTNNA2 | catenin (cadherin-associated protein), alpha 2 (CTNNA2), mRNA. | 5.454091618 | 104 | 159 | 98 | 183 | 64 | 40 | 38 | 26 | 12 | 21 | 15 | 16 | 19 |
| 12744597 | CTSO | Cathepsin O | 4.097216208 | 275 | 230 | 221 | 225 | 208 | 195 | 149 | 51 | 50 | 56 | 52 | 52 | 54 |
| 12910294 | CXHXorf22 | PREDICTED: chromosome X open reading frame, human CXorf22 (CXHXorf22), mRNA. | 8.244378291 | 224 | 248 | 333 | 266 | 61 | 86 | 97 | 17 | 17 | 22 | 26 | 31 | 23 |
| 12910004 | CXHXorf30 | PREDICTED: chromosome X open reading frame, human CXorf30 (CXHXorf30), mRNA. | 5.683383627 | 142 | 138 | 162 | 141 | 36 | 52 | 45 | 13 | 14 | 12 | 25 | 25 | 18 |
| 12688063 | CYP19A1 | cytochrome P450, family 19, subfamily A, polypeptide 1 (CYP19A1), mRNA. | 95.11976933 | 6580 | 6627 | 7000 | 6757 | 4209 | 4086 | 4747 | 107 | 83 | 126 | 14 | 15 | 15 |
| 12886857 | CYP4F3 | cytochrome P450, family 4, subfamily F, polypeptide 3 (CYP4F3), mRNA. | 9.879735327 | 88 | 119 | 65 | 123 | 552 | 279 | 618 | 36 | 21 | 23 | 27 | 29 | 24 |
| 12712565 | DACH1 | Dachshund Family Transcription Factor 1 | 5.053023866 | 665 | 765 | 627 | 650 | 242 | 178 | 230 | 143 | 127 | 150 | 45 | 66 | 39 |
| 12891606 | DAPK1 | death-associated protein kinase 1 (DAPK1), mRNA. | 6.484532111 | 313 | 335 | 372 | 348 | 224 | 175 | 148 | 44 | 48 | 44 | 35 | 42 | 40 |
| 12853829 | DBF4 | DBF4 homolog (S. cerevisiae) (DBF4), mRNA. | 4.286971648 | 212 | 207 | 193 | 200 | 82 | 63 | 83 | 41 | 35 | 31 | 31 | 30 | 39 |
| 12875995 | DCAF16 | DDB1 And CUL4 Associated Factor 16 | 2.069249303 | 384 | 439 | 382 | 376 | 299 | 361 | 348 | 155 | 146 | 138 | 177 | 228 | 229 |
| 12853963 | DENND2A | DENN/MADD Domain Containing 2A | 2.817042746 | 264 | 257 | 233 | 267 | 190 | 186 | 170 | 95 | 123 | 69 | 69 | 70 | 51 |
| 12839301 | DEPDC1 | PREDICTED: DEP domain containing 1 (DEPDC1), mRNA. | 20.33178834 | 442 | 458 | 461 | 445 | 126 | 75 | 102 | 16 | 13 | 10 | 24 | 15 | 10 |
| 12786454 | DEPDC1B | DEP domain containing 1B (DEPDC1B), mRNA. | 6.637548314 | 124 | 120 | 143 | 106 | 37 | 36 | 40 | 10 | 12 | 16 | 11 | 11 | 18 |
| 12864719 | DGKA | diacylglycerol kinase, alpha 80kDa (DGKA), mRNA. | 2.059141054 | 176 | 220 | 264 | 136 | 216 | 190 | 170 | 150 | 91 | 91 | 89 | 66 | 83 |
| 12884439 | DHFR | dihydrofolate reductase (DHFR), mRNA. | 3.74040994 | 411 | 451 | 348 | 275 | 180 | 202 | 221 | 81 | 71 | 72 | 114 | 69 | 71 |
| 12709849 | DIAPH3 | diaphanous homolog 3 (Drosophila) (DIAPH3), mRNA. | 5.953267657 | 151 | 164 | 152 | 150 | 72 | 54 | 54 | 18 | 18 | 20 | 22 | 20 | 17 |
| 12694290 | DLGAP5 | discs, large (Drosophila) homolog-associated protein 5 (DLGAP5), mRNA. | 11.02784377 | 424 | 423 | 425 | 385 | 141 | 107 | 118 | 26 | 38 | 17 | 34 | 21 | 21 |
| 12828817 | DNA2 | DNA replication helicase 2 homolog (yeast) (DNA2), mRNA. | 5.087616463 | 137 | 148 | 106 | 105 | 39 | 38 | 39 | 17 | 19 | 18 | 18 | 13 | 18 |
| 12891218 | DNAJC25 | DnaJ (Hsp40) homolog, subfamily C , member 25 (DNAJC25), mRNA. | 5.141038943 | 913 | 953 | 837 | 1141 | 318 | 216 | 296 | 151 | 135 | 133 | 126 | 110 | 124 |
| 12827978 | DNAJC9 | DnaJ (Hsp40) homolog, subfamily C, member 9 (DNAJC9), mRNA. | 3.85691003 | 325 | 342 | 292 | 331 | 117 | 106 | 116 | 50 | 55 | 61 | 64 | 58 | 75 |
| 12883086 | DNMT1 | DNA (cytosine-5-)-methyltransferase 1 (DNMT1), mRNA. | 2.609492634 | 511 | 540 | 431 | 349 | 220 | 201 | 214 | 132 | 144 | 120 | 123 | 147 | 145 |
| 12889361 | DOCK5 | PREDICTED: dedicator of cytokinesis 5 (DOCK5), mRNA. | 4.624367326 | 598 | 705 | 557 | 541 | 273 | 199 | 196 | 98 | 70 | 90 | 100 | 125 | 86 |
| 12682695 | DYNLT1 | dynein, light chain, Tctex-type 1 (DYNLT1), mRNA. | 3.030109644 | 855 | 1031 | 750 | 706 | 527 | 572 | 624 | 266 | 224 | 267 | 272 | 209 | 196 |
| 12681678 | DZIP3 | DAZ interacting protein 3, zinc finger (DZIP3), mRNA. | 2.419291632 | 211 | 266 | 320 | 263 | 229 | 258 | 231 | 74 | 85 | 67 | 117 | 141 | 145 |
| 12832191 | E2F8 | E2F transcription factor 8 (E2F8), mRNA. | 4.325146451 | 193 | 180 | 142 | 164 | 48 | 45 | 56 | 28 | 22 | 29 | 37 | 23 | 25 |
| 12683782 | ECT2 | epithelial cell transforming sequence 2 oncogene (ECT2), mRNA. | 4.401108358 | 609 | 635 | 600 | 581 | 211 | 156 | 185 | 78 | 78 | 70 | 113 | 120 | 120 |
| 12887066 | EDIL3 | EGF-Like Repeats And Discoidin I-Like Domains 3 | 13.79012718 | 553 | 735 | 384 | 1287 | 289 | 211 | 229 | 78 | 28 | 61 | 19 | 27 | 16 |
| 12876696 | EFNA5 | ephrin-A5 (EFNA5), mRNA. | 8.869075962 | 3468 | 2781 | 4651 | 4359 | 786 | 951 | 745 | 327 | 439 | 404 | 167 | 215 | 163 |
| 12798175 | EGFR | isolate HNBTA1007153 epidermal growth factor receptor (EGFR) mRNA, complete cds. | 3.284500768 | 690 | 652 | 560 | 647 | 551 | 555 | 432 | 189 | 167 | 201 | 184 | 167 | 158 |
| 12751758 | EIF3K | eukaryotic translation initiation factor 3, subunit K (EIF3K), mRNA. | 2.141614651 | 849 | 896 | 714 | 572 | 1297 | 1486 | 1219 | 445 | 422 | 454 | 482 | 494 | 518 |
| 12696285 | EML5 | echinoderm microtubule associated protein like 5 (EML5), mRNA. | 4.242521564 | 343 | 341 | 425 | 354 | 238 | 230 | 222 | 49 | 53 | 58 | 73 | 96 | 106 |
| 12696017 | ENTPD5 | ectonucleoside triphosphate diphosphohydrolase 5 (ENTPD5), mRNA. | 2.584270461 | 166 | 170 | 169 | 177 | 131 | 133 | 124 | 69 | 52 | 80 | 53 | 51 | 50 |
| 12873095 | EPHA5 | PREDICTED: ePH receptor A5-like (LOC100337226), mRNA. | 8.625697464 | 873 | 851 | 818 | 1003 | 316 | 327 | 280 | 104 | 70 | 110 | 63 | 55 | 43 |
| 12796560 | ERC2 | ELKS/RAB6-Interacting/CAST Family Member 2 | 7.083452313 | 188 | 235 | 231 | 233 | 75 | 59 | 37 | 28 | 21 | 30 | 13 | 20 | 15 |
| 12906051 | ERCC6L | excision repair cross-complementing rodent repair deficiency, complementation group 6-like (ERCC6L), mRNA. | 3.44303101 | 164 | 176 | 154 | 124 | 71 | 51 | 69 | 46 | 28 | 27 | 36 | 26 | 39 |
| 12894921 | ESCO2 | establishment of cohesion 1 homolog 2 (S. cerevisiae) (ESCO2), mRNA. | 8.976406959 | 501 | 565 | 430 | 390 | 124 | 84 | 115 | 44 | 35 | 36 | 37 | 26 | 33 |
| 12864825 | ESPL1 | extra spindle pole bodies homolog 1 (S. cerevisiae) (ESPL1), mRNA. | 3.971874718 | 174 | 173 | 192 | 123 | 86 | 81 | 87 | 28 | 34 | 51 | 39 | 25 | 20 |
| 12693406 | ESR2 | estrogen receptor 2 (ER beta) (ESR2), mRNA. | 21.08861338 | 713 | 757 | 380 | 625 | 214 | 130 | 130 | 21 | 15 | 18 | 20 | 27 | 19 |
| 12740236 | ETNK2 | PREDICTED: ethanolamine kinase 2 (ETNK2), mRNA. | 3.762936569 | 959 | 1140 | 1277 | 1562 | 1295 | 1105 | 1027 | 313 | 240 | 308 | 334 | 398 | 312 |
| 12738902 | EXO1 | PREDICTED: exonuclease 1 (EXO1), mRNA. | 4.815757761 | 123 | 118 | 88 | 103 | 42 | 24 | 37 | 19 | 17 | 16 | 11 | 15 | 17 |
| 12736803 | EXOSC10 | exosome component 10 (EXOSC10), mRNA. | 2.742388127 | 1825 | 1972 | 1986 | 1648 | 1301 | 1237 | 1165 | 479 | 484 | 377 | 610 | 921 | 608 |
| 12853746 | EZH2 | enhancer of zeste homolog 2 (Drosophila) (EZH2), mRNA. | 3.413024512 | 548 | 562 | 410 | 397 | 169 | 183 | 176 | 92 | 135 | 140 | 78 | 78 | 92 |
| 12839662 | FAF1 | Fas (TNFRSF6) associated factor 1 (FAF1), mRNA. | 2.217109745 | 498 | 542 | 500 | 623 | 345 | 374 | 351 | 174 | 184 | 170 | 218 | 263 | 241 |
| 12870843 | FAM114A1 | PREDICTED: family with sequence similarity 114, member A1 (FAM114A1), mRNA. | 2.363982223 | 1953 | 2057 | 2123 | 2141 | 1756 | 1721 | 1804 | 604 | 548 | 460 | 1153 | 1193 | 955 |
| 12823910 | FAM149A | Family With Sequence Similarity 149, Member A | 2.67260146 | 140 | 155 | 138 | 230 | 142 | 132 | 103 | 73 | 53 | 42 | 79 | 30 | 57 |
| 12784152 | FAM171B | family with sequence similarity 171, member B (FAM171B), mRNA. | 13.22832374 | 922 | 925 | 826 | 1105 | 324 | 281 | 347 | 91 | 59 | 90 | 20 | 22 | 26 |
| 12720625 | FAM188A | family with sequence similarity 188, member A (FAM188A), mRNA. | 3.164111458 | 1792 | 1916 | 1497 | 1891 | 713 | 664 | 758 | 462 | 446 | 488 | 394 | 383 | 327 |
| 12900926 | FAM54A | family with sequence similarity 54, member A (FAM54A), mRNA. | 6.631025236 | 500 | 480 | 410 | 324 | 178 | 120 | 114 | 39 | 52 | 51 | 42 | 43 | 47 |
| 12772448 | FAM64A | family with sequence similarity 64, member A (FAM64A), mRNA. | 3.713477373 | 155 | 152 | 186 | 95 | 78 | 67 | 63 | 24 | 33 | 36 | 24 | 32 | 34 |
| 12704512 | FAM78A | chromosome 9 open reading frame 59 (C9orf59), mRNA, complete cds. | 13.86461981 | 2152 | 2036 | 1663 | 2470 | 1063 | 789 | 644 | 85 | 144 | 71 | 141 | 119 | 109 |
| 12759146 | FAM96B | family with sequence similarity 96, member B (FAM96B), mRNA. | 2.58563758 | 716 | 762 | 711 | 544 | 416 | 310 | 372 | 241 | 182 | 250 | 192 | 195 | 211 |
| 12790238 | FANCI | Fanconi anemia, complementation group I (FANCI), mRNA. | 6.545405099 | 411 | 370 | 378 | 296 | 123 | 118 | 134 | 57 | 46 | 34 | 35 | 38 | 30 |
| 12900227 | FBXO5 | F-box protein 5 (FBXO5), mRNA. | 16.00295057 | 588 | 545 | 488 | 457 | 123 | 116 | 136 | 23 | 28 | 14 | 26 | 18 | 22 |
| 12890898 | FBXW12 | F-box and WD repeat domain containing 12 (FBXW12), mRNA. | 2.04794871 | 216 | 255 | 249 | 190 | 336 | 359 | 284 | 116 | 127 | 94 | 141 | 137 | 176 |
| 12842770 | FCER1G | Fc fragment of IgE, high affinity I, receptor for; gamma polypeptide (FCER1G), mRNA. | 2.337137735 | 71 | 72 | 106 | 71 | 73 | 102 | 141 | 36 | 45 | 44 | 32 | 40 | 37 |
| 12720176 | FLRT3 | fibronectin leucine rich transmembrane protein 3 (FLRT3), mRNA. | 12.65435031 | 455 | 516 | 368 | 527 | 174 | 159 | 255 | 42 | 33 | 28 | 19 | 21 | 23 |
| 12686845 | FNDC3B | fibronectin type III domain containing 3B (FNDC3B), mRNA. | 2.222060985 | 5622 | 5995 | 5510 | 5083 | 3668 | 3485 | 2894 | 1969 | 1740 | 1534 | 2482 | 2499 | 2218 |
| 12678679 | FOXL2 | forkhead box L2 (FOXL2), mRNA. | 3.49599944 | 912 | 872 | 784 | 554 | 526 | 407 | 421 | 284 | 138 | 258 | 165 | 129 | 123 |
| 12859343 | FOXM1 | PREDICTED: forkhead box M1, transcript variant 1 (FOXM1), mRNA. | 2.377097197 | 198 | 144 | 171 | 118 | 119 | 100 | 93 | 64 | 59 | 68 | 55 | 51 | 43 |
| 12853453 | FOXP2 | forkhead box P2 (FOXP2), mRNA. | 3.005417714 | 549 | 556 | 686 | 659 | 717 | 832 | 743 | 156 | 189 | 126 | 253 | 298 | 329 |
| 12703820 | FSHR | follicle stimulating hormone receptor (FSHR), mRNA. | 35.38408587 | 1327 | 1800 | 1175 | 2205 | 497 | 513 | 427 | 68 | 25 | 49 | 19 | 15 | 16 |
| 12787048 | FST | follistatin (FST), mRNA. | 6.94844564 | 3796 | 4208 | 3850 | 4626 | 3026 | 1787 | 2328 | 922 | 575 | 1199 | 91 | 64 | 62 |
| 12849474 | FZD1 | frizzled family receptor 1 (FZD1), mRNA. | 2.641583232 | 334 | 321 | 400 | 329 | 335 | 366 | 423 | 121 | 113 | 101 | 150 | 166 | 162 |
| 12789584 | G2E3 | G2/M-phase specific E3 ubiquitin ligase, mRNA (cDNA clone MGC:133923 IMAGE:8055575), complete cds. | 2.779166255 | 492 | 519 | 522 | 495 | 295 | 246 | 240 | 117 | 136 | 140 | 145 | 160 | 169 |
| 12890675 | GALNT7 | PREDICTED: UDP-N-acetyl-alpha-D-galactosamine:polypeptide N-acetylgalactosaminyltransferase 7 (GalNAc-T7) (GALNT7), mRNA. | 4.802547998 | 769 | 842 | 929 | 777 | 435 | 380 | 436 | 184 | 188 | 137 | 112 | 117 | 76 |
| 12862938 | GAS2L3 | PREDICTED: growth arrest-specific 2 like 3 (GAS2L3), mRNA. | 6.091270032 | 331 | 404 | 375 | 280 | 160 | 141 | 148 | 46 | 51 | 40 | 46 | 36 | 39 |
| 12810695 | GATA6 | GATA-6 mRNA, partial cds. | 2.530438918 | 2832 | 2896 | 3614 | 3390 | 2148 | 1965 | 2127 | 812 | 898 | 728 | 1170 | 1487 | 1331 |
| 12801746 | GCLC | glutamate-cysteine ligase, catalytic subunit (GCLC), mRNA. | 3.729408733 | 3814 | 3940 | 3768 | 3945 | 2026 | 1774 | 1486 | 658 | 614 | 887 | 732 | 1068 | 810 |
| 12708565 | GEN1 | Gen homolog 1, endonuclease (Drosophila) (GEN1), mRNA. | 7.500082833 | 233 | 234 | 170 | 157 | 87 | 63 | 71 | 14 | 21 | 13 | 24 | 21 | 23 |
| 12715654 | GINS1 | GINS complex subunit 1 (Psf1 homolog) (GINS1), mRNA. | 4.526975448 | 416 | 431 | 380 | 316 | 176 | 138 | 167 | 71 | 68 | 49 | 70 | 66 | 60 |
| 12899452 | GJA1 | gap junction protein, alpha 1, 43kDa (GJA1), mRNA. | 3.360585988 | 4373 | 4971 | 4289 | 5124 | 3621 | 3450 | 4229 | 1307 | 1316 | 1171 | 1069 | 1473 | 1330 |
| 12851692 | GLCCI1 | PREDICTED: glucocorticoid induced transcript 1 (GLCCI1), mRNA. | 4.49101099 | 843 | 837 | 874 | 735 | 581 | 574 | 439 | 123 | 144 | 141 | 155 | 193 | 177 |
| 12891722 | GNG10 | guanine nucleotide binding protein (G protein), gamma 10 (GNG10), mRNA. | 4.600620093 | 3299 | 3750 | 3500 | 4570 | 2079 | 1994 | 1848 | 696 | 647 | 754 | 645 | 576 | 602 |
| 12828674 | GNPAT | glyceronephosphate O-acyltransferase (GNPAT), mRNA. | 2.050051516 | 482 | 462 | 415 | 647 | 362 | 360 | 313 | 202 | 230 | 186 | 228 | 219 | 206 |
| 12875557 | GPR125 | PREDICTED: G protein-coupled receptor 125 (GPR125), mRNA. | 4.475959222 | 1938 | 2011 | 1648 | 1993 | 1416 | 1304 | 982 | 374 | 347 | 215 | 403 | 431 | 392 |
| 12904056 | GPR173 | G protein-coupled receptor 173 (GPR173), mRNA. | 2.112684848 | 103 | 112 | 129 | 116 | 98 | 93 | 100 | 65 | 61 | 48 | 41 | 50 | 39 |
| 12849517 | GPR85 | G protein-coupled receptor 85 (GPR85), mRNA. | 14.92170589 | 197 | 227 | 102 | 482 | 38 | 22 | 75 | 12 | 10 | 8 | 10 | 15 | 10 |
| 12847775 | GPR89 | G protein-coupled receptor 89 (GPR89), mRNA. | 2.014538944 | 231 | 233 | 211 | 246 | 244 | 233 | 240 | 124 | 105 | 112 | 115 | 101 | 139 |
| 12725857 | GPT | glutamic-pyruvate transaminase (alanine aminotransferase) (GPT), mRNA. | 4.775866184 | 225 | 213 | 211 | 270 | 112 | 63 | 81 | 32 | 28 | 31 | 43 | 34 | 42 |
| 12794332 | GPX1 | glutathione peroxidase 1 (GPX1), mRNA. | 2.548325672 | 4545 | 4627 | 4811 | 3344 | 3418 | 3119 | 2811 | 1769 | 1376 | 1528 | 1865 | 1279 | 1154 |
| 12775222 | GRB14 | growth factor receptor-bound protein 14 (GRB14), mRNA. | 5.094603612 | 1581 | 1589 | 1650 | 1791 | 1211 | 1103 | 1187 | 163 | 170 | 159 | 320 | 460 | 430 |
| 12704770 | GREB1 | growth regulation by estrogen in breast cancer 1 (GREB1), mRNA. | 73.46763923 | 1813 | 1756 | 1715 | 1461 | 1316 | 1258 | 1054 | 19 | 17 | 28 | 21 | 22 | 14 |
| 12810286 | GREB1L | Growth Regulation By Estrogen In Breast Cancer-Like | 8.4750124 | 1949 | 1954 | 1747 | 1861 | 1371 | 1345 | 1226 | 130 | 191 | 97 | 237 | 256 | 246 |
| 12753198 | GRIK5 | glutamate receptor, ionotropic, kainate 5 (GRIK5), mRNA. | 2.541612815 | 117 | 94 | 117 | 84 | 77 | 69 | 81 | 34 | 25 | 45 | 42 | 38 | 31 |
| 12824957 | GSR | Glutathione Reductase | 2.006408008 | 231 | 238 | 246 | 334 | 277 | 173 | 264 | 142 | 106 | 136 | 136 | 116 | 116 |
| 12805860 | GSTA5 | Glutathione S-Transferase Alpha 5 | 48.86880515 | 1423 | 1619 | 1986 | 570 | 2590 | 498 | 708 | 38 | 22 | 23 | 37 | 14 | 30 |
| 12777908 | GTDC1 | glycosyltransferase-like domain containing 1 (GTDC1), mRNA. | 2.386343186 | 320 | 369 | 389 | 428 | 258 | 230 | 222 | 135 | 103 | 123 | 136 | 138 | 161 |
| 12858506 | GTSE1 | G-2 and S-phase expressed 1 (GTSE1), mRNA. | 3.701834377 | 121 | 140 | 115 | 81 | 59 | 46 | 48 | 29 | 17 | 22 | 29 | 21 | 23 |
| 12727451 | GYLTL1B | glycosyltransferase-like 1B (GYLTL1B), mRNA. | 5.068356163 | 171 | 215 | 107 | 191 | 157 | 72 | 46 | 32 | 29 | 27 | 24 | 30 | 20 |
| 12804010 | H2B | histone H2B (H2B), mRNA. | 3.945677331 | 310 | 319 | 386 | 268 | 368 | 239 | 249 | 60 | 91 | 90 | 81 | 78 | 65 |
| 12903920 | HDAC6 | Histone Deacetylase 6 | 2.121467072 | 265 | 294 | 316 | 281 | 364 | 308 | 261 | 125 | 126 | 118 | 154 | 158 | 163 |
| 12807695 | HDHD2 | Haloacid Dehalogenase-Like Hydrolase Domain Containing 2 | 2.500708213 | 2067 | 2008 | 1670 | 2137 | 1323 | 1067 | 1088 | 691 | 622 | 576 | 799 | 606 | 599 |
| 12897007 | HEBP2 | heme binding protein 2 (HEBP2), mRNA. | 2.615815837 | 352 | 380 | 447 | 471 | 438 | 393 | 396 | 185 | 168 | 217 | 161 | 86 | 126 |
| 12820806 | HELLS | PREDICTED: helicase, lymphoid-specific (HELLS), mRNA. | 7.877500184 | 377 | 420 | 305 | 364 | 132 | 103 | 110 | 35 | 35 | 35 | 38 | 26 | 29 |
| 12875415 | HELQ | PREDICTED: helicase, POLQ-like (HELQ), mRNA. | 2.274252893 | 160 | 181 | 184 | 210 | 108 | 110 | 144 | 62 | 61 | 56 | 71 | 84 | 80 |
| 12806854 | HIST1H1E | PREDICTED: histone cluster 1, H1e (HIST1H1E), mRNA. | 5.320123241 | 5709 | 5270 | 5820 | 4081 | 3069 | 3335 | 2899 | 712 | 747 | 623 | 843 | 1074 | 863 |
| 12800906 | HIST1H2AD | PREDICTED: histone cluster 1, H2 (HIST1H2AD), mRNA. | 53.46072279 | 2923 | 3421 | 3026 | 2601 | 2321 | 1012 | 1111 | 47 | 45 | 19 | 48 | 52 | 53 |
| 12807066 | HIST1H2AJ | PREDICTED: histone cluster 1, H2aj (HIST1H2AJ), mRNA. | 4.828999794 | 6688 | 6789 | 7068 | 6611 | 5786 | 3419 | 3825 | 1101 | 1078 | 1019 | 1322 | 1301 | 1312 |
| 12802519 | HIST1H2BB | PREDICTED: histone cluster 1, H2bb (HIST1H2BB), mRNA. | 9.957500482 | 1266 | 1226 | 971 | 823 | 494 | 216 | 283 | 69 | 58 | 72 | 83 | 89 | 83 |
| 12805809 | HIST1H4B | Histone Cluster 1, H4b | 3.258042422 | 1439 | 1316 | 1306 | 1235 | 904 | 710 | 738 | 284 | 229 | 282 | 402 | 419 | 396 |
| 12841202 | HIST2H2AB | PREDICTED: histone cluster 2, H2ab (HIST2H2AB), mRNA. | 4.785181747 | 645 | 651 | 573 | 325 | 295 | 216 | 301 | 88 | 77 | 126 | 84 | 76 | 86 |
| 12847661 | HJURP | Holliday Junction Recognition Protein | 5.901549066 | 234 | 246 | 190 | 157 | 60 | 73 | 72 | 20 | 24 | 19 | 31 | 28 | 28 |
| 12895062 | HMGB2 | high mobility group box 2 (HMGB2), mRNA. | 5.077085252 | 727 | 771 | 721 | 668 | 355 | 399 | 503 | 105 | 124 | 145 | 99 | 116 | 112 |
| 12781396 | HMGN2 | high mobility group nucleosomal binding domain 2 (HMGN2), mRNA. | 2.983044259 | 674 | 583 | 562 | 662 | 556 | 479 | 472 | 159 | 192 | 190 | 205 | 214 | 185 |
| 12876902 | HMMR | hyaluronan-mediated motility receptor (RHAMM) (HMMR), mRNA. | 6.138182347 | 337 | 379 | 336 | 290 | 92 | 69 | 92 | 38 | 40 | 38 | 34 | 31 | 42 |
| 12873186 | HPSE | Heparanase | 22.24478393 | 412 | 386 | 267 | 455 | 41 | 47 | 54 | 13 | 9 | 9 | 9 | 11 | 13 |
| 12761316 | HSD17B1 | hydroxysteroid (17-beta) dehydrogenase 1 (HSD17B1), mRNA. | 6.197762469 | 328 | 295 | 442 | 261 | 257 | 124 | 83 | 29 | 50 | 46 | 31 | 41 | 51 |
| 12803773 | HSP90AB1 | heat shock protein 90kDa alpha (cytosolic), class B member 1 (HSP90AB1), mRNA. | 2.363374929 | 331 | 347 | 232 | 455 | 520 | 429 | 303 | 162 | 160 | 153 | 154 | 185 | 135 |
| 12707900 | IAH1 | isoamyl acetate-hydrolyzing esterase 1 homolog (S. cerevisiae) (IAH1), mRNA. | 2.812786833 | 450 | 469 | 564 | 464 | 388 | 325 | 565 | 132 | 177 | 150 | 163 | 162 | 199 |
| 12789104 | IDH3A | Isocitrate Dehydrogenase 3 (NAD+) Alpha | 4.21808227 | 1338 | 1867 | 2181 | 1958 | 730 | 594 | 804 | 377 | 328 | 429 | 322 | 217 | 251 |
| 12810714 | IER3IP1 | immediate early response 3 interacting protein 1 (IER3IP1), mRNA. | 3.611514455 | 911 | 1087 | 1008 | 1140 | 556 | 450 | 487 | 211 | 185 | 219 | 269 | 199 | 255 |
| 12887803 | IFI30 | Interferon, Gamma-Inducible Protein 30 | 2.832544959 | 756 | 843 | 587 | 901 | 1058 | 705 | 745 | 286 | 221 | 217 | 299 | 303 | 366 |
| 12686432 | IGSF11 | immunoglobulin superfamily, member 11 (IGSF11), mRNA. | 3.813168669 | 2772 | 2771 | 2462 | 2890 | 1356 | 1041 | 1277 | 577 | 599 | 466 | 601 | 609 | 423 |
| 12797388 | IL17RD | interleukin 17 receptor D (IL17RD), mRNA. | 3.604523297 | 335 | 324 | 324 | 437 | 180 | 164 | 189 | 104 | 97 | 99 | 54 | 45 | 66 |
| 12843689 | IL6R | interleukin 6 receptor (IL6R), mRNA. | 7.373858848 | 681 | 813 | 734 | 1098 | 308 | 222 | 218 | 113 | 106 | 120 | 34 | 49 | 51 |
| 12795314 | IMPDH2 | IMP (inosine 5'-monophosphate) dehydrogenase 2 (IMPDH2), mRNA. | 3.015608674 | 347 | 307 | 260 | 265 | 410 | 329 | 275 | 75 | 90 | 107 | 93 | 141 | 118 |
| 12774405 | INHA | Inhibin, Alpha | 81.21359021 | 4313 | 4455 | 4291 | 3467 | 3451 | 2569 | 2596 | 50 | 26 | 46 | 35 | 59 | 49 |
| 12848966 | INHBA | inhibin, beta A (INHBA), mRNA. | 28.23868506 | 5085 | 5141 | 5052 | 5515 | 3977 | 2720 | 3009 | 175 | 145 | 197 | 100 | 177 | 133 |
| 12777446 | INHBB | inhibin, beta B (INHBB), mRNA. | 11.51125411 | 1445 | 1339 | 1084 | 1980 | 365 | 348 | 298 | 101 | 80 | 113 | 70 | 81 | 67 |
| 12860307 | IPO8 | importin 8 (IPO8), mRNA. | 2.021139958 | 591 | 634 | 552 | 643 | 366 | 383 | 426 | 237 | 281 | 232 | 234 | 275 | 266 |
| 12836813 | IQGAP3 | IQ motif containing GTPase activating protein 3 (IQGAP3), mRNA. | 3.904274589 | 163 | 146 | 141 | 99 | 69 | 49 | 79 | 36 | 31 | 22 | 25 | 21 | 28 |
| 12783512 | IRS1 | PREDICTED: insulin receptor substrate 1, transcript variant 1 (IRS1), mRNA. | 2.603598736 | 263 | 258 | 335 | 322 | 319 | 375 | 204 | 86 | 71 | 58 | 154 | 171 | 142 |
| 12887383 | ISOC1 | Isochorismatase Domain Containing 1 | 2.214409772 | 223 | 249 | 215 | 223 | 211 | 181 | 189 | 128 | 72 | 120 | 100 | 71 | 85 |
| 12880959 | JAK3 | Janus Kinase 3 | 18.89313039 | 989 | 918 | 1358 | 1012 | 1908 | 1266 | 1499 | 59 | 63 | 70 | 75 | 79 | 60 |
| 12852052 | JAZF1 | JAZF zinc finger 1 (JAZF1), mRNA. | 2.928590772 | 716 | 792 | 673 | 721 | 336 | 292 | 297 | 162 | 169 | 174 | 245 | 198 | 173 |
| 12883582 | KCNN1 | Potassium Channel, Calcium Activated Intermediate/Small Conductance Subfamily N Alpha, Member 1 | 5.143119397 | 305 | 302 | 173 | 416 | 140 | 92 | 78 | 32 | 27 | 41 | 62 | 49 | 40 |
| 12688260 | KCNN2 | Potassium Channel, Calcium Activated Intermediate/Small Conductance Subfamily N Alpha, Member 1 | 5.117442417 | 169 | 198 | 124 | 160 | 65 | 82 | 54 | 19 | 19 | 20 | 16 | 39 | 30 |
| 12875452 | KCTD8 | potassium channel tetramerisation domain containing 8 (KCTD8), mRNA. | 3.194802742 | 84 | 107 | 87 | 144 | 85 | 64 | 62 | 29 | 36 | 30 | 22 | 34 | 19 |
| 12711797 | KDELC1 | KDEL (Lys-Asp-Glu-Leu) containing 1 (KDELC1), mRNA. | 3.093627783 | 465 | 531 | 502 | 614 | 331 | 306 | 349 | 146 | 127 | 115 | 190 | 144 | 136 |
| 12691481 | KIAA0101 | KIAA0101 ortholog (KIAA0101), mRNA. | 8.392465321 | 356 | 309 | 262 | 218 | 190 | 101 | 128 | 24 | 23 | 26 | 29 | 37 | 22 |
| 12686124 | KIAA1524 | KIAA1524 ortholog (KIAA1524), mRNA. | 5.73789259 | 375 | 439 | 396 | 413 | 223 | 153 | 174 | 45 | 54 | 34 | 60 | 72 | 59 |
| 12819342 | KIF11 | kinesin family member 11 (KIF11), mRNA. | 18.00106071 | 590 | 683 | 579 | 532 | 174 | 168 | 160 | 26 | 18 | 24 | 20 | 25 | 24 |
| 12796835 | KIF15 | kinesin family member 15 (KIF15), mRNA. | 9.401071861 | 291 | 285 | 215 | 236 | 77 | 62 | 78 | 17 | 18 | 14 | 23 | 23 | 19 |
| 12732959 | KIF18A | kinesin family member 18A (KIF18A), mRNA. | 13.55623631 | 478 | 463 | 314 | 367 | 86 | 65 | 90 | 21 | 17 | 14 | 26 | 22 | 19 |
| 12880178 | KIF20A | kinesin family member 20A (KIF20A), mRNA. | 6.886727634 | 241 | 270 | 455 | 327 | 198 | 247 | 219 | 57 | 43 | 41 | 36 | 32 | 34 |
| 12815755 | KIF22 | kinesin family member 22 (KIF22), mRNA. | 4.261713207 | 151 | 161 | 153 | 117 | 66 | 55 | 59 | 26 | 24 | 25 | 32 | 17 | 28 |
| 12689529 | KIF23 | kinesin family member 23 (KIF23), mRNA. | 4.521538285 | 432 | 463 | 453 | 338 | 159 | 160 | 156 | 64 | 71 | 47 | 82 | 84 | 62 |
| 12846146 | KIF2C | kinesin family member 2C (KIF2C), mRNA. | 4.811156473 | 121 | 130 | 129 | 98 | 54 | 51 | 52 | 17 | 28 | 18 | 19 | 15 | 16 |
| 12909607 | KIF4A | kinesin family member 4A (KIF4A), mRNA. | 12.94422872 | 296 | 308 | 277 | 304 | 84 | 83 | 92 | 18 | 21 | 14 | 15 | 13 | 14 |
| 12892001 | KLHL9 | kelch-like 9 (Drosophila) (KLHL9), mRNA. | 2.054299709 | 410 | 456 | 375 | 423 | 294 | 273 | 348 | 222 | 188 | 177 | 194 | 132 | 163 |
| 12745867 | KNTC1 | kinetochore associated 1 (KNTC1), mRNA. | 13.48171792 | 387 | 400 | 292 | 261 | 99 | 75 | 93 | 18 | 19 | 14 | 19 | 16 | 16 |
| 12809293 | LAMA1 | PREDICTED: laminin, alpha 1 (LAMA1), mRNA. | 2.438679984 | 1408 | 1408 | 1711 | 1261 | 1133 | 1112 | 997 | 465 | 540 | 337 | 502 | 720 | 609 |
| 12840769 | LEPRE1 | leucine proline-enriched proteoglycan (leprecan) 1 (LEPRE1), mRNA. | 2.021589691 | 186 | 220 | 215 | 267 | 263 | 202 | 200 | 125 | 92 | 85 | 119 | 126 | 111 |
| 12773652 | LEPREL4 | PREDICTED: leprecan-like 4 (LEPREL4), mRNA. | 2.226697226 | 266 | 322 | 346 | 333 | 686 | 544 | 637 | 239 | 141 | 162 | 242 | 214 | 208 |
| 12754992 | LIG1 | ligase I, DNA, ATP-dependent (LIG1), mRNA. | 2.865848597 | 181 | 152 | 143 | 151 | 108 | 106 | 94 | 34 | 43 | 54 | 67 | 46 | 35 |
| 12866791 | LIN7A | lin-7 homolog A (C. elegans) (LIN7A), mRNA. | 3.806966872 | 166 | 172 | 189 | 167 | 55 | 49 | 56 | 25 | 35 | 25 | 38 | 39 | 30 |
| 12891544 | LINGO2 | PREDICTED: leucine rich repeat and Ig domain containing 2, transcript variant 1 (LINGO2), mRNA. | 10.50658841 | 259 | 315 | 300 | 406 | 102 | 81 | 85 | 26 | 30 | 19 | 13 | 23 | 15 |
| 12887402 | LMNB1 | lamin B1 (LMNB1), mRNA. | 5.74320981 | 164 | 174 | 126 | 146 | 73 | 75 | 72 | 17 | 21 | 20 | 21 | 24 | 21 |
| 12709937 | LNX2 | ligand of numb-protein X 2 (LNX2), mRNA. | 2.425052826 | 864 | 821 | 739 | 882 | 857 | 675 | 676 | 310 | 273 | 201 | 515 | 330 | 321 |
| 12902050 | LOC100138905 | cdna:known chromosome:UMD3.1:18:58688854:58765602:1 | 2.347607233 | 960 | 1064 | 901 | 1021 | 647 | 762 | 664 | 269 | 354 | 265 | 394 | 478 | 438 |
| 12823376 | LOC100295597 | PREDICTED: histone cluster 2, H2be-like (LOC100295597), mRNA. | 3.548563968 | 329 | 381 | 364 | 282 | 301 | 201 | 265 | 83 | 55 | 92 | 95 | 91 | 96 |
| 12906463 | LOC100296441 | PREDICTED: uncharacterized LOC100296441 (LOC100296441), miscRNA. | 8.901557626 | 316 | 269 | 182 | 283 | 173 | 80 | 202 | 32 | 29 | 28 | 15 | 23 | 18 |
| 12703589 | LOC100300483 | PREDICTED: antimicrobial peptide NK-lysin-like (LOC100300483), mRNA. // PREDICTED: antimicrobial peptide NK-lysin-like (LOC100300483), mRNA. | 2.463515802 | 185 | 162 | 118 | 153 | 157 | 157 | 185 | 40 | 56 | 58 | 77 | 89 | 68 |
| 12872964 | LOC100301175 | cdna:known chromosome:UMD3.1:6:16367635:16367865:-1 | 2.085552631 | 103 | 130 | 94 | 171 | 97 | 74 | 84 | 58 | 45 | 48 | 60 | 50 | 47 |
| 12738122 | LOC100302586 | uncharacterized LOC100302586 (LOC100302586), mRNA. | 4.084470757 | 227 | 211 | 211 | 161 | 173 | 94 | 138 | 44 | 31 | 52 | 42 | 46 | 40 |
| 12779994 | LOC100335993 | cdna:known chromosome:UMD3.1:2:115971118:116104632:-1 | 5.041334334 | 115 | 133 | 132 | 138 | 272 | 262 | 182 | 27 | 27 | 13 | 34 | 84 | 25 |
| 12717822 | LOC100336350 | PREDICTED: histone H2B type 1-like (LOC100336350), mRNA. | 3.733101171 | 384 | 390 | 327 | 288 | 211 | 160 | 220 | 75 | 74 | 79 | 67 | 68 | 91 |
| 12896448 | LOC100336854 | PREDICTED: uncharacterized LOC100336854 (LOC100336854), mRNA. | 13.02465417 | 344 | 380 | 226 | 440 | 85 | 48 | 48 | 15 | 21 | 14 | 20 | 11 | 22 |
| 12906512 | LOC100336905 | cDNA clone MGC:148868 IMAGE:8258061, complete cds. | 4.62637233 | 246 | 315 | 306 | 364 | 164 | 137 | 140 | 84 | 51 | 86 | 20 | 17 | 51 |
| 12807421 | LOC100336909 | PREDICTED: gATA binding protein 6-like (LOC100336909), mRNA. | 3.083640247 | 440 | 438 | 773 | 356 | 292 | 257 | 224 | 104 | 99 | 84 | 162 | 179 | 146 |
| 12902737 | LOC100336947 | PREDICTED: rho GTPase-activating protein 12-like (LOC100336947), miscRNA. | 5.047542965 | 96 | 129 | 54 | 130 | 35 | 35 | 30 | 14 | 13 | 13 | 16 | 18 | 12 |
| 12809083 | LOC100337099 | cdna:known chromosome:UMD3.1:24:47271783:47297664:-1 | 2.352264527 | 1038 | 977 | 903 | 921 | 605 | 449 | 471 | 334 | 302 | 314 | 378 | 300 | 326 |
| 12788927 | LOC100337259 | PREDICTED: calcium and integrin binding family member 2-like (LOC100337259), mRNA. | 2.117081951 | 272 | 270 | 304 | 261 | 198 | 181 | 181 | 136 | 122 | 108 | 116 | 113 | 80 |
| 12841924 | LOC504599 | histone H3.2 (LOC504599), mRNA. | 11.40615024 | 3007 | 2940 | 3325 | 2831 | 2828 | 1895 | 2057 | 221 | 220 | 182 | 269 | 272 | 255 |
| 12804914 | LOC505183 | PREDICTED: histone H2B type 1-like (LOC505183), mRNA. | 6.588068964 | 2044 | 2066 | 1512 | 1183 | 874 | 578 | 632 | 223 | 164 | 159 | 212 | 244 | 155 |
| 12683974 | LOC507471 | PREDICTED: mpv17-like protein 2-like (LOC507471), miscRNA. | 2.919641628 | 589 | 631 | 632 | 436 | 533 | 338 | 286 | 122 | 156 | 109 | 132 | 240 | 253 |
| 12830486 | LOC512435 | cdna:known chromosome:UMD3.1:29:272264:273010:1 | 2.884186408 | 1971 | 2028 | 1770 | 2236 | 1148 | 1250 | 1151 | 618 | 678 | 581 | 568 | 505 | 483 |
| 12830490 | LOC512612 | PREDICTED: histone H2B type 1-like (LOC512612), mRNA. | 2.03558339 | 408 | 498 | 427 | 371 | 360 | 308 | 353 | 183 | 201 | 177 | 191 | 211 | 184 |
| 12805811 | LOC517139 | PREDICTED: histone cluster 1, H3a-like (LOC517139), mRNA. | 6.705200488 | 487 | 487 | 408 | 691 | 357 | 174 | 253 | 68 | 59 | 46 | 65 | 67 | 61 |
| 12719715 | LOC519069 | PREDICTED: histone H2B type 1-like (LOC519069), mRNA. | 4.670203328 | 668 | 767 | 607 | 419 | 441 | 323 | 413 | 112 | 90 | 110 | 117 | 144 | 95 |
| 12860174 | LOC521525 | PREDICTED: histone cluster 2, H2be-like (LOC521525), mRNA. | 2.495663041 | 186 | 174 | 156 | 168 | 115 | 120 | 136 | 60 | 81 | 52 | 66 | 67 | 36 |
| 12805856 | LOC521580 | PREDICTED: histone H2B type 1-like (LOC521580), mRNA. | 7.981203572 | 202 | 136 | 174 | 176 | 78 | 43 | 45 | 16 | 20 | 16 | 15 | 15 | 9 |
| 12802386 | LOC521581 | PREDICTED: histone cluster 1, H3a-like (LOC521581), mRNA. | 6.580703466 | 5662 | 4595 | 5445 | 4463 | 3948 | 3027 | 2889 | 753 | 498 | 520 | 757 | 799 | 584 |
| 12805950 | LOC525511 | PREDICTED: histone cluster 1, H3a-like (LOC525511), mRNA. | 13.5152739 | 1168 | 1098 | 1059 | 868 | 690 | 457 | 469 | 42 | 49 | 46 | 94 | 68 | 70 |
| 12728515 | LOC525863 | PREDICTED: histone cluster 1, H4i-like (LOC525863), mRNA. | 3.390363801 | 1808 | 2108 | 2088 | 1833 | 1415 | 1060 | 1074 | 423 | 393 | 421 | 524 | 618 | 499 |
| 12802575 | LOC527388 | PREDICTED: histone cluster 1, H4i-like (LOC527388), mRNA. | 6.363386971 | 455 | 366 | 324 | 350 | 229 | 98 | 136 | 29 | 55 | 39 | 45 | 39 | 56 |
| 12777625 | LOC531990 | cdna:known chromosome:UMD3.1:2:89166306:89166719:1 | 4.154850957 | 169 | 136 | 147 | 146 | 145 | 71 | 67 | 33 | 34 | 30 | 38 | 17 | 30 |
| 12764263 | LOC532189 | cdna:known chromosome:UMD3.1:19:22025796:22086708:1 | 3.290021309 | 4663 | 5124 | 4738 | 5005 | 3063 | 2975 | 3088 | 1370 | 1233 | 1481 | 1098 | 1147 | 1137 |
| 12834394 | LOC532848 | cdna:known chromosome:UMD3.1:29:1067600:1115956:-1 | 2.324293398 | 274 | 286 | 281 | 293 | 183 | 222 | 228 | 97 | 100 | 90 | 115 | 136 | 114 |
| 12740359 | LOC534587 | cdna:known chromosome:UMD3.1:16:29993988:30067842:-1 | 3.023865353 | 145 | 154 | 110 | 116 | 66 | 66 | 69 | 36 | 34 | 32 | 30 | 46 | 28 |
| 12803265 | LOC539875 | PREDICTED: histone cluster 1, H3a-like (LOC539875), mRNA. | 15.88800535 | 1268 | 1074 | 1565 | 874 | 864 | 714 | 608 | 62 | 72 | 34 | 86 | 62 | 59 |
| 12740723 | LOC539953 | cdna:known chromosome:UMD3.1:16:73315563:73364134:-1 | 8.057704015 | 685 | 650 | 563 | 463 | 235 | 179 | 250 | 59 | 62 | 47 | 63 | 45 | 46 |
| 12691776 | LOC613752 | cdna:known chromosome:UMD3.1:10:65747190:65747567:1 | 2.056209848 | 117 | 114 | 126 | 132 | 118 | 75 | 87 | 43 | 63 | 74 | 46 | 48 | 47 |
| 12786562 | LOC614206 | PREDICTED: histone H2B type 1-like (LOC614206), mRNA. | 3.192498444 | 184 | 220 | 207 | 316 | 197 | 130 | 143 | 46 | 79 | 63 | 60 | 60 | 67 |
| 12720844 | LOC614378 | PREDICTED: histone H2B type 1-like (LOC614378), mRNA. | 2.097372129 | 148 | 133 | 145 | 145 | 129 | 109 | 147 | 64 | 79 | 63 | 68 | 56 | 61 |
| 12806766 | LOC616611 | PREDICTED: histone cluster 1, H2ai-like (LOC616611), mRNA. | 3.433059297 | 338 | 348 | 341 | 207 | 251 | 126 | 155 | 77 | 65 | 108 | 67 | 63 | 61 |
| 12806778 | LOC616776 | PREDICTED: histone H2B type 1-like (LOC616776), mRNA. | 4.282334864 | 4417 | 4205 | 4441 | 3398 | 2770 | 2108 | 2438 | 782 | 699 | 786 | 750 | 1031 | 711 |
| 12803582 | LOC616800 | PREDICTED: histone cluster 1, H3a-like (LOC616800), mRNA. | 7.81829214 | 3160 | 3128 | 3064 | 2459 | 2632 | 1831 | 1820 | 357 | 244 | 278 | 365 | 462 | 277 |
| 12803584 | LOC616819 | PREDICTED: histone cluster 1, H3a-like (LOC616819), mRNA. | 12.58005134 | 5866 | 5190 | 5811 | 2578 | 4488 | 3291 | 2545 | 321 | 290 | 247 | 424 | 329 | 418 |
| 12682510 | LOC616840 | PREDICTED: 60S ribosomal protein L3-like (LOC616840), miscRNA. | 3.234427466 | 190 | 225 | 199 | 179 | 116 | 148 | 107 | 37 | 49 | 59 | 58 | 50 | 56 |
| 12710644 | LOC617043 | PREDICTED: histone H2B type 1-like (LOC617043), mRNA. | 2.470334758 | 116 | 97 | 107 | 96 | 75 | 84 | 79 | 37 | 32 | 42 | 33 | 39 | 44 |
| 12765661 | LOC618012 | PREDICTED: histone H2B type 1-like (LOC618012), mRNA. | 4.481512462 | 920 | 1038 | 1007 | 711 | 664 | 479 | 503 | 170 | 157 | 182 | 162 | 202 | 145 |
| 12803716 | LOC618824 | PREDICTED: histone cluster 1, H2ai-like (LOC618824), mRNA. | 3.531095822 | 6621 | 6123 | 6294 | 6967 | 6305 | 3583 | 4155 | 1671 | 1355 | 1273 | 2008 | 1768 | 1647 |
| 12687118 | LOC781410 | PREDICTED: histone cluster 1, H2bm-like (LOC781410), mRNA. | 3.019209507 | 111 | 143 | 109 | 99 | 81 | 58 | 59 | 26 | 25 | 25 | 30 | 46 | 35 |
| 12862888 | LOC781777 | cdna:known chromosome:UMD3.1:5:107749635:107750145:1 | 2.182870664 | 223 | 287 | 162 | 291 | 437 | 391 | 463 | 166 | 134 | 121 | 155 | 164 | 146 |
| 12872704 | LOC782350 | PREDICTED: histone cluster 2, H2be-like (LOC782350), mRNA. | 4.751455517 | 164 | 252 | 167 | 152 | 118 | 65 | 80 | 38 | 22 | 31 | 38 | 25 | 26 |
| 12803875 | LOC782971 | PREDICTED: histone H2B type 1-like (LOC782971), mRNA. | 4.170422459 | 3582 | 4190 | 4101 | 3118 | 2878 | 2596 | 2894 | 839 | 684 | 826 | 825 | 939 | 688 |
| 12821102 | LOC783804 | cdna:known chromosome:UMD3.1:26:21057820:21058839:1 | 4.278855469 | 387 | 388 | 348 | 321 | 139 | 104 | 111 | 79 | 67 | 72 | 55 | 37 | 50 |
| 12905452 | LOC784476 | cdna:known chromosome:UMD3.1:X:19420491:19476518:1 | 6.00281425 | 129 | 165 | 250 | 398 | 88 | 115 | 112 | 36 | 31 | 57 | 16 | 20 | 19 |
| 12726253 | LOC784747 | cdna:known chromosome:UMD3.1:14:60994139:61520208:-1 | 6.42426065 | 1066 | 1046 | 1196 | 919 | 743 | 687 | 732 | 167 | 175 | 138 | 89 | 175 | 108 |
| 12852506 | LOC786657 | PREDICTED: uncharacterized LOC786657 (LOC786657), mRNA. | 3.352542412 | 177 | 217 | 216 | 183 | 87 | 87 | 87 | 52 | 41 | 40 | 35 | 52 | 50 |
| 12871367 | LOC786787 | PREDICTED: histone H2B type 1-like, transcript variant 1 (LOC786787), mRNA. | 2.921234781 | 462 | 512 | 489 | 373 | 387 | 252 | 363 | 106 | 143 | 119 | 115 | 174 | 176 |
| 12807147 | LOC787465 | PREDICTED: histone H2B type 1-like (LOC787465), mRNA. | 7.296026129 | 2977 | 2611 | 2912 | 2325 | 1771 | 1100 | 1167 | 288 | 281 | 236 | 367 | 286 | 287 |
| 12804021 | LOC788250 | PREDICTED: histone cluster 1, H3a-like (LOC788250), mRNA. | 10.63148937 | 1477 | 1313 | 1445 | 1207 | 662 | 497 | 453 | 124 | 74 | 154 | 79 | 58 | 80 |
| 12821137 | LOC788635 | PREDICTED: uncharacterized LOC788635 (LOC788635), mRNA. | 2.979197693 | 127 | 125 | 134 | 165 | 83 | 88 | 79 | 37 | 41 | 31 | 42 | 37 | 42 |
| 12890327 | LPPR1 | lipid phosphate phosphatase-related protein type 1 (LPPR1), mRNA. | 26.62023604 | 844 | 1108 | 798 | 978 | 173 | 342 | 425 | 23 | 27 | 26 | 29 | 26 | 20 |
| 12836232 | LRP8 | low density lipoprotein receptor-related protein 8, apolipoprotein e receptor (LRP8), mRNA. | 35.57825624 | 5761 | 5672 | 6791 | 5010 | 3775 | 3711 | 3998 | 201 | 125 | 145 | 145 | 88 | 132 |
| 12806287 | LRRC16A | leucine rich repeat containing 16A (LRRC16A), mRNA. | 4.52616004 | 260 | 300 | 281 | 332 | 299 | 245 | 299 | 73 | 50 | 78 | 65 | 52 | 64 |
| 12881518 | LSM4 | LSM4 homolog, U6 small nuclear RNA associated (S. cerevisiae) (LSM4), mRNA. | 2.335124673 | 854 | 844 | 750 | 541 | 815 | 647 | 917 | 329 | 341 | 326 | 346 | 248 | 380 |
| 12750495 | LSR | Lipolysis Stimulated Lipoprotein Receptor | 2.875943188 | 303 | 272 | 284 | 253 | 346 | 191 | 224 | 89 | 90 | 110 | 91 | 84 | 94 |
| 12784665 | LYPLA2 | Lysophospholipase II | 2.286838365 | 521 | 439 | 417 | 407 | 358 | 343 | 362 | 169 | 168 | 188 | 183 | 167 | 191 |
| 12870366 | MAD2L1 | MAD2 mitotic arrest deficient-like 1 (yeast) (MAD2L1), mRNA. | 3.38390859 | 118 | 133 | 171 | 176 | 82 | 63 | 67 | 19 | 50 | 46 | 23 | 38 | 29 |
| 12902720 | MAGED4B | melanoma antigen family D, 4B (MAGED4B), mRNA. | 2.666726711 | 88 | 93 | 109 | 148 | 137 | 193 | 139 | 55 | 40 | 35 | 50 | 54 | 59 |
| 12724911 | MAL2 | mal, T-cell differentiation protein 2 (gene/pseudogene) (MAL2), mRNA. | 3.560472418 | 1398 | 1422 | 1697 | 1478 | 1340 | 864 | 1080 | 386 | 300 | 316 | 345 | 401 | 486 |
| 12901045 | MAP3K5 | mitogen-activated protein kinase kinase kinase 5 (MAP3K5), mRNA. | 4.298644899 | 788 | 806 | 603 | 747 | 261 | 199 | 236 | 95 | 120 | 98 | 82 | 164 | 166 |
| 12737033 | MARK1 | MAP/microtubule affinity-regulating kinase 1 (MARK1), mRNA. | 2.804928255 | 624 | 780 | 540 | 1261 | 616 | 551 | 467 | 191 | 135 | 173 | 306 | 340 | 334 |
| 12716738 | MASTL | microtubule associated serine/threonine kinase-like (MASTL), mRNA. | 7.139442916 | 195 | 215 | 160 | 177 | 70 | 63 | 82 | 12 | 10 | 18 | 21 | 24 | 30 |
| 12726215 | MATN2 | matrilin 2 (MATN2), mRNA. | 6.106348136 | 394 | 367 | 290 | 375 | 471 | 809 | 727 | 84 | 75 | 73 | 108 | 70 | 72 |
| 12798786 | MCM2 | Minichromosome Maintenance Complex Component 2 | 2.735142852 | 250 | 257 | 204 | 157 | 165 | 135 | 144 | 60 | 77 | 54 | 73 | 69 | 78 |
| 12804452 | MCM3 | minichromosome maintenance complex component 3 (MCM3), mRNA. | 2.785693451 | 225 | 237 | 201 | 162 | 120 | 85 | 108 | 59 | 68 | 42 | 64 | 68 | 49 |
| 12823429 | MCM4 | Minichromosome Maintenance Complex Component 4 | 2.407501554 | 436 | 380 | 347 | 297 | 190 | 223 | 220 | 137 | 122 | 140 | 107 | 124 | 115 |
| 12858387 | MCM5 | minichromosome maintenance complex component 5 (MCM5), mRNA. | 2.573695381 | 329 | 281 | 227 | 159 | 187 | 114 | 124 | 65 | 71 | 78 | 95 | 83 | 81 |
| 12776770 | MCM6 | Minichromosome Maintenance Complex Component 6 | 2.625297932 | 485 | 451 | 417 | 363 | 260 | 267 | 329 | 143 | 147 | 131 | 139 | 140 | 139 |
| 12813792 | MCM7 | Minichromosome Maintenance Complex Component 7 | 2.258402919 | 520 | 443 | 424 | 348 | 283 | 281 | 275 | 156 | 170 | 133 | 175 | 194 | 148 |
| 12890559 | MELK | maternal embryonic leucine zipper kinase (MELK), mRNA. | 7.382786824 | 347 | 383 | 331 | 289 | 99 | 67 | 104 | 35 | 35 | 27 | 31 | 29 | 31 |
| 12849407 | MEST | mesoderm specific transcript homolog (mouse) (MEST), mRNA. | 2.334363734 | 135 | 149 | 96 | 112 | 156 | 196 | 178 | 116 | 64 | 66 | 46 | 43 | 40 |
| 12810060 | MEX3C | Mex-3 RNA Binding Family Member C | 3.267996328 | 1330 | 1428 | 1279 | 1504 | 471 | 420 | 613 | 336 | 301 | 237 | 330 | 343 | 301 |
| 12774541 | MFAP2 | microfibrillar-associated protein 2 (MFAP2), mRNA. | 5.824798251 | 112 | 92 | 86 | 93 | 403 | 402 | 277 | 29 | 42 | 41 | 36 | 44 | 24 |
| 12791683 | MFGE8 | milk fat globule-EGF factor 8 protein (MFGE8), mRNA. | 4.553951011 | 5551 | 5524 | 5859 | 5086 | 2964 | 2941 | 2901 | 1186 | 862 | 891 | 1276 | 747 | 841 |
| 12850071 | MIOS | missing oocyte, meiosis regulator, homolog (Drosophila) (MIOS), mRNA. | 2.330566234 | 239 | 249 | 254 | 313 | 136 | 170 | 156 | 99 | 69 | 85 | 95 | 96 | 115 |
| 12794225 | MIR1284 | microRNA mir-1284 (MIR1284), microRNA. | 6.835906968 | 102 | 115 | 115 | 178 | 44 | 44 | 38 | 9 | 13 | 8 | 13 | 17 | 19 |
| 12708822 | MIR2303 | microRNA mir-2303 (MIR2303), microRNA. | 2.837894731 | 110 | 116 | 116 | 134 | 108 | 104 | 97 | 26 | 42 | 28 | 27 | 63 | 51 |
| 12872980 | MIR2450A | microRNA mir-2450a (MIR2450A), microRNA. | 2.341002924 | 187 | 228 | 186 | 189 | 237 | 214 | 248 | 54 | 90 | 79 | 46 | 131 | 145 |
| 12872978 | MIR2450B | microRNA mir-2450b (MIR2450B), microRNA. | 2.244147772 | 216 | 199 | 218 | 235 | 270 | 257 | 335 | 60 | 92 | 102 | 95 | 164 | 148 |
| 12882846 | MIR2463 | microRNA mir-2463 (MIR2463), microRNA. | 2.528816752 | 115 | 109 | 98 | 122 | 73 | 66 | 98 | 30 | 42 | 24 | 44 | 42 | 49 |
| 12902558 | MIR421 | microRNA mir-421 (MIR421), microRNA. | 3.727031108 | 211 | 200 | 192 | 198 | 275 | 368 | 403 | 46 | 89 | 32 | 45 | 136 | 77 |
| 12792052 | MIS18BP1 | MIS18 binding protein 1 (MIS18BP1), mRNA. | 7.181301801 | 441 | 470 | 395 | 467 | 128 | 103 | 102 | 36 | 37 | 44 | 54 | 32 | 48 |
| 12822057 | MKI67 | Marker Of Proliferation Ki-67 | 14.97292677 | 613 | 595 | 605 | 452 | 274 | 209 | 218 | 26 | 29 | 23 | 28 | 35 | 28 |
| 12715490 | MKX | mohawk homeobox (MKX), mRNA. | 7.843886832 | 521 | 533 | 532 | 659 | 227 | 192 | 204 | 88 | 55 | 86 | 26 | 25 | 34 |
| 12770033 | MMD | monocyte to macrophage differentiation-associated (MMD), mRNA. | 2.179599059 | 1208 | 1051 | 747 | 933 | 641 | 656 | 547 | 482 | 314 | 333 | 453 | 344 | 348 |
| 12897842 | MMS22L | MMS22-like, DNA repair protein (MMS22L), mRNA. | 3.06523895 | 209 | 185 | 165 | 159 | 64 | 75 | 97 | 55 | 42 | 30 | 48 | 34 | 58 |
| 12891600 | MOB3B | MOB kinase activator 3B (MOB3B), mRNA. | 3.307505161 | 390 | 478 | 329 | 345 | 269 | 222 | 193 | 144 | 127 | 145 | 60 | 53 | 48 |
| 12764060 | MRC2 | mannose receptor, C type 2 (MRC2), mRNA. | 3.476085288 | 262 | 274 | 215 | 281 | 304 | 321 | 261 | 83 | 81 | 84 | 84 | 73 | 68 |
| 12809952 | MRO | maestro (MRO), mRNA. | 6.688016514 | 2699 | 2753 | 3221 | 2088 | 1763 | 1817 | 1347 | 176 | 227 | 152 | 382 | 650 | 424 |
| 12704484 | MRPL41 | Mitochondrial Ribosomal Protein L41 | 2.345167891 | 1938 | 1751 | 1616 | 1406 | 2439 | 1824 | 1533 | 662 | 718 | 631 | 969 | 773 | 819 |
| 12686832 | MRPS6 | mitochondrial ribosomal protein S6 (MRPS6), nuclear gene encoding mitochondrial protein, mRNA. | 4.401908877 | 1602 | 1729 | 1724 | 1856 | 854 | 719 | 721 | 316 | 309 | 351 | 280 | 254 | 283 |
| 12829664 | MS4A8B | membrane-spanning 4-domains, subfamily A, member 8B (MS4A8B), mRNA. | 3.046423004 | 1649 | 1514 | 2293 | 729 | 2124 | 2361 | 3095 | 592 | 619 | 799 | 631 | 445 | 786 |
| 12900935 | MTHFD1L | methylenetetrahydrofolate dehydrogenase (NADP+ dependent) 1-like (MTHFD1L), nuclear gene encoding mitochondrial protein, mRNA. | 6.843463624 | 678 | 702 | 467 | 887 | 266 | 217 | 259 | 57 | 54 | 50 | 78 | 124 | 72 |
| 12905341 | MUM1L1 | PREDICTED: melanoma associated antigen (mutated) 1-like 1 (MUM1L1), mRNA. | 3.058319129 | 237 | 256 | 313 | 293 | 283 | 274 | 247 | 107 | 77 | 91 | 86 | 102 | 69 |
| 12886543 | MXD3 | MAX dimerization protein 3 (MXD3), mRNA. | 2.151511671 | 154 | 141 | 134 | 107 | 84 | 94 | 108 | 34 | 63 | 56 | 56 | 48 | 69 |
| 12724318 | MYBL1 | v-myb myeloblastosis viral oncogene homolog (avian)-like 1 (MYBL1), mRNA. | 4.935734323 | 342 | 340 | 336 | 250 | 77 | 62 | 81 | 44 | 36 | 37 | 49 | 41 | 54 |
| 12714668 | MYBL2 | v-myb myeloblastosis viral oncogene homolog (avian)-like 2 (MYBL2), mRNA. | 3.169131299 | 280 | 223 | 184 | 148 | 144 | 112 | 104 | 58 | 57 | 72 | 43 | 44 | 49 |
| 12689969 | MYEF2 | myelin expression factor 2 (MYEF2), mRNA. | 5.78271043 | 1110 | 1281 | 1139 | 1506 | 528 | 489 | 489 | 144 | 135 | 141 | 162 | 172 | 216 |
| 12778245 | MYO1B | myosin IB (MYO1B), mRNA. | 3.354894233 | 1069 | 1125 | 1008 | 1189 | 489 | 466 | 482 | 313 | 270 | 294 | 171 | 192 | 248 |
| 12844388 | NASP | nuclear autoantigenic sperm protein (histone-binding) (NASP), mRNA. | 2.470881001 | 182 | 188 | 165 | 227 | 109 | 130 | 137 | 56 | 65 | 60 | 78 | 73 | 64 |
| 12869291 | NCAPD2 | non-SMC condensin I complex, subunit D2 (NCAPD2), mRNA. | 2.907880188 | 273 | 264 | 271 | 225 | 153 | 161 | 126 | 72 | 74 | 53 | 77 | 90 | 67 |
| 12871525 | NCAPG | Non-SMC Condensin I Complex, Subunit G | 13.86448539 | 647 | 656 | 441 | 599 | 173 | 153 | 177 | 39 | 28 | 27 | 31 | 24 | 26 |
| 12854230 | NCAPG2 | non-SMC condensin II complex, subunit G2 (NCAPG2), mRNA. | 3.708404353 | 267 | 271 | 270 | 193 | 129 | 98 | 112 | 54 | 61 | 44 | 52 | 48 | 50 |
| 12698951 | NCAPH | non-SMC condensin I complex, subunit H (NCAPH), mRNA. | 8.660109184 | 512 | 513 | 494 | 307 | 191 | 143 | 159 | 43 | 34 | 42 | 36 | 36 | 38 |
| 12808472 | NDC80 | NDC80 homolog, kinetochore complex component (S. cerevisiae) (NDC80), mRNA. | 13.24770456 | 354 | 373 | 354 | 357 | 84 | 77 | 81 | 15 | 21 | 25 | 18 | 15 | 14 |
| 12719311 | NDRG3 | NDRG family member 3 (NDRG3), mRNA. | 2.644587089 | 1119 | 1309 | 1568 | 1321 | 804 | 852 | 833 | 520 | 369 | 549 | 432 | 309 | 350 |
| 12787041 | NDUFS4 | NADH dehydrogenase (ubiquinone) Fe-S protein 4, 18kDa (NADH-coenzyme Q reductase) (NDUFS4), nuclear gene encoding mitochondrial protein, mRNA. | 2.631558388 | 2369 | 2570 | 2267 | 3223 | 1513 | 1019 | 1198 | 864 | 764 | 938 | 784 | 632 | 632 |
| 12736304 | NEK2 | NIMA (never in mitosis gene a)-related kinase 2 (NEK2), mRNA. | 3.670404734 | 101 | 109 | 106 | 96 | 43 | 29 | 40 | 26 | 14 | 19 | 21 | 16 | 26 |
| 12902214 | NGFRAP1 | nerve growth factor receptor (TNFRSF16) associated protein 1 (NGFRAP1), mRNA. | 2.403725274 | 212 | 202 | 245 | 284 | 247 | 192 | 203 | 69 | 95 | 59 | 102 | 120 | 120 |
| 12775945 | NINJ1 | Ninjurin 1 | 2.959908189 | 736 | 748 | 999 | 632 | 424 | 445 | 530 | 338 | 207 | 327 | 149 | 124 | 162 |
| 12762062 | NLK | nemo-like kinase (NLK), mRNA. | 2.419722329 | 493 | 487 | 406 | 612 | 217 | 259 | 250 | 151 | 186 | 158 | 161 | 148 | 161 |
| 12791964 | NMB | Neuromedin B | 4.194549434 | 515 | 494 | 473 | 568 | 422 | 528 | 417 | 70 | 79 | 90 | 113 | 180 | 166 |
| 12851927 | NME2 | non-metastatic cells 2, protein (NM23B) expressed in (NME2), mRNA. | 2.049280774 | 770 | 752 | 788 | 1007 | 1738 | 1231 | 1412 | 482 | 438 | 700 | 601 | 389 | 611 |
| 12812658 | NOMO2 | PREDICTED: NODAL modulator 2 (NOMO2), mRNA. | 3.202652074 | 2956 | 2721 | 3293 | 2284 | 1673 | 1199 | 1353 | 957 | 692 | 935 | 567 | 503 | 488 |
| 12767532 | NOS2 | nitric oxide synthase 2, inducible (NOS2), mRNA. | 10.61497233 | 755 | 764 | 580 | 583 | 377 | 147 | 124 | 54 | 59 | 49 | 32 | 41 | 34 |
| 12702509 | NPAS2 | neuronal PAS domain protein 2 (NPAS2), mRNA. | 3.611184663 | 184 | 192 | 172 | 154 | 163 | 124 | 111 | 48 | 55 | 42 | 40 | 34 | 43 |
| 12732503 | NPAT | PREDICTED: nuclear protein, ataxia-telangiectasia locus (NPAT), mRNA. | 2.305369689 | 374 | 408 | 347 | 439 | 198 | 187 | 207 | 129 | 136 | 118 | 139 | 133 | 149 |
| 12888975 | NPR2 | mRNA for retina guanylate cyclase. | 2.624847244 | 399 | 436 | 272 | 436 | 235 | 237 | 286 | 133 | 106 | 109 | 118 | 141 | 144 |
| 12805296 | NRM | nurim (nuclear envelope membrane protein) (NRM), mRNA. | 2.388209576 | 367 | 299 | 338 | 201 | 219 | 164 | 158 | 103 | 114 | 101 | 94 | 120 | 94 |
| 12772938 | NT5M | 5',3'-nucleotidase, mitochondrial (NT5M), nuclear gene encoding mitochondrial protein, mRNA. | 2.44987274 | 161 | 137 | 184 | 170 | 148 | 126 | 126 | 69 | 56 | 63 | 57 | 65 | 58 |
| 12763683 | NTN1 | netrin 1 (NTN1), mRNA. | 3.490141192 | 381 | 360 | 250 | 354 | 162 | 158 | 161 | 93 | 89 | 84 | 57 | 75 | 50 |
| 12842942 | NTRK1 | type II neuronal form TrkA mRNA, partial cds. | 3.936117028 | 604 | 520 | 256 | 249 | 797 | 380 | 541 | 102 | 237 | 154 | 51 | 52 | 133 |
| 12845106 | NUF2 | NUF2, NDC80 kinetochore complex component, homolog (S. cerevisiae) (NUF2), mRNA. | 11.11111921 | 346 | 342 | 327 | 346 | 110 | 72 | 82 | 21 | 19 | 16 | 24 | 23 | 23 |
| 12785270 | NUP155 | nucleoporin 155kDa (NUP155), mRNA. | 2.277612411 | 371 | 364 | 339 | 347 | 210 | 284 | 268 | 133 | 144 | 139 | 136 | 119 | 150 |
| 12733180 | NUP160 | nucleoporin 160kDa (NUP160), mRNA. | 2.059445926 | 175 | 170 | 171 | 154 | 121 | 105 | 116 | 77 | 74 | 64 | 73 | 64 | 70 |
| 12698906 | NUP188 | nucleoporin 188kDa (NUP188), mRNA. | 2.874925201 | 489 | 504 | 508 | 490 | 299 | 257 | 318 | 153 | 134 | 137 | 139 | 125 | 167 |
| 12850474 | NUP205 | nucleoporin 205kDa (NUP205), mRNA. | 2.573094877 | 614 | 629 | 648 | 596 | 369 | 397 | 372 | 201 | 208 | 185 | 243 | 153 | 217 |
| 12900514 | NUP43 | nucleoporin 43kDa (NUP43), mRNA. | 2.108209671 | 197 | 195 | 181 | 214 | 111 | 95 | 171 | 69 | 72 | 85 | 84 | 85 | 79 |
| 12894401 | NXNL2 | nucleoredoxin-like 2 (NXNL2), mRNA. | 4.542812556 | 341 | 346 | 228 | 383 | 128 | 135 | 123 | 38 | 54 | 65 | 46 | 58 | 56 |
| 12715468 | NXT1 | Nuclear Transport Factor 2-Like Export Factor 1 | 2.233325122 | 143 | 145 | 146 | 127 | 125 | 95 | 93 | 61 | 60 | 64 | 70 | 46 | 36 |
| 12695057 | NYNRIN | NYN domain and retroviral integrase containing (NYNRIN), mRNA. | 3.35681924 | 268 | 334 | 342 | 283 | 237 | 257 | 267 | 136 | 103 | 104 | 61 | 57 | 46 |
| 12781258 | OBSL1 | obscurin-like 1 (OBSL1), mRNA. | 7.334646906 | 447 | 399 | 488 | 347 | 251 | 170 | 175 | 39 | 50 | 38 | 48 | 52 | 40 |
| 12839892 | ODF2L | outer dense fiber of sperm tails 2-like (ODF2L), mRNA. | 3.288120381 | 513 | 603 | 707 | 960 | 390 | 358 | 306 | 103 | 161 | 139 | 187 | 237 | 174 |
| 12906742 | ODZ1 | odz, odd Oz/ten-m homolog 1 (Drosophila) (ODZ1), mRNA. | 6.121232786 | 179 | 195 | 258 | 266 | 86 | 86 | 69 | 24 | 19 | 32 | 27 | 26 | 30 |
| 12838179 | ORC1 | origin recognition complex, subunit 1 (ORC1), mRNA. | 3.343747891 | 118 | 125 | 81 | 92 | 61 | 50 | 46 | 23 | 28 | 23 | 26 | 24 | 23 |
| 12900595 | ORC3 | origin recognition complex, subunit 3 (ORC3), mRNA. | 3.192984645 | 290 | 321 | 262 | 341 | 186 | 179 | 176 | 64 | 86 | 78 | 81 | 70 | 92 |
| 12867044 | OSBPL8 | Oxysterol Binding Protein-Like 8 | 2.39322097 | 1254 | 1312 | 759 | 1051 | 495 | 515 | 542 | 340 | 352 | 348 | 377 | 367 | 340 |
| 12776601 | OSGEPL1 | O-sialoglycoprotein endopeptidase-like 1 (OSGEPL1), mRNA. | 2.073827567 | 138 | 142 | 98 | 117 | 84 | 83 | 82 | 54 | 49 | 45 | 52 | 50 | 57 |
| 12839991 | PABPC4 | poly(A) binding protein, cytoplasmic 4 (inducible form) (PABPC4), mRNA. | 2.034852369 | 824 | 842 | 984 | 929 | 1206 | 1172 | 896 | 458 | 442 | 468 | 545 | 490 | 483 |
| 12891796 | PALM2 | paralemmin 2 (PALM2), mRNA. | 3.66826988 | 1005 | 1135 | 624 | 1138 | 843 | 871 | 704 | 280 | 228 | 260 | 216 | 268 | 225 |
| 12862370 | PARPBP | PREDICTED: chromosome 5 open reading frame, human C12orf48 (C5H12orf48), mRNA. | 6.129188471 | 157 | 160 | 142 | 150 | 44 | 33 | 42 | 15 | 20 | 16 | 18 | 20 | 12 |
| 12891134 | PBK | PDZ binding kinase (PBK), mRNA. | 13.64599483 | 413 | 380 | 387 | 381 | 149 | 124 | 130 | 17 | 21 | 17 | 24 | 20 | 23 |
| 12815388 | PCOLCE | procollagen C-endopeptidase enhancer (PCOLCE), mRNA. | 4.758732789 | 599 | 552 | 753 | 597 | 516 | 684 | 610 | 174 | 120 | 141 | 92 | 148 | 100 |
| 12879073 | PCYOX1L | prenylcysteine oxidase 1 like (PCYOX1L), mRNA. | 2.125756676 | 290 | 251 | 239 | 215 | 206 | 146 | 169 | 110 | 126 | 95 | 96 | 102 | 83 |
| 12781114 | PDIK1L | PDLIM1 interacting kinase 1 like (PDIK1L), mRNA. | 2.448164069 | 209 | 247 | 221 | 292 | 112 | 134 | 160 | 94 | 74 | 74 | 89 | 68 | 83 |
| 12718907 | PDSS1 | prenyl (decaprenyl) diphosphate synthase, subunit 1 (PDSS1), mRNA. | 13.22534811 | 778 | 890 | 1120 | 1197 | 309 | 237 | 275 | 58 | 57 | 42 | 47 | 52 | 56 |
| 12761334 | PEMT | phosphatidylethanolamine N-methyltransferase (PEMT), nuclear gene encoding mitochondrial protein, mRNA. | 2.281013697 | 544 | 445 | 462 | 361 | 586 | 352 | 284 | 195 | 160 | 177 | 248 | 191 | 170 |
| 12780621 | PGAP1 | post-GPI attachment to proteins 1 (PGAP1), mRNA. | 2.409792128 | 271 | 259 | 185 | 295 | 160 | 141 | 133 | 80 | 67 | 90 | 104 | 87 | 86 |
| 12887821 | PGLS | 6-Phosphogluconolactonase | 2.223608981 | 214 | 231 | 213 | 238 | 305 | 188 | 216 | 126 | 82 | 86 | 108 | 109 | 108 |
| 12726622 | PGR | Progesterone Receptor | 3.221238279 | 135 | 147 | 146 | 171 | 139 | 127 | 130 | 51 | 38 | 38 | 49 | 44 | 45 |
| 12903405 | PHF16 | PREDICTED: PHD finger protein 16 (PHF16), mRNA. | 3.076761225 | 352 | 361 | 401 | 425 | 119 | 138 | 142 | 97 | 77 | 88 | 88 | 68 | 123 |
| 12731671 | PHF21A | PHD finger protein 21A (PHF21A), mRNA. | 2.88724785 | 782 | 881 | 820 | 761 | 468 | 459 | 395 | 205 | 195 | 195 | 231 | 258 | 270 |
| 12900556 | PHIP | PREDICTED: pleckstrin homology domain interacting protein (PHIP), mRNA. | 2.189678466 | 642 | 695 | 699 | 817 | 441 | 528 | 517 | 240 | 337 | 242 | 270 | 300 | 310 |
| 12904175 | PHKA2 | phosphorylase kinase, alpha 2 (liver) (PHKA2), mRNA. | 3.847551067 | 140 | 122 | 125 | 120 | 188 | 205 | 119 | 36 | 38 | 40 | 36 | 38 | 38 |
| 12752815 | PHLPP2 | PH domain and leucine rich repeat protein phosphatase 2 (PHLPP2), mRNA. | 3.270227867 | 737 | 766 | 700 | 912 | 521 | 468 | 415 | 161 | 177 | 123 | 220 | 268 | 235 |
| 12854547 | PHTF2 | putative homeodomain transcription factor 2 (PHTF2), mRNA. | 2.198664948 | 475 | 511 | 569 | 603 | 459 | 405 | 475 | 210 | 233 | 243 | 209 | 237 | 231 |
| 12697186 | PIGB | phosphatidylinositol glycan anchor biosynthesis, class B (PIGB), mRNA. | 3.568251078 | 395 | 437 | 380 | 463 | 191 | 161 | 238 | 93 | 73 | 82 | 109 | 56 | 132 |
| 12786990 | PIK3R1 | phosphoinositide-3-kinase, regulatory subunit 1 (alpha) (PIK3R1), mRNA. | 2.673011814 | 2564 | 2689 | 2541 | 2959 | 1720 | 1638 | 1605 | 756 | 792 | 630 | 1045 | 968 | 849 |
| 12702080 | PKDCC | protein kinase domain containing, cytoplasmic homolog (mouse) (PKDCC), mRNA. | 2.004332717 | 421 | 364 | 259 | 298 | 403 | 305 | 286 | 160 | 193 | 114 | 133 | 258 | 141 |
| 12713650 | PLCB4 | phospholipase C, beta 4 (PLCB4), mRNA. | 3.880871418 | 1366 | 1521 | 1186 | 1989 | 858 | 974 | 765 | 356 | 432 | 369 | 226 | 287 | 242 |
| 12680375 | PLD1 | phospholipase D1, phosphatidylcholine-specific (PLD1), mRNA. | 3.106985585 | 1275 | 1460 | 1243 | 1186 | 1851 | 1934 | 2467 | 356 | 469 | 397 | 402 | 566 | 960 |
| 12824248 | PLEKHA2 | pleckstrin homology domain containing, family A (phosphoinositide binding specific) member 2, mRNA (cDNA clone MGC:128862 IMAGE:8119745), complete cds. | 2.068193481 | 357 | 360 | 380 | 402 | 266 | 251 | 264 | 185 | 157 | 150 | 195 | 131 | 126 |
| 12866960 | PLEKHA5 | Pleckstrin Homology Domain Containing, Family A Member 5 | 2.193411267 | 137 | 157 | 155 | 161 | 110 | 129 | 137 | 57 | 83 | 42 | 70 | 61 | 73 |
| 12698109 | PLEKHH2 | Pleckstrin Homology Domain Containing, Family H (With MyTH4 Domain) Member 2 | 3.700224843 | 124 | 142 | 109 | 149 | 344 | 527 | 353 | 58 | 54 | 56 | 75 | 70 | 91 |
| 12813690 | PLK1 | Polo-Like Kinase 1 | 4.452101992 | 402 | 349 | 388 | 305 | 228 | 199 | 172 | 73 | 83 | 54 | 51 | 70 | 63 |
| 12746604 | PLK4 | polo-like kinase 4 (PLK4), mRNA. | 9.895056974 | 662 | 696 | 612 | 639 | 198 | 141 | 215 | 47 | 52 | 40 | 45 | 46 | 44 |
| 12796403 | POC1A | POC1 centriolar protein homolog A (Chlamydomonas) (POC1A), mRNA. | 3.076509928 | 239 | 236 | 204 | 189 | 147 | 112 | 92 | 66 | 52 | 58 | 52 | 63 | 48 |
| 12744811 | POLE | polymerase (DNA directed), epsilon, catalytic subunit (POLE), mRNA. | 4.267649226 | 205 | 191 | 142 | 102 | 81 | 57 | 74 | 31 | 27 | 22 | 28 | 25 | 39 |
| 12685836 | POLQ | PREDICTED: polymerase (DNA directed), theta (POLQ), mRNA. | 8.076978059 | 277 | 282 | 234 | 259 | 81 | 69 | 76 | 22 | 18 | 25 | 19 | 28 | 23 |
| 12683883 | POLR2H | polymerase (RNA) II (DNA directed) polypeptide H (POLR2H), mRNA. | 2.084207321 | 178 | 207 | 186 | 221 | 202 | 179 | 161 | 81 | 90 | 101 | 102 | 79 | 96 |
| 12896203 | POPDC3 | popeye domain containing 3 (POPDC3), mRNA. | 12.68821348 | 258 | 326 | 206 | 418 | 63 | 32 | 46 | 20 | 15 | 13 | 17 | 16 | 11 |
| 12797985 | PPARG | peroxisome proliferator-activated receptor gamma (PPARG), mRNA. | 11.78769304 | 560 | 599 | 284 | 724 | 127 | 107 | 107 | 29 | 32 | 47 | 36 | 17 | 21 |
| 12800563 | PPARG-TSEN2 | PPARG-TSEN2 (PPARG-TSEN2), transcript variant 1, non-coding RNA. | 9.710591682 | 465 | 479 | 198 | 464 | 96 | 85 | 63 | 30 | 22 | 29 | 34 | 32 | 16 |
| 12872195 | PPM1K | protein phosphatase, Mg2+/Mn2+ dependent, 1K (PPM1K), nuclear gene encoding mitochondrial protein, mRNA. | 3.07531713 | 676 | 698 | 1245 | 967 | 489 | 748 | 614 | 258 | 243 | 351 | 177 | 204 | 282 |
| 12902136 | PPP1R12B | PREDICTED: protein phosphatase 1, regulatory subunit 12B (PPP1R12B), partial mRNA. | 2.004404533 | 167 | 152 | 164 | 133 | 103 | 109 | 118 | 61 | 68 | 73 | 71 | 82 | 50 |
| 12759234 | PPP1R14A | protein phosphatase 1, regulatory (inhibitor) subunit 14A (PPP1R14A), mRNA. | 3.97473812 | 233 | 244 | 233 | 250 | 108 | 95 | 109 | 60 | 38 | 56 | 46 | 32 | 42 |
| 12862788 | PPP1R1A | Protein Phosphatase 1, Regulatory (Inhibitor) Subunit 1A | 4.795520605 | 167 | 149 | 185 | 126 | 435 | 709 | 573 | 56 | 75 | 51 | 70 | 83 | 85 |
| 12730475 | PPP2R1B | Protein Phosphatase 2, Regulatory Subunit A, Beta | 2.283087412 | 699 | 727 | 653 | 652 | 503 | 537 | 590 | 275 | 247 | 278 | 281 | 272 | 284 |
| 12791280 | PRC1 | protein regulator of cytokinesis 1 (PRC1), mRNA. | 10.23393987 | 467 | 529 | 595 | 475 | 161 | 132 | 155 | 44 | 36 | 28 | 39 | 36 | 28 |
| 12835988 | PRDX1 | peroxiredoxin 1 (PRDX1), mRNA. | 2.004107136 | 1091 | 1092 | 1214 | 1477 | 1270 | 874 | 1412 | 578 | 588 | 604 | 678 | 526 | 630 |
| 12858052 | PRIM1 | primase, DNA, polypeptide 1 (49kDa) (PRIM1), mRNA. | 3.688743551 | 475 | 515 | 350 | 354 | 157 | 166 | 150 | 77 | 75 | 77 | 70 | 97 | 107 |
| 12805638 | PRIM2 | primase, DNA, polypeptide 2 (58kDa) (PRIM2), mRNA. | 2.196726311 | 272 | 259 | 216 | 200 | 166 | 135 | 168 | 83 | 83 | 72 | 114 | 102 | 98 |
| 12781370 | PRKAG3 | protein kinase, AMP-activated, gamma 3 non-catalytic subunit (PRKAG3), transcript variant 1, mRNA. | 6.332047351 | 141 | 104 | 87 | 100 | 635 | 726 | 473 | 65 | 58 | 44 | 43 | 53 | 45 |
| 12849057 | PRKAR2B | Protein Kinase, CAMP-Dependent, Regulatory, Type II, Beta | 4.306897968 | 1684 | 1861 | 1769 | 2140 | 1317 | 1246 | 1110 | 259 | 223 | 211 | 513 | 620 | 389 |
| 12793185 | PRKD1 | PREDICTED: protein kinase D1 (PRKD1), mRNA. | 2.547891169 | 961 | 999 | 1109 | 1455 | 679 | 582 | 696 | 375 | 437 | 464 | 270 | 262 | 372 |
| 12774650 | PRKRA | protein kinase, interferon-inducible double stranded RNA dependent activator (PRKRA), mRNA. | 2.313958038 | 113 | 128 | 159 | 180 | 78 | 81 | 93 | 64 | 49 | 51 | 50 | 54 | 40 |
| 12905259 | PRPS2 | phosphoribosyl pyrophosphate synthetase 2 (PRPS2), mRNA. | 4.486437442 | 354 | 334 | 271 | 681 | 985 | 915 | 819 | 117 | 149 | 130 | 131 | 138 | 168 |
| 12765398 | PRR11 | proline rich 11 (PRR11), mRNA. | 6.992568529 | 251 | 295 | 317 | 237 | 91 | 68 | 73 | 27 | 31 | 31 | 27 | 16 | 30 |
| 12855425 | PRR15 | proline rich 15 (PRR15), mRNA. | 4.124154279 | 239 | 239 | 291 | 235 | 95 | 86 | 77 | 43 | 42 | 35 | 44 | 53 | 46 |
| 12898870 | PRSS35 | protease, serine, 35 (PRSS35), mRNA. | 4.272365338 | 205 | 193 | 115 | 202 | 85 | 147 | 106 | 56 | 30 | 25 | 34 | 46 | 21 |
| 12789704 | PSMA4 | proteasome (prosome, macropain) subunit, alpha type, 4 (PSMA4), mRNA. | 2.591110016 | 1065 | 1537 | 1958 | 1772 | 1295 | 1328 | 1628 | 509 | 611 | 595 | 620 | 549 | 617 |
| 12764414 | PSMB3 | proteasome (prosome, macropain) subunit, beta type, 3 (PSMB3), mRNA. | 2.027390642 | 1993 | 1931 | 1988 | 2312 | 2585 | 1920 | 2291 | 1143 | 1108 | 1208 | 1037 | 857 | 998 |
| 12808197 | PSMG2 | proteasome (prosome, macropain) assembly chaperone 2 (PSMG2), mRNA. | 2.810358536 | 628 | 793 | 690 | 862 | 374 | 632 | 560 | 212 | 188 | 210 | 266 | 206 | 301 |
| 12841028 | PSRC1 | proline/serine-rich coiled-coil 1 (PSRC1), mRNA. | 2.945831191 | 159 | 144 | 168 | 139 | 102 | 83 | 75 | 47 | 36 | 52 | 44 | 37 | 38 |
| 12729909 | PTPMT1 | protein tyrosine phosphatase, mitochondrial 1 (PTPMT1), nuclear gene encoding mitochondrial protein, mRNA. | 2.600680062 | 365 | 311 | 323 | 410 | 349 | 263 | 209 | 127 | 101 | 100 | 151 | 122 | 134 |
| 12698608 | PTPN13 | Protein Tyrosine Phosphatase, Non-Receptor Type 13 (APO-1/CD95 (Fas)-Associated Phosphatase) | 2.892006919 | 1069 | 1139 | 1267 | 1552 | 522 | 739 | 678 | 354 | 348 | 364 | 330 | 245 | 423 |
| 12776378 | PTPN4 | protein tyrosine phosphatase, non-receptor type 4 (megakaryocyte) (PTPN4), mRNA. | 2.392324554 | 477 | 666 | 469 | 599 | 481 | 434 | 483 | 217 | 159 | 191 | 272 | 261 | 194 |
| 12891005 | PTPRD | Protein Tyrosine Phosphatase, Receptor Type, D | 4.689575736 | 509 | 521 | 468 | 762 | 196 | 213 | 232 | 110 | 92 | 129 | 43 | 41 | 115 |
| 12702823 | PTRHD1 | peptidyl-tRNA hydrolase domain containing 1 (PTRHD1), mRNA. | 2.058103287 | 182 | 208 | 215 | 216 | 196 | 329 | 253 | 86 | 88 | 98 | 127 | 125 | 141 |
| 12891786 | R3HCC1 | R3H Domain And Coiled-Coil Containing 1 | 2.451656467 | 239 | 275 | 467 | 350 | 267 | 226 | 245 | 116 | 131 | 134 | 114 | 111 | 116 |
| 12697094 | RAB15 | RAB15, member RAS onocogene family (RAB15), mRNA. | 3.05659022 | 394 | 358 | 336 | 487 | 221 | 162 | 139 | 118 | 84 | 104 | 99 | 103 | 80 |
| 12807534 | RAB27B | RAB27B, member RAS oncogene family (RAB27B), mRNA. | 5.165094917 | 319 | 430 | 255 | 373 | 128 | 116 | 111 | 62 | 56 | 43 | 40 | 24 | 63 |
| 12681097 | RAB6B | RAB6B, member RAS oncogene family (RAB6B), mRNA. | 2.0305663 | 317 | 273 | 215 | 215 | 268 | 174 | 182 | 126 | 107 | 114 | 135 | 131 | 80 |
| 12812817 | RABL5 | RAB, member RAS oncogene family-like 5 (RABL5), mRNA. | 2.320527871 | 476 | 495 | 476 | 409 | 236 | 278 | 330 | 158 | 163 | 161 | 168 | 202 | 146 |
| 12859556 | RACGAP1 | PREDICTED: Rac GTPase activating protein 1, transcript variant 1 (RACGAP1), mRNA. | 5.853923436 | 728 | 692 | 635 | 466 | 232 | 219 | 266 | 67 | 84 | 68 | 106 | 80 | 69 |
| 12689358 | RAD51 | RAD51 homolog (S. cerevisiae) (RAD51), mRNA. | 4.300359134 | 293 | 282 | 212 | 167 | 100 | 68 | 109 | 42 | 44 | 32 | 50 | 40 | 37 |
| 12868512 | RAD51AP1 | RAD51 associated protein 1 (RAD51AP1), mRNA. | 3.543351235 | 150 | 155 | 121 | 142 | 67 | 59 | 54 | 32 | 32 | 25 | 24 | 39 | 29 |
| 12842304 | RAD54L | RAD54-like (S. cerevisiae) (RAD54L), mRNA. | 3.802921048 | 193 | 186 | 153 | 133 | 79 | 64 | 68 | 32 | 41 | 27 | 29 | 31 | 38 |
| 12873282 | RAP1GDS1 | RAP1, GTP-GDP dissociation stimulator 1 (RAP1GDS1), mRNA. | 2.358369538 | 314 | 372 | 442 | 424 | 229 | 238 | 204 | 131 | 155 | 151 | 134 | 110 | 126 |
| 12897599 | RARS2 | arginyl-tRNA synthetase 2, mitochondrial (RARS2), nuclear gene encoding mitochondrial protein, mRNA. | 2.835412067 | 304 | 329 | 288 | 379 | 252 | 229 | 227 | 76 | 105 | 74 | 132 | 115 | 106 |
| 12871419 | RASL11B | RAS-like, family 11, member B (RASL11B), mRNA. | 3.393325517 | 262 | 238 | 91 | 198 | 192 | 187 | 142 | 56 | 56 | 62 | 36 | 55 | 65 |
| 12845288 | RAVER2 | Ribonucleoprotein, PTB-Binding 2 | 5.551564587 | 498 | 599 | 414 | 810 | 320 | 479 | 249 | 63 | 52 | 24 | 109 | 177 | 94 |
| 12809623 | RBBP8 | retinoblastoma binding protein 8 (RBBP8), mRNA. | 2.94704194 | 498 | 621 | 472 | 687 | 233 | 247 | 240 | 165 | 135 | 154 | 126 | 142 | 150 |
| 12847143 | RBM15 | RNA Binding Motif Protein 15 | 2.210492853 | 144 | 172 | 136 | 140 | 123 | 105 | 102 | 53 | 51 | 58 | 70 | 70 | 56 |
| 12782928 | RCC1 | regulator of chromosome condensation 1 (RCC1), mRNA. | 2.134188701 | 201 | 195 | 181 | 141 | 124 | 111 | 103 | 59 | 69 | 76 | 77 | 78 | 65 |
| 12860884 | RECQL | RecQ protein-like (DNA helicase Q1-like) (RECQL), mRNA. | 3.000649006 | 279 | 332 | 242 | 271 | 119 | 123 | 143 | 62 | 71 | 53 | 61 | 90 | 94 |
| 12894072 | REEP4 | receptor accessory protein 4 (REEP4), mRNA. | 2.789932895 | 202 | 210 | 206 | 191 | 107 | 85 | 87 | 57 | 67 | 44 | 58 | 56 | 52 |
| 12879664 | REEP6 | receptor accessory protein 6 (REEP6), mRNA. | 2.594579955 | 165 | 135 | 135 | 76 | 92 | 70 | 75 | 45 | 44 | 45 | 42 | 37 | 34 |
| 12678992 | RFC4 | replication factor C (activator 1) 4, 37kDa (RFC4), mRNA. | 3.962061086 | 532 | 456 | 333 | 391 | 172 | 124 | 164 | 74 | 77 | 63 | 86 | 91 | 78 |
| 12746075 | RFC5 | replication factor C (activator 1) 5, 36.5kDa (RFC5), mRNA. | 2.049209414 | 623 | 588 | 553 | 405 | 427 | 426 | 401 | 214 | 224 | 232 | 263 | 245 | 254 |
| 12788381 | RGNEF | 190 kDa guanine nucleotide exchange factor (RGNEF), mRNA. | 10.08791631 | 1326 | 1376 | 1156 | 1278 | 547 | 398 | 398 | 143 | 127 | 101 | 68 | 62 | 50 |
| 12899705 | RGS17 | regulator of G-protein signaling 17 (RGS17), mRNA. | 19.20071617 | 406 | 506 | 288 | 512 | 68 | 74 | 82 | 13 | 16 | 12 | 17 | 14 | 14 |
| 12842845 | RHBG | Rh family, B glycoprotein (gene/pseudogene) (RHBG), mRNA. | 6.155758126 | 117 | 103 | 64 | 98 | 135 | 107 | 66 | 15 | 14 | 20 | 20 | 14 | 13 |
| 12783964 | RIF1 | PREDICTED: RAP1 interacting factor homolog (yeast), transcript variant 2 (RIF1), mRNA. | 2.505974404 | 443 | 534 | 498 | 690 | 260 | 297 | 299 | 153 | 160 | 171 | 173 | 181 | 195 |
| 12867705 | RIMKLB | ribosomal modification protein rimK-like family member B (RIMKLB), mRNA. | 2.503476755 | 231 | 324 | 298 | 383 | 229 | 237 | 174 | 96 | 81 | 103 | 85 | 131 | 145 |
| 12885141 | RNASEH2A | ribonuclease H2, subunit A (RNASEH2A), mRNA. | 2.634855845 | 300 | 272 | 244 | 254 | 230 | 167 | 139 | 126 | 69 | 99 | 66 | 81 | 81 |
| 12833086 | RNASEH2C | ribonuclease H2, subunit C (RNASEH2C), mRNA. | 3.247285845 | 1717 | 1492 | 1418 | 679 | 974 | 979 | 1089 | 331 | 370 | 303 | 376 | 436 | 388 |
| 12772834 | RNFT1 | ring finger protein, transmembrane 1 (RNFT1), mRNA. | 2.169708409 | 925 | 1098 | 885 | 1539 | 731 | 554 | 620 | 398 | 378 | 404 | 457 | 420 | 453 |
| 12847867 | RNPC3 | RNA-binding region (RNP1, RRM) containing 3 (RNPC3), mRNA. | 2.389890701 | 279 | 368 | 341 | 491 | 247 | 337 | 289 | 93 | 166 | 140 | 115 | 156 | 174 |
| 12681539 | ROBO2 | Roundabout Guidance Receptor 2 | 2.415480429 | 1039 | 1148 | 1182 | 1004 | 933 | 798 | 902 | 286 | 378 | 249 | 526 | 494 | 552 |
| 12892173 | ROR2 | receptor tyrosine kinase-like orphan receptor 2 (ROR2), mRNA. | 11.16343326 | 588 | 644 | 388 | 704 | 194 | 161 | 164 | 34 | 43 | 47 | 32 | 34 | 29 |
| 12853992 | RPA3 | replication protein A3, 14kDa (RPA3), mRNA. | 3.623925277 | 987 | 1005 | 654 | 1095 | 595 | 240 | 401 | 223 | 170 | 216 | 211 | 170 | 187 |
| 12781418 | RPL11 | ribosomal protein L11 (RPL11), mRNA. | 2.056934807 | 1335 | 1313 | 1236 | 1401 | 2337 | 2001 | 1877 | 805 | 714 | 682 | 857 | 913 | 822 |
| 12750457 | RPL13 | ribosomal protein L13 (RPL13), mRNA. | 2.678530979 | 440 | 371 | 323 | 432 | 875 | 632 | 467 | 179 | 174 | 125 | 224 | 245 | 186 |
| 12883492 | RPL18A | ribosomal protein L18a (RPL18A), mRNA. | 2.038646283 | 4308 | 4057 | 3771 | 2593 | 5154 | 4032 | 3688 | 1701 | 1815 | 1586 | 2041 | 2423 | 2039 |
| 12725262 | RPL23A | Ribosomal Protein L23a | 2.585426922 | 494 | 550 | 466 | 584 | 708 | 654 | 539 | 163 | 200 | 209 | 175 | 289 | 289 |
| 12869612 | RPL23A | ribosomal protein L23a (RPL23A), mRNA. | 2.527992022 | 491 | 549 | 465 | 593 | 715 | 653 | 533 | 164 | 206 | 213 | 177 | 299 | 297 |
| 12683400 | RPL24 | ribosomal protein L24 (RPL24), mRNA. | 2.561198929 | 115 | 81 | 66 | 212 | 119 | 116 | 129 | 37 | 44 | 41 | 52 | 61 | 47 |
| 12786132 | RPL26L1 | ribosomal protein L26-like 1 (RPL26L1), mRNA. | 2.450851721 | 132 | 156 | 107 | 206 | 102 | 87 | 101 | 48 | 52 | 54 | 60 | 54 | 43 |
| 12761361 | RPL27 | ribosomal protein L27 (RPL27), mRNA. | 2.555009051 | 212 | 233 | 219 | 257 | 486 | 403 | 364 | 114 | 96 | 97 | 139 | 156 | 127 |
| 12701406 | RPL31 | Ribosomal Protein L31 | 2.10356087 | 971 | 1014 | 769 | 1382 | 1434 | 1301 | 1163 | 526 | 433 | 546 | 566 | 644 | 560 |
| 12873633 | RPL34 | ribosomal protein L34 (RPL34), mRNA. | 2.806388837 | 1310 | 1318 | 1304 | 1969 | 2019 | 1965 | 1729 | 550 | 468 | 503 | 666 | 684 | 677 |
| 12906453 | RPL39 | ribosomal protein L39 (RPL39), mRNA. | 2.16689994 | 1189 | 1331 | 1068 | 1595 | 2486 | 2256 | 2070 | 675 | 610 | 730 | 890 | 1011 | 828 |
| 12725267 | RPL7 | ribosomal protein L7 (RPL7), mRNA. | 2.155751439 | 510 | 398 | 389 | 795 | 618 | 516 | 569 | 190 | 258 | 220 | 315 | 220 | 306 |
| 12725721 | RPL8 | ribosomal protein L8 (RPL8), mRNA. | 2.112829433 | 1445 | 1499 | 975 | 1459 | 2226 | 2305 | 1852 | 732 | 648 | 653 | 924 | 1019 | 796 |
| 12756387 | RPS16 | ribosomal protein S16 (RPS16), mRNA. | 2.045345888 | 3161 | 2645 | 2414 | 3095 | 5949 | 5417 | 4509 | 1837 | 1798 | 1621 | 1999 | 2080 | 2058 |
| 12757414 | RPS19 | Ribosomal Protein S19 | 2.013155396 | 1887 | 1779 | 1567 | 1237 | 2804 | 2688 | 2540 | 868 | 916 | 888 | 1029 | 1332 | 1142 |
| 12720857 | RPS21 | Ribosomal Protein S21 | 2.49956004 | 427 | 416 | 324 | 468 | 1135 | 842 | 720 | 250 | 226 | 217 | 248 | 290 | 255 |
| 12865092 | RPS26 | ribosomal protein S26 (RPS26), mRNA. | 2.746926772 | 127 | 118 | 100 | 175 | 157 | 55 | 92 | 36 | 36 | 39 | 50 | 52 | 45 |
| 12847493 | RPS27 | ribosomal protein S27 (RPS27), transcript variant 2, non-coding RNA. | 2.962614344 | 589 | 658 | 337 | 925 | 352 | 469 | 308 | 171 | 193 | 145 | 136 | 201 | 206 |
| 12877215 | RPS28 | ribosomal protein S28 (RPS28), mRNA. | 2.4706852 | 938 | 711 | 672 | 648 | 1185 | 1175 | 1017 | 334 | 303 | 347 | 353 | 439 | 427 |
| 12750150 | RPS5 | ribosomal protein S5 (RPS5), mRNA. | 3.586381699 | 651 | 627 | 480 | 735 | 1943 | 2223 | 1996 | 299 | 283 | 262 | 346 | 436 | 443 |
| 12904595 | RPS6KA6 | ribosomal protein S6 kinase, 90kDa, polypeptide 6 (RPS6KA6), mRNA. | 3.774955447 | 393 | 472 | 393 | 515 | 200 | 185 | 204 | 83 | 71 | 66 | 79 | 128 | 109 |
| 12740218 | RPS6KC1 | ribosomal protein S6 kinase, 52kDa, polypeptide 1 (RPS6KC1), mRNA. | 2.452280703 | 288 | 333 | 318 | 440 | 257 | 253 | 213 | 139 | 130 | 127 | 128 | 108 | 104 |
| 12892930 | RPS7 | ribosomal protein S7 (RPS7), mRNA. | 2.186420519 | 942 | 975 | 692 | 1122 | 1465 | 1628 | 1570 | 491 | 508 | 476 | 576 | 668 | 570 |
| 12728269 | RRAS2 | related RAS viral (r-ras) oncogene homolog 2 (RRAS2), mRNA. | 3.3018399 | 542 | 535 | 499 | 628 | 304 | 326 | 340 | 170 | 184 | 207 | 81 | 101 | 81 |
| 12731246 | RRM1 | ribonucleotide reductase M1 (RRM1), mRNA. | 3.032117433 | 760 | 701 | 687 | 584 | 354 | 319 | 385 | 207 | 182 | 172 | 154 | 182 | 174 |
| 12704737 | RRM2 | ribonucleotide reductase M2 (RRM2), transcript variant 1, mRNA. | 10.68218174 | 513 | 467 | 439 | 351 | 248 | 191 | 247 | 34 | 37 | 22 | 43 | 34 | 28 |
| 12871032 | RUFY3 | RUN and FYVE domain containing 3 (RUFY3), mRNA. | 2.17296871 | 313 | 331 | 390 | 328 | 242 | 270 | 310 | 151 | 147 | 140 | 113 | 155 | 156 |
| 12682656 | RYK | PREDICTED: RYK receptor-like tyrosine kinase (RYK), partial mRNA. | 5.86510831 | 3059 | 3097 | 2939 | 3307 | 2227 | 2086 | 1995 | 435 | 466 | 434 | 505 | 420 | 474 |
| 12726998 | SAAL1 | serum amyloid A-like 1 (SAAL1), mRNA. | 2.518565509 | 187 | 193 | 213 | 238 | 197 | 232 | 235 | 89 | 84 | 78 | 89 | 78 | 91 |
| 12706167 | SCAI | suppressor of cancer cell invasion (SCAI), mRNA. | 4.134403006 | 329 | 376 | 379 | 538 | 182 | 213 | 211 | 56 | 75 | 63 | 91 | 78 | 99 |
| 12904467 | SCML2 | sex comb on midleg-like 2 (Drosophila) (SCML2), mRNA. | 6.253670941 | 186 | 198 | 199 | 171 | 98 | 105 | 115 | 10 | 25 | 17 | 29 | 31 | 35 |
| 12791715 | SEC11A | SEC11 Homolog A, Signal Peptidase Complex Subunit | 2.32927187 | 960 | 927 | 773 | 1375 | 979 | 861 | 809 | 342 | 328 | 293 | 482 | 559 | 457 |
| 12695131 | SEMA6D | sema domain, transmembrane domain (TM), and cytoplasmic domain, (semaphorin) 6D (SEMA6D), mRNA. | 4.371395913 | 2225 | 2417 | 2403 | 2559 | 823 | 871 | 807 | 470 | 348 | 360 | 417 | 430 | 348 |
| 12871784 | SEPT11 | septin 11 (SEPT11), mRNA. | 3.693372269 | 457 | 460 | 527 | 402 | 283 | 390 | 378 | 169 | 147 | 159 | 84 | 57 | 55 |
| 12728123 | SESN3 | Sestrin 3 | 2.390884902 | 123 | 159 | 180 | 213 | 413 | 441 | 379 | 112 | 128 | 104 | 103 | 134 | 104 |
| 12685821 | SETMAR | SET domain and mariner transposase fusion gene (SETMAR), mRNA. | 2.18756376 | 1699 | 1735 | 1448 | 1405 | 1129 | 1191 | 1192 | 491 | 612 | 492 | 730 | 756 | 759 |
| 12771261 | SGK494 | Uncharacterized Serine/Threonine-Protein Kinase SgK494 | 2.071978924 | 160 | 140 | 152 | 104 | 113 | 116 | 101 | 53 | 72 | 64 | 49 | 70 | 58 |
| 12686668 | SGOL1 | shugoshin-like 1 (S. pombe) (SGOL1), mRNA. | 6.583861993 | 390 | 415 | 329 | 377 | 130 | 124 | 115 | 38 | 37 | 42 | 42 | 24 | 62 |
| 12777652 | SGOL2 | shugoshin-like 2 (S. pombe) (SGOL2), mRNA. | 3.102540026 | 244 | 332 | 263 | 317 | 125 | 132 | 133 | 74 | 73 | 104 | 43 | 71 | 61 |
| 12743852 | SH3D19 | PREDICTED: SH3 domain containing 19, transcript variant 3 (SH3D19), mRNA. | 3.031490354 | 1266 | 1332 | 1131 | 1427 | 930 | 775 | 719 | 381 | 374 | 424 | 332 | 290 | 343 |
| 12837079 | SIKE1 | suppressor of IKBKE 1 (SIKE1), mRNA. | 2.285542237 | 365 | 419 | 278 | 351 | 251 | 223 | 243 | 123 | 134 | 136 | 134 | 128 | 144 |
| 12772810 | SKA2 | spindle and kinetochore associated complex subunit 2 (SKA2), mRNA. | 3.925314299 | 170 | 218 | 132 | 151 | 141 | 128 | 118 | 29 | 28 | 19 | 55 | 52 | 49 |
| 12709472 | SKA3 | spindle and kinetochore associated complex subunit 3 (SKA3), mRNA. | 4.463074382 | 331 | 334 | 335 | 269 | 132 | 149 | 146 | 37 | 54 | 54 | 66 | 55 | 60 |
| 12787593 | SKP2 | S-phase kinase-associated protein 2 (p45) (SKP2), mRNA. | 3.075738634 | 369 | 341 | 335 | 240 | 171 | 189 | 188 | 90 | 95 | 74 | 74 | 102 | 75 |
| 12898333 | SLC22A3 | PREDICTED: solute carrier family 22 (extraneuronal monoamine transporter), member 3 (SLC22A3), mRNA. | 4.450662069 | 327 | 339 | 238 | 529 | 177 | 213 | 180 | 80 | 69 | 98 | 47 | 46 | 46 |
| 12902960 | SLC25A5 | solute carrier family 25 (mitochondrial carrier; adenine nucleotide translocator), member 5 (SLC25A5), nuclear gene encoding mitochondrial protein, mRNA. | 2.470215118 | 1471 | 1490 | 1130 | 1707 | 1279 | 1341 | 1271 | 617 | 556 | 561 | 639 | 523 | 466 |
| 12885340 | SLC27A1 | solute carrier family 27 (fatty acid transporter), member 1 (SLC27A1), mRNA. | 2.793154206 | 213 | 243 | 203 | 240 | 399 | 244 | 266 | 67 | 73 | 64 | 113 | 98 | 139 |
| 12844142 | SLC27A3 | PREDICTED: solute carrier family 27 (fatty acid transporter), member 3 (SLC27A3), mRNA. | 11.45536306 | 347 | 406 | 358 | 534 | 162 | 144 | 166 | 23 | 24 | 25 | 21 | 35 | 30 |
| 12855695 | SLC35B4 | solute carrier family 35, member B4 (SLC35B4), mRNA. | 2.354882572 | 206 | 216 | 237 | 178 | 184 | 144 | 160 | 117 | 90 | 100 | 47 | 61 | 67 |
| 12821015 | SLC35G1 | solute carrier family 35, member G1 (SLC35G1), mRNA. | 18.5662491 | 2046 | 2026 | 1756 | 1966 | 896 | 625 | 987 | 131 | 82 | 122 | 63 | 26 | 52 |
| 12860556 | SLC38A1 | Solute Carrier Family 38, Member 1 | 5.946548574 | 1771 | 1825 | 1345 | 1692 | 641 | 507 | 572 | 264 | 212 | 269 | 167 | 181 | 111 |
| 12843700 | SLC44A3 | solute carrier family 44, member 3 (SLC44A3), mRNA. | 3.93463242 | 480 | 491 | 458 | 529 | 209 | 163 | 169 | 72 | 58 | 60 | 124 | 137 | 93 |
| 12777931 | SLC4A3 | solute carrier family 4, anion exchanger, member 3 (SLC4A3), mRNA. | 7.187133821 | 975 | 974 | 964 | 663 | 578 | 501 | 542 | 77 | 65 | 73 | 101 | 192 | 112 |
| 12683494 | SLC5A3 | solute carrier family 5 (sodium/myo-inositol cotransporter), member 3 (SLC5A3), mRNA. | 4.210791916 | 3559 | 3519 | 3845 | 3323 | 2175 | 1489 | 1609 | 780 | 802 | 897 | 458 | 528 | 509 |
| 12791856 | SLCO3A1 | solute carrier organic anion transporter family, member 3A1 (SLCO3A1), mRNA. | 4.806505862 | 1110 | 1003 | 960 | 735 | 328 | 329 | 230 | 178 | 208 | 179 | 91 | 65 | 116 |
| 12909850 | SLITRK2 | PREDICTED: SLIT and NTRK-like family, member 2 (SLITRK2), mRNA. | 7.006155435 | 313 | 353 | 225 | 260 | 210 | 87 | 210 | 46 | 51 | 39 | 19 | 28 | 20 |
| 12809770 | SMAD2 | SMAD family member 2 (SMAD2), mRNA. | 3.415086506 | 2198 | 2416 | 2048 | 2629 | 948 | 765 | 811 | 524 | 454 | 473 | 541 | 463 | 511 |
| 12891472 | SMC2 | Structural Maintenance Of Chromosomes 2 | 5.69110295 | 778 | 830 | 657 | 597 | 252 | 235 | 288 | 90 | 103 | 70 | 119 | 77 | 88 |
| 12683595 | SMC4 | structural maintenance of chromosomes 4, mRNA (cDNA clone MGC:140334 IMAGE:8187023), complete cds. | 2.944594549 | 609 | 753 | 533 | 660 | 234 | 217 | 258 | 149 | 164 | 159 | 152 | 156 | 170 |
| 12785096 | SMN1 | survival of motor neuron 1, telomeric (SMN1), mRNA. | 2.31695801 | 182 | 202 | 188 | 202 | 117 | 132 | 125 | 73 | 80 | 67 | 57 | 81 | 67 |
| 12851559 | SMO | smoothened, frizzled family receptor (SMO), mRNA. | 2.32825587 | 311 | 288 | 399 | 263 | 392 | 360 | 308 | 136 | 157 | 146 | 121 | 168 | 128 |
| 12748161 | SNRPD3 | Small Nuclear Ribonucleoprotein D3 Polypeptide 18kDa | 2.603446539 | 892 | 832 | 864 | 1315 | 553 | 574 | 774 | 232 | 314 | 317 | 314 | 346 | 389 |
| 12736543 | SNRPE | small nuclear ribonucleoprotein polypeptide E (SNRPE), mRNA. | 2.067373786 | 432 | 439 | 408 | 484 | 463 | 405 | 402 | 193 | 221 | 211 | 225 | 221 | 187 |
| 12706195 | SNRPG | small nuclear ribonucleoprotein polypeptide G (SNRPG), mRNA. | 2.224187401 | 341 | 258 | 262 | 370 | 299 | 261 | 295 | 135 | 122 | 135 | 143 | 119 | 149 |
| 12824307 | SNX25 | sorting nexin 25 (SNX25), mRNA. | 2.93004685 | 217 | 224 | 230 | 220 | 110 | 84 | 127 | 60 | 59 | 61 | 62 | 53 | 59 |
| 12825497 | SORBS2 | sorbin and SH3 domain containing 2 (SORBS2), mRNA. | 2.688628112 | 2082 | 2350 | 2182 | 2234 | 1451 | 1543 | 1329 | 563 | 607 | 557 | 641 | 1110 | 721 |
| 12728901 | SORL1 | sortilin-related receptor, L(DLR class) A repeats containing (SORL1), mRNA. | 3.1467725 | 195 | 204 | 254 | 208 | 209 | 220 | 175 | 83 | 56 | 77 | 59 | 57 | 68 |
| 12861001 | SOX5 | SRY (sex determining region Y)-box 5 (SOX5), mRNA. | 2.80906504 | 105 | 162 | 95 | 141 | 92 | 95 | 72 | 46 | 42 | 46 | 35 | 37 | 27 |
| 12768031 | SPAG5 | sperm associated antigen 5 (SPAG5), mRNA. | 6.451973227 | 232 | 236 | 215 | 173 | 113 | 105 | 113 | 20 | 33 | 23 | 27 | 27 | 29 |
| 12745177 | SPATA5 | Spermatogenesis Associated 5 | 2.499466208 | 225 | 204 | 208 | 239 | 150 | 166 | 178 | 85 | 82 | 66 | 73 | 79 | 85 |
| 12884486 | SPC24 | SPC24, NDC80 Kinetochore Complex Component | 6.351281797 | 275 | 285 | 211 | 239 | 178 | 139 | 142 | 29 | 25 | 30 | 31 | 36 | 48 |
| 12775328 | SPC25 | SPC25, NDC80 kinetochore complex component, homolog (S. cerevisiae) (SPC25), mRNA. | 7.026656796 | 158 | 235 | 138 | 177 | 60 | 37 | 45 | 21 | 17 | 14 | 17 | 14 | 20 |
| 12684728 | SPICE1 | spindle and centriole associated protein 1 (SPICE1), mRNA. | 2.019169145 | 182 | 190 | 183 | 191 | 137 | 139 | 124 | 73 | 63 | 75 | 84 | 94 | 97 |
| 12730969 | SPON1 | Spondin 1, Extracellular Matrix Protein | 19.76003737 | 1196 | 1292 | 645 | 1579 | 1233 | 1708 | 1373 | 93 | 56 | 50 | 88 | 59 | 46 |
| 12826119 | SRGN | serglycin (SRGN), mRNA. | 6.335977297 | 5427 | 6222 | 4866 | 7173 | 2934 | 1858 | 1841 | 1233 | 815 | 1101 | 354 | 346 | 252 |
| 12735060 | SRM | PREDICTED: spermidine synthase, transcript variant 1 (SRM), mRNA. | 2.169707886 | 193 | 239 | 172 | 126 | 177 | 125 | 169 | 88 | 74 | 84 | 67 | 94 | 68 |
| 12903267 | SSR4 | signal sequence receptor, delta (SSR4), mRNA. | 2.926456606 | 1853 | 1723 | 1488 | 1475 | 3247 | 3552 | 3274 | 890 | 621 | 804 | 847 | 904 | 800 |
| 12829642 | ST3GAL4 | ST3 beta-galactoside alpha-2,3-sialyltransferase 4 (ST3GAL4), mRNA. | 4.959661942 | 2045 | 2083 | 2769 | 2909 | 1721 | 962 | 1472 | 517 | 377 | 406 | 513 | 338 | 261 |
| 12837565 | STIL | SCL/TAL1 interrupting locus (STIL), mRNA. | 5.765675668 | 301 | 311 | 271 | 256 | 83 | 81 | 97 | 44 | 51 | 31 | 25 | 33 | 24 |
| 12779317 | STMN1 | stathmin 1 (STMN1), mRNA. | 11.28545501 | 1495 | 1636 | 1465 | 1421 | 814 | 815 | 816 | 138 | 141 | 117 | 92 | 82 | 72 |
| 12698011 | STON1 | stonin 1 (STON1), mRNA. | 2.510376691 | 265 | 273 | 270 | 283 | 275 | 284 | 354 | 127 | 95 | 146 | 119 | 90 | 107 |
| 12765663 | STRA13 | stimulated by retinoic acid 13 homolog (mouse) (STRA13), mRNA. | 3.75953024 | 655 | 723 | 666 | 338 | 561 | 374 | 375 | 153 | 150 | 114 | 152 | 128 | 145 |
| 12790079 | STRA6 | Stimulated By Retinoic Acid 6 | 37.15932224 | 1771 | 1713 | 1211 | 1613 | 562 | 339 | 290 | 25 | 39 | 21 | 31 | 32 | 25 |
| 12830042 | STT3A | STT3, subunit of the oligosaccharyltransferase complex, homolog A (S. cerevisiae) (STT3A), mRNA. | 2.035696181 | 2255 | 2333 | 2328 | 2637 | 2073 | 2234 | 2627 | 1242 | 1094 | 1025 | 1276 | 1103 | 1202 |
| 12815257 | STX1B | Syntaxin 1B | 2.90233592 | 89 | 132 | 177 | 150 | 60 | 75 | 62 | 36 | 31 | 42 | 45 | 32 | 34 |
| 12739111 | SUSD4 | sushi domain containing 4 (SUSD4), mRNA. | 19.17407529 | 1137 | 1351 | 1078 | 1098 | 636 | 318 | 428 | 101 | 52 | 31 | 28 | 28 | 31 |
| 12716539 | SUV39H2 | suppressor of variegation 3-9 homolog 2 (Drosophila) (SUV39H2), mRNA. | 3.148181814 | 114 | 126 | 93 | 134 | 59 | 47 | 43 | 25 | 30 | 19 | 34 | 27 | 33 |
| 12800256 | SYNPR | Synaptoporin | 3.03024679 | 191 | 230 | 179 | 296 | 115 | 89 | 81 | 56 | 58 | 47 | 64 | 55 | 54 |
| 12856920 | SYT1 | synaptotagmin I (SYT1), mRNA. | 5.287986724 | 134 | 173 | 199 | 138 | 98 | 198 | 223 | 36 | 18 | 43 | 20 | 44 | 28 |
| 12732926 | SYT9 | PREDICTED: synaptotagmin IX (SYT9), mRNA. | 6.796537168 | 545 | 514 | 707 | 334 | 265 | 132 | 290 | 48 | 79 | 59 | 56 | 45 | 64 |
| 12856932 | TAC3 | tachykinin 3 (TAC3), mRNA. | 3.467191806 | 133 | 170 | 96 | 110 | 51 | 42 | 54 | 25 | 26 | 21 | 28 | 32 | 30 |
| 12706420 | TBC1D8 | TBC1 domain family, member 8 (with GRAM domain) (TBC1D8), mRNA. | 3.575762009 | 548 | 546 | 463 | 526 | 296 | 201 | 169 | 136 | 130 | 130 | 83 | 99 | 81 |
| 12693669 | TBCA | tubulin folding cofactor A (TBCA), mRNA. | 2.150204727 | 355 | 401 | 367 | 638 | 369 | 359 | 342 | 181 | 186 | 214 | 206 | 161 | 181 |
| 12777797 | TCEA3 | transcription elongation factor A (SII), 3 (TCEA3), mRNA. | 4.929981563 | 311 | 279 | 205 | 240 | 180 | 137 | 118 | 52 | 44 | 33 | 36 | 32 | 58 |
| 12910390 | TCEAL8 | transcription elongation factor A (SII)-like 8 (TCEAL8), mRNA. | 2.151994393 | 586 | 732 | 879 | 724 | 596 | 686 | 915 | 290 | 344 | 292 | 388 | 391 | 334 |
| 12805578 | TCF19 | transcription factor 19 (TCF19), mRNA. | 2.464921781 | 219 | 226 | 150 | 108 | 104 | 106 | 104 | 49 | 58 | 60 | 59 | 72 | 56 |
| 12689601 | TDP1 | tyrosyl-DNA phosphodiesterase 1 (TDP1), mRNA. | 3.720121575 | 326 | 339 | 292 | 272 | 197 | 143 | 143 | 66 | 55 | 54 | 82 | 72 | 65 |
| 12712739 | TEX30 | testis expressed 30 (TEX30), mRNA. | 2.375382255 | 113 | 106 | 75 | 109 | 64 | 47 | 56 | 27 | 43 | 30 | 32 | 36 | 38 |
| 12710183 | TFDP1 | transcription factor Dp-1 (TFDP1), mRNA. | 2.042059067 | 228 | 226 | 206 | 171 | 123 | 132 | 128 | 108 | 68 | 78 | 82 | 100 | 73 |
| 12683750 | TFRC | transferrin receptor (p90, CD71) (TFRC), mRNA. | 3.155956464 | 1133 | 1119 | 1078 | 1046 | 486 | 494 | 606 | 264 | 252 | 148 | 362 | 324 | 269 |
| 12716471 | TGIF2 | TGFB-induced factor homeobox 2 (TGIF2), mRNA. | 2.372488976 | 203 | 199 | 223 | 190 | 135 | 108 | 116 | 82 | 74 | 78 | 58 | 62 | 69 |
| 12808881 | THOC1 | THO complex 1 (THOC1), mRNA. | 2.338862449 | 199 | 216 | 200 | 267 | 123 | 135 | 126 | 59 | 82 | 67 | 65 | 83 | 109 |
| 12707288 | TIA1 | TIA1 cytotoxic granule-associated RNA binding protein (TIA1), mRNA. | 2.101157129 | 796 | 853 | 850 | 905 | 733 | 810 | 763 | 335 | 370 | 355 | 331 | 470 | 467 |
| 12733738 | TIMM8B | translocase of inner mitochondrial membrane 8 homolog B (yeast) (TIMM8B), nuclear gene encoding mitochondrial protein, mRNA. | 2.333790028 | 213 | 230 | 161 | 223 | 231 | 188 | 228 | 76 | 84 | 82 | 119 | 88 | 92 |
| 12761510 | TK1 | thymidine kinase 1, soluble (TK1), mRNA. | 11.90851232 | 813 | 842 | 966 | 595 | 204 | 136 | 137 | 61 | 39 | 44 | 50 | 42 | 29 |
| 12821682 | TLL2 | PREDICTED: tolloid-like 2, transcript variant 2 (TLL2), mRNA. | 7.335763632 | 1673 | 1688 | 1714 | 1788 | 598 | 451 | 509 | 137 | 154 | 126 | 204 | 89 | 275 |
| 12875578 | TLR1 | toll-like receptor 1 (TLR1), mRNA. | 3.232995119 | 603 | 652 | 467 | 540 | 307 | 278 | 236 | 96 | 82 | 105 | 159 | 172 | 204 |
| 12873446 | TLR6 | toll-like receptor 6 (TLR6), mRNA. | 5.294134382 | 564 | 549 | 547 | 798 | 375 | 398 | 307 | 92 | 84 | 76 | 109 | 102 | 110 |
| 12888738 | TMEFF1 | transmembrane protein with EGF-like and two follistatin-like domains 1 (TMEFF1), mRNA. | 7.146362382 | 223 | 193 | 196 | 218 | 83 | 99 | 85 | 31 | 20 | 24 | 18 | 18 | 21 |
| 12817058 | TMEM120A | transmembrane protein 120A (TMEM120A), mRNA. | 6.061992855 | 2631 | 2917 | 2526 | 3200 | 1299 | 1073 | 947 | 396 | 334 | 341 | 385 | 243 | 365 |
| 12851703 | TMEM168 | transmembrane protein 168 (TMEM168), mRNA. | 2.179633458 | 347 | 374 | 319 | 494 | 203 | 174 | 237 | 143 | 147 | 133 | 144 | 139 | 140 |
| 12850562 | TMEM178B | PREDICTED: uncharacterized LOC523235 (LOC523235), mRNA. | 5.301961691 | 252 | 246 | 408 | 245 | 151 | 209 | 108 | 58 | 31 | 68 | 33 | 34 | 40 |
| 12830423 | TMEM179B | transmembrane protein 179B (TMEM179B), mRNA. | 2.140983253 | 1743 | 1795 | 1718 | 1563 | 1353 | 1257 | 1190 | 665 | 767 | 612 | 739 | 659 | 809 |
| 12861056 | TMEM194A | transmembrane protein 194A (TMEM194A), mRNA. | 3.641325003 | 575 | 503 | 389 | 452 | 241 | 206 | 193 | 101 | 99 | 109 | 105 | 97 | 91 |
| 12841278 | TMEM48 | transmembrane protein 48 | 3.609184623 | 400 | 368 | 347 | 390 | 173 | 162 | 194 | 66 | 70 | 65 | 97 | 94 | 91 |
| 12847346 | TMEM56 | transmembrane protein 56 | 2.350740913 | 5181 | 5482 | 5418 | 5328 | 3240 | 2556 | 2957 | 1549 | 1318 | 1553 | 2364 | 1951 | 2263 |
| 12770055 | TMEM98 | transmembrane protein 98 | 2.490008591 | 287 | 293 | 287 | 172 | 402 | 229 | 202 | 68 | 89 | 73 | 103 | 185 | 127 |
| 12751034 | TNNI3 | troponin I type 3 (cardiac) (TNNI3), mRNA. | 4.413047234 | 692 | 644 | 651 | 739 | 659 | 393 | 535 | 60 | 57 | 43 | 135 | 341 | 201 |
| 12788447 | TNPO1 | transportin 1 (TNPO1), mRNA. | 3.157307178 | 2659 | 2847 | 2567 | 3504 | 1358 | 1328 | 1396 | 756 | 712 | 679 | 754 | 678 | 673 |
| 12773515 | TOP2A | Topoisomerase (DNA) II Alpha | 39.52289327 | 1294 | 1489 | 1267 | 1295 | 361 | 370 | 420 | 42 | 23 | 18 | 15 | 28 | 15 |
| 12725510 | TOX | thymocyte selection-associated high mobility group box (TOX), mRNA. | 20.59914768 | 1054 | 1040 | 1508 | 982 | 401 | 429 | 385 | 38 | 49 | 42 | 36 | 32 | 43 |
| 12898619 | TPBG | Trophoblast Glycoprotein | 3.920471251 | 209 | 184 | 285 | 258 | 228 | 329 | 298 | 92 | 66 | 108 | 31 | 37 | 58 |
| 12836295 | TPM3 | tropomyosin 3 (TPM3), mRNA. | 6.780897429 | 437 | 402 | 439 | 406 | 157 | 234 | 169 | 42 | 41 | 17 | 71 | 25 | 87 |
| 12714352 | TPX2 | TPX2, Microtubule-Associated | 8.405770805 | 437 | 447 | 420 | 412 | 164 | 201 | 188 | 56 | 42 | 44 | 27 | 36 | 26 |
| 12704157 | TRIB2 | tribbles homolog 2 (Drosophila) (TRIB2), mRNA. | 15.30582562 | 5702 | 5440 | 5074 | 5337 | 3972 | 3640 | 3475 | 459 | 377 | 350 | 213 | 277 | 150 |
| 12864675 | TROAP | trophinin associated protein (tastin) (TROAP), mRNA. | 3.399615363 | 274 | 262 | 273 | 148 | 136 | 109 | 111 | 57 | 60 | 51 | 49 | 52 | 61 |
| 12895096 | TRPM3 | transient receptor potential cation channel, subfamily M, member 3 (TRPM3), mRNA. | 5.365282144 | 220 | 216 | 279 | 225 | 165 | 183 | 167 | 41 | 62 | 34 | 24 | 29 | 43 |
| 12815851 | TRRAP | transformation/transcription domain-associated protein (TRRAP), mRNA. | 2.121875434 | 435 | 437 | 447 | 282 | 241 | 221 | 194 | 144 | 167 | 135 | 136 | 175 | 155 |
| 12684443 | TTC14 | tetratricopeptide repeat domain 14 (TTC14), mRNA. | 2.337632196 | 287 | 294 | 286 | 384 | 202 | 253 | 274 | 118 | 131 | 119 | 110 | 121 | 126 |
| 12862452 | TTC38 | tetratricopeptide repeat domain 38 (TTC38), mRNA. | 2.118126505 | 152 | 189 | 215 | 172 | 147 | 101 | 147 | 81 | 71 | 71 | 71 | 82 | 78 |
| 12846005 | TTF2 | transcription termination factor, RNA polymerase II (TTF2), mRNA. | 2.683703801 | 150 | 139 | 116 | 109 | 71 | 63 | 58 | 35 | 35 | 35 | 40 | 40 | 41 |
| 12806713 | TUBB | tubulin, beta class I (TUBB), mRNA. | 3.673873525 | 1030 | 1213 | 1016 | 905 | 956 | 969 | 994 | 292 | 263 | 251 | 212 | 375 | 259 |
| 12714343 | UBE2C | Ubiquitin-Conjugating Enzyme E2C | 9.200948403 | 1163 | 1028 | 1290 | 790 | 489 | 474 | 523 | 112 | 98 | 105 | 91 | 61 | 70 |
| 12711895 | UGGT2 | PREDICTED: UDP-glucose glycoprotein glucosyltransferase 2 (UGGT2), mRNA. | 4.033467434 | 339 | 343 | 373 | 497 | 155 | 151 | 160 | 69 | 66 | 61 | 86 | 77 | 70 |
| 12886305 | UHRF1 | ubiquitin-like with PHD and ring finger domains 1 (UHRF1), mRNA. | 3.386273179 | 229 | 202 | 205 | 138 | 131 | 105 | 112 | 54 | 44 | 46 | 45 | 49 | 46 |
| 12895239 | UHRF2 | ubiquitin-like with PHD and ring finger domains 2 (UHRF2), mRNA. | 2.157885337 | 254 | 232 | 234 | 254 | 148 | 158 | 163 | 77 | 118 | 85 | 80 | 107 | 106 |
| 12790703 | UNC79 | Unc-79 Homolog (C. Elegans) | 4.249842046 | 107 | 100 | 165 | 110 | 41 | 40 | 60 | 27 | 15 | 29 | 23 | 17 | 16 |
| 12877237 | UQCRQ | ubiquinol-cytochrome c reductase, complex III subunit VII, 9.5kDa (UQCRQ), nuclear gene encoding mitochondrial protein, mRNA. | 2.430437038 | 117 | 123 | 105 | 229 | 185 | 102 | 126 | 61 | 51 | 80 | 71 | 35 | 51 |
| 12760418 | USMG5 | Up-Regulated During Skeletal Muscle Growth 5 Homolog (Mouse) | 2.713473167 | 241 | 290 | 246 | 336 | 630 | 341 | 587 | 151 | 103 | 178 | 153 | 100 | 159 |
| 12844960 | USP1 | ubiquitin specific peptidase 1 (USP1), mRNA. | 2.430746731 | 781 | 799 | 608 | 717 | 441 | 322 | 369 | 220 | 213 | 243 | 271 | 262 | 215 |
| 12845960 | USP40 | PREDICTED: ubiquitin specific peptidase 40 (USP40), mRNA. | 2.677936857 | 357 | 392 | 372 | 466 | 223 | 237 | 219 | 111 | 131 | 106 | 129 | 122 | 128 |
| 12874296 | USP46 | ubiquitin specific peptidase 46 (USP46), mRNA. | 2.508695143 | 826 | 823 | 660 | 745 | 423 | 391 | 418 | 238 | 214 | 167 | 295 | 282 | 268 |
| 12773891 | USP48 | Ubiquitin Specific Peptidase 48 | 2.072241014 | 263 | 283 | 306 | 388 | 225 | 208 | 239 | 127 | 128 | 127 | 132 | 121 | 156 |
| 12908896 | UXT | ubiquitously-expressed transcript (UXT), mRNA. | 2.463914581 | 463 | 492 | 333 | 521 | 807 | 734 | 591 | 219 | 226 | 234 | 242 | 231 | 219 |
| 12877198 | VCAN | versican (VCAN), mRNA. | 41.22245872 | 4532 | 4753 | 5198 | 5325 | 3083 | 2682 | 2643 | 132 | 87 | 82 | 91 | 76 | 120 |
| 12791266 | VRK1 | vaccinia related kinase 1 (VRK1), mRNA. | 4.013438906 | 467 | 446 | 386 | 437 | 181 | 107 | 162 | 92 | 80 | 71 | 84 | 60 | 80 |
| 12826506 | WDFY4 | WDFY family member 4 (WDFY4), mRNA. | 2.700185439 | 156 | 137 | 119 | 100 | 114 | 99 | 67 | 36 | 43 | 46 | 29 | 58 | 39 |
| 12696877 | WDHD1 | WD repeat and HMG-box DNA binding protein 1 (WDHD1), mRNA. | 6.17281667 | 285 | 247 | 175 | 199 | 78 | 78 | 87 | 29 | 29 | 23 | 28 | 24 | 27 |
| 12871651 | WDR19 | WD repeat domain 19 (WDR19), mRNA. | 3.407054043 | 1589 | 1848 | 1438 | 1503 | 711 | 777 | 645 | 285 | 274 | 247 | 386 | 480 | 470 |
| 12752612 | WDR62 | PREDICTED: WD repeat domain 62 (WDR62), mRNA. | 2.409791444 | 125 | 113 | 131 | 82 | 66 | 65 | 53 | 36 | 44 | 31 | 47 | 31 | 37 |
| 12724824 | WDR67 | WD repeat domain 67 (WDR67), mRNA. | 2.992983092 | 408 | 445 | 518 | 628 | 248 | 259 | 236 | 91 | 123 | 86 | 134 | 162 | 190 |
| 12782027 | WDR75 | WD Repeat Domain 75 | 2.18207915 | 451 | 489 | 351 | 426 | 258 | 295 | 298 | 168 | 136 | 151 | 184 | 180 | 189 |
| 12790034 | WDR76 | WD Repeat Domain 76 | 3.507977125 | 168 | 189 | 155 | 206 | 92 | 82 | 99 | 46 | 49 | 40 | 39 | 38 | 30 |
| 12733232 | WEE1 | WEE1 homolog (S. pombe) (WEE1), mRNA. | 2.633331669 | 694 | 773 | 656 | 557 | 284 | 332 | 318 | 164 | 205 | 138 | 228 | 228 | 213 |
| 12906804 | WNK3 | WNK lysine deficient protein kinase 3 (WNK3), mRNA. | 4.608862515 | 106 | 112 | 146 | 107 | 100 | 115 | 95 | 32 | 28 | 32 | 14 | 19 | 20 |
| 12856220 | XRCC2 | X-ray repair complementing defective repair in Chinese hamster cells 2 (XRCC2), mRNA. | 3.446853311 | 169 | 158 | 104 | 114 | 78 | 64 | 69 | 33 | 39 | 20 | 27 | 38 | 29 |
| 12777605 | XRCC5 | X-ray repair complementing defective repair in Chinese hamster cells 5 (double-strand-break rejoining) (XRCC5), mRNA. | 2.061793242 | 382 | 385 | 359 | 432 | 296 | 326 | 257 | 149 | 148 | 117 | 171 | 239 | 189 |
| 12729557 | ZBED5 | zinc finger, BED-type containing 5 (ZBED5), mRNA. | 2.244795898 | 258 | 281 | 284 | 284 | 228 | 296 | 234 | 94 | 107 | 61 | 135 | 183 | 133 |
| 12897316 | ZDHHC14 | zinc finger, DHHC-type containing 14 (ZDHHC14), mRNA. | 3.016869816 | 341 | 285 | 247 | 260 | 155 | 185 | 196 | 86 | 74 | 62 | 68 | 99 | 86 |
| 12814901 | ZKSCAN1 | zinc finger with KRAB and SCAN domains 1 (ZKSCAN1), mRNA. | 4.33771877 | 1267 | 1366 | 932 | 1391 | 706 | 616 | 600 | 228 | 216 | 196 | 247 | 250 | 222 |
| 12827916 | ZMYND17 | zinc finger, MYND-type containing 17 (ZMYND17), mRNA. | 2.623400156 | 114 | 107 | 107 | 96 | 68 | 73 | 69 | 31 | 37 | 20 | 33 | 38 | 49 |
| 12795742 | ZNF197 | zinc finger protein 197 (ZNF197), mRNA. | 2.710269274 | 132 | 112 | 119 | 115 | 86 | 89 | 72 | 39 | 32 | 35 | 41 | 42 | 40 |
| 12742235 | ZNF268 | Zinc Finger Protein 268 | 2.061732153 | 180 | 182 | 175 | 183 | 118 | 138 | 113 | 85 | 91 | 73 | 67 | 50 | 85 |
| 12910670 | ZNF280C | Zinc Finger Protein 280C | 2.390803732 | 227 | 277 | 285 | 284 | 165 | 176 | 163 | 79 | 87 | 86 | 104 | 109 | 101 |
| 12796699 | ZNF502 | zinc finger protein 502 (ZNF502), mRNA. | 2.882490464 | 96 | 138 | 120 | 152 | 70 | 80 | 87 | 28 | 43 | 27 | 43 | 45 | 34 |
| 12752064 | ZNF536 | Zinc Finger Protein 536 | 9.460590092 | 365 | 356 | 471 | 403 | 160 | 126 | 145 | 28 | 29 | 55 | 25 | 19 | 29 |
| 12810843 | ZNF75A | zinc finger protein 75a (ZNF75A), mRNA. | 2.022355024 | 146 | 194 | 117 | 161 | 112 | 84 | 102 | 63 | 60 | 70 | 79 | 67 | 50 |
| 12743827 | ZNF827 | Zinc Finger Protein 827 | 2.369588249 | 297 | 309 | 398 | 275 | 206 | 197 | 184 | 127 | 122 | 119 | 89 | 115 | 104 |
| 12837426 | ZRANB2 | zinc finger, RAN-binding domain containing 2 (ZRANB2), mRNA. | 2.121319901 | 502 | 605 | 469 | 599 | 305 | 374 | 358 | 202 | 194 | 245 | 207 | 198 | 252 |
| 12897900 | ZUFSP | zinc finger with UFM1-specific peptidase domain (ZUFSP), mRNA. | 2.212330816 | 88 | 101 | 104 | 114 | 66 | 64 | 76 | 32 | 30 | 39 | 49 | 44 | 43 |
