# Supplementary material for: Transcriptomes of bovine ovarian follicular and luteal cells
Source: Data Brief. 2016 Dec 10;10:335–9. doi: 10.1016/j.dib.2016.11.093 (PMC5157705; doi:10.1016/j.dib.2016.11.093)
Supplement: Supplementary file 7 — Supplementary material [file mmc7.docx]

| **Table 6. Genes Enriched (≥ 2-fold greater) in Luteal Cells Compared to Follicular Cells** | | |  | **Linear Microarray Results (arbitrary units)** | | | | | | | | | | | | |
| --- | --- | --- | --- | --- | --- | --- | --- | --- | --- | --- | --- | --- | --- | --- | --- | --- |
| **Probe Set ID** | **Gene Symbol** | **Description** | **Fold Change Luteal vs Follicle** | **GC1** | **GC2** | **GC3** | **GC4** | **TC1** | **TC2** | **TC3** | **LLC1** | **LLC2** | **LLC3** | **SLC1** | **SLC2** | **SLC3** |
| 12859375 | A2M | alpha-2-macroglobulin (A2M), mRNA. | 8.077443572 | 41 | 41 | 57 | 37 | 564 | 1024 | 786 | 2328 | 2160 | 1681 | 3768 | 3704 | 4020 |
| 12745778 | AACS | acetoacetyl-CoA synthetase (AACS), mRNA. | 2.989674157 | 172 | 168 | 151 | 129 | 145 | 137 | 135 | 387 | 332 | 394 | 632 | 308 | 604 |
| 12894704 | ABCA1 | ATP-binding cassette, sub-family A (ABC1), member 1 (ABCA1), mRNA. | 6.27403182 | 38 | 35 | 52 | 24 | 130 | 138 | 92 | 504 | 644 | 463 | 332 | 417 | 375 |
| 12798391 | ABHD6 | abhydrolase domain containing 6 (ABHD6), mRNA. | 8.284170938 | 126 | 154 | 238 | 134 | 217 | 225 | 454 | 2503 | 2550 | 2649 | 938 | 615 | 1737 |
| 12881541 | ABLIM3 | actin binding LIM protein family, member 3 (ABLIM3), mRNA. | 2.831816448 | 23 | 19 | 21 | 16 | 42 | 59 | 43 | 117 | 113 | 90 | 91 | 69 | 60 |
| 12897137 | ACAT2 | Acetyl-CoA Acetyltransferase 2 | 2.855285495 | 837 | 701 | 395 | 445 | 972 | 964 | 1009 | 1724 | 1660 | 2026 | 2649 | 2270 | 2697 |
| 12769528 | ACLY | ATP Citrate Lyase | 2.96432653 | 1126 | 1111 | 883 | 599 | 1041 | 1204 | 1436 | 2572 | 3003 | 3461 | 3326 | 2393 | 4047 |
| 12773229 | ACSF2 | acyl-CoA synthetase family member 2 (ACSF2), mRNA. | 2.027322187 | 231 | 200 | 217 | 250 | 362 | 342 | 326 | 557 | 604 | 648 | 595 | 461 | 485 |
| 12773837 | ACSL3 | acyl-CoA synthetase long-chain family member 3 (ACSL3), mRNA. | 2.09837271 | 388 | 385 | 369 | 332 | 351 | 293 | 372 | 674 | 619 | 714 | 865 | 745 | 862 |
| 12909544 | ACSL4 | acyl-CoA synthetase long-chain family member 4, mRNA (cDNA clone IMAGE:8445367), partial cds. | 4.461220018 | 315 | 355 | 244 | 261 | 242 | 218 | 288 | 1485 | 1233 | 2284 | 676 | 670 | 1000 |
| 12811550 | ACSM1 | acyl-CoA synthetase medium-chain family member 1 (ACSM1), mRNA. | 10.18122197 | 52 | 55 | 94 | 50 | 116 | 74 | 78 | 787 | 502 | 1329 | 598 | 828 | 475 |
| 12714225 | ACSS2 | acyl-CoA synthetase short-chain family member 2 (ACSS2), mRNA. | 7.755619995 | 464 | 425 | 249 | 254 | 495 | 392 | 363 | 2451 | 3224 | 3469 | 3021 | 2021 | 3380 |
| 12836080 | ADAMTS4 | ADAM metallopeptidase with thrombospondin type 1 motif, 4 (ADAMTS4), mRNA. | 8.968141207 | 87 | 69 | 70 | 35 | 70 | 62 | 99 | 850 | 782 | 995 | 345 | 389 | 419 |
| 12771716 | ADAP2 | ArfGAP with dual PH domains 2 (ADAP2), mRNA. | 3.973030357 | 17 | 16 | 22 | 12 | 87 | 94 | 69 | 216 | 193 | 133 | 169 | 178 | 191 |
| 12701669 | ADCY3 | adenylate cyclase 3 (ADCY3), mRNA. | 4.141905258 | 63 | 69 | 69 | 63 | 252 | 238 | 250 | 487 | 497 | 325 | 946 | 693 | 616 |
| 12873696 | ADD1 | adducin 1 (alpha) (ADD1), mRNA. | 2.399176449 | 436 | 396 | 450 | 395 | 544 | 557 | 440 | 1227 | 971 | 1039 | 1210 | 1158 | 1011 |
| 12870772 | ADH6 | alcohol dehydrogenase 6 (class V) (ADH6), mRNA. | 15.59314189 | 30 | 43 | 26 | 63 | 49 | 36 | 14 | 611 | 468 | 850 | 407 | 384 | 773 |
| 12876951 | ADRB2 | adrenergic, beta-2-, receptor, surface (ADRB2), mRNA. | 7.298060685 | 15 | 19 | 26 | 20 | 29 | 48 | 31 | 247 | 358 | 295 | 75 | 107 | 94 |
| 12816222 | AGFG2 | ArfGAP with FG repeats 2 (AGFG2), mRNA. | 3.627915511 | 313 | 254 | 257 | 127 | 325 | 325 | 294 | 823 | 946 | 950 | 1083 | 735 | 1354 |
| 12871228 | AGPAT9 | 1-acylglycerol-3-phosphate O-acyltransferase 9 (AGPAT9), mRNA. | 3.38341618 | 61 | 58 | 55 | 69 | 62 | 72 | 109 | 196 | 241 | 258 | 214 | 161 | 340 |
| 12848811 | AHR | aryl hydrocarbon receptor (AHR), mRNA. | 6.007113259 | 47 | 70 | 69 | 64 | 148 | 221 | 228 | 659 | 802 | 677 | 665 | 779 | 782 |
| 12702928 | AIF1L | allograft inflammatory factor 1-like (AIF1L), mRNA. | 13.6594165 | 85 | 99 | 101 | 61 | 88 | 90 | 115 | 2022 | 1522 | 2312 | 772 | 413 | 436 |
| 12703774 | AK1 | adenylate kinase 1 (AK1), mRNA. | 3.031357575 | 38 | 48 | 42 | 36 | 80 | 68 | 67 | 158 | 222 | 161 | 175 | 95 | 176 |
| 12897211 | AKAP12 | PREDICTED: A kinase (PRKA) anchor protein 12 (AKAP12), partial mRNA. | 6.228172763 | 32 | 21 | 16 | 22 | 106 | 137 | 77 | 352 | 397 | 484 | 267 | 263 | 432 |
| 12789222 | AKAP13 | PREDICTED: A kinase (PRKA) anchor protein 13 (AKAP13), mRNA. | 2.507186729 | 98 | 98 | 119 | 89 | 162 | 186 | 166 | 291 | 387 | 262 | 290 | 413 | 330 |
| 12907161 | AKAP4 | A kinase (PRKA) anchor protein 4 (AKAP4), mRNA. | 2.598773598 | 134 | 136 | 91 | 134 | 88 | 70 | 87 | 319 | 223 | 288 | 329 | 133 | 356 |
| 12746119 | ALDH2 | aldehyde dehydrogenase 2 family (mitochondrial) (ALDH2), nuclear gene encoding mitochondrial protein, mRNA. | 3.466179476 | 283 | 264 | 286 | 241 | 504 | 423 | 323 | 1223 | 1120 | 1375 | 1204 | 940 | 1047 |
| 12840394 | ALDH9A1 | aldehyde dehydrogenase 9 family, member A1 (ALDH9A1), mRNA. | 2.135622351 | 269 | 236 | 266 | 247 | 388 | 224 | 220 | 634 | 565 | 521 | 576 | 559 | 529 |
| 12768020 | ALDOC | aldolase C, fructose-bisphosphate (ALDOC), mRNA. | 2.621314595 | 303 | 317 | 337 | 231 | 421 | 254 | 248 | 684 | 846 | 972 | 783 | 562 | 898 |
| 12790124 | ALPK3 | Alpha-Kinase 3 | 2.642588943 | 116 | 111 | 146 | 101 | 107 | 110 | 107 | 187 | 266 | 219 | 324 | 424 | 388 |
| 12794199 | AMIGO3 | PREDICTED: adhesion molecule with Ig-like domain 3 (AMIGO3), mRNA. | 2.841537321 | 148 | 156 | 135 | 99 | 124 | 98 | 118 | 293 | 411 | 378 | 226 | 390 | 441 |
| 12697507 | ANG | angiogenin, ribonuclease, RNase A family, 5 (ANG), mRNA. | 39.39376832 | 8 | 7 | 18 | 13 | 17 | 22 | 47 | 1259 | 839 | 2002 | 230 | 58 | 95 |
| 12697629 | ANG2 | angiogenin 2 (ANG2), mRNA. | 15.01087173 | 12 | 16 | 17 | 13 | 15 | 25 | 29 | 591 | 257 | 619 | 80 | 36 | 60 |
| 12799853 | ANO10 | Anoctamin 10 | 2.526043713 | 174 | 188 | 226 | 217 | 367 | 453 | 381 | 636 | 571 | 536 | 913 | 816 | 873 |
| 12866524 | ANO6 | PREDICTED: anoctamin 6 (ANO6), mRNA. | 2.452431203 | 79 | 82 | 80 | 84 | 182 | 310 | 211 | 355 | 412 | 342 | 353 | 341 | 358 |
| 12871193 | ANXA3 | annexin A3 (ANXA3), mRNA. | 5.727646203 | 12 | 20 | 29 | 17 | 97 | 137 | 116 | 391 | 446 | 337 | 249 | 360 | 318 |
| 12874187 | APBB2 | amyloid beta (A4) precursor protein-binding, family B, member 2 (APBB2), mRNA. | 3.432206098 | 191 | 219 | 230 | 156 | 425 | 643 | 534 | 1477 | 1252 | 1339 | 1020 | 939 | 1024 |
| 12807757 | APCDD1 | adenomatosis polyposis coli down-regulated 1 (APCDD1), NotI truncated, mRNA, incomplete 3' cds. | 2.542218699 | 56 | 60 | 52 | 52 | 54 | 67 | 62 | 212 | 120 | 120 | 183 | 143 | 100 |
| 12832151 | APLP2 | PREDICTED: amyloid beta (A4) precursor-like protein 2 (APLP2), mRNA. | 2.2843822 | 558 | 616 | 678 | 514 | 837 | 918 | 926 | 1500 | 1821 | 1410 | 1734 | 1736 | 1679 |
| 12682282 | APOD | Apolipoprotein D | 5.423356735 | 18 | 20 | 21 | 19 | 694 | 1412 | 1213 | 2708 | 2319 | 2636 | 2694 | 2720 | 2717 |
| 12867769 | APOLD1 | apolipoprotein L domain containing 1 (APOLD1), mRNA. | 5.653190556 | 46 | 31 | 45 | 34 | 51 | 91 | 83 | 400 | 445 | 567 | 135 | 156 | 142 |
| 12853145 | AQP1 | aquaporin 1 (Colton blood group) (AQP1), mRNA. | 8.457973981 | 31 | 27 | 35 | 32 | 83 | 76 | 83 | 519 | 872 | 720 | 139 | 273 | 128 |
| 12872153 | ARHGAP24 | Rho GTPase activating protein 24 (ARHGAP24), mRNA. | 6.009628167 | 24 | 23 | 29 | 25 | 46 | 46 | 42 | 156 | 202 | 171 | 125 | 294 | 264 |
| 12876682 | ARHGAP26 | Rho GTPase activating protein 26 (ARHGAP26), mRNA. | 3.666288616 | 33 | 43 | 31 | 25 | 56 | 67 | 54 | 167 | 173 | 198 | 119 | 132 | 187 |
| 12823316 | ARHGEF10 | Rho Guanine Nucleotide Exchange Factor (GEF) 10 | 2.524319591 | 81 | 83 | 97 | 73 | 122 | 111 | 96 | 338 | 260 | 333 | 154 | 136 | 212 |
| 12700817 | ARID5A | AT rich interactive domain 5A (MRF1-like) (ARID5A), mRNA. | 3.765608648 | 48 | 45 | 43 | 44 | 45 | 55 | 51 | 163 | 193 | 211 | 139 | 160 | 199 |
| 12827244 | ARID5B | PREDICTED: AT rich interactive domain 5B (MRF1-like) (ARID5B), mRNA. | 5.319482577 | 128 | 130 | 98 | 141 | 357 | 377 | 253 | 902 | 1009 | 897 | 1126 | 1450 | 1389 |
| 12848299 | ARL4C | ADP-ribosylation factor-like 4C (ARL4C), mRNA. | 2.914920748 | 38 | 46 | 44 | 42 | 48 | 42 | 35 | 154 | 82 | 95 | 140 | 141 | 125 |
| 12885849 | ARRDC2 | arrestin domain containing 2 (ARRDC2), mRNA. | 3.095098301 | 30 | 26 | 27 | 30 | 34 | 43 | 45 | 130 | 125 | 93 | 98 | 99 | 80 |
| 12887514 | ARRDC3 | arrestin domain containing 3 (ARRDC3), mRNA. | 5.624862214 | 223 | 271 | 365 | 602 | 328 | 290 | 390 | 1642 | 1407 | 1725 | 1997 | 2723 | 2407 |
| 12823782 | ASAH1 | N-acylsphingosine amidohydrolase (acid ceramidase) 1 (ASAH1), mRNA. | 4.282624239 | 79 | 108 | 95 | 124 | 138 | 135 | 146 | 414 | 416 | 458 | 640 | 600 | 500 |
| 12745734 | ASCC2 | Activating Signal Cointegrator 1 Complex Subunit 2 | 2.136404633 | 129 | 126 | 118 | 107 | 142 | 125 | 115 | 262 | 257 | 234 | 327 | 220 | 277 |
| 12744314 | ASPHD2 | PREDICTED: aspartate beta-hydroxylase domain containing 2 (ASPHD2), mRNA. | 3.374911473 | 65 | 57 | 62 | 43 | 83 | 77 | 62 | 224 | 197 | 164 | 282 | 241 | 190 |
| 12739608 | ATF3 | activating transcription factor 3 (ATF3), mRNA. | 19.04662806 | 25 | 26 | 24 | 22 | 36 | 36 | 40 | 541 | 696 | 649 | 459 | 562 | 493 |
| 12810552 | ATP8B1 | ATPase, aminophospholipid transporter, class I, type 8B, member 1 (ATP8B1), mRNA. | 10.58392482 | 16 | 14 | 15 | 20 | 32 | 43 | 33 | 345 | 370 | 396 | 137 | 132 | 190 |
| 12823046 | AVPI1 | arginine vasopressin-induced 1 (AVPI1), mRNA. | 2.805202433 | 85 | 80 | 100 | 80 | 108 | 85 | 86 | 309 | 241 | 346 | 220 | 224 | 161 |
| 12892724 | B4GALT1 | UDP-Gal:betaGlcNAc beta 1,4- galactosyltransferase, polypeptide 1 (B4GALT1), mRNA. | 2.574221422 | 310 | 317 | 280 | 420 | 378 | 402 | 603 | 957 | 1070 | 870 | 1132 | 870 | 1078 |
| 12718108 | B4GALT5 | B.taurus partial mRNA homologous to N-acetyllactosamine synthase. | 2.033076206 | 297 | 337 | 354 | 453 | 212 | 206 | 211 | 487 | 485 | 578 | 667 | 569 | 825 |
| 12685540 | BACH1 | PREDICTED: BTB and CNC homology 1, basic leucine zipper transcription factor 1 (BACH1), mRNA. | 2.692573011 | 65 | 62 | 55 | 51 | 74 | 63 | 71 | 108 | 148 | 129 | 177 | 251 | 205 |
| 12821078 | BAG3 | BCL2-associated athanogene 3 (BAG3), mRNA. | 8.339760523 | 187 | 170 | 179 | 91 | 267 | 160 | 156 | 1723 | 1759 | 1604 | 1058 | 1223 | 1281 |
| 12792851 | BAG5 | BCL2-associated athanogene 5 (BAG5), mRNA. | 2.389641161 | 71 | 72 | 72 | 69 | 86 | 81 | 87 | 195 | 271 | 208 | 154 | 153 | 122 |
| 12768609 | BAIAP2 | BAI1-associated protein 2 (BAIAP2), mRNA. | 2.907311528 | 104 | 87 | 237 | 63 | 95 | 68 | 66 | 302 | 290 | 420 | 255 | 266 | 266 |
| 12720208 | BAMBI | BMP and activin membrane-bound inhibitor homolog (Xenopus laevis) (BAMBI), mRNA. | 4.26938874 | 34 | 26 | 27 | 32 | 41 | 58 | 56 | 189 | 186 | 167 | 171 | 146 | 141 |
| 12787023 | BASP1 | brain abundant, membrane attached signal protein 1 (BASP1), mRNA. | 4.836993926 | 31 | 53 | 53 | 228 | 362 | 395 | 302 | 677 | 898 | 891 | 1103 | 1075 | 1253 |
| 12727180 | BBOX1 | butyrobetaine (gamma), 2-oxoglutarate dioxygenase (gamma-butyrobetaine hydroxylase) 1 (BBOX1), mRNA. | 13.81369498 | 12 | 14 | 8 | 14 | 21 | 21 | 21 | 311 | 234 | 410 | 123 | 99 | 126 |
| 12749545 | BCAM | basal cell adhesion molecule (Lutheran blood group) (BCAM), mRNA. | 6.495965269 | 103 | 100 | 141 | 89 | 257 | 353 | 265 | 1384 | 1422 | 1212 | 1104 | 1186 | 975 |
| 12681523 | BCHE | butyrylcholinesterase | 7.255613167 | 12 | 12 | 11 | 11 | 14 | 28 | 18 | 174 | 77 | 260 | 72 | 34 | 43 |
| 12704547 | BCL2L11 | BCL2-Like 11 (Apoptosis Facilitator) | 2.827277529 | 45 | 31 | 33 | 28 | 35 | 28 | 27 | 89 | 88 | 68 | 122 | 109 | 74 |
| 12752709 | BCL3 | B-cell CLL/lymphoma 3 (BCL3), mRNA. | 4.57380646 | 62 | 45 | 67 | 52 | 83 | 81 | 80 | 301 | 393 | 343 | 269 | 381 | 164 |
| 12681831 | BCL6 | B-cell CLL/lymphoma 6 | 3.746254795 | 137 | 146 | 129 | 131 | 231 | 145 | 90 | 340 | 495 | 395 | 499 | 699 | 810 |
| 12760266 | BCL6B | B-cell CLL/lymphoma 6, member B (BCL6B), mRNA. | 2.943618349 | 41 | 36 | 39 | 33 | 51 | 76 | 69 | 197 | 181 | 195 | 91 | 132 | 74 |
| 12798575 | BHLHE40 | basic helix-loop-helix family, member e40 (BHLHE40), mRNA. | 9.790105043 | 89 | 142 | 287 | 148 | 151 | 113 | 112 | 1966 | 1695 | 2384 | 927 | 882 | 890 |
| 12862249 | BHLHE41 | basic helix-loop-helix family, member e41 (BHLHE41), mRNA. | 3.927271831 | 42 | 39 | 66 | 71 | 70 | 66 | 56 | 187 | 153 | 156 | 304 | 326 | 254 |
| 12732071 | BIRC3 | baculoviral IAP repeat containing 3 (BIRC3), mRNA. | 5.748232703 | 215 | 220 | 203 | 275 | 163 | 166 | 222 | 1012 | 1113 | 1331 | 1116 | 1118 | 1524 |
| 12872081 | BMP3 | bone morphogenetic protein 3 (BMP3), mRNA. | 2.98411574 | 49 | 55 | 56 | 51 | 59 | 58 | 55 | 101 | 85 | 89 | 250 | 167 | 290 |
| 12866290 | BPIFC | BPI fold containing family C (BPIFC), mRNA. | 41.43878837 | 12 | 14 | 12 | 11 | 12 | 14 | 13 | 911 | 739 | 832 | 182 | 101 | 296 |
| 12707882 | BRE | brain and reproductive organ-expressed (TNFRSF1A modulator) (BRE), mRNA. | 2.3330515 | 428 | 449 | 540 | 351 | 486 | 613 | 517 | 1193 | 1166 | 1337 | 956 | 942 | 1174 |
| 12737028 | BTG2 | PREDICTED: BTG family, member 2 (BTG2), mRNA. | 11.58966948 | 203 | 231 | 201 | 188 | 307 | 269 | 394 | 2817 | 2629 | 2610 | 3161 | 3488 | 3106 |
| 12827009 | C10orf10 | chromosome 28 open reading frame, human C10orf10 (C28H10orf10), mRNA. | 2.91232315 | 42 | 40 | 37 | 38 | 56 | 68 | 63 | 180 | 157 | 160 | 117 | 154 | 88 |
| 12737604 | C16H1orf115 | chromosome 16 open reading frame, human C1orf115 (C16H1orf115), mRNA. | 2.347954693 | 69 | 64 | 71 | 73 | 183 | 135 | 121 | 280 | 223 | 252 | 232 | 258 | 194 |
| 12684168 | C1H3orf58 | chromosome 1 open reading frame, human C3orf58 (C1H3orf58), mRNA. | 2.916612648 | 190 | 186 | 182 | 171 | 127 | 120 | 129 | 509 | 494 | 513 | 412 | 402 | 435 |
| 12859128 | C1R | complement component 1, r subcomponent (C1R), mRNA. | 8.902610346 | 40 | 18 | 34 | 29 | 188 | 155 | 164 | 490 | 501 | 526 | 1090 | 947 | 1235 |
| 12858465 | C1RL | complement component 1, r subcomponent-like (C1RL), mRNA. | 3.034295874 | 46 | 39 | 54 | 38 | 72 | 68 | 70 | 149 | 171 | 116 | 206 | 140 | 225 |
| 12868860 | C1S | complement component 1, s subcomponent (C1S), mRNA. | 18.42322449 | 28 | 21 | 20 | 18 | 181 | 117 | 150 | 914 | 781 | 725 | 1938 | 2145 | 1952 |
| 12690238 | C2CD4B | C2 calcium-dependent domain containing 4B (C2CD4B), mRNA. | 6.396835787 | 14 | 12 | 15 | 16 | 12 | 15 | 14 | 79 | 119 | 141 | 68 | 42 | 92 |
| 12865677 | C5H12orf44 | chromosome 5 open reading frame, human C12orf44 (C5H12orf44), mRNA. | 2.189654646 | 119 | 96 | 118 | 119 | 158 | 148 | 127 | 270 | 270 | 236 | 306 | 277 | 305 |
| 12891206 | C8H9orf91 | chromosome 8 open reading frame, human C9orf91 (C8H9orf91), mRNA. | 2.406337973 | 164 | 156 | 173 | 138 | 343 | 298 | 339 | 493 | 421 | 487 | 758 | 493 | 669 |
| 12725230 | CA13 | carbonic anhydrase XIII, mRNA (cDNA clone MGC:179400 IMAGE:8560871), complete cds. | 47.79590884 | 16 | 14 | 13 | 13 | 16 | 12 | 14 | 893 | 601 | 1161 | 427 | 357 | 536 |
| 12724157 | CA2 | carbonic anhydrase II (CA2), mRNA. | 9.373870313 | 29 | 23 | 40 | 24 | 95 | 63 | 90 | 593 | 1011 | 627 | 228 | 195 | 280 |
| 12766949 | CA4 | carbonic anhydrase IV (CA4), mRNA. | 3.308603636 | 43 | 38 | 36 | 34 | 50 | 72 | 56 | 228 | 234 | 143 | 96 | 105 | 129 |
| 12713901 | CACNB2 | Calcium Channel, Voltage-Dependent, Beta 2 Subunit | 3.838159316 | 30 | 38 | 28 | 26 | 103 | 135 | 109 | 172 | 298 | 209 | 238 | 259 | 369 |
| 12861583 | CALCOCO1 | calcium binding and coiled-coil domain 1 (CALCOCO1), mRNA. | 2.771086003 | 122 | 127 | 115 | 113 | 125 | 144 | 102 | 334 | 303 | 297 | 408 | 365 | 312 |
| 12703271 | CAMKMT | calmodulin-lysine N-methyltransferase (CAMKMT), mRNA. | 2.508140645 | 58 | 65 | 61 | 70 | 58 | 59 | 49 | 124 | 128 | 146 | 194 | 135 | 178 |
| 12820137 | CASP7 | PREDICTED: caspase 7, apoptosis-related cysteine peptidase (CASP7), mRNA. | 2.202462541 | 55 | 53 | 57 | 55 | 82 | 99 | 88 | 122 | 169 | 196 | 109 | 161 | 165 |
| 12775648 | CASP8 | caspase 8, apoptosis-related cysteine peptidase (CASP8), mRNA. | 3.57390814 | 39 | 56 | 55 | 35 | 120 | 165 | 218 | 338 | 424 | 386 | 341 | 279 | 340 |
| 12719139 | CASS4 | Cas Scaffolding Protein Family Member 4 | 5.299085431 | 44 | 37 | 43 | 39 | 42 | 46 | 48 | 191 | 116 | 80 | 294 | 433 | 252 |
| 12876822 | CAST | calpastatin (CAST), transcript variant 1, mRNA. | 3.712581072 | 99 | 89 | 122 | 100 | 306 | 495 | 345 | 765 | 827 | 743 | 998 | 777 | 842 |
| 12810233 | CCBE1 | Collagen And Calcium Binding EGF Domains 1 | 12.65217829 | 54 | 23 | 43 | 29 | 80 | 78 | 50 | 294 | 339 | 170 | 802 | 1558 | 712 |
| 12899105 | CCDC170 | Coiled-Coil Domain Containing 170 | 3.198686494 | 40 | 22 | 37 | 32 | 94 | 95 | 70 | 147 | 122 | 128 | 209 | 237 | 228 |
| 12767023 | CCL2 | chemokine (C-C motif) ligand 2 (CCL2), mRNA. | 5.960383455 | 24 | 39 | 30 | 25 | 37 | 109 | 147 | 369 | 487 | 358 | 229 | 346 | 307 |
| 12893390 | CCL21 | chemokine (C-C motif) ligand 21 (CCL21), mRNA. | 17.04369931 | 44 | 42 | 37 | 38 | 62 | 158 | 54 | 1179 | 1752 | 837 | 687 | 1416 | 486 |
| 12806571 | CCND3 | cyclin D3 (CCND3), mRNA. | 2.63674209 | 260 | 220 | 245 | 197 | 545 | 443 | 538 | 762 | 918 | 572 | 1325 | 1062 | 894 |
| 12680419 | CCNL1 | cyclin L1 | 3.303003372 | 262 | 277 | 288 | 335 | 190 | 206 | 280 | 611 | 854 | 855 | 586 | 1072 | 1229 |
| 12691088 | CCPG1 | cell cycle progression 1, mRNA (cDNA clone IMAGE:8060449), partial cds. | 2.005337583 | 596 | 650 | 599 | 693 | 420 | 413 | 469 | 1058 | 1011 | 1153 | 1047 | 1200 | 1129 |
| 12747887 | CCRN4L | Nocturnin (NOCT; CCRN4L) | 3.806169692 | 132 | 105 | 118 | 95 | 66 | 83 | 69 | 451 | 403 | 458 | 280 | 285 | 305 |
| 12894605 | CD274 | CD274 molecule (CD274), mRNA. | 15.63469756 | 19 | 24 | 24 | 28 | 114 | 62 | 143 | 327 | 486 | 511 | 878 | 1562 | 1793 |
| 12773826 | CD302 | CD302 molecule (CD302), mRNA. | 3.756361831 | 37 | 47 | 74 | 37 | 210 | 305 | 201 | 465 | 378 | 346 | 523 | 577 | 643 |
| 12852910 | CD36 | CD36 molecule (thrombospondin receptor) (CD36), mRNA. | 2.898785242 | 103 | 119 | 130 | 143 | 118 | 183 | 152 | 286 | 236 | 211 | 443 | 755 | 421 |
| 12713828 | CD40 | CD40 Molecule, TNF Receptor Superfamily Member 5 | 4.46074588 | 27 | 31 | 29 | 28 | 43 | 42 | 61 | 184 | 211 | 168 | 99 | 171 | 166 |
| 12739756 | CD55 | CD55 Molecule, Decay Accelerating Factor For Complement (Cromer Blood Group) | 6.47183419 | 26 | 36 | 45 | 21 | 162 | 208 | 165 | 540 | 548 | 604 | 684 | 639 | 663 |
| 12731267 | CD59 | CD59 molecule, complement regulatory protein (CD59), mRNA. | 4.140199525 | 125 | 138 | 143 | 104 | 119 | 113 | 108 | 609 | 501 | 562 | 455 | 412 | 474 |
| 12727146 | CD82 | CD82 molecule (CD82), mRNA. | 3.721029571 | 170 | 166 | 191 | 202 | 1777 | 1040 | 1270 | 2699 | 2484 | 2555 | 2975 | 2353 | 2294 |
| 12806784 | CD83 | CD83 Molecule | 9.120671451 | 23 | 16 | 22 | 19 | 28 | 29 | 29 | 227 | 199 | 211 | 231 | 222 | 213 |
| 12863892 | CD9 | CD9 molecule (CD9), mRNA. | 3.603080435 | 371 | 414 | 474 | 202 | 381 | 615 | 569 | 2139 | 1817 | 2036 | 1156 | 1066 | 1129 |
| 12749523 | CDH1 | Cadherin 1, Type 1 | 3.064667334 | 30 | 31 | 45 | 24 | 25 | 24 | 24 | 134 | 86 | 142 | 37 | 57 | 75 |
| 12722069 | CDH17 | cadherin 17, LI cadherin (liver-intestine) (CDH17), mRNA. | 2.933699743 | 39 | 26 | 32 | 38 | 33 | 27 | 29 | 79 | 96 | 67 | 89 | 131 | 102 |
| 12801900 | CDKN1A | cyclin-dependent kinase inhibitor 1A (p21, Cip1) (CDKN1A), mRNA. | 10.72988101 | 84 | 81 | 91 | 46 | 75 | 64 | 74 | 779 | 857 | 921 | 762 | 749 | 663 |
| 12758721 | CDYL2 | chromodomain protein, Y-like 2 (CDYL2), mRNA. | 2.076691482 | 64 | 45 | 61 | 58 | 101 | 78 | 65 | 137 | 139 | 119 | 143 | 149 | 154 |
| 12749645 | CEACAM1 | carcinoembryonic antigen-related cell adhesion molecule 1 (biliary glycoprotein) (CEACAM1), mRNA. | 3.512099768 | 38 | 36 | 38 | 33 | 102 | 81 | 79 | 178 | 213 | 144 | 230 | 235 | 224 |
| 12721602 | CEBPD | CCAAT/enhancer binding protein (C/EBP), delta (CEBPD), mRNA. | 3.957070418 | 262 | 254 | 441 | 344 | 685 | 555 | 737 | 1451 | 1449 | 1503 | 2050 | 2396 | 2270 |
| 12784416 | CERS6 | ceramide synthase 6 (CERS6), mRNA. | 11.0938961 | 34 | 39 | 41 | 27 | 62 | 70 | 63 | 441 | 507 | 381 | 787 | 412 | 675 |
| 12775239 | CFLAR | CASP8 and FADD-like apoptosis regulator (CFLAR), mRNA. | 3.003537711 | 87 | 102 | 65 | 88 | 178 | 182 | 197 | 313 | 316 | 306 | 415 | 520 | 441 |
| 12738652 | CHI3L1 | chitinase 3-like 1 (cartilage glycoprotein-39) (CHI3L1), mRNA. | 4.928381953 | 33 | 31 | 34 | 26 | 34 | 40 | 38 | 117 | 181 | 116 | 217 | 141 | 226 |
| 12724566 | CHMP4C | charged multivesicular body protein 4C (CHMP4C), mRNA. | 2.439035901 | 34 | 45 | 41 | 35 | 96 | 55 | 55 | 110 | 110 | 113 | 121 | 154 | 149 |
| 12797038 | CISH | cytokine inducible SH2-containing protein (CISH), mRNA. | 3.651658237 | 53 | 53 | 47 | 40 | 74 | 63 | 74 | 160 | 247 | 188 | 180 | 266 | 224 |
| 12836500 | CITED4 | Cbp/p300-interacting transactivator, with Glu/Asp-rich carboxy-terminal domain, 4 (CITED4), mRNA. | 2.733341844 | 106 | 81 | 69 | 82 | 103 | 105 | 93 | 301 | 339 | 240 | 240 | 192 | 184 |
| 12842670 | CLCA3 | chloride channel regulator 3 (CLCA3), mRNA. | 5.334152083 | 14 | 17 | 14 | 14 | 26 | 29 | 56 | 97 | 157 | 115 | 120 | 154 | 128 |
| 12748152 | CLDN5 | claudin 5 (CLDN5), mRNA. | 3.862723442 | 49 | 36 | 41 | 38 | 74 | 75 | 64 | 272 | 269 | 307 | 106 | 199 | 97 |
| 12856372 | CLEC2B | PREDICTED: C-type lectin domain family 2, member B (CLEC2B), mRNA. | 10.46417044 | 8 | 13 | 7 | 8 | 22 | 68 | 41 | 211 | 257 | 375 | 218 | 208 | 218 |
| 12904833 | CLIC2 | chloride intracellular channel 2 (CLIC2), mRNA. | 5.225301892 | 14 | 21 | 16 | 16 | 71 | 68 | 58 | 247 | 283 | 209 | 149 | 168 | 135 |
| 12705776 | CLIP4 | CAP-GLY domain containing linker protein family, member 4 (CLIP4), mRNA. | 3.921316844 | 29 | 22 | 32 | 20 | 36 | 39 | 48 | 148 | 139 | 155 | 114 | 91 | 114 |
| 12709983 | CLN5 | ceroid-lipofuscinosis, neuronal 5 (CLN5), mRNA. | 2.626388616 | 187 | 220 | 202 | 240 | 236 | 202 | 239 | 790 | 526 | 902 | 436 | 357 | 424 |
| 12889007 | CLU | clusterin (CLU), mRNA. | 4.195342754 | 213 | 178 | 162 | 99 | 655 | 784 | 851 | 2126 | 2035 | 2257 | 1283 | 1682 | 1202 |
| 12899815 | CNKSR3 | CNKSR family member 3 (CNKSR3), mRNA. | 2.400924504 | 103 | 99 | 151 | 80 | 89 | 73 | 101 | 303 | 242 | 267 | 197 | 224 | 202 |
| 12760627 | CNP | 2',3'-Cyclic Nucleotide 3' Phosphodiesterase | 3.407447927 | 84 | 82 | 102 | 110 | 238 | 196 | 278 | 466 | 403 | 491 | 526 | 502 | 796 |
| 12896338 | CNR1 | cannabinoid receptor 1 (brain) (CNR1), mRNA. | 10.86150944 | 28 | 26 | 28 | 25 | 49 | 59 | 31 | 177 | 687 | 460 | 283 | 169 | 527 |
| 12895030 | CNTFR | PREDICTED: ciliary neurotrophic factor receptor, transcript variant 1 (CNTFR), mRNA. | 2.246599428 | 75 | 57 | 65 | 54 | 64 | 63 | 67 | 109 | 112 | 122 | 178 | 130 | 206 |
| 12777627 | COBLL1 | Cordon-Bleu WH2 Repeat Protein-Like 1 | 5.725077641 | 52 | 59 | 149 | 41 | 227 | 238 | 312 | 845 | 839 | 904 | 833 | 762 | 1111 |
| 12691915 | CORO2B | Coronin, Actin Binding Protein, 2B | 6.958315278 | 31 | 26 | 31 | 30 | 30 | 43 | 44 | 334 | 266 | 291 | 239 | 138 | 131 |
| 12759360 | COTL1 | Coactosin-Like F-Actin Binding Protein 1 | 2.730731002 | 27 | 46 | 57 | 30 | 112 | 101 | 74 | 195 | 183 | 196 | 170 | 162 | 142 |
| 12872049 | CPEB2 | Cytoplasmic Polyadenylation Element Binding Protein 2 | 2.928330968 | 168 | 187 | 251 | 250 | 440 | 528 | 631 | 748 | 1033 | 907 | 1271 | 1006 | 1195 |
| 12705521 | CRAT | carnitine O-acetyltransferase (CRAT), nuclear gene encoding mitochondrial protein, mRNA. | 2.395002596 | 460 | 487 | 495 | 363 | 1253 | 882 | 1000 | 2036 | 1776 | 1797 | 1880 | 1341 | 1310 |
| 12727699 | CREB3L1 | cAMP responsive element binding protein 3-like 1 (CREB3L1), mRNA. | 3.741774575 | 77 | 45 | 55 | 52 | 139 | 146 | 113 | 314 | 246 | 178 | 502 | 409 | 364 |
| 12867556 | CREBL2 | cAMP responsive element binding protein-like 2 (CREBL2), mRNA. | 2.056429926 | 138 | 138 | 141 | 131 | 162 | 161 | 172 | 336 | 371 | 439 | 245 | 222 | 228 |
| 12839535 | CREG1 | cellular repressor of E1A-stimulated genes 1 (CREG1), mRNA. | 2.430531412 | 186 | 285 | 259 | 441 | 604 | 390 | 452 | 814 | 802 | 787 | 970 | 970 | 1106 |
| 12720762 | CREM | cAMP responsive element modulator (CREM), mRNA. | 3.047608586 | 63 | 55 | 78 | 93 | 29 | 29 | 36 | 179 | 196 | 143 | 178 | 104 | 200 |
| 12852971 | CROT | carnitine O-octanoyltransferase (CROT), mRNA. | 3.343072944 | 94 | 90 | 161 | 135 | 144 | 149 | 188 | 368 | 397 | 354 | 597 | 520 | 514 |
| 12730813 | CRYAB | Crystallin, Alpha B | 14.78943795 | 27 | 19 | 17 | 14 | 96 | 105 | 67 | 586 | 917 | 609 | 689 | 790 | 793 |
| 12842594 | CSF1 | Colony Stimulating Factor 1 (Macrophage) | 4.098809051 | 30 | 30 | 27 | 31 | 75 | 106 | 124 | 182 | 261 | 230 | 222 | 335 | 259 |
| 12860625 | CSF2RB | colony stimulating factor 2 receptor, beta, low-affinity (granulocyte-macrophage) (CSF2RB), mRNA. | 2.993144884 | 36 | 38 | 45 | 36 | 72 | 59 | 57 | 84 | 121 | 100 | 227 | 140 | 207 |
| 12799096 | CSRNP1 | Cysteine-Serine-Rich Nuclear Protein 1 | 5.386516727 | 64 | 63 | 50 | 47 | 71 | 60 | 62 | 323 | 257 | 369 | 295 | 357 | 327 |
| 12867807 | CSRP2 | cysteine and glycine-rich protein 2 (CSRP2), mRNA. | 3.304494115 | 389 | 396 | 240 | 265 | 364 | 302 | 391 | 857 | 848 | 759 | 1096 | 1947 | 1141 |
| 12899445 | CTGF | Connective Tissue Growth Factor | 5.732636893 | 66 | 76 | 87 | 47 | 1607 | 1581 | 1151 | 2558 | 2955 | 2673 | 4313 | 5304 | 4865 |
| 12715356 | CTSA | cathepsin A (CTSA), mRNA. | 2.028401707 | 741 | 803 | 955 | 641 | 1112 | 988 | 988 | 1404 | 1542 | 1416 | 2028 | 2146 | 2291 |
| 12829510 | CTSD | cathepsin D (CTSD), mRNA. | 2.459304012 | 824 | 831 | 1179 | 635 | 1227 | 1142 | 1167 | 2641 | 2296 | 2579 | 2611 | 2355 | 2285 |
| 12833395 | CTSF | cathepsin F (CTSF), mRNA. | 7.500854412 | 197 | 162 | 179 | 135 | 800 | 707 | 563 | 2291 | 2597 | 2215 | 3237 | 3669 | 3629 |
| 12713983 | CTSZ | cathepsin Z (CTSZ), mRNA. | 2.466258368 | 639 | 533 | 544 | 306 | 624 | 268 | 393 | 1619 | 1222 | 1475 | 1074 | 785 | 814 |
| 12751936 | CX3CL1 | Chemokine (C-X3-C Motif) Ligand 1 | 5.340258051 | 11 | 16 | 13 | 12 | 24 | 34 | 31 | 180 | 151 | 158 | 58 | 52 | 45 |
| 12683338 | CXADR | coxsackie virus and adenovirus receptor (CXADR), mRNA. | 3.779460159 | 110 | 98 | 97 | 82 | 216 | 238 | 345 | 475 | 380 | 358 | 1017 | 924 | 693 |
| 12762860 | CXCL16 | chemokine (C-X-C motif) ligand 16 (CXCL16), mRNA. | 5.894315615 | 50 | 53 | 90 | 49 | 319 | 362 | 249 | 805 | 736 | 865 | 934 | 1216 | 1374 |
| 12870009 | CXCL2 | chemokine (C-X-C motif) ligand 2 (CXCL2), mRNA. | 7.714519395 | 49 | 47 | 53 | 34 | 71 | 83 | 75 | 685 | 785 | 588 | 254 | 178 | 240 |
| 12872284 | CXCL3 | chemokine (C-X-C motif) ligand 3 (CXCL3), mRNA. | 20.66000159 | 10 | 7 | 9 | 6 | 13 | 52 | 46 | 601 | 579 | 668 | 201 | 221 | 254 |
| 12869838 | CXCL8 | Chemokine (C-X-C Motif) Ligand 8 (CXCL8; IL8) | 9.798538968 | 22 | 20 | 23 | 26 | 17 | 24 | 23 | 196 | 165 | 390 | 146 | 183 | 236 |
| 12762051 | CYB5D1 | cytochrome b5 domain containing 1 (CYB5D1), mRNA. | 2.138537466 | 56 | 55 | 45 | 30 | 40 | 44 | 34 | 97 | 118 | 120 | 60 | 80 | 83 |
| 12843892 | CYR61 | cysteine-rich, angiogenic inducer, 61 (CYR61), mRNA. | 4.41142458 | 181 | 150 | 230 | 182 | 1090 | 1116 | 1059 | 1981 | 2125 | 1829 | 2690 | 3345 | 3188 |
| 12882137 | CYSTM1 | cysteine-rich transmembrane module containing 1 (CYSTM1), mRNA. | 2.188715102 | 578 | 570 | 568 | 406 | 635 | 618 | 649 | 1316 | 1401 | 1122 | 1336 | 1116 | 1258 |
| 12803894 | DAAM2 | Dishevelled Associated Activator Of Morphogenesis 2 | 5.675631547 | 18 | 23 | 23 | 30 | 107 | 96 | 80 | 339 | 315 | 388 | 182 | 123 | 483 |
| 12879927 | DAPK3 | death-associated protein kinase 3 (DAPK3), mRNA. | 2.583152982 | 131 | 143 | 149 | 134 | 226 | 250 | 186 | 424 | 471 | 448 | 412 | 514 | 426 |
| 12756093 | DBP | D site of albumin promoter (albumin D-box) binding protein (DBP), mRNA. | 2.628303917 | 80 | 76 | 115 | 72 | 123 | 95 | 111 | 173 | 235 | 217 | 192 | 427 | 273 |
| 12685136 | DCBLD2 | discoidin, CUB and LCCL domain containing 2 (DCBLD2), mRNA. | 2.219009897 | 841 | 847 | 840 | 585 | 774 | 686 | 739 | 1533 | 1616 | 1606 | 1905 | 1422 | 2021 |
| 12815458 | DCUN1D3 | DCN1, defective in cullin neddylation 1, domain containing 3 (S. cerevisiae) (DCUN1D3), mRNA. | 2.545274603 | 123 | 168 | 135 | 125 | 116 | 111 | 110 | 185 | 240 | 187 | 386 | 522 | 417 |
| 12862848 | DDIT3 | DNA-damage-inducible transcript 3 (DDIT3), mRNA. | 5.923059438 | 140 | 163 | 170 | 157 | 187 | 207 | 238 | 822 | 928 | 1073 | 959 | 1234 | 1397 |
| 12826569 | DDIT4 | DNA-damage-inducible transcript 4 (DDIT4), mRNA. | 2.772679829 | 172 | 211 | 272 | 221 | 205 | 118 | 204 | 806 | 538 | 766 | 543 | 241 | 443 |
| 12896602 | DDO | D-Aspartate Oxidase | 4.737350383 | 17 | 28 | 37 | 26 | 30 | 38 | 26 | 98 | 119 | 83 | 156 | 193 | 172 |
| 12907898 | DDX3X | DEAD (Asp-Glu-Ala-Asp) box polypeptide 3, X-linked (DDX3X), mRNA. | 2.12299134 | 494 | 610 | 678 | 640 | 599 | 769 | 845 | 1512 | 1340 | 1341 | 1250 | 1569 | 1422 |
| 12814482 | DECR2 | 2,4-dienoyl CoA reductase 2, peroxisomal (DECR2), mRNA. | 2.19430164 | 70 | 70 | 80 | 74 | 96 | 96 | 101 | 176 | 153 | 200 | 161 | 161 | 253 |
| 12724574 | DENND3 | PREDICTED: DENN/MADD domain containing 3 (DENND3), mRNA. | 2.86350015 | 54 | 51 | 63 | 44 | 58 | 60 | 57 | 171 | 186 | 189 | 149 | 136 | 120 |
| 12728877 | DEPDC7 | DEP domain containing 7 (DEPDC7), mRNA. | 4.41575731 | 24 | 25 | 19 | 24 | 24 | 21 | 25 | 59 | 119 | 71 | 122 | 110 | 128 |
| 12833750 | DHCR7 | 7-dehydrocholesterol reductase (DHCR7), mRNA. | 2.387468667 | 1659 | 1411 | 1152 | 711 | 2032 | 1828 | 1759 | 3132 | 3164 | 3856 | 4157 | 3200 | 4086 |
| 12693416 | DHRS4 | Dehydrogenase/Reductase (SDR Family) Member 4 | 4.213295576 | 309 | 267 | 229 | 144 | 333 | 277 | 225 | 944 | 1458 | 1130 | 1081 | 813 | 1021 |
| 12872256 | DKK2 | dickkopf homolog 2 (Xenopus laevis) (DKK2), mRNA. | 24.91270079 | 39 | 38 | 48 | 41 | 80 | 104 | 102 | 1011 | 1157 | 597 | 2040 | 2741 | 2099 |
| 12823815 | DLC1 | deleted in liver cancer 1 (DLC1), mRNA. | 4.717752612 | 43 | 37 | 36 | 31 | 120 | 176 | 144 | 525 | 484 | 452 | 309 | 332 | 269 |
| 12757161 | DMPK | Dystrophia Myotonica-Protein Kinase | 2.066125746 | 74 | 64 | 71 | 57 | 119 | 117 | 123 | 160 | 220 | 154 | 219 | 162 | 192 |
| 12910548 | DNAJA1 | DnaJ (Hsp40) homolog, subfamily A, member 1, mRNA (cDNA clone MGC:159450 IMAGE:8166261), complete cds. | 2.86459491 | 195 | 210 | 122 | 182 | 167 | 160 | 189 | 387 | 650 | 503 | 410 | 489 | 570 |
| 12790479 | DNAJA4 | DnaJ (Hsp40) homolog, subfamily A, member 4 (DNAJA4), mRNA. | 3.257953875 | 158 | 157 | 157 | 144 | 80 | 55 | 66 | 413 | 494 | 408 | 268 | 317 | 382 |
| 12880985 | DNAJB1 | DnaJ (Hsp40) homolog, subfamily B, member 1 (DNAJB1), mRNA. | 11.43448618 | 319 | 255 | 182 | 168 | 285 | 185 | 265 | 3091 | 2821 | 2890 | 2311 | 2695 | 2450 |
| 12777779 | DNAJB2 | DnaJ (Hsp40) homolog, subfamily B, member 2 (DNAJB2), mRNA. | 2.246867217 | 160 | 159 | 180 | 100 | 204 | 191 | 168 | 361 | 391 | 335 | 357 | 379 | 414 |
| 12847198 | DNAJB4 | DnaJ (Hsp40) homolog, subfamily B, member 4 (DNAJB4), mRNA. | 8.171289858 | 83 | 92 | 117 | 122 | 117 | 87 | 118 | 719 | 831 | 900 | 772 | 1034 | 903 |
| 12855730 | DNAJB9 | DnaJ (Hsp40) homolog, subfamily B, member 9 (DNAJB9), mRNA. | 2.176858055 | 101 | 121 | 94 | 101 | 59 | 48 | 74 | 252 | 157 | 229 | 168 | 138 | 172 |
| 12735104 | DNM3 | dynamin 3 (DNM3), mRNA. | 2.34436887 | 56 | 75 | 100 | 84 | 120 | 150 | 121 | 265 | 277 | 234 | 193 | 250 | 199 |
| 12851048 | DOCK4 | Dedicator Of Cytokinesis 4 | 2.552454079 | 34 | 38 | 33 | 33 | 60 | 80 | 64 | 126 | 153 | 122 | 88 | 135 | 122 |
| 12880011 | DOHH | deoxyhypusine hydroxylase/monooxygenase (DOHH), mRNA. | 3.439632529 | 58 | 47 | 62 | 53 | 70 | 47 | 46 | 246 | 186 | 182 | 209 | 149 | 158 |
| 12714968 | DSTN | destrin (actin depolymerizing factor) (DSTN), mRNA. | 2.372186271 | 357 | 411 | 384 | 342 | 769 | 871 | 652 | 1244 | 1159 | 1468 | 1159 | 1344 | 1325 |
| 12742583 | DTX1 | deltex homolog 1 (Drosophila) (DTX1), mRNA. | 2.667549225 | 37 | 36 | 38 | 31 | 46 | 46 | 39 | 120 | 132 | 98 | 102 | 104 | 73 |
| 12788100 | DUSP1 | dual specificity phosphatase 1 (DUSP1), mRNA. | 11.74989655 | 205 | 190 | 182 | 165 | 516 | 480 | 751 | 4021 | 3893 | 3744 | 4053 | 5164 | 4190 |
| 12707400 | DUSP2 | dual specificity phosphatase 2 (DUSP2), mRNA. | 2.839594252 | 36 | 30 | 39 | 35 | 37 | 39 | 53 | 123 | 84 | 125 | 153 | 87 | 85 |
| 12819271 | DUSP5 | Dual Specificity Phosphatase 5 | 23.09793462 | 55 | 55 | 39 | 32 | 61 | 72 | 91 | 1037 | 1005 | 941 | 1500 | 1725 | 1805 |
| 12734981 | DYRK3 | dual-specificity tyrosine-(Y)-phosphorylation regulated kinase 3 (DYRK3), mRNA. | 5.656122654 | 37 | 32 | 36 | 41 | 35 | 38 | 33 | 265 | 235 | 273 | 163 | 170 | 118 |
| 12704678 | DYSF | dysferlin, limb girdle muscular dystrophy 2B (autosomal recessive) (DYSF), mRNA. | 3.929074425 | 73 | 69 | 65 | 80 | 115 | 132 | 104 | 567 | 508 | 414 | 249 | 255 | 150 |
| 12683292 | ECE2 | Endothelin Converting Enzyme 2 | 2.932446156 | 73 | 70 | 67 | 69 | 85 | 74 | 59 | 196 | 176 | 279 | 169 | 189 | 240 |
| 12845421 | ECM1 | extracellular matrix protein 1 (ECM1), mRNA. | 3.969269557 | 31 | 21 | 34 | 25 | 39 | 37 | 42 | 199 | 156 | 155 | 98 | 85 | 79 |
| 12843663 | EFNA1 | Ephrin-A1 | 2.780274645 | 37 | 32 | 36 | 33 | 46 | 58 | 54 | 175 | 144 | 142 | 67 | 105 | 73 |
| 12710948 | EFNB2 | PREDICTED: ephrin-B2 (EFNB2), mRNA. | 6.981585754 | 25 | 26 | 30 | 19 | 40 | 55 | 47 | 304 | 495 | 254 | 145 | 141 | 112 |
| 12877406 | EGR1 | Early Growth Response 1 | 6.304655915 | 889 | 753 | 979 | 369 | 554 | 689 | 667 | 3967 | 4199 | 4041 | 4440 | 5191 | 4636 |
| 12827463 | EGR2 | PREDICTED: early growth response 2, transcript variant 1 (EGR2), mRNA. | 3.250086444 | 31 | 28 | 28 | 24 | 39 | 41 | 40 | 182 | 100 | 78 | 79 | 147 | 51 |
| 12833536 | EHD1 | EH-Domain Containing 1 | 2.614199275 | 81 | 80 | 88 | 103 | 152 | 121 | 121 | 241 | 260 | 237 | 349 | 279 | 307 |
| 12693873 | EHD4 | EH-domain containing 4 (EHD4), mRNA. | 7.175132811 | 52 | 30 | 53 | 38 | 122 | 160 | 183 | 978 | 878 | 880 | 382 | 419 | 393 |
| 12806809 | ELOVL5 | ELOVL fatty acid elongase 5 (ELOVL5), mRNA. | 2.508497722 | 779 | 797 | 668 | 762 | 1293 | 1190 | 1263 | 1748 | 2329 | 2014 | 2557 | 2585 | 3283 |
| 12807909 | EMILIN2 | Elastin Microfibril Interfacer 2 | 5.69083886 | 37 | 34 | 55 | 27 | 52 | 42 | 71 | 244 | 224 | 356 | 264 | 249 | 214 |
| 12869336 | EMP1 | PREDICTED: epithelial membrane protein 1, transcript variant 1 (EMP1), mRNA. | 13.79457601 | 26 | 23 | 43 | 31 | 76 | 148 | 121 | 977 | 1223 | 1522 | 653 | 573 | 577 |
| 12733011 | ENDOD1 | endonuclease domain containing 1 (ENDOD1), mRNA. | 4.921940311 | 71 | 71 | 71 | 64 | 167 | 144 | 142 | 771 | 502 | 605 | 456 | 413 | 339 |
| 12703748 | ENG | Endoglin | 5.444052485 | 66 | 49 | 48 | 54 | 200 | 361 | 314 | 1293 | 1231 | 989 | 533 | 604 | 448 |
| 12810638 | ENOSF1 | enolase superfamily member 1 (ENOSF1), mRNA. | 2.126939873 | 217 | 220 | 259 | 311 | 352 | 274 | 184 | 365 | 489 | 395 | 698 | 642 | 723 |
| 12698660 | EPAS1 | Endothelial PAS Domain Protein 1 | 8.300049933 | 83 | 90 | 88 | 83 | 348 | 681 | 526 | 3415 | 3278 | 3092 | 1347 | 1288 | 1098 |
| 12776360 | EPHA2 | EPH receptor A2 (EPHA2), mRNA. | 2.96116636 | 87 | 98 | 78 | 98 | 72 | 86 | 64 | 316 | 304 | 261 | 186 | 214 | 198 |
| 12890086 | EPHX2 | epoxide hydrolase 2, cytoplasmic (EPHX2), mRNA. | 2.52706126 | 193 | 187 | 213 | 256 | 207 | 218 | 231 | 464 | 554 | 490 | 649 | 544 | 558 |
| 12861534 | EPS8 | epidermal growth factor receptor pathway substrate 8, mRNA (cDNA clone MGC:137738 IMAGE:8164331), complete cds. | 4.705225076 | 23 | 23 | 25 | 19 | 29 | 41 | 31 | 198 | 191 | 216 | 64 | 48 | 51 |
| 12754861 | ERF | Ets2 repressor factor (ERF), mRNA. | 3.243920905 | 109 | 101 | 101 | 80 | 111 | 95 | 78 | 408 | 276 | 347 | 308 | 299 | 237 |
| 12891664 | ERMP1 | PREDICTED: endoplasmic reticulum metallopeptidase 1 (ERMP1), mRNA. | 2.172811666 | 272 | 266 | 319 | 401 | 234 | 239 | 201 | 875 | 562 | 732 | 562 | 413 | 455 |
| 12771139 | ERN1 | PREDICTED: endoplasmic reticulum to nucleus signaling 1 (ERN1), mRNA. | 2.201943023 | 147 | 150 | 170 | 155 | 152 | 129 | 136 | 439 | 342 | 420 | 268 | 257 | 236 |
| 12735710 | ERRFI1 | ERBB receptor feedback inhibitor 1 (ERRFI1), mRNA. | 2.922909873 | 391 | 396 | 380 | 284 | 1021 | 669 | 742 | 1472 | 1209 | 1428 | 1695 | 2144 | 1779 |
| 12786321 | ESM1 | Endothelial Cell-Specific Molecule 1 | 11.96858231 | 48 | 56 | 29 | 46 | 93 | 186 | 207 | 2013 | 1458 | 1635 | 758 | 579 | 377 |
| 12834727 | ETS1 | v-ets erythroblastosis virus E26 oncogene homolog 1 (avian) (ETS1), mRNA. | 6.431461331 | 23 | 14 | 21 | 27 | 109 | 135 | 99 | 616 | 568 | 518 | 203 | 262 | 193 |
| 12678583 | ETS2 | v-ets erythroblastosis virus E26 oncogene homolog 2 (avian) | 9.700593785 | 26 | 16 | 21 | 22 | 42 | 62 | 53 | 409 | 402 | 498 | 194 | 275 | 226 |
| 12855561 | ETV1 | ets variant 1 (ETV1), mRNA. | 3.903718242 | 19 | 26 | 37 | 23 | 26 | 28 | 36 | 122 | 106 | 85 | 154 | 71 | 116 |
| 12840580 | ETV3 | ets variant 3 (ETV3), mRNA. | 2.537694602 | 346 | 337 | 314 | 321 | 308 | 247 | 300 | 864 | 871 | 903 | 719 | 686 | 683 |
| 12902334 | F8 | coagulation factor VIII, procoagulant component (F8), mRNA. | 3.26001333 | 102 | 105 | 115 | 100 | 157 | 182 | 145 | 437 | 505 | 310 | 428 | 482 | 372 |
| 12774529 | FABP3 | Fatty Acid Binding Protein 3, Muscle And Heart | 9.517364457 | 105 | 98 | 96 | 74 | 87 | 48 | 55 | 735 | 884 | 1488 | 460 | 312 | 707 |
| 12721605 | FABP5 | fatty acid binding protein 5 (psoriasis-associated) (FABP5), mRNA. | 8.266016834 | 9 | 12 | 17 | 14 | 21 | 26 | 25 | 179 | 79 | 61 | 224 | 120 | 210 |
| 12763768 | FADS6 | fatty acid desaturase domain family, member 6 (FADS6), mRNA. | 2.984314859 | 80 | 89 | 61 | 52 | 100 | 95 | 107 | 275 | 257 | 285 | 263 | 192 | 218 |
| 12773136 | FAM100B | PREDICTED: family with sequence similarity 100, member B (FAM100B), mRNA. | 2.051536396 | 226 | 196 | 238 | 154 | 185 | 177 | 122 | 312 | 317 | 363 | 440 | 405 | 445 |
| 12723640 | FAM110B | family with sequence similarity 110, member B, mRNA (cDNA clone MGC:128948 IMAGE:8120596), complete cds. | 3.177163288 | 154 | 119 | 212 | 139 | 156 | 115 | 155 | 372 | 436 | 358 | 587 | 571 | 537 |
| 12712983 | FAM155A | family with sequence similarity 155, member A (FAM155A), mRNA. | 5.301219321 | 21 | 20 | 26 | 17 | 18 | 19 | 26 | 67 | 75 | 78 | 110 | 150 | 193 |
| 12910066 | FAM155B | PREDICTED: family with sequence similarity 155, member B (FAM155B), mRNA. | 2.147198473 | 59 | 84 | 60 | 69 | 78 | 64 | 66 | 103 | 179 | 123 | 151 | 162 | 163 |
| 12823271 | FAM196A | Family With Sequence Similarity 196, Member A | 2.499975469 | 29 | 58 | 37 | 39 | 41 | 51 | 47 | 99 | 66 | 71 | 167 | 136 | 106 |
| 12775507 | FAM46B | family with sequence similarity 46, member B, mRNA (cDNA clone MGC:137624 IMAGE:8180983), complete cds. | 3.87004464 | 55 | 42 | 52 | 45 | 74 | 66 | 58 | 189 | 198 | 151 | 276 | 231 | 251 |
| 12698988 | FAM49A | family with sequence similarity 49, member A (FAM49A), mRNA. | 5.164748294 | 28 | 29 | 50 | 18 | 81 | 106 | 95 | 282 | 259 | 218 | 485 | 295 | 262 |
| 12719555 | FAM65C | PREDICTED: family with sequence similarity 65, member C (FAM65C), mRNA. | 8.24278383 | 38 | 25 | 35 | 29 | 50 | 47 | 48 | 179 | 171 | 138 | 405 | 611 | 409 |
| 12699208 | FAM82A1 | family with sequence similarity 82, member A1 (FAM82A1), mRNA. | 2.968283204 | 133 | 132 | 147 | 155 | 305 | 149 | 238 | 488 | 453 | 530 | 584 | 489 | 660 |
| 12760706 | FASN | fatty acid synthase (FASN), mRNA. | 2.829770378 | 626 | 621 | 579 | 396 | 1024 | 769 | 752 | 1608 | 1574 | 1872 | 2264 | 1916 | 2328 |
| 12798904 | FBLN2 | Fibulin 2 | 9.868185626 | 60 | 43 | 57 | 57 | 91 | 92 | 91 | 300 | 454 | 445 | 536 | 1463 | 958 |
| 12793564 | FBXO33 | F-box protein 33 (FBXO33), mRNA. | 2.95802385 | 210 | 192 | 158 | 189 | 223 | 155 | 207 | 538 | 506 | 516 | 594 | 690 | 538 |
| 12858588 | FBXO7 | F-box protein 7 (FBXO7), mRNA. | 2.379644479 | 326 | 300 | 269 | 276 | 259 | 277 | 296 | 636 | 640 | 638 | 786 | 562 | 823 |
| 12726690 | FDX1 | ferredoxin 1 (FDX1), nuclear gene encoding mitochondrial protein, mRNA. | 3.953722083 | 481 | 448 | 389 | 542 | 1609 | 1151 | 1488 | 4157 | 3794 | 4504 | 2881 | 2348 | 3019 |
| 12719918 | FERMT1 | fermitin family member 1 (FERMT1), mRNA. | 2.431810426 | 23 | 24 | 28 | 30 | 41 | 87 | 51 | 87 | 113 | 89 | 80 | 111 | 113 |
| 12864575 | FGD4 | FYVE, RhoGEF And PH Domain Containing 4 | 2.65455618 | 35 | 28 | 35 | 24 | 42 | 58 | 64 | 120 | 131 | 120 | 76 | 118 | 87 |
| 12867839 | FGD6 | FYVE, RhoGEF and PH domain containing 6, mRNA (cDNA clone IMAGE:8084684), partial cds. | 3.129517428 | 19 | 23 | 24 | 27 | 39 | 61 | 49 | 115 | 157 | 123 | 82 | 89 | 87 |
| 12883137 | FGF1 | Fibroblast Growth Factor 1 (Acidic) | 3.744201393 | 17 | 15 | 19 | 14 | 46 | 56 | 43 | 93 | 116 | 82 | 135 | 165 | 82 |
| 12745287 | FGF2 | fibroblast growth factor 2 (basic) (FGF2), mRNA. | 2.142599404 | 72 | 88 | 89 | 81 | 121 | 105 | 115 | 181 | 230 | 248 | 160 | 177 | 239 |
| 12741910 | FGG | fibrinogen gamma chain (FGG), mRNA. | 221.0162179 | 10 | 10 | 8 | 10 | 7 | 10 | 8 | 3275 | 2044 | 3408 | 1154 | 1136 | 1154 |
| 12823715 | FGL1 | fibrinogen-like 1 (FGL1), mRNA. | 4.913287147 | 14 | 15 | 13 | 15 | 23 | 28 | 27 | 100 | 121 | 81 | 106 | 90 | 71 |
| 12726408 | FJX1 | PREDICTED: four jointed box 1 (Drosophila) (FJX1), partial mRNA. | 2.501670922 | 54 | 35 | 43 | 42 | 63 | 65 | 57 | 127 | 150 | 115 | 94 | 150 | 133 |
| 12709110 | FLT1 | fms-related tyrosine kinase 1 (vascular endothelial growth factor/vascular permeability factor receptor) (FLT1), mRNA. | 8.419005089 | 31 | 26 | 30 | 23 | 78 | 192 | 158 | 1030 | 1112 | 772 | 382 | 380 | 212 |
| 12877300 | FLT4 | Fms-Related Tyrosine Kinase 4 | 7.19226099 | 43 | 43 | 38 | 32 | 47 | 64 | 51 | 320 | 550 | 270 | 220 | 411 | 187 |
| 12703109 | FNDC4 | Fibronectin Type III Domain Containing 4 | 7.603550719 | 48 | 46 | 61 | 32 | 110 | 86 | 144 | 503 | 472 | 386 | 584 | 505 | 985 |
| 12687887 | FOS | FBJ murine osteosarcoma viral oncogene homolog (FOS), mRNA. | 13.87901844 | 247 | 216 | 176 | 72 | 164 | 418 | 328 | 2937 | 2932 | 3204 | 3098 | 3577 | 3530 |
| 12753541 | FOSB | FBJ murine osteosarcoma viral oncogene homolog B (FOSB), mRNA. | 16.2644783 | 223 | 185 | 153 | 68 | 171 | 132 | 123 | 2182 | 2444 | 2499 | 2309 | 2845 | 2426 |
| 12834336 | FOSL1 | FOS-like antigen 1 (FOSL1), mRNA. | 4.283167359 | 27 | 28 | 27 | 21 | 41 | 27 | 33 | 167 | 155 | 162 | 73 | 121 | 73 |
| 12705036 | FOSL2 | FOS-like antigen 2 (FOSL2), mRNA. | 9.231428596 | 144 | 130 | 71 | 83 | 213 | 269 | 181 | 1130 | 1376 | 1288 | 1506 | 1674 | 1674 |
| 12830237 | FOXRED1 | FAD-dependent oxidoreductase domain containing 1 (FOXRED1), nuclear gene encoding mitochondrial protein, mRNA. | 2.785588852 | 170 | 176 | 180 | 199 | 223 | 214 | 205 | 613 | 488 | 586 | 552 | 439 | 587 |
| 12796093 | FRMD4B | FERM domain containing 4B (FRMD4B), mRNA. | 5.847954034 | 20 | 20 | 55 | 18 | 104 | 119 | 98 | 356 | 414 | 384 | 297 | 389 | 339 |
| 12690306 | FRMD6 | FERM domain containing 6 (FRMD6), mRNA. | 2.644817345 | 32 | 27 | 38 | 26 | 106 | 177 | 141 | 207 | 233 | 182 | 195 | 200 | 221 |
| 12879171 | FSTL3 | Follistatin-Like 3 (Secreted Glycoprotein) | 5.256851768 | 109 | 91 | 65 | 37 | 143 | 84 | 87 | 352 | 460 | 314 | 690 | 400 | 563 |
| 12900274 | FUCA2 | fucosidase, alpha-L- 2, plasma (FUCA2), mRNA. | 3.875543757 | 56 | 64 | 54 | 66 | 85 | 63 | 72 | 340 | 213 | 455 | 188 | 164 | 169 |
| 12897625 | FYN | FYN oncogene related to SRC, FGR, YES (FYN), mRNA. | 2.041743867 | 55 | 63 | 84 | 97 | 70 | 129 | 108 | 210 | 197 | 152 | 198 | 151 | 153 |
| 12829673 | FZD4 | frizzled family receptor 4 (FZD4), mRNA. | 3.254344432 | 54 | 44 | 58 | 37 | 82 | 113 | 105 | 290 | 355 | 260 | 161 | 164 | 150 |
| 12907094 | G6PD | glucose-6-phosphate dehydrogenase (G6PD), mRNA. | 2.074227095 | 1058 | 986 | 1154 | 657 | 1442 | 1145 | 1076 | 2360 | 1982 | 2088 | 2494 | 2080 | 2360 |
| 12864320 | GABARAPL1 | GABA(A) receptor-associated protein like 1 (GABARAPL1), mRNA. | 2.399033612 | 1577 | 1477 | 1847 | 1895 | 1727 | 1245 | 1341 | 3656 | 3723 | 3690 | 3721 | 4026 | 4030 |
| 12749494 | GABARAPL2 | GABA(A) Receptor-Associated Protein Like 2 | 2.03266016 | 362 | 382 | 393 | 493 | 325 | 294 | 305 | 930 | 770 | 779 | 731 | 644 | 594 |
| 12893958 | GABBR2 | PREDICTED: gamma-aminobutyric acid (GABA) B receptor, 2 (GABBR2), partial mRNA. | 5.133917725 | 33 | 38 | 24 | 22 | 42 | 39 | 35 | 241 | 165 | 148 | 320 | 79 | 73 |
| 12843331 | GADD45A | growth arrest and DNA-damage-inducible, alpha (GADD45A), mRNA. | 13.18507548 | 33 | 36 | 49 | 35 | 98 | 69 | 73 | 690 | 555 | 588 | 902 | 929 | 774 |
| 12888026 | GADD45B | growth arrest and DNA-damage-inducible, beta (GADD45B), mRNA. | 2.224635665 | 1206 | 1056 | 1369 | 1024 | 634 | 391 | 829 | 1688 | 1777 | 1801 | 2270 | 2591 | 2285 |
| 12892887 | GADD45G | growth arrest and DNA-damage-inducible, gamma (GADD45G), mRNA. | 2.339086872 | 89 | 93 | 95 | 103 | 81 | 94 | 79 | 159 | 169 | 131 | 241 | 317 | 253 |
| 12696080 | GALC | galactosylceramidase (GALC), mRNA. | 4.738440581 | 26 | 28 | 34 | 36 | 99 | 103 | 102 | 331 | 234 | 271 | 296 | 306 | 297 |
| 12702773 | GALM | galactose mutarotase (aldose 1-epimerase) (GALM), mRNA. | 2.413805725 | 172 | 179 | 188 | 151 | 204 | 130 | 168 | 326 | 436 | 400 | 485 | 364 | 457 |
| 12690032 | GALNTL1 | UDP-N-acetyl-alpha-D-galactosamine:polypeptide N-acetylgalactosaminyltransferase-like 1 (GALNTL1), mRNA. | 5.715939891 | 87 | 82 | 104 | 74 | 75 | 84 | 64 | 634 | 448 | 518 | 611 | 228 | 349 |
| 12708304 | GBGT1 | globoside alpha-1,3-N-acetylgalactosaminyltransferase 1 (GBGT1), mRNA. | 2.016451204 | 141 | 154 | 127 | 116 | 110 | 102 | 154 | 250 | 181 | 246 | 354 | 251 | 281 |
| 12857338 | GCAT | glycine C-acetyltransferase (GCAT), nuclear gene encoding mitochondrial protein, mRNA. | 2.213826867 | 53 | 50 | 58 | 48 | 90 | 90 | 82 | 147 | 134 | 157 | 148 | 112 | 196 |
| 12765414 | GDPD1 | glycerophosphodiester phosphodiesterase domain containing 1 (GDPD1), mRNA. | 2.023691492 | 79 | 65 | 96 | 83 | 57 | 69 | 77 | 156 | 159 | 123 | 183 | 143 | 146 |
| 12723272 | GEM | GTP binding protein overexpressed in skeletal muscle (GEM), mRNA. | 7.887503861 | 132 | 106 | 130 | 123 | 310 | 247 | 260 | 1331 | 1497 | 1325 | 1576 | 1623 | 1484 |
| 12880314 | GFPT2 | glutamine-fructose-6-phosphate transaminase 2 (GFPT2), mRNA. | 22.23063947 | 63 | 59 | 84 | 28 | 248 | 159 | 250 | 2825 | 2827 | 2659 | 3034 | 2604 | 3044 |
| 12893753 | GFRA2 | GDNF family receptor alpha 2 (GFRA2), mRNA. | 3.326313461 | 30 | 28 | 33 | 26 | 66 | 53 | 46 | 159 | 82 | 92 | 208 | 168 | 97 |
| 12703901 | GGTA1P | Glycoprotein, Alpha-Galactosyltransferase 1 Pseudogene (GGTA1; GGTA1P) | 8.168668263 | 22 | 24 | 31 | 17 | 121 | 144 | 134 | 630 | 553 | 622 | 485 | 595 | 576 |
| 12825959 | GHITM | growth hormone inducible transmembrane protein (GHITM), mRNA. | 2.19815718 | 243 | 251 | 244 | 217 | 256 | 168 | 354 | 689 | 562 | 758 | 471 | 385 | 399 |
| 12786883 | GHR | Growth Hormone Receptor | 4.979798267 | 129 | 151 | 142 | 138 | 104 | 99 | 115 | 799 | 631 | 849 | 496 | 465 | 512 |
| 12846830 | GJA4 | Gap Junction Protein, Alpha 4, 37kDa | 4.276648893 | 54 | 59 | 83 | 60 | 105 | 149 | 149 | 781 | 502 | 493 | 218 | 223 | 202 |
| 12795499 | GMPPB | GDP-Mannose Pyrophosphorylase B | 3.256466844 | 188 | 161 | 147 | 163 | 105 | 87 | 109 | 408 | 438 | 504 | 340 | 481 | 507 |
| 12690792 | GNB5 | guanine nucleotide binding protein (G protein), beta 5 (GNB5), mRNA. | 2.231819814 | 184 | 200 | 222 | 184 | 192 | 160 | 221 | 397 | 449 | 475 | 487 | 344 | 457 |
| 12859179 | GNS | Glucosamine (N-Acetyl)-6-Sulfatase | 3.352406023 | 1118 | 1073 | 1409 | 1484 | 1266 | 1263 | 1308 | 4302 | 4155 | 4266 | 4614 | 3998 | 4299 |
| 12821398 | GOT1 | glutamic-oxaloacetic transaminase 1, soluble (aspartate aminotransferase 1) (GOT1), mRNA. | 2.260645305 | 194 | 214 | 314 | 199 | 229 | 221 | 368 | 706 | 494 | 742 | 463 | 426 | 541 |
| 12821591 | GPAM | Glycerol-3-Phosphate Acyltransferase, Mitochondrial | 5.282386494 | 140 | 147 | 136 | 200 | 160 | 211 | 128 | 840 | 794 | 989 | 932 | 735 | 795 |
| 12909853 | GPC4 | glypican 4 (GPC4), mRNA. | 4.502758753 | 60 | 51 | 51 | 53 | 121 | 113 | 125 | 430 | 331 | 356 | 393 | 320 | 385 |
| 12849788 | GPNMB | Glycoprotein (Transmembrane) Nmb | 18.74884547 | 51 | 39 | 225 | 29 | 95 | 68 | 38 | 735 | 983 | 579 | 1841 | 2472 | 2172 |
| 12816911 | GPRC5B | G protein-coupled receptor, family C, group 5, member B (GPRC5B), mRNA. | 10.71377556 | 42 | 35 | 45 | 28 | 104 | 103 | 52 | 826 | 682 | 629 | 451 | 628 | 551 |
| 12871189 | GPRIN3 | GPRIN Family Member 3 | 9.089180093 | 15 | 11 | 12 | 10 | 14 | 12 | 12 | 118 | 82 | 127 | 178 | 85 | 84 |
| 12864901 | GRASP | GRP1 (general receptor for phosphoinositides 1)-associated scaffold protein (GRASP), mRNA. | 4.909526819 | 40 | 37 | 44 | 43 | 46 | 41 | 39 | 265 | 183 | 206 | 198 | 221 | 146 |
| 12849431 | GRB10 | growth factor receptor-bound protein 10 (GRB10), mRNA. | 3.797157105 | 33 | 35 | 43 | 25 | 87 | 108 | 84 | 317 | 273 | 211 | 190 | 199 | 159 |
| 12708178 | GRHL1 | Grainyhead-Like Transcription Factor 1 | 2.154698647 | 103 | 95 | 117 | 97 | 117 | 134 | 89 | 181 | 229 | 276 | 227 | 159 | 317 |
| 12905032 | GRIA3 | glutamate receptor, ionotrophic, AMPA 3 (GRIA3), mRNA. | 8.026595527 | 39 | 32 | 44 | 37 | 36 | 41 | 33 | 689 | 429 | 490 | 93 | 46 | 55 |
| 12759917 | GYS1 | glycogen synthase 1 (muscle) (GYS1), mRNA. | 3.032300846 | 88 | 68 | 119 | 56 | 111 | 79 | 72 | 298 | 286 | 286 | 234 | 213 | 227 |
| 12890288 | HABP4 | hyaluronan binding protein 4 (HABP4), mRNA. | 2.764163562 | 200 | 188 | 156 | 164 | 128 | 125 | 122 | 394 | 355 | 654 | 418 | 332 | 413 |
| 12885817 | HBEGF | heparin-binding EGF-like growth factor (HBEGF), mRNA. | 3.796152769 | 61 | 41 | 39 | 46 | 52 | 71 | 60 | 288 | 266 | 252 | 128 | 130 | 134 |
| 12873900 | HERC3 | HECT and RLD domain containing E3 ubiquitin protein ligase 3 (HERC3), mRNA. | 3.056042804 | 57 | 70 | 77 | 71 | 74 | 71 | 67 | 197 | 175 | 238 | 193 | 249 | 224 |
| 12686304 | HES1 | hairy and enhancer of split 1, (Drosophila) (HES1), mRNA. | 8.646119429 | 26 | 24 | 25 | 17 | 48 | 85 | 47 | 553 | 527 | 432 | 174 | 193 | 137 |
| 12765052 | HEXIM1 | hexamethylene bis-acetamide inducible 1 (HEXIM1), mRNA. | 2.168849834 | 233 | 225 | 229 | 188 | 233 | 150 | 257 | 529 | 476 | 498 | 489 | 416 | 409 |
| 12840420 | HIST2H2BE | histone cluster 2, H2be (HIST2H2BE), mRNA. | 2.765738307 | 512 | 491 | 638 | 506 | 964 | 1042 | 1030 | 2195 | 2281 | 2276 | 1984 | 1713 | 1840 |
| 12881065 | HIST3H2A | histone cluster 3, H2a (HIST3H2A), mRNA. | 6.534810017 | 100 | 114 | 118 | 81 | 51 | 35 | 42 | 280 | 461 | 760 | 376 | 346 | 810 |
| 12901165 | HIVEP2 | PREDICTED: human immunodeficiency virus type I enhancer binding protein 2 (HIVEP2), mRNA. | 2.492144507 | 41 | 45 | 64 | 56 | 103 | 116 | 97 | 194 | 212 | 218 | 136 | 196 | 161 |
| 12785196 | HMGCS1 | HMGCS1 protein-like (HMGCS1), mRNA. | 2.750542607 | 1715 | 1721 | 1053 | 1182 | 1079 | 1532 | 1631 | 3644 | 3146 | 3849 | 4213 | 4027 | 4491 |
| 12812158 | HMOX2 | heme oxygenase (decycling) 2 (HMOX2), mRNA. | 2.748861479 | 253 | 257 | 251 | 214 | 284 | 238 | 209 | 656 | 646 | 757 | 636 | 586 | 740 |
| 12730451 | HSD17B12 | hydroxysteroid (17-beta) dehydrogenase 12 (HSD17B12), mRNA. | 2.451100143 | 349 | 400 | 319 | 266 | 541 | 523 | 523 | 849 | 1073 | 1231 | 972 | 590 | 1422 |
| 12843282 | HSD17B7 | hydroxysteroid (17-beta) dehydrogenase 7 (HSD17B7), mRNA. | 2.345380556 | 280 | 300 | 259 | 338 | 406 | 444 | 368 | 603 | 688 | 663 | 1010 | 804 | 1048 |
| 12842687 | HSD3B1 | hydroxy-delta-5-steroid dehydrogenase, 3 beta- and steroid delta-isomerase 1 (HSD3B1), mRNA. | 2.903322256 | 1965 | 2077 | 4525 | 1685 | 2895 | 2531 | 2805 | 7798 | 8754 | 8071 | 7830 | 6494 | 7055 |
| 12804546 | HSPA1A | heat shock 70kDa protein 1A (HSPA1A), mRNA. | 16.71880141 | 487 | 409 | 266 | 110 | 628 | 155 | 284 | 6323 | 6659 | 5588 | 4707 | 5139 | 5116 |
| 12688092 | HSPA2 | heat shock 70kDa protein 2 (HSPA2), mRNA. | 4.360200008 | 179 | 178 | 213 | 213 | 192 | 273 | 140 | 633 | 844 | 703 | 865 | 912 | 1228 |
| 12704208 | HSPA5 | heat shock 70kDa protein 5 (glucose-regulated protein, 78kDa) (HSPA5), mRNA. | 2.102866939 | 2722 | 2919 | 2314 | 2439 | 1747 | 1765 | 2319 | 4742 | 4721 | 4898 | 4580 | 5187 | 5120 |
| 12846968 | HSPA6 | PREDICTED: heat shock 70kDa protein 6 (HSP70B') (HSPA6), mRNA. | 10.97308077 | 18 | 16 | 18 | 18 | 14 | 15 | 15 | 234 | 235 | 217 | 133 | 136 | 113 |
| 12747823 | HSPB8 | heat shock 22kDa protein 8 (HSPB8), mRNA. | 4.868709126 | 29 | 23 | 28 | 27 | 112 | 105 | 87 | 470 | 465 | 314 | 161 | 184 | 123 |
| 12709282 | HSPH1 | heat shock 105kDa/110kDa protein 1 (HSPH1), mRNA. | 3.455852811 | 964 | 862 | 385 | 470 | 423 | 224 | 320 | 1965 | 2420 | 2073 | 1269 | 1642 | 1434 |
| 12873199 | IBSP | integrin-binding sialoprotein (IBSP), mRNA. | 83.35153727 | 10 | 11 | 9 | 9 | 8 | 8 | 8 | 535 | 2418 | 491 | 607 | 126 | 296 |
| 12876963 | ICAM1 | Intercellular Adhesion Molecule 1 | 8.370254694 | 28 | 17 | 27 | 19 | 223 | 275 | 167 | 871 | 940 | 1022 | 576 | 1021 | 990 |
| 12714001 | ID1 | inhibitor of DNA binding 1, dominant negative helix-loop-helix protein (ID1), mRNA. | 2.808409895 | 32 | 35 | 36 | 19 | 40 | 48 | 66 | 159 | 136 | 98 | 92 | 97 | 80 |
| 12909959 | IDS | iduronate 2-sulfatase (IDS), mRNA. | 2.855075257 | 188 | 206 | 213 | 179 | 230 | 167 | 152 | 593 | 473 | 560 | 630 | 480 | 531 |
| 12885965 | IER2 | immediate early response 2 (IER2), mRNA. | 8.057913245 | 63 | 57 | 89 | 53 | 63 | 57 | 69 | 446 | 505 | 552 | 513 | 477 | 622 |
| 12801358 | IER3 | immediate early response 3 (IER3), mRNA. | 16.66367214 | 90 | 115 | 126 | 130 | 155 | 136 | 147 | 1631 | 1395 | 1846 | 2377 | 2677 | 2906 |
| 12736300 | IER5 | Immediate Early Response 5 | 5.156895898 | 87 | 81 | 77 | 65 | 61 | 77 | 52 | 334 | 309 | 278 | 363 | 478 | 448 |
| 12683452 | IFNAR2 | interferon (alpha, beta and omega) receptor 2 (IFNAR2), mRNA. | 3.173747849 | 106 | 127 | 133 | 158 | 127 | 149 | 118 | 401 | 412 | 402 | 384 | 433 | 466 |
| 12855628 | IFRD1 | Interferon-Related Developmental Regulator 1 | 3.801797374 | 253 | 260 | 242 | 414 | 221 | 188 | 260 | 802 | 963 | 996 | 858 | 1220 | 1149 |
| 12780541 | IGFBP5 | insulin-like growth factor binding protein 5 (IGFBP5), mRNA. | 12.39404347 | 132 | 102 | 167 | 77 | 236 | 321 | 210 | 1548 | 1620 | 1267 | 2916 | 3408 | 2475 |
| 12798353 | IL17RC | interleukin 17 receptor C (IL17RC), mRNA. | 2.031000249 | 58 | 60 | 56 | 51 | 83 | 94 | 71 | 124 | 156 | 150 | 129 | 114 | 150 |
| 12703833 | IL1A | interleukin 1, alpha (IL1A), mRNA. | 13.64895875 | 10 | 10 | 12 | 9 | 11 | 10 | 16 | 154 | 140 | 164 | 225 | 121 | 112 |
| 12848835 | IL6 | interleukin 6 (interferon, beta 2) (IL6), mRNA. | 8.348285557 | 19 | 22 | 14 | 16 | 14 | 17 | 21 | 176 | 141 | 227 | 87 | 58 | 192 |
| 12785824 | IL6ST | PREDICTED: interleukin 6 signal transducer (gp130, oncostatin M receptor) (IL6ST), mRNA. | 2.946895578 | 531 | 564 | 547 | 750 | 1012 | 1220 | 1113 | 2129 | 2526 | 2231 | 2562 | 2323 | 2719 |
| 12850024 | INSIG1 | insulin induced gene 1 (INSIG1), mRNA. | 3.544518154 | 1068 | 965 | 606 | 422 | 552 | 499 | 586 | 2054 | 1573 | 2194 | 2642 | 2958 | 2854 |
| 12799059 | IRAK2 | interleukin-1 receptor-associated kinase 2 (IRAK2), mRNA. | 2.181186698 | 114 | 139 | 155 | 166 | 81 | 106 | 69 | 195 | 220 | 222 | 279 | 324 | 310 |
| 12865249 | IRAK3 | interleukin-1 receptor-associated kinase 3 (IRAK3), mRNA. | 3.733834958 | 40 | 35 | 36 | 19 | 219 | 319 | 242 | 457 | 463 | 386 | 572 | 478 | 560 |
| 12877288 | IRF1 | interferon regulatory factor 1 (IRF1), mRNA. | 14.79037992 | 63 | 64 | 65 | 60 | 137 | 109 | 154 | 812 | 1099 | 1121 | 1197 | 2086 | 1929 |
| 12742671 | ISCU | iron-sulfur cluster scaffold homolog (E. coli) (ISCU), nuclear gene encoding mitochondrial protein, mRNA. | 2.664458001 | 781 | 812 | 886 | 683 | 1570 | 1629 | 1908 | 3412 | 2911 | 2954 | 3537 | 3281 | 2790 |
| 12856953 | ITGA5 | integrin, alpha 5 (fibronectin receptor, alpha polypeptide) (ITGA5), mRNA. | 5.36776594 | 33 | 29 | 52 | 47 | 105 | 143 | 116 | 486 | 553 | 414 | 356 | 273 | 339 |
| 12796270 | ITGA9 | integrin, alpha 9 (ITGA9), mRNA. | 4.977031387 | 30 | 33 | 40 | 23 | 508 | 673 | 346 | 1090 | 1292 | 1130 | 1060 | 1217 | 1261 |
| 12768342 | ITGB4 | integrin, beta 4 (ITGB4), mRNA. | 2.416525796 | 38 | 32 | 36 | 32 | 41 | 53 | 53 | 104 | 131 | 112 | 80 | 98 | 63 |
| 12792677 | ITPK1 | inositol-tetrakisphosphate 1-kinase (ITPK1), mRNA. | 5.066989165 | 144 | 120 | 133 | 132 | 99 | 87 | 98 | 360 | 920 | 721 | 242 | 286 | 1000 |
| 12691986 | ITPKA | inositol-trisphosphate 3-kinase A (ITPKA), mRNA. | 4.144870723 | 87 | 77 | 105 | 77 | 188 | 270 | 189 | 560 | 544 | 622 | 795 | 391 | 616 |
| 12865396 | JOSD1 | Josephin domain containing 1 (JOSD1), mRNA. | 2.121288585 | 143 | 159 | 151 | 131 | 181 | 108 | 133 | 302 | 327 | 259 | 319 | 301 | 322 |
| 12725785 | JPH1 | junctophilin 1 (JPH1), mRNA. | 2.572698534 | 39 | 35 | 47 | 41 | 57 | 72 | 61 | 114 | 104 | 149 | 147 | 116 | 149 |
| 12835737 | JUN | jun proto-oncogene (JUN), mRNA. | 8.442234364 | 450 | 444 | 576 | 471 | 496 | 314 | 368 | 3529 | 3609 | 3422 | 3402 | 4670 | 3943 |
| 12885365 | JUNB | jun B proto-oncogene (JUNB), mRNA. | 15.89368568 | 269 | 232 | 315 | 139 | 156 | 193 | 217 | 3079 | 3100 | 3550 | 3264 | 3823 | 3895 |
| 12879314 | JUND | jun D proto-oncogene (JUND), mRNA. | 3.465765086 | 173 | 164 | 171 | 173 | 134 | 96 | 140 | 473 | 454 | 549 | 481 | 610 | 556 |
| 12884686 | KANK3 | KN motif and ankyrin repeat domains 3 (KANK3), mRNA. | 2.431504818 | 49 | 51 | 45 | 51 | 58 | 79 | 63 | 171 | 170 | 129 | 106 | 153 | 96 |
| 12738604 | KCNK2 | Potassium Channel, Two Pore Domain Subfamily K, Member 2 | 16.19241335 | 20 | 24 | 22 | 32 | 29 | 25 | 49 | 297 | 794 | 512 | 353 | 277 | 566 |
| 12706090 | KCNK3 | PREDICTED: potassium channel, subfamily K, member 3 (KCNK3), mRNA. | 3.114305851 | 50 | 46 | 76 | 54 | 120 | 145 | 139 | 279 | 317 | 195 | 322 | 353 | 215 |
| 12866439 | KCNMB4 | potassium large conductance calcium-activated channel, subfamily M, beta member 4 (KCNMB4), mRNA. | 4.255365404 | 27 | 19 | 21 | 18 | 31 | 37 | 29 | 87 | 191 | 143 | 85 | 53 | 105 |
| 12839963 | KCNN3 | PREDICTED: potassium intermediate/small conductance calcium-activated channel, subfamily N, member 3, transcript variant 2 (KCNN3), mRNA. | 7.914046056 | 16 | 13 | 12 | 12 | 14 | 15 | 11 | 123 | 171 | 159 | 83 | 56 | 46 |
| 12712968 | KCTD12 | PREDICTED: potassium channel tetramerisation domain containing 12 (KCTD12), mRNA. | 9.707293192 | 35 | 29 | 53 | 29 | 79 | 60 | 66 | 640 | 515 | 599 | 510 | 228 | 433 |
| 12760305 | KDM6B | Lysine (K)-Specific Demethylase 6B | 2.203837723 | 92 | 89 | 118 | 101 | 102 | 94 | 83 | 188 | 252 | 229 | 170 | 221 | 223 |
| 12714880 | KIAA1217 | KIAA1217 | 2.983414711 | 108 | 134 | 105 | 119 | 131 | 160 | 127 | 381 | 300 | 430 | 383 | 332 | 434 |
| 12721273 | KIAA1462 | KIAA1462 ortholog (KIAA1462), mRNA. | 2.627682457 | 42 | 44 | 52 | 39 | 59 | 75 | 53 | 183 | 190 | 149 | 117 | 102 | 78 |
| 12894778 | KIF27 | kinesin family member 27 (KIF27), mRNA. | 2.335649534 | 101 | 102 | 79 | 131 | 143 | 149 | 114 | 204 | 260 | 258 | 304 | 296 | 317 |
| 12869844 | KIT | v-kit Hardy-Zuckerman 4 feline sarcoma viral oncogene homolog (KIT), mRNA. | 8.296586918 | 20 | 28 | 24 | 39 | 88 | 131 | 268 | 325 | 600 | 264 | 841 | 1138 | 1083 |
| 12885748 | KLF2 | PREDICTED: Kruppel-like factor 2 (lung) (KLF2), mRNA. | 5.950845549 | 50 | 30 | 46 | 40 | 74 | 90 | 85 | 349 | 409 | 324 | 327 | 431 | 274 |
| 12894113 | KLF4 | Kruppel-like factor 4 (gut) (KLF4), mRNA. | 10.40625889 | 29 | 22 | 26 | 28 | 37 | 39 | 39 | 366 | 360 | 349 | 292 | 380 | 232 |
| 12714168 | KLF6 | Kruppel-like factor 6 (KLF6), mRNA. | 5.733773315 | 136 | 127 | 137 | 152 | 254 | 281 | 205 | 871 | 861 | 812 | 1176 | 1449 | 1179 |
| 12783426 | KLF7 | Kruppel-like factor 7 (ubiquitous) (KLF7), mRNA. | 2.278843347 | 186 | 186 | 161 | 122 | 229 | 314 | 228 | 475 | 455 | 358 | 427 | 591 | 480 |
| 12894967 | KLF9 | Kruppel-like factor 9 (KLF9), mRNA. | 4.254171376 | 26 | 28 | 41 | 26 | 102 | 94 | 90 | 274 | 244 | 262 | 237 | 255 | 216 |
| 12709032 | LAMP1 | lysosomal-associated membrane protein 1 (LAMP1), mRNA. | 2.261637893 | 542 | 496 | 550 | 564 | 804 | 615 | 510 | 1115 | 1198 | 1260 | 1357 | 1442 | 1540 |
| 12872678 | LAP3 | leucine aminopeptidase 3 (LAP3), mRNA. | 2.772949949 | 115 | 93 | 92 | 115 | 205 | 175 | 237 | 450 | 379 | 486 | 357 | 378 | 401 |
| 12876921 | LDLR | low density lipoprotein receptor (LDLR), mRNA. | 3.008744733 | 1481 | 1257 | 1382 | 674 | 1001 | 1149 | 1213 | 2952 | 2626 | 3408 | 3942 | 3573 | 4536 |
| 12679954 | LEPREL1 | leprecan-like 1 | 2.505990992 | 199 | 233 | 670 | 279 | 251 | 360 | 237 | 706 | 678 | 577 | 929 | 1122 | 772 |
| 12691569 | LGALS3 | lectin, galactoside-binding, soluble, 3 (LGALS3), mRNA. | 4.280764294 | 24 | 22 | 27 | 25 | 35 | 25 | 26 | 152 | 109 | 202 | 59 | 75 | 71 |
| 12764212 | LGALS3BP | lectin, galactoside-binding, soluble, 3 binding protein (LGALS3BP), mRNA. | 2.06236533 | 824 | 856 | 782 | 854 | 1225 | 691 | 1416 | 2257 | 1826 | 2230 | 1935 | 1645 | 1858 |
| 12791600 | LGMN | Legumain | 4.115758944 | 83 | 92 | 212 | 65 | 255 | 267 | 244 | 640 | 794 | 669 | 690 | 639 | 863 |
| 12742621 | LIMK2 | LIM Domain Kinase 2 | 2.127605267 | 69 | 54 | 63 | 58 | 125 | 101 | 102 | 192 | 188 | 150 | 199 | 165 | 153 |
| 12821141 | LIPA | lipase A, lysosomal acid, cholesterol esterase (LIPA), mRNA. | 2.526294154 | 291 | 306 | 502 | 172 | 304 | 256 | 451 | 870 | 759 | 983 | 879 | 651 | 802 |
| 12749602 | LIPE | lipase, hormone-sensitive (LIPE), mRNA. | 2.830349561 | 59 | 47 | 42 | 45 | 57 | 48 | 42 | 181 | 147 | 208 | 111 | 71 | 108 |
| 12807778 | LIPG | PREDICTED: lipase, endothelial (LIPG), mRNA. | 6.497985264 | 21 | 18 | 15 | 14 | 65 | 83 | 101 | 174 | 173 | 144 | 382 | 370 | 522 |
| 12817072 | LITAF | Lipopolysaccharide-Induced TNF Factor | 3.340471277 | 366 | 299 | 394 | 276 | 533 | 621 | 533 | 1344 | 1443 | 1373 | 1314 | 1638 | 1542 |
| 12842993 | LMNA | lamin A/C (LMNA), mRNA. | 2.592175595 | 112 | 127 | 143 | 131 | 275 | 288 | 232 | 460 | 555 | 606 | 438 | 443 | 404 |
| 12733478 | LMO2 | LIM Domain Only 2 (Rhombotin-Like 1) | 4.179970273 | 95 | 76 | 97 | 68 | 188 | 190 | 187 | 796 | 692 | 693 | 331 | 475 | 244 |
| 12794183 | LOC100294865 | PREDICTED: uncharacterized LOC100294865, transcript variant 2 (LOC100294865), mRNA. | 2.701757709 | 74 | 49 | 67 | 48 | 59 | 55 | 52 | 233 | 164 | 208 | 106 | 125 | 102 |
| 12824695 | LOC100295410 | cDNA clone IMAGE:8656618, partial cds. | 3.906681225 | 22 | 17 | 23 | 16 | 22 | 35 | 28 | 114 | 137 | 108 | 54 | 68 | 60 |
| 12835528 | LOC100296849 | PREDICTED: protein BEX3-like (LOC100296849), miscRNA. | 5.099463218 | 17 | 19 | 17 | 17 | 11 | 14 | 19 | 86 | 82 | 132 | 59 | 76 | 59 |
| 12693066 | LOC100297185 | cDNA clone IMAGE:8191093. | 2.791727685 | 47 | 48 | 37 | 33 | 35 | 41 | 41 | 77 | 96 | 114 | 110 | 143 | 135 |
| 12863581 | LOC100297676 | PREDICTED: C-type lectin domain family 2 member G-like (LOC100297676), mRNA. | 4.46586263 | 16 | 15 | 24 | 13 | 83 | 70 | 101 | 152 | 233 | 182 | 176 | 227 | 255 |
| 12738082 | LOC100299281 | cDNA clone IMAGE:8473920, partial cds. | 3.925485914 | 151 | 133 | 217 | 192 | 151 | 181 | 173 | 675 | 746 | 1120 | 587 | 218 | 685 |
| 12888944 | LOC100336666 | PREDICTED: uncharacterized LOC100336666 (LOC100336666), mRNA. | 5.823168763 | 48 | 58 | 38 | 25 | 52 | 53 | 55 | 141 | 227 | 269 | 292 | 307 | 407 |
| 12768239 | LOC506088 | PREDICTED: intercellular adhesion molecule 2-like (LOC506088), mRNA. | 5.2393158 | 19 | 18 | 18 | 14 | 19 | 23 | 25 | 141 | 149 | 164 | 42 | 53 | 61 |
| 12858297 | LOC506828 | cdna:known chromosome:UMD3.1:5:101210292:101266954:1 | 39.32095485 | 19 | 22 | 20 | 16 | 20 | 18 | 24 | 533 | 1200 | 612 | 1100 | 482 | 744 |
| 12858844 | LOC510193 | apolipoprotein L, 3-like (LOC510193), mRNA. | 6.30799088 | 26 | 26 | 19 | 22 | 35 | 73 | 63 | 407 | 337 | 264 | 164 | 168 | 86 |
| 12812361 | LOC512271 | cdna:known chromosome:UMD3.1:25:41273562:41295884:1 | 2.222362022 | 170 | 165 | 179 | 134 | 297 | 267 | 221 | 396 | 411 | 301 | 611 | 484 | 525 |
| 12801783 | LOC512672 | major histocompatibility complex, class I (LOC512672), mRNA. | 3.142570098 | 189 | 196 | 155 | 214 | 431 | 707 | 627 | 1122 | 1159 | 1166 | 1003 | 1247 | 1089 |
| 12879098 | LOC514750 | cdna:known chromosome:UMD3.1:7:63153227:63182560:1 | 2.384543377 | 62 | 50 | 82 | 61 | 62 | 69 | 59 | 156 | 165 | 178 | 138 | 124 | 150 |
| 12684882 | LOC518080 | PREDICTED: aTP-binding cassette, sub-family C (CFTR/MRP), member 1-like (LOC518080), mRNA. | 2.787923914 | 27 | 14 | 22 | 19 | 62 | 65 | 50 | 138 | 77 | 101 | 125 | 97 | 82 |
| 12822732 | LOC534506 | cdna:known chromosome:UMD3.1:26:44047737:44081511:-1 | 5.072961871 | 281 | 261 | 443 | 174 | 448 | 383 | 560 | 1951 | 1874 | 1663 | 2146 | 1637 | 1816 |
| 12901415 | LOC616198 | PREDICTED: uncharacterized LOC616198 (LOC616198), mRNA. | 6.007498288 | 17 | 20 | 23 | 20 | 60 | 55 | 39 | 140 | 128 | 161 | 198 | 296 | 281 |
| 12892094 | LOC783399 | PREDICTED: major allergen Equ c 1-like, transcript variant 2 (LOC783399), mRNA. | 12.03470361 | 9 | 19 | 9 | 10 | 21 | 38 | 23 | 405 | 198 | 137 | 214 | 90 | 281 |
| 12863031 | LOC784451 | PREDICTED: C-type lectin domain family 2 member D11-like (LOC784451), mRNA. | 4.736499201 | 25 | 18 | 27 | 16 | 46 | 83 | 85 | 302 | 327 | 241 | 106 | 139 | 102 |
| 12793437 | LOC787624 | cdna:known chromosome:UMD3.1:21:24596916:24836070:-1 | 4.480402943 | 23 | 25 | 21 | 19 | 30 | 29 | 29 | 156 | 107 | 160 | 114 | 64 | 73 |
| 12847982 | LOC788268 | cdna:known chromosome:UMD3.1:3:6938875:6939732:-1 | 7.931355089 | 25 | 16 | 26 | 32 | 273 | 336 | 499 | 1492 | 1456 | 1708 | 1320 | 1019 | 1213 |
| 12903516 | LONRF3 | LON peptidase N-terminal domain and ring finger 3 (LONRF3), mRNA. | 5.5510306 | 22 | 26 | 31 | 24 | 47 | 42 | 48 | 135 | 244 | 288 | 115 | 109 | 248 |
| 12751621 | LPCAT2 | lysophosphatidylcholine acyltransferase 2 (LPCAT2), mRNA. | 3.342822954 | 25 | 20 | 31 | 28 | 121 | 162 | 146 | 251 | 359 | 269 | 169 | 239 | 237 |
| 12859656 | LPCAT3 | lysophosphatidylcholine acyltransferase 3 (LPCAT3), mRNA. | 2.17721746 | 576 | 641 | 640 | 402 | 718 | 512 | 700 | 1257 | 1006 | 984 | 1683 | 1145 | 1741 |
| 12707198 | LPIN1 | lipin 1 (LPIN1), mRNA. | 7.153172698 | 117 | 136 | 88 | 94 | 89 | 119 | 85 | 495 | 506 | 1081 | 626 | 467 | 1288 |
| 12889017 | LPL | Lipoprotein Lipase | 6.274391935 | 107 | 132 | 106 | 137 | 574 | 470 | 742 | 2272 | 1807 | 2660 | 1921 | 1446 | 2091 |
| 12840607 | LRIF1 | chromosome 1 open reading frame 103 ortholog, mRNA (cDNA clone MGC:148531 IMAGE:8289640), complete cds. | 2.022692419 | 198 | 173 | 173 | 168 | 218 | 229 | 204 | 379 | 380 | 359 | 437 | 427 | 381 |
| 12733526 | LRRC32 | leucine rich repeat containing 32 (LRRC32), mRNA. | 3.713746203 | 29 | 21 | 28 | 21 | 41 | 54 | 46 | 191 | 204 | 152 | 65 | 85 | 69 |
| 12841361 | LRRFIP1 | leucine rich repeat (in FLII) interacting protein 1 (LRRFIP1), mRNA. | 2.926103228 | 59 | 43 | 40 | 40 | 86 | 109 | 88 | 187 | 274 | 229 | 102 | 173 | 202 |
| 12855476 | LRRN3 | PREDICTED: leucine rich repeat neuronal 3 (LRRN3), mRNA. | 9.005667914 | 20 | 22 | 18 | 25 | 16 | 30 | 22 | 100 | 103 | 459 | 125 | 86 | 303 |
| 12686938 | LSS | lanosterol synthase (2,3-oxidosqualene-lanosterol cyclase) (LSS), mRNA. | 2.121037928 | 502 | 449 | 441 | 325 | 544 | 435 | 501 | 848 | 816 | 825 | 1198 | 989 | 1134 |
| 12895371 | LURAP1L | leucine rich adaptor protein 1-like (LURAP1L), mRNA. | 2.575670232 | 31 | 30 | 24 | 25 | 37 | 48 | 46 | 144 | 75 | 108 | 70 | 80 | 55 |
| 12776740 | LUZP1 | leucine zipper protein 1 (LUZP1), mRNA. | 2.081213532 | 117 | 125 | 129 | 97 | 164 | 156 | 129 | 301 | 358 | 268 | 213 | 250 | 246 |
| 12777791 | LYPD1 | LY6/PLAUR domain containing 1 (LYPD1), mRNA. | 12.3173944 | 45 | 38 | 34 | 30 | 33 | 29 | 32 | 205 | 916 | 349 | 422 | 215 | 450 |
| 12902272 | MAMLD1 | Mastermind-Like Domain Containing 1 | 2.036687249 | 110 | 114 | 134 | 73 | 144 | 152 | 126 | 236 | 298 | 180 | 312 | 244 | 219 |
| 12897782 | MAN1A1 | mannosidase, alpha, class 1A, member 1 (MAN1A1), mRNA. | 2.75646958 | 258 | 254 | 239 | 238 | 517 | 421 | 329 | 830 | 880 | 729 | 1037 | 1074 | 784 |
| 12877062 | MAN2B1 | mannosidase, alpha, class 2B, member 1 (MAN2B1), mRNA. | 5.126628129 | 189 | 209 | 219 | 131 | 372 | 379 | 262 | 1115 | 1209 | 806 | 1592 | 1508 | 1507 |
| 12749707 | MAP1LC3B | microtubule-associated protein 1 light chain 3 beta (MAP1LC3B), mRNA. | 2.328373775 | 183 | 178 | 189 | 231 | 150 | 149 | 116 | 537 | 343 | 473 | 359 | 336 | 339 |
| 12716461 | MAP3K8 | mitogen-activated protein kinase kinase kinase 8 (MAP3K8), mRNA. | 23.3740327 | 26 | 25 | 33 | 33 | 39 | 23 | 35 | 545 | 544 | 555 | 592 | 1053 | 987 |
| 12834051 | MAP4K2 | mitogen-activated protein kinase kinase kinase kinase 2 (MAP4K2), mRNA. | 2.543299303 | 46 | 51 | 64 | 51 | 71 | 70 | 53 | 171 | 233 | 148 | 117 | 122 | 88 |
| 12844285 | MAP7D1 | MAP7 Domain Containing 1 | 2.158871368 | 100 | 93 | 95 | 100 | 165 | 178 | 158 | 281 | 333 | 305 | 257 | 236 | 235 |
| 12800454 | MAPKAPK3 | mitogen-activated protein kinase-activated protein kinase 3 (MAPKAPK3), mRNA. | 2.675151244 | 102 | 113 | 120 | 99 | 107 | 91 | 103 | 269 | 299 | 255 | 332 | 240 | 286 |
| 12760750 | MAPT | microtubule-associated protein tau (MAPT), mRNA. | 4.15283321 | 28 | 29 | 29 | 27 | 28 | 35 | 37 | 76 | 121 | 104 | 117 | 169 | 169 |
| 12878322 | MARCH2 | membrane-associated ring finger (C3HC4) 2 (MARCH2), mRNA. | 2.08279586 | 181 | 180 | 259 | 153 | 226 | 176 | 144 | 569 | 298 | 468 | 383 | 343 | 293 |
| 12879457 | MARCH3 | membrane-associated ring finger (C3HC4) 3 (MARCH3), mRNA. | 14.87008426 | 15 | 32 | 20 | 20 | 23 | 34 | 31 | 225 | 327 | 270 | 334 | 502 | 574 |
| 12787849 | MAST4 | Microtubule Associated Serine/Threonine Kinase Family Member 4 | 3.455817367 | 35 | 31 | 26 | 27 | 52 | 72 | 60 | 165 | 177 | 156 | 143 | 112 | 144 |
| 12909914 | MBNL3 | muscleblind-like 3 (Drosophila) (MBNL3), mRNA. | 5.246948245 | 64 | 71 | 71 | 63 | 114 | 114 | 147 | 472 | 501 | 376 | 464 | 353 | 731 |
| 12841926 | MCL1 | myeloid cell leukemia sequence 1 (BCL2-related) (MCL1), nuclear gene encoding mitochondrial protein, mRNA. | 3.307756999 | 244 | 208 | 229 | 158 | 295 | 276 | 275 | 773 | 731 | 745 | 795 | 1025 | 709 |
| 12894596 | MEGF9 | Multiple EGF-Like-Domains 9 | 3.491462502 | 58 | 61 | 63 | 47 | 180 | 191 | 194 | 365 | 361 | 346 | 399 | 454 | 450 |
| 12704003 | MGAT4A | mannosyl (alpha-1,3-)-glycoprotein beta-1,4-N-acetylglucosaminyltransferase, isozyme A (MGAT4A), mRNA. | 5.910897268 | 26 | 37 | 29 | 23 | 24 | 31 | 24 | 111 | 151 | 112 | 211 | 205 | 191 |
| 12756707 | MGC139164 | Solute Carrier Family 7 (Cationic Amino Acid Transporter, Y+ System), Member 3 Pseudogene (LOC284379) | 4.085757663 | 17 | 20 | 20 | 32 | 49 | 66 | 55 | 204 | 172 | 210 | 86 | 149 | 82 |
| 12910177 | MID1IP1 | MID1 interacting protein 1 (gastrulation specific G12 homolog (zebrafish)) (MID1IP1), mRNA. | 5.308228627 | 305 | 295 | 255 | 186 | 282 | 221 | 230 | 1380 | 1267 | 1177 | 1386 | 1621 | 1246 |
| 12879792 | MIDN | Midnolin | 2.333354244 | 92 | 82 | 94 | 68 | 98 | 76 | 91 | 237 | 231 | 213 | 191 | 182 | 148 |
| 12734842 | MIR 6121 | miRNA 6121 | 8.052211884 | 144 | 144 | 130 | 113 | 247 | 251 | 206 | 1921 | 1885 | 1553 | 1314 | 1004 | 853 |
| 12888606 | MIR23A | microRNA mir-23a (MIR23A), microRNA. | 3.517884185 | 33 | 26 | 25 | 12 | 28 | 21 | 31 | 98 | 132 | 94 | 40 | 87 | 75 |
| 12876521 | MIR2456 | microRNA mir-2456 (MIR2456), microRNA. | 2.453221208 | 49 | 28 | 53 | 39 | 54 | 78 | 57 | 82 | 181 | 113 | 140 | 122 | 115 |
| 12797806 | MIR885 | microRNA mir-885 (MIR885), microRNA. | 2.021864956 | 39 | 57 | 53 | 51 | 43 | 52 | 56 | 111 | 82 | 72 | 134 | 71 | 137 |
| 12798204 | MITF | microphthalmia-associated transcription factor (MITF), mRNA. | 2.532043837 | 61 | 74 | 67 | 77 | 93 | 116 | 80 | 159 | 181 | 136 | 234 | 279 | 245 |
| 12748301 | MLXIP | MLX interacting protein (MLXIP), mRNA. | 2.18536276 | 116 | 104 | 102 | 70 | 124 | 122 | 89 | 159 | 216 | 179 | 230 | 271 | 307 |
| 12726834 | MMP7 | matrix metallopeptidase 7 (matrilysin, uterine) (MMP7), mRNA. | 8.289599103 | 10 | 9 | 8 | 9 | 7 | 9 | 8 | 42 | 39 | 32 | 116 | 87 | 101 |
| 12874324 | MMRN1 | multimerin 1 (MMRN1), mRNA. | 9.941348267 | 18 | 18 | 19 | 22 | 87 | 141 | 32 | 567 | 848 | 370 | 316 | 531 | 240 |
| 12907669 | MOSPD2 | motile sperm domain containing 2 (MOSPD2), mRNA. | 2.472174198 | 128 | 112 | 107 | 128 | 115 | 144 | 130 | 261 | 222 | 237 | 348 | 385 | 379 |
| 12903657 | MPP1 | membrane protein, palmitoylated 1, 55kDa (MPP1), mRNA. | 4.576800296 | 169 | 173 | 148 | 205 | 363 | 422 | 327 | 886 | 886 | 640 | 1790 | 1406 | 1483 |
| 12858434 | MPST | mercaptopyruvate sulfurtransferase (MPST), nuclear gene encoding mitochondrial protein, transcript variant 1, mRNA. | 2.279460999 | 91 | 78 | 89 | 73 | 218 | 163 | 138 | 293 | 256 | 288 | 291 | 288 | 248 |
| 12812824 | MSLN | mesothelin (MSLN), mRNA. | 8.765602051 | 18 | 18 | 21 | 17 | 23 | 25 | 22 | 137 | 145 | 133 | 331 | 87 | 255 |
| 12795644 | MST1 | macrophage stimulating 1 (hepatocyte growth factor-like) (MST1), mRNA. | 4.20090625 | 66 | 48 | 82 | 49 | 47 | 45 | 40 | 157 | 198 | 168 | 322 | 246 | 268 |
| 12706002 | MTHFD2 | methylenetetrahydrofolate dehydrogenase (NADP+ dependent) 2, methenyltetrahydrofolate cyclohydrolase (MTHFD2), nuclear gene encoding mitochondrial protein, mRNA. | 2.583289421 | 180 | 171 | 158 | 123 | 136 | 83 | 151 | 205 | 292 | 342 | 315 | 423 | 641 |
| 12722787 | MTSS1 | metastasis suppressor 1 (MTSS1), mRNA. | 2.252141012 | 129 | 158 | 180 | 133 | 174 | 169 | 137 | 433 | 444 | 406 | 250 | 265 | 286 |
| 12824009 | MTUS1 | microtubule associated tumor suppressor 1 (MTUS1), nuclear gene encoding mitochondrial protein, mRNA. | 4.678747561 | 29 | 41 | 47 | 31 | 66 | 89 | 82 | 301 | 337 | 344 | 175 | 205 | 184 |
| 12756748 | MVD | mevalonate (diphospho) decarboxylase (MVD), mRNA. | 2.433965563 | 123 | 122 | 94 | 82 | 147 | 122 | 116 | 210 | 196 | 259 | 413 | 253 | 352 |
| 12816921 | MVP | major vault protein (MVP), mRNA. | 2.311082628 | 98 | 117 | 130 | 113 | 179 | 149 | 139 | 293 | 265 | 256 | 401 | 317 | 302 |
| 12700012 | MXD1 | MAX dimerization protein 1 (MXD1), mRNA. | 4.958623134 | 106 | 127 | 119 | 91 | 113 | 83 | 78 | 620 | 593 | 641 | 392 | 401 | 399 |
| 12806585 | MYLIP | myosin regulatory light chain interacting protein (MYLIP), mRNA. | 2.294113471 | 140 | 163 | 138 | 185 | 198 | 278 | 217 | 413 | 583 | 404 | 319 | 477 | 396 |
| 12710632 | N4BP2L1 | NEDD4 binding protein 2-like 1 (N4BP2L1), mRNA. | 2.201666665 | 112 | 125 | 143 | 138 | 153 | 218 | 126 | 352 | 366 | 312 | 238 | 382 | 265 |
| 12781710 | NAB1 | NGFI-A binding protein 1 (EGR1 binding protein 1) (NAB1), mRNA. | 2.049107263 | 128 | 124 | 123 | 157 | 146 | 143 | 156 | 379 | 285 | 329 | 238 | 230 | 256 |
| 12854527 | NAMPT | nicotinamide phosphoribosyltransferase (NAMPT), mRNA. | 2.511319599 | 198 | 209 | 213 | 280 | 201 | 179 | 223 | 507 | 547 | 655 | 443 | 500 | 584 |
| 12870674 | NAP1L5 | Nucleosome Assembly Protein 1-Like 5 | 5.381987209 | 47 | 43 | 50 | 46 | 64 | 55 | 59 | 186 | 211 | 381 | 240 | 152 | 512 |
| 12759721 | NAPA | N-ethylmaleimide-sensitive factor attachment protein, alpha (NAPA), mRNA. | 2.109835265 | 241 | 236 | 276 | 146 | 278 | 219 | 182 | 496 | 506 | 501 | 536 | 354 | 459 |
| 12721639 | NCALD | Neurocalcin Delta | 8.263679413 | 19 | 12 | 18 | 16 | 39 | 33 | 40 | 192 | 243 | 168 | 169 | 148 | 328 |
| 12762713 | NDEL1 | nudE nuclear distribution gene E homolog (A. nidulans)-like 1 (NDEL1), mRNA. | 2.444064792 | 159 | 177 | 189 | 157 | 112 | 123 | 133 | 366 | 382 | 441 | 325 | 349 | 338 |
| 12871222 | NDNF | neuron-derived neurotrophic factor (NDNF), mRNA. | 7.686580883 | 51 | 37 | 57 | 54 | 88 | 68 | 75 | 944 | 688 | 821 | 118 | 120 | 149 |
| 12721948 | NDRG1 | N-myc downstream regulated 1 (NDRG1), mRNA. | 7.057171078 | 52 | 50 | 43 | 38 | 196 | 144 | 153 | 498 | 657 | 688 | 589 | 888 | 765 |
| 12700140 | NEK6 | NIMA (never in mitosis gene a)-related kinase 6 (NEK6), mRNA. | 7.481771267 | 16 | 22 | 27 | 28 | 77 | 86 | 112 | 548 | 487 | 494 | 286 | 201 | 352 |
| 12889469 | NFIL3 | nuclear factor, interleukin 3 regulated (NFIL3), mRNA. | 17.07617872 | 57 | 57 | 52 | 60 | 56 | 81 | 47 | 864 | 806 | 867 | 1047 | 1174 | 1242 |
| 12820145 | NFKB2 | nuclear factor of kappa light polypeptide gene enhancer in B-cells 2 (p49/p100) (NFKB2), mRNA. | 2.004907629 | 91 | 81 | 86 | 94 | 102 | 95 | 109 | 159 | 172 | 191 | 159 | 206 | 243 |
| 12791746 | NFKBIA | nuclear factor of kappa light polypeptide gene enhancer in B-cells inhibitor, alpha (NFKBIA), mRNA. | 12.71078863 | 122 | 116 | 110 | 102 | 234 | 199 | 179 | 1508 | 1558 | 1723 | 2111 | 2225 | 2437 |
| 12678828 | NFKBIZ | nuclear factor of kappa light polypeptide gene enhancer in B-cells inhibitor, zeta | 13.41531979 | 127 | 146 | 155 | 127 | 179 | 245 | 168 | 1730 | 2021 | 2152 | 2153 | 2594 | 2542 |
| 12711403 | NHLRC3 | NHL repeat containing 3 (NHLRC3), mRNA. | 3.151918185 | 80 | 95 | 89 | 132 | 95 | 113 | 108 | 256 | 219 | 158 | 451 | 464 | 375 |
| 12693390 | NPC2 | Niemann-Pick disease, type C2 (NPC2), mRNA. | 2.38241682 | 275 | 281 | 305 | 353 | 510 | 547 | 555 | 823 | 920 | 851 | 1054 | 1009 | 1113 |
| 12697937 | NPDC1 | neural proliferation, differentiation and control, 1 (NPDC1), mRNA. | 4.873045474 | 25 | 18 | 18 | 15 | 54 | 103 | 69 | 266 | 271 | 260 | 137 | 205 | 118 |
| 12805169 | NQO2 | NAD(P)H dehydrogenase, quinone 2 (NQO2), mRNA. | 2.904102995 | 58 | 51 | 63 | 54 | 83 | 72 | 80 | 139 | 171 | 180 | 163 | 169 | 329 |
| 12773218 | NR1D1 | nuclear receptor subfamily 1, group D, member 1 (NR1D1), mRNA. | 6.640655143 | 45 | 33 | 33 | 33 | 63 | 58 | 40 | 322 | 256 | 364 | 299 | 185 | 315 |
| 12866799 | NR4A1 | nuclear receptor subfamily 4, group A, member 1 (NR4A1), mRNA. | 17.60554821 | 42 | 61 | 50 | 37 | 222 | 144 | 236 | 1811 | 1903 | 2029 | 2276 | 2096 | 1840 |
| 12778619 | NR4A2 | nuclear receptor subfamily 4, group A, member 2 (NR4A2), mRNA. | 3.618950208 | 56 | 58 | 50 | 44 | 57 | 58 | 64 | 242 | 254 | 205 | 192 | 168 | 142 |
| 12905830 | NSDHL | NAD(P) dependent steroid dehydrogenase-like (NSDHL), mRNA. | 2.520962403 | 956 | 943 | 699 | 513 | 756 | 804 | 860 | 1559 | 1772 | 1902 | 2180 | 1957 | 2581 |
| 12866509 | NT5DC3 | PREDICTED: 5'-nucleotidase domain containing 3 (NT5DC3), mRNA. | 5.070461857 | 36 | 36 | 28 | 24 | 263 | 176 | 194 | 563 | 412 | 409 | 721 | 650 | 537 |
| 12750040 | NUCB1 | nucleobindin 1 (NUCB1), mRNA. | 2.358521793 | 556 | 665 | 724 | 565 | 1369 | 1180 | 1061 | 1807 | 1868 | 1693 | 2255 | 2287 | 2464 |
| 12862277 | NUDT4 | nudix (nucleoside diphosphate linked moiety X)-type motif 4 (NUDT4), mRNA. | 4.529866749 | 450 | 398 | 415 | 407 | 439 | 534 | 614 | 1415 | 1963 | 1460 | 2462 | 2223 | 3122 |
| 12873639 | OCIAD2 | OCIA domain containing 2 (OCIAD2), mRNA. | 5.72529998 | 31 | 34 | 35 | 44 | 174 | 131 | 143 | 441 | 460 | 593 | 572 | 358 | 478 |
| 12830194 | ODZ4 | PREDICTED: odz, odd Oz/ten-m homolog 4 (Drosophila) (ODZ4), mRNA. | 5.486326243 | 35 | 24 | 46 | 26 | 38 | 46 | 46 | 277 | 199 | 240 | 206 | 130 | 176 |
| 12856897 | OLR1 | oxidized low density lipoprotein (lectin-like) receptor 1 (OLR1), mRNA. | 18.07359704 | 15 | 15 | 17 | 23 | 16 | 22 | 25 | 271 | 220 | 238 | 508 | 240 | 575 |
| 12816321 | ORAI2 | ORAI calcium release-activated calcium modulator 2 (ORAI2), mRNA. | 3.579085365 | 58 | 58 | 65 | 45 | 67 | 75 | 66 | 199 | 123 | 173 | 311 | 263 | 264 |
| 12724640 | OSR2 | odd-skipped related 2 (Drosophila) (OSR2), mRNA. | 5.793822089 | 32 | 28 | 33 | 30 | 288 | 233 | 155 | 342 | 543 | 315 | 852 | 1021 | 895 |
| 12889132 | OSTF1 | osteoclast stimulating factor 1 (OSTF1), mRNA. | 2.941961487 | 86 | 106 | 131 | 119 | 225 | 240 | 308 | 664 | 539 | 693 | 443 | 342 | 384 |
| 12897367 | OSTM1 | osteopetrosis associated transmembrane protein 1 (OSTM1), mRNA. | 2.13851045 | 109 | 131 | 197 | 213 | 136 | 117 | 116 | 355 | 347 | 396 | 317 | 218 | 236 |
| 12716096 | OTUD1 | PREDICTED: OTU domain containing 1, transcript variant 2 (OTUD1), mRNA. | 2.661466788 | 69 | 56 | 70 | 68 | 30 | 30 | 30 | 201 | 134 | 160 | 95 | 109 | 107 |
| 12726830 | P2RY2 | purinergic receptor P2Y, G-protein coupled, 2 (P2RY2), mRNA. | 2.426456346 | 92 | 73 | 78 | 61 | 68 | 74 | 81 | 247 | 228 | 214 | 168 | 124 | 116 |
| 12758260 | PAF1 | PAF1 Homolog, Paf1/RNA Polymerase II Complex Component | 2.205669139 | 305 | 286 | 278 | 252 | 266 | 183 | 234 | 623 | 539 | 696 | 493 | 515 | 546 |
| 12703330 | PAIP2B | Poly(A) Binding Protein Interacting Protein 2B | 3.21721874 | 56 | 81 | 76 | 82 | 61 | 55 | 55 | 182 | 174 | 162 | 247 | 278 | 246 |
| 12732039 | PAMR1 | peptidase domain containing associated with muscle regeneration 1 (PAMR1), mRNA. | 16.32798776 | 29 | 26 | 27 | 23 | 31 | 27 | 28 | 166 | 322 | 184 | 545 | 655 | 806 |
| 12871206 | PARM1 | prostate androgen-regulated mucin-like protein 1 (PARM1), mRNA. | 14.5504686 | 32 | 29 | 29 | 29 | 49 | 57 | 54 | 1194 | 912 | 1041 | 118 | 127 | 85 |
| 12798624 | PARP3 | Poly (ADP-Ribose) Polymerase Family, Member 3 | 3.496662883 | 44 | 54 | 46 | 42 | 75 | 81 | 68 | 141 | 181 | 167 | 214 | 263 | 265 |
| 12836974 | PBXIP1 | pre-B-cell leukemia homeobox interacting protein 1 (PBXIP1), mRNA. | 2.904963061 | 143 | 119 | 134 | 127 | 234 | 247 | 224 | 412 | 399 | 393 | 617 | 639 | 597 |
| 12880370 | PCDHGB4 | protocadherin gamma subfamily B, 4 (PCDHGB4), mRNA. | 2.342703111 | 73 | 97 | 126 | 64 | 86 | 90 | 64 | 161 | 191 | 153 | 157 | 300 | 242 |
| 12820218 | PCGF5 | polycomb group ring finger 5 (PCGF5), mRNA. | 2.002686843 | 155 | 149 | 170 | 191 | 118 | 100 | 111 | 230 | 271 | 243 | 266 | 344 | 354 |
| 12725399 | PCMTD1 | protein-L-isoaspartate (D-aspartate) O-methyltransferase domain containing 1 (PCMTD1), mRNA. | 2.708479353 | 287 | 268 | 234 | 313 | 454 | 476 | 443 | 951 | 922 | 898 | 964 | 1110 | 904 |
| 12883237 | PCSK1 | Proprotein Convertase Subtilisin/Kexin Type 1 | 34.00728978 | 24 | 21 | 29 | 26 | 18 | 24 | 24 | 805 | 968 | 567 | 1105 | 550 | 837 |
| 12762576 | PCYT2 | phosphate cytidylyltransferase 2, ethanolamine (PCYT2), mRNA. | 2.270914892 | 262 | 246 | 197 | 212 | 185 | 176 | 164 | 373 | 423 | 489 | 564 | 357 | 602 |
| 12732706 | PDE3B | PREDICTED: phosphodiesterase 3B, cGMP-inhibited (PDE3B), mRNA. | 3.054350905 | 29 | 23 | 26 | 26 | 50 | 40 | 39 | 96 | 107 | 96 | 70 | 62 | 178 |
| 12728524 | PDGFD | platelet derived growth factor D (PDGFD), mRNA. | 8.381786552 | 51 | 35 | 38 | 38 | 87 | 99 | 117 | 510 | 557 | 412 | 532 | 667 | 661 |
| 12907350 | PDHA1 | pyruvate dehydrogenase (lipoamide) alpha 1 (PDHA1), nuclear gene encoding mitochondrial protein, mRNA. | 2.369534508 | 385 | 358 | 409 | 358 | 426 | 429 | 438 | 974 | 1062 | 965 | 1078 | 747 | 869 |
| 12853639 | PDK4 | pyruvate dehydrogenase kinase, isozyme 4 (PDK4), nuclear gene encoding mitochondrial protein, mRNA. | 9.464724361 | 56 | 64 | 122 | 67 | 574 | 784 | 754 | 2817 | 2740 | 2790 | 3657 | 4177 | 3461 |
| 12873486 | PDLIM5 | PDZ and LIM domain 5 (PDLIM5), mRNA. | 3.131830703 | 179 | 171 | 321 | 176 | 235 | 300 | 212 | 701 | 672 | 900 | 516 | 748 | 740 |
| 12739170 | PDPN | Podoplanin | 15.09630218 | 35 | 39 | 61 | 58 | 244 | 364 | 209 | 2633 | 2538 | 2279 | 1924 | 1599 | 2097 |
| 12707062 | PELI1 | pellino homolog 1 (Drosophila) (PELI1), mRNA. | 2.188192505 | 329 | 337 | 348 | 306 | 285 | 350 | 350 | 704 | 792 | 866 | 644 | 639 | 681 |
| 12866000 | PEX5 | peroxisomal biogenesis factor 5 (PEX5), mRNA. | 2.019844638 | 88 | 79 | 92 | 78 | 97 | 77 | 80 | 139 | 172 | 160 | 167 | 158 | 226 |
| 12734691 | PFKFB2 | 6-phosphofructo-2-kinase/fructose-2,6-biphosphatase 2 (PFKFB2), mRNA. | 2.190607493 | 81 | 90 | 95 | 79 | 123 | 111 | 113 | 160 | 199 | 199 | 207 | 234 | 301 |
| 12718246 | PFKFB3 | 6-phosphofructo-2-kinase/fructose-2,6-biphosphatase 3 (PFKFB3), mRNA. | 3.074060953 | 81 | 59 | 75 | 61 | 51 | 37 | 31 | 152 | 148 | 186 | 184 | 196 | 175 |
| 12887724 | PGPEP1 | pyroglutamyl-peptidase I (PGPEP1), mRNA. | 2.79868547 | 71 | 89 | 93 | 81 | 290 | 244 | 305 | 451 | 494 | 459 | 569 | 430 | 413 |
| 12867979 | PHLDA1 | pleckstrin homology-like domain, family A, member 1 (PHLDA1), mRNA. | 2.298300456 | 78 | 76 | 65 | 66 | 108 | 84 | 83 | 225 | 214 | 200 | 187 | 126 | 151 |
| 12717982 | PHYH | Phytanoyl-CoA 2-Hydroxylase | 3.842029139 | 174 | 154 | 221 | 175 | 274 | 244 | 279 | 1054 | 855 | 1301 | 676 | 508 | 615 |
| 12836111 | PIK3R3 | Phosphoinositide-3-Kinase, Regulatory Subunit 3 (Gamma) | 4.346892088 | 19 | 28 | 34 | 33 | 49 | 62 | 56 | 187 | 229 | 189 | 146 | 137 | 157 |
| 12804472 | PIM1 | Pim-1 Proto-Oncogene, Serine/Threonine Kinase | 4.776086984 | 104 | 101 | 66 | 67 | 139 | 100 | 83 | 239 | 259 | 254 | 499 | 866 | 587 |
| 12907694 | PIM2 | pim-2 oncogene (PIM2), mRNA. | 3.605937486 | 158 | 167 | 202 | 127 | 113 | 80 | 103 | 627 | 575 | 648 | 583 | 226 | 276 |
| 12856550 | PIM3 | Pim-3 Proto-Oncogene, Serine/Threonine Kinase | 3.27052652 | 139 | 130 | 105 | 106 | 107 | 97 | 136 | 607 | 310 | 676 | 310 | 159 | 237 |
| 12743075 | PITPNM2 | phosphatidylinositol transfer protein, membrane-associated 2 (PITPNM2), mRNA. | 2.073559128 | 44 | 49 | 53 | 42 | 62 | 64 | 67 | 166 | 121 | 97 | 102 | 93 | 97 |
| 12871398 | PLA2G12A | phospholipase A2, group XIIA (PLA2G12A), mRNA. | 2.729931857 | 152 | 188 | 216 | 184 | 215 | 174 | 260 | 679 | 543 | 809 | 412 | 319 | 489 |
| 12774753 | PLA2R1 | phospholipase A2 receptor 1, 180kDa (PLA2R1), mRNA. | 4.814234981 | 23 | 21 | 24 | 18 | 232 | 196 | 204 | 331 | 342 | 285 | 679 | 553 | 774 |
| 12825859 | PLAU | plasminogen activator, urokinase (PLAU), mRNA. | 5.338766876 | 44 | 33 | 43 | 46 | 58 | 68 | 91 | 454 | 410 | 394 | 185 | 187 | 119 |
| 12755776 | PLAUR | plasminogen activator, urokinase receptor (PLAUR), mRNA. | 3.467865209 | 42 | 33 | 42 | 27 | 37 | 41 | 55 | 170 | 136 | 109 | 119 | 119 | 170 |
| 12746590 | PLBD2 | phospholipase B domain containing 2 (PLBD2), mRNA. | 3.159542413 | 479 | 414 | 491 | 318 | 703 | 512 | 483 | 1366 | 1385 | 1240 | 1834 | 1608 | 1774 |
| 12778337 | PLCL1 | phospholipase C-like 1 (PLCL1), mRNA. | 9.308109696 | 15 | 16 | 16 | 16 | 30 | 38 | 37 | 295 | 225 | 353 | 163 | 128 | 172 |
| 12889041 | PLIN2 | Perilipin 2 | 4.161598852 | 584 | 551 | 592 | 400 | 523 | 512 | 557 | 1704 | 1806 | 1775 | 2478 | 2758 | 2744 |
| 12786260 | PLK2 | polo-like kinase 2 (PLK2), mRNA. | 8.45988845 | 21 | 26 | 24 | 29 | 104 | 266 | 175 | 1009 | 888 | 845 | 633 | 755 | 547 |
| 12843108 | PLK3 | polo-like kinase 3 (PLK3), mRNA. | 5.015528028 | 39 | 40 | 35 | 33 | 61 | 61 | 57 | 311 | 323 | 340 | 137 | 140 | 152 |
| 12807296 | PMAIP1 | PREDICTED: phorbol-12-myristate-13-acetate-induced protein 1 (PMAIP1), mRNA. | 8.718063935 | 15 | 12 | 14 | 14 | 16 | 16 | 19 | 122 | 217 | 154 | 106 | 88 | 109 |
| 12812212 | PMM2 | phosphomannomutase 2 (PMM2), mRNA. | 2.05408052 | 173 | 191 | 170 | 169 | 156 | 145 | 139 | 314 | 344 | 411 | 311 | 278 | 354 |
| 12838199 | PMVK | Phosphomevalonate Kinase | 2.449982957 | 82 | 57 | 48 | 45 | 98 | 90 | 82 | 153 | 168 | 170 | 199 | 148 | 218 |
| 12779334 | PNKD | paroxysmal nonkinesigenic dyskinesia (PNKD), nuclear gene encoding mitochondrial protein, transcript variant 1, mRNA. | 2.176105075 | 104 | 107 | 109 | 73 | 90 | 77 | 82 | 233 | 226 | 315 | 159 | 128 | 139 |
| 12693791 | PNP | purine nucleoside phosphorylase (PNP), mRNA. | 3.193528779 | 78 | 63 | 54 | 50 | 76 | 111 | 87 | 285 | 268 | 306 | 164 | 174 | 219 |
| 12769861 | PNPO | Pyridoxamine 5'-Phosphate Oxidase | 2.769057534 | 49 | 49 | 57 | 55 | 120 | 114 | 147 | 154 | 239 | 235 | 281 | 207 | 288 |
| 12899887 | PNRC1 | proline-rich nuclear receptor coactivator 1 (PNRC1), mRNA. | 3.838441986 | 581 | 570 | 686 | 616 | 567 | 424 | 339 | 1953 | 1785 | 2040 | 2174 | 2289 | 2204 |
| 12855916 | PODXL | Podocalyxin-Like | 7.580140653 | 45 | 30 | 32 | 28 | 80 | 145 | 128 | 846 | 869 | 733 | 211 | 304 | 214 |
| 12768134 | POLG2 | polymerase (DNA directed), gamma 2, accessory subunit (POLG2), nuclear gene encoding mitochondrial protein, mRNA. | 3.175712466 | 117 | 144 | 133 | 133 | 74 | 87 | 79 | 243 | 378 | 473 | 201 | 320 | 478 |
| 12883718 | PPAP2C | phosphatidic acid phosphatase type 2C (PPAP2C), mRNA. | 3.268281448 | 20 | 19 | 32 | 22 | 37 | 29 | 26 | 70 | 65 | 66 | 98 | 101 | 114 |
| 12801263 | PPARD | peroxisome proliferator-activated receptor delta (PPARD), mRNA. | 3.324994032 | 180 | 172 | 228 | 126 | 171 | 183 | 195 | 582 | 559 | 569 | 636 | 623 | 608 |
| 12792239 | PPIP5K1 | diphosphoinositol pentakisphosphate kinase 1 (PPIP5K1), mRNA. | 3.269334354 | 76 | 86 | 78 | 61 | 101 | 63 | 84 | 280 | 280 | 276 | 248 | 177 | 279 |
| 12752595 | PPM1N | protein phosphatase, Mg2+/Mn2+ dependent, 1N (putative) (PPM1N), mRNA. | 3.602751197 | 31 | 27 | 30 | 33 | 54 | 45 | 50 | 106 | 144 | 129 | 167 | 154 | 137 |
| 12751650 | PPP1R15A | protein phosphatase 1, regulatory subunit 15A (PPP1R15A), mRNA. | 18.0311681 | 116 | 91 | 115 | 73 | 81 | 69 | 78 | 1504 | 1511 | 1517 | 1729 | 1835 | 1526 |
| 12687633 | PPP1R36 | Protein Phosphatase 1, Regulatory Subunit 36 | 4.187976834 | 51 | 73 | 65 | 104 | 36 | 44 | 37 | 214 | 231 | 242 | 246 | 217 | 321 |
| 12825144 | PPP1R3B | Protein Phosphatase 1, Regulatory Subunit 3B | 2.712972896 | 81 | 94 | 67 | 62 | 63 | 71 | 66 | 170 | 145 | 161 | 195 | 226 | 274 |
| 12870296 | PPP3CA | Protein Phosphatase 3, Catalytic Subunit, Alpha Isozyme | 2.482470967 | 160 | 171 | 147 | 142 | 174 | 210 | 180 | 440 | 465 | 449 | 371 | 368 | 428 |
| 12901108 | PRDM1 | PR domain containing 1, with ZNF domain (PRDM1), mRNA. | 21.83501578 | 25 | 21 | 25 | 15 | 37 | 41 | 36 | 609 | 608 | 601 | 592 | 760 | 608 |
| 12785083 | PRLR | Prolactin Receptor | 38.78926985 | 15 | 13 | 17 | 14 | 29 | 28 | 19 | 1735 | 860 | 1692 | 73 | 114 | 42 |
| 12713603 | PRNP | prion protein (PRNP), mRNA. | 3.084513027 | 81 | 90 | 109 | 72 | 143 | 108 | 105 | 324 | 323 | 326 | 320 | 304 | 277 |
| 12713731 | PROCR | Protein C Receptor, Endothelial | 3.655965632 | 25 | 38 | 27 | 26 | 37 | 41 | 35 | 141 | 183 | 129 | 92 | 103 | 65 |
| 12727012 | PRR5L | proline rich 5 like (PRR5L), mRNA. | 4.170102323 | 27 | 21 | 21 | 22 | 22 | 29 | 24 | 95 | 82 | 119 | 100 | 45 | 156 |
| 12737194 | PRRX1 | paired related homeobox 1 (PRRX1), mRNA. | 4.558890111 | 44 | 38 | 66 | 31 | 71 | 59 | 119 | 197 | 347 | 178 | 308 | 252 | 392 |
| 12893989 | PRUNE2 | prune homolog 2 (Drosophila), mRNA (cDNA clone MGC:166262 IMAGE:8214184), complete cds. | 8.240242637 | 52 | 49 | 73 | 49 | 49 | 110 | 59 | 330 | 587 | 387 | 510 | 480 | 824 |
| 12827687 | PSAP | prosaposin (PSAP), mRNA. | 2.54560888 | 731 | 758 | 1200 | 695 | 1236 | 1278 | 1260 | 2123 | 2411 | 1835 | 2900 | 3340 | 3015 |
| 12809935 | PSTPIP2 | proline-serine-threonine phosphatase interacting protein 2 (PSTPIP2), mRNA. | 3.469042564 | 28 | 37 | 33 | 20 | 75 | 66 | 67 | 115 | 107 | 95 | 250 | 211 | 190 |
| 12842705 | PTGFR | prostaglandin F receptor (FP) (PTGFR), mRNA. | 10.77818191 | 457 | 469 | 211 | 607 | 281 | 155 | 307 | 6932 | 5607 | 7278 | 1199 | 735 | 1235 |
| 12718058 | PTGIS | Prostaglandin I2 (Prostacyclin) Synthase | 10.3329112 | 44 | 36 | 50 | 37 | 225 | 315 | 185 | 1001 | 937 | 438 | 2065 | 2002 | 1461 |
| 12723906 | PTP4A3 | protein tyrosine phosphatase type IVA, member 3 (PTP4A3), mRNA. | 5.213894763 | 34 | 25 | 35 | 29 | 73 | 97 | 85 | 478 | 392 | 378 | 170 | 161 | 110 |
| 12727236 | PTPRJ | Protein Tyrosine Phosphatase, Receptor Type, J | 2.535273247 | 84 | 72 | 101 | 74 | 302 | 283 | 300 | 445 | 468 | 428 | 439 | 446 | 415 |
| 12808262 | PTPRM | Protein Tyrosine Phosphatase, Receptor Type, M | 2.134146273 | 372 | 404 | 501 | 269 | 325 | 363 | 321 | 981 | 925 | 856 | 664 | 612 | 634 |
| 12780430 | PTPRN | protein tyrosine phosphatase, receptor type, N (PTPRN), mRNA. | 6.159131373 | 27 | 22 | 25 | 25 | 23 | 23 | 26 | 52 | 66 | 64 | 143 | 335 | 246 |
| 12686574 | PTX3 | pentraxin 3, long (PTX3), mRNA. | 39.85247383 | 33 | 23 | 30 | 28 | 27 | 41 | 28 | 557 | 968 | 695 | 1448 | 1579 | 1942 |
| 12752457 | PVR | PREDICTED: poliovirus receptor (PVR), mRNA. | 3.973444416 | 70 | 53 | 81 | 57 | 114 | 133 | 131 | 495 | 506 | 523 | 210 | 213 | 230 |
| 12800376 | PXK | PX domain containing serine/threonine kinase (PXK), mRNA. | 3.295438522 | 93 | 123 | 120 | 113 | 179 | 209 | 195 | 360 | 426 | 383 | 638 | 530 | 577 |
| 12693905 | PYGL | phosphorylase, glycogen, liver (PYGL), mRNA. | 2.191481969 | 814 | 841 | 1407 | 906 | 1174 | 1129 | 1207 | 2762 | 2210 | 2648 | 2316 | 1936 | 2176 |
| 12818183 | QPRT | quinolinate phosphoribosyltransferase (QPRT), mRNA. | 4.434236918 | 94 | 87 | 72 | 78 | 145 | 141 | 138 | 508 | 388 | 626 | 522 | 288 | 540 |
| 12692187 | RAB27A | RAB27A, member RAS oncogene family (RAB27A), mRNA. | 6.778311866 | 94 | 85 | 76 | 86 | 182 | 144 | 237 | 771 | 852 | 743 | 1141 | 797 | 953 |
| 12903629 | RAB33A | RAB33A, member RAS oncogene family (RAB33A), mRNA. | 2.066889958 | 82 | 64 | 60 | 57 | 55 | 52 | 43 | 86 | 102 | 114 | 183 | 94 | 156 |
| 12705580 | RALGDS | ral guanine nucleotide dissociation stimulator (RALGDS), mRNA. | 2.606153298 | 79 | 69 | 79 | 59 | 74 | 67 | 70 | 263 | 261 | 221 | 126 | 118 | 117 |
| 12761434 | RAMP2 | receptor (G protein-coupled) activity modifying protein 2 (RAMP2), mRNA. | 4.741917405 | 38 | 37 | 26 | 33 | 215 | 414 | 275 | 904 | 1019 | 787 | 410 | 607 | 490 |
| 12855665 | RAMP3 | receptor (G protein-coupled) activity modifying protein 3 (RAMP3), mRNA. | 6.576633857 | 27 | 37 | 28 | 37 | 61 | 79 | 77 | 472 | 516 | 393 | 199 | 238 | 130 |
| 12784836 | RANBP3L | RAN Binding Protein 3-Like | 44.75507631 | 8 | 7 | 6 | 7 | 6 | 7 | 8 | 151 | 397 | 299 | 293 | 147 | 581 |
| 12854454 | RAPGEF5 | PREDICTED: Rap guanine nucleotide exchange factor (GEF) 5 (RAPGEF5), mRNA. | 4.524922533 | 21 | 15 | 22 | 18 | 31 | 50 | 37 | 199 | 213 | 142 | 71 | 75 | 52 |
| 12737168 | RASAL2 | PREDICTED: RAS protein activator like 2 (RASAL2), mRNA. | 2.26729699 | 119 | 92 | 149 | 132 | 81 | 139 | 127 | 353 | 375 | 339 | 160 | 217 | 188 |
| 12879860 | RASGRF2 | PREDICTED: Ras protein-specific guanine nucleotide-releasing factor 2 (RASGRF2), partial mRNA. | 8.401456833 | 26 | 30 | 44 | 55 | 35 | 56 | 53 | 312 | 413 | 261 | 675 | 276 | 218 |
| 12756319 | RASIP1 | Ras interacting protein 1 (RASIP1), mRNA. | 4.401237769 | 94 | 74 | 68 | 82 | 118 | 146 | 114 | 530 | 636 | 533 | 307 | 309 | 306 |
| 12867519 | RASSF3 | Ras Association (RalGDS/AF-6) Domain Family Member 3 | 2.575401139 | 32 | 37 | 37 | 37 | 90 | 62 | 63 | 154 | 159 | 160 | 115 | 98 | 104 |
| 12699819 | RBKS | ribokinase (RBKS), mRNA. | 3.354972397 | 155 | 158 | 144 | 150 | 91 | 76 | 96 | 575 | 464 | 674 | 322 | 200 | 271 |
| 12821410 | RBP4 | retinol binding protein 4, plasma (RBP4), mRNA. | 2.014055612 | 98 | 93 | 129 | 89 | 53 | 79 | 68 | 188 | 153 | 194 | 162 | 159 | 197 |
| 12824460 | RBPMS | RNA binding protein with multiple splicing (RBPMS), mRNA. | 5.318390818 | 21 | 16 | 19 | 17 | 154 | 204 | 105 | 312 | 342 | 318 | 388 | 627 | 454 |
| 12681965 | RCAN1 | regulator of calcineurin 1 | 9.867862894 | 69 | 72 | 88 | 90 | 109 | 80 | 109 | 532 | 720 | 572 | 1020 | 1044 | 1334 |
| 12699543 | REL | v-rel reticuloendotheliosis viral oncogene homolog (avian) (REL), mRNA. | 4.144453532 | 16 | 17 | 26 | 19 | 40 | 56 | 46 | 160 | 194 | 178 | 68 | 84 | 98 |
| 12752266 | RELB | V-Rel Avian Reticuloendotheliosis Viral Oncogene Homolog B | 3.530463237 | 45 | 48 | 50 | 40 | 49 | 46 | 49 | 134 | 144 | 156 | 126 | 200 | 232 |
| 12853004 | RELN | reelin (RELN), mRNA. | 7.825308804 | 19 | 18 | 24 | 15 | 65 | 92 | 27 | 335 | 499 | 243 | 194 | 318 | 161 |
| 12702548 | RETSAT | retinol saturase (all-trans-retinol 13,14-reductase) (RETSAT), mRNA. | 2.311801166 | 104 | 113 | 153 | 113 | 309 | 302 | 285 | 456 | 496 | 510 | 494 | 377 | 398 |
| 12729629 | REXO2 | REX2, RNA exonuclease 2 homolog (S. cerevisiae) (REXO2), nuclear gene encoding mitochondrial protein, mRNA. | 2.23189892 | 366 | 431 | 307 | 451 | 796 | 484 | 804 | 1138 | 1101 | 1065 | 1458 | 1037 | 1163 |
| 12738588 | RGS16 | regulator of G-protein signaling 16 (RGS16), mRNA. | 16.09309962 | 36 | 25 | 25 | 24 | 32 | 35 | 40 | 261 | 408 | 239 | 653 | 874 | 571 |
| 12739391 | RGS2 | Regulator Of G-Protein Signaling 2 | 5.974432156 | 27 | 37 | 42 | 33 | 70 | 57 | 79 | 297 | 464 | 276 | 260 | 276 | 198 |
| 12705763 | RHOB | ras homolog gene family, member B (RHOB), mRNA. | 4.683798576 | 336 | 312 | 335 | 291 | 394 | 253 | 280 | 1428 | 1404 | 1208 | 1445 | 1805 | 1545 |
| 12691467 | RHOJ | ras homolog gene family, member J (RHOJ), mRNA. | 5.140378085 | 31 | 32 | 25 | 36 | 137 | 206 | 160 | 675 | 718 | 547 | 239 | 378 | 207 |
| 12697991 | RHOQ | ras homolog gene family, member Q (RHOQ), mRNA. | 2.587142019 | 223 | 267 | 371 | 341 | 464 | 358 | 416 | 1086 | 882 | 1170 | 764 | 730 | 780 |
| 12773396 | RILP | PREDICTED: Rab interacting lysosomal protein (RILP), mRNA. | 2.405768488 | 49 | 41 | 44 | 31 | 50 | 58 | 57 | 117 | 125 | 111 | 124 | 82 | 119 |
| 12809919 | RIOK3 | RIO kinase 3 (yeast) (RIOK3), mRNA. | 2.809929989 | 406 | 446 | 420 | 524 | 519 | 499 | 481 | 1119 | 1258 | 1339 | 1362 | 1347 | 1511 |
| 12697204 | RNASE4 | ribonuclease, RNase A family, 4 (RNASE4), mRNA. | 20.43604117 | 25 | 18 | 61 | 38 | 130 | 265 | 247 | 3284 | 3126 | 5275 | 997 | 304 | 745 |
| 12899892 | RNASET2 | ribonuclease T2 (RNASET2), mRNA. | 3.067888308 | 169 | 150 | 145 | 122 | 183 | 152 | 170 | 494 | 471 | 427 | 527 | 536 | 411 |
| 12858617 | RND1 | Rho family GTPase 1 (RND1), mRNA. | 32.08581682 | 38 | 29 | 35 | 28 | 37 | 36 | 29 | 897 | 1230 | 1222 | 776 | 1143 | 1080 |
| 12778611 | RND3 | Rho family GTPase 3 (RND3), mRNA. | 15.84010886 | 34 | 22 | 42 | 31 | 169 | 195 | 192 | 1195 | 1282 | 1245 | 1650 | 2110 | 1817 |
| 12825057 | RNF122 | ring finger protein 122 (RNF122), mRNA. | 6.236989492 | 52 | 57 | 88 | 56 | 52 | 48 | 58 | 328 | 351 | 411 | 331 | 466 | 298 |
| 12809835 | RNF125 | ring finger protein 125 (RNF125), mRNA. | 2.752290329 | 40 | 53 | 79 | 64 | 78 | 92 | 80 | 238 | 224 | 189 | 162 | 186 | 148 |
| 12909463 | RNF128 | Ring Finger Protein 128, E3 Ubiquitin Protein Ligase | 3.123435295 | 515 | 498 | 717 | 659 | 675 | 492 | 985 | 2290 | 2360 | 2040 | 2176 | 1377 | 1919 |
| 12744445 | RNF150 | PREDICTED: ring finger protein 150 (RNF150), mRNA. | 8.525324617 | 13 | 18 | 28 | 17 | 132 | 140 | 78 | 270 | 426 | 260 | 551 | 749 | 863 |
| 12775981 | RNF19B | ring finger protein 19B (RNF19B), mRNA. | 4.493258806 | 60 | 54 | 57 | 55 | 55 | 52 | 57 | 253 | 292 | 288 | 182 | 238 | 249 |
| 12759295 | RRAS | related RAS viral (r-ras) oncogene homolog (RRAS), mRNA. | 3.637040142 | 109 | 113 | 133 | 116 | 160 | 167 | 142 | 507 | 495 | 451 | 577 | 458 | 441 |
| 12803139 | RUNX2 | PREDICTED: runt-related transcription factor 2, transcript variant 1 (RUNX2), mRNA. | 18.34665494 | 37 | 24 | 44 | 25 | 47 | 38 | 40 | 689 | 587 | 750 | 542 | 550 | 922 |
| 12889697 | RUSC2 | RUN and SH3 domain containing 2 (RUSC2), mRNA. | 2.374470905 | 46 | 39 | 54 | 49 | 85 | 105 | 103 | 133 | 178 | 158 | 134 | 192 | 185 |
| 12748645 | RYR1 | ryanodine receptor 1 (skeletal) (RYR1), mRNA. | 3.190302552 | 29 | 29 | 26 | 23 | 30 | 32 | 33 | 54 | 66 | 60 | 114 | 90 | 169 |
| 12841505 | S100A14 | S100 calcium binding protein A14 (S100A14), mRNA. | 2.475830987 | 32 | 32 | 29 | 26 | 49 | 50 | 49 | 81 | 98 | 65 | 138 | 84 | 99 |
| 12836716 | S100A16 | S100 calcium binding protein A16 (S100A16), mRNA. | 2.035991592 | 103 | 146 | 91 | 137 | 207 | 182 | 166 | 288 | 322 | 252 | 390 | 302 | 248 |
| 12842602 | S1PR1 | sphingosine-1-phosphate receptor 1 (S1PR1), mRNA. | 4.881260753 | 35 | 20 | 32 | 26 | 103 | 170 | 122 | 513 | 525 | 467 | 200 | 254 | 163 |
| 12898520 | SASH1 | PREDICTED: SAM and SH3 domain containing 1 (SASH1), mRNA. | 3.324540179 | 87 | 82 | 51 | 74 | 99 | 120 | 106 | 326 | 403 | 315 | 206 | 208 | 308 |
| 12907723 | SAT1 | spermidine/spermine N1-acetyltransferase 1 (SAT1), mRNA. | 6.682643203 | 215 | 215 | 312 | 264 | 1017 | 878 | 821 | 3411 | 3720 | 3714 | 3251 | 3331 | 3892 |
| 12885092 | SBNO2 | strawberry notch homolog 2 (Drosophila) (SBNO2), mRNA. | 2.221737948 | 132 | 124 | 137 | 112 | 95 | 91 | 97 | 204 | 269 | 259 | 195 | 249 | 327 |
| 12728499 | SC5DL | sterol-C5-desaturase (ERG3 delta-5-desaturase homolog, S. cerevisiae)-like (SC5DL), mRNA. | 2.260579582 | 1100 | 1072 | 881 | 928 | 931 | 1094 | 1086 | 1963 | 1995 | 2368 | 2503 | 2252 | 2662 |
| 12794901 | SCAP | SREBF chaperone (SCAP), mRNA. | 2.444962108 | 571 | 544 | 429 | 476 | 641 | 526 | 355 | 1315 | 1280 | 1488 | 1145 | 1132 | 1064 |
| 12772913 | SCARF1 | Scavenger Receptor Class F, Member 1 | 2.508980508 | 48 | 48 | 44 | 38 | 56 | 83 | 72 | 164 | 154 | 139 | 118 | 122 | 140 |
| 12754130 | SCN1B | sodium channel, voltage-gated, type I, beta subunit (SCN1B), mRNA. | 3.690645576 | 44 | 31 | 36 | 39 | 66 | 71 | 52 | 179 | 236 | 159 | 158 | 197 | 146 |
| 12718768 | SDC4 | Syndecan 4 | 18.75267722 | 97 | 123 | 108 | 93 | 124 | 82 | 51 | 1458 | 1433 | 1834 | 1786 | 1871 | 2502 |
| 12837582 | SELENBP1 | selenium binding protein 1 (SELENBP1), mRNA. | 2.745602117 | 35 | 27 | 30 | 22 | 54 | 55 | 51 | 96 | 85 | 72 | 163 | 104 | 127 |
| 12738559 | SELP | Selectin P (Granule Membrane Protein 140kDa, Antigen CD62) | 16.63839334 | 26 | 21 | 27 | 17 | 37 | 65 | 60 | 933 | 1015 | 1043 | 210 | 189 | 223 |
| 12851272 | SEMA3D | sema domain, immunoglobulin domain (Ig), short basic domain, secreted, (semaphorin) 3D (SEMA3D), mRNA. | 9.873188405 | 12 | 10 | 12 | 13 | 52 | 57 | 69 | 304 | 318 | 369 | 291 | 214 | 397 |
| 12799597 | SEMA3F | PREDICTED: sema domain, immunoglobulin domain (Ig), short basic domain, secreted, (semaphorin) 3F (SEMA3F), mRNA. | 5.186775779 | 46 | 33 | 39 | 34 | 79 | 110 | 79 | 420 | 463 | 366 | 193 | 230 | 195 |
| 12880155 | SEMA6B | PREDICTED: sema domain, transmembrane domain (TM), and cytoplasmic domain, (semaphorin) 6B (SEMA6B), mRNA. | 3.439472138 | 63 | 67 | 66 | 48 | 60 | 67 | 67 | 192 | 229 | 180 | 198 | 219 | 272 |
| 12708624 | SERTAD2 | PREDICTED: SERTA domain containing 2 (SERTAD2), mRNA. | 2.875721127 | 66 | 66 | 74 | 46 | 50 | 53 | 48 | 173 | 200 | 205 | 132 | 161 | 123 |
| 12742746 | SETD7 | KIAA1717-like protein (SETD7-1) mRNA, partial cds. | 3.065489201 | 197 | 196 | 152 | 204 | 271 | 273 | 251 | 598 | 633 | 526 | 791 | 778 | 732 |
| 12715964 | SFMBT2 | Scm-like with four mbt domains 2 (SFMBT2), mRNA. | 3.467339225 | 27 | 22 | 29 | 32 | 47 | 49 | 49 | 106 | 116 | 107 | 122 | 150 | 156 |
| 12900295 | SGK1 | serum/glucocorticoid regulated kinase 1 (SGK1), mRNA. | 12.45279863 | 37 | 37 | 76 | 20 | 94 | 195 | 169 | 1485 | 1392 | 1812 | 675 | 660 | 679 |
| 12903638 | SH3KBP1 | SH3-domain kinase binding protein 1 (SH3KBP1), mRNA. | 4.915102909 | 76 | 63 | 91 | 54 | 122 | 152 | 113 | 463 | 529 | 632 | 308 | 273 | 621 |
| 12787557 | SH3PXD2B | PREDICTED: SH3 and PX domains 2B (SH3PXD2B), mRNA. | 3.911651908 | 58 | 65 | 58 | 38 | 116 | 147 | 125 | 309 | 396 | 257 | 428 | 290 | 357 |
| 12822921 | SLC16A12 | Solute Carrier Family 16, Member 12 | 11.08011015 | 122 | 115 | 80 | 117 | 29 | 20 | 36 | 1100 | 912 | 1083 | 834 | 459 | 549 |
| 12783622 | SLC16A14 | solute carrier family 16, member 14 (monocarboxylic acid transporter 14) (SLC16A14), mRNA. | 2.506706261 | 77 | 93 | 126 | 181 | 174 | 149 | 129 | 313 | 429 | 315 | 333 | 215 | 392 |
| 12740186 | SLC19A2 | solute carrier family 19 (thiamine transporter), member 2 (SLC19A2), mRNA. | 2.488391324 | 54 | 50 | 59 | 59 | 82 | 85 | 104 | 131 | 129 | 175 | 163 | 238 | 216 |
| 12699945 | SLC20A1 | solute carrier family 20 (phosphate transporter), member 1 (SLC20A1), mRNA. | 2.885356084 | 438 | 424 | 429 | 415 | 329 | 249 | 369 | 1196 | 1185 | 1519 | 912 | 748 | 1004 |
| 12825182 | SLC20A2 | Solute Carrier Family 20 (Phosphate Transporter), Member 2 | 2.877899355 | 82 | 70 | 82 | 61 | 83 | 94 | 80 | 311 | 286 | 267 | 157 | 154 | 185 |
| 12721219 | SLC23A2 | solute carrier family 23 (nucleobase transporters), member 2 (SLC23A2), mRNA. | 3.463647113 | 202 | 197 | 234 | 215 | 329 | 339 | 360 | 765 | 822 | 798 | 1117 | 860 | 1209 |
| 12701090 | SLC25A25 | Solute Carrier Family 25 (Mitochondrial Carrier; Phosphate Carrier), Member 25 | 4.409052112 | 105 | 96 | 83 | 67 | 77 | 67 | 87 | 383 | 302 | 331 | 430 | 385 | 365 |
| 12771254 | SLC25A35 | solute carrier family 25, member 35 (SLC25A35), mRNA. | 4.028686449 | 41 | 26 | 41 | 28 | 79 | 57 | 86 | 184 | 131 | 167 | 318 | 123 | 311 |
| 12823572 | SLC25A4 | solute carrier family 25 (mitochondrial carrier; adenine nucleotide translocator), member 4 (SLC25A4), nuclear gene encoding mitochondrial protein, mRNA. | 2.261532385 | 140 | 157 | 152 | 208 | 131 | 106 | 122 | 291 | 244 | 418 | 315 | 226 | 475 |
| 12905068 | SLC25A43 | Solute Carrier Family 25, Member 43 | 2.37669577 | 46 | 51 | 60 | 58 | 63 | 66 | 72 | 182 | 144 | 135 | 160 | 98 | 128 |
| 12836031 | SLC2A1 | solute carrier family 2 (facilitated glucose transporter), member 1 (SLC2A1), mRNA. | 4.878958772 | 289 | 280 | 395 | 269 | 233 | 276 | 218 | 1314 | 1125 | 1581 | 1453 | 1323 | 1404 |
| 12864151 | SLC2A3 | solute carrier family 2 (facilitated glucose transporter), member 3 (SLC2A3), mRNA. | 5.524684266 | 100 | 109 | 189 | 201 | 53 | 59 | 48 | 801 | 633 | 1025 | 326 | 471 | 338 |
| 12873040 | SLC2A9 | PREDICTED: solute carrier family 2 (facilitated glucose transporter), member 9 (SLC2A9), mRNA. | 4.661844487 | 45 | 52 | 48 | 45 | 122 | 133 | 111 | 308 | 458 | 402 | 346 | 236 | 477 |
| 12890767 | SLC31A2 | solute carrier family 31 (copper transporters), member 2 (SLC31A2), mRNA. | 2.598860258 | 63 | 50 | 47 | 38 | 113 | 109 | 93 | 181 | 140 | 154 | 235 | 240 | 193 |
| 12831879 | SLC36A4 | PREDICTED: solute carrier family 36 (proton/amino acid symporter), member 4 (SLC36A4), mRNA. | 2.118498519 | 67 | 101 | 89 | 94 | 79 | 106 | 95 | 218 | 174 | 187 | 197 | 204 | 164 |
| 12829973 | SLC3A2 | solute carrier family 3 (activators of dibasic and neutral amino acid transport), member 2 (SLC3A2), mRNA. | 2.188410811 | 472 | 454 | 486 | 479 | 390 | 415 | 343 | 881 | 1010 | 974 | 989 | 811 | 1034 |
| 12732213 | SLC43A3 | solute carrier family 43, member 3 (SLC43A3), mRNA. | 3.357371905 | 56 | 56 | 67 | 46 | 70 | 90 | 92 | 183 | 232 | 143 | 290 | 258 | 267 |
| 12710773 | SLC46A3 | solute carrier family 46, member 3 (SLC46A3), mRNA. | 5.194196768 | 58 | 50 | 53 | 67 | 57 | 42 | 40 | 220 | 218 | 174 | 307 | 329 | 385 |
| 12798047 | SLC6A6 | solute carrier family 6 (neurotransmitter transporter, taurine), member 6 (SLC6A6), mRNA. | 4.634478215 | 42 | 54 | 50 | 36 | 95 | 117 | 102 | 403 | 387 | 399 | 253 | 221 | 307 |
| 12902939 | SLC6A8 | solute carrier family 6 (neurotransmitter transporter, creatine), member 8 (SLC6A8), mRNA. | 3.408230147 | 211 | 176 | 193 | 155 | 201 | 161 | 156 | 605 | 734 | 775 | 549 | 470 | 525 |
| 12774671 | SLC9A1 | solute carrier family 9 (sodium/hydrogen exchanger), member 1 (SLC9A1), mRNA. | 3.002459118 | 138 | 147 | 157 | 90 | 142 | 152 | 132 | 497 | 499 | 411 | 421 | 307 | 331 |
| 12681617 | SLC9A9 | Solute Carrier Family 9, Subfamily A (NHE9, Cation Proton Antiporter 9), Member 9 | 6.63412638 | 16 | 24 | 38 | 18 | 73 | 67 | 70 | 233 | 263 | 303 | 324 | 280 | 343 |
| 12857240 | SLCO1A2 | Solute Carrier Organic Anion Transporter Family, Member 1A2 | 5.342510919 | 9 | 11 | 11 | 18 | 32 | 13 | 33 | 52 | 87 | 92 | 74 | 74 | 198 |
| 12678807 | SLCO2A1 | solute carrier organic anion transporter family, member 2A1 | 6.512077123 | 37 | 47 | 45 | 35 | 352 | 748 | 462 | 2404 | 2476 | 1687 | 953 | 1391 | 727 |
| 12718754 | SLCO4A1 | solute carrier organic anion transporter family, member 4A1 (SLCO4A1), mRNA. | 5.819476847 | 42 | 39 | 36 | 33 | 30 | 34 | 34 | 217 | 169 | 293 | 146 | 111 | 300 |
| 12810353 | SMAD7 | SMAD family member 7 (SMAD7), mRNA. | 6.285138071 | 43 | 37 | 39 | 28 | 40 | 38 | 40 | 254 | 203 | 201 | 313 | 254 | 204 |
| 12714026 | SNAI1 | snail homolog 1 (Drosophila) (SNAI1), mRNA. | 3.846930929 | 28 | 22 | 25 | 24 | 36 | 31 | 30 | 165 | 168 | 115 | 63 | 83 | 49 |
| 12796510 | SNRK | PREDICTED: SNF related kinase (SNRK), mRNA. | 2.996072498 | 121 | 112 | 120 | 139 | 152 | 229 | 229 | 748 | 589 | 594 | 297 | 368 | 238 |
| 12788045 | SNX18 | sorting nexin 18 (SNX18), mRNA. | 2.946886175 | 201 | 190 | 221 | 196 | 162 | 187 | 175 | 365 | 456 | 447 | 591 | 836 | 668 |
| 12817048 | SOCS1 | PREDICTED: suppressor of cytokine signaling 1 (SOCS1), mRNA. | 2.783692059 | 39 | 30 | 35 | 34 | 52 | 55 | 55 | 81 | 115 | 69 | 118 | 187 | 147 |
| 12857394 | SOCS2 | suppressor of cytokine signaling 2 (SOCS2), mRNA. | 2.708943292 | 65 | 58 | 46 | 40 | 42 | 53 | 63 | 226 | 126 | 169 | 92 | 84 | 154 |
| 12760916 | SOCS3 | suppressor of cytokine signaling 3 (SOCS3), mRNA. | 19.13972944 | 86 | 79 | 54 | 58 | 59 | 66 | 56 | 1028 | 1058 | 1008 | 1276 | 1676 | 1448 |
| 12871557 | SOD3 | superoxide dismutase 3, extracellular (SOD3), mRNA. | 5.046049331 | 71 | 47 | 78 | 44 | 108 | 137 | 130 | 441 | 326 | 396 | 347 | 782 | 369 |
| 12715424 | SOX18 | SRY (sex determining region Y)-box 18 (SOX18), mRNA. | 4.520465642 | 42 | 29 | 40 | 36 | 57 | 74 | 73 | 271 | 459 | 238 | 155 | 129 | 104 |
| 12769620 | SPATA20 | spermatogenesis associated 20 (SPATA20), mRNA. | 2.645177486 | 33 | 24 | 52 | 53 | 68 | 97 | 69 | 158 | 130 | 172 | 161 | 152 | 125 |
| 12873212 | SPP1 | Secreted Phosphoprotein 1 | 8.707173931 | 89 | 86 | 311 | 24 | 35 | 57 | 79 | 870 | 1471 | 316 | 1488 | 468 | 468 |
| 12689764 | SPRED1 | sprouty-related, EVH1 domain containing 1 (SPRED1), mRNA. | 2.152692731 | 170 | 170 | 161 | 232 | 257 | 360 | 327 | 567 | 608 | 498 | 477 | 491 | 452 |
| 12706080 | SPRED2 | Sprouty-Related, EVH1 Domain Containing 2 | 2.239780903 | 99 | 88 | 87 | 99 | 88 | 127 | 104 | 211 | 222 | 196 | 224 | 256 | 218 |
| 12745981 | SPRY1 | sprouty homolog 1, antagonist of FGF signaling (Drosophila) (SPRY1), mRNA. | 3.785260199 | 51 | 85 | 87 | 140 | 136 | 253 | 248 | 635 | 617 | 471 | 485 | 529 | 511 |
| 12883735 | SPRY4 | sprouty homolog 4 (Drosophila) (SPRY4), mRNA. | 3.883889892 | 32 | 32 | 27 | 23 | 37 | 47 | 46 | 193 | 161 | 177 | 135 | 78 | 67 |
| 12738745 | SPSB1 | SplA/Ryanodine Receptor Domain And SOCS Box Containing 1 | 3.479208345 | 513 | 540 | 398 | 282 | 593 | 343 | 250 | 1019 | 1018 | 1012 | 1762 | 1888 | 2006 |
| 12694111 | SQRDL | sulfide quinone reductase-like (yeast) (SQRDL), nuclear gene encoding mitochondrial protein, mRNA. | 5.242038624 | 224 | 243 | 135 | 150 | 646 | 475 | 450 | 1903 | 1736 | 2175 | 1576 | 1554 | 1494 |
| 12883433 | SQSTM1 | sequestosome 1 (SQSTM1), mRNA. | 2.514077714 | 362 | 407 | 700 | 408 | 709 | 526 | 546 | 1281 | 1250 | 1569 | 1084 | 1303 | 1393 |
| 12788328 | SRD5A1 | steroid-5-alpha-reductase, alpha polypeptide 1 (3-oxo-5 alpha-steroid delta 4-dehydrogenase alpha 1) (SRD5A1), mRNA. | 2.267929571 | 58 | 50 | 58 | 58 | 66 | 56 | 49 | 122 | 99 | 82 | 161 | 186 | 117 |
| 12716125 | SRXN1 | sulfiredoxin 1 (SRXN1), mRNA. | 6.631965784 | 36 | 39 | 43 | 28 | 67 | 38 | 68 | 338 | 298 | 365 | 294 | 197 | 323 |
| 12884325 | SSBP4 | single stranded DNA binding protein 4 (SSBP4), mRNA. | 2.319346882 | 61 | 47 | 62 | 35 | 79 | 79 | 73 | 168 | 134 | 120 | 156 | 148 | 141 |
| 12783574 | SSFA2 | sperm specific antigen 2 (SSFA2), mRNA. | 2.106894985 | 131 | 135 | 124 | 137 | 116 | 151 | 146 | 329 | 363 | 336 | 224 | 218 | 225 |
| 12769871 | SSH2 | Slingshot Protein Phosphatase 2 | 2.728843215 | 42 | 56 | 56 | 48 | 127 | 135 | 102 | 201 | 231 | 193 | 208 | 252 | 238 |
| 12678550 | SST | Somatostatin | 37.59260817 | 11 | 15 | 11 | 14 | 15 | 14 | 11 | 561 | 608 | 122 | 908 | 435 | 287 |
| 12721672 | ST3GAL1 | ST3 beta-galactoside alpha-2,3-sialyltransferase 1 (ST3GAL1), mRNA. | 2.689842006 | 55 | 58 | 64 | 76 | 66 | 68 | 67 | 201 | 160 | 162 | 192 | 133 | 200 |
| 12704183 | ST6GALNAC4 | ST6 (alpha-N-acetyl-neuraminyl-2,3-beta-galactosyl-1, 3)-N-acetylgalactosaminide alpha-2,6-sialyltransferase 4 (ST6GALNAC4), mRNA. | 3.015318488 | 315 | 363 | 354 | 214 | 346 | 281 | 388 | 972 | 988 | 910 | 1153 | 723 | 1100 |
| 12704200 | ST6GALNAC6 | ST6 (alpha-N-acetyl-neuraminyl-2,3-beta-galactosyl-1, 3)-N-acetylgalactosaminide alpha-2,6-sialyltransferase 6 (ST6GALNAC6), mRNA. | 2.180736028 | 225 | 233 | 355 | 258 | 305 | 252 | 265 | 548 | 590 | 539 | 645 | 549 | 670 |
| 12824769 | STAR | steroidogenic acute regulatory protein (STAR), nuclear gene encoding mitochondrial protein, mRNA. | 5.501730721 | 30 | 80 | 123 | 40 | 1138 | 1194 | 2110 | 4075 | 3720 | 3896 | 3500 | 3274 | 3776 |
| 12710347 | STARD13 | StAR-related lipid transfer (START) domain containing 13 (STARD13), mRNA. | 4.520394335 | 24 | 23 | 52 | 21 | 112 | 108 | 161 | 253 | 291 | 281 | 326 | 410 | 377 |
| 12688345 | STARD4 | StAR-Related Lipid Transfer (START) Domain Containing 4 | 3.331350705 | 119 | 106 | 114 | 77 | 98 | 55 | 87 | 357 | 222 | 298 | 374 | 312 | 309 |
| 12909481 | STARD8 | StAR-related lipid transfer (START) domain containing 8 (STARD8), mRNA. | 2.847903458 | 41 | 40 | 47 | 44 | 66 | 87 | 88 | 201 | 194 | 161 | 127 | 178 | 145 |
| 12768925 | STAT3 | signal transducer and activator of transcription 3 (acute-phase response factor) (STAT3), mRNA. | 3.366627615 | 290 | 306 | 376 | 314 | 534 | 575 | 690 | 1558 | 1645 | 1808 | 1218 | 1209 | 1464 |
| 12761115 | STAT5A | signal transducer and activator of transcription 5A (STAT5A), mRNA. | 2.037002111 | 101 | 88 | 85 | 56 | 92 | 96 | 78 | 151 | 193 | 181 | 168 | 163 | 186 |
| 12767469 | STAT5B | signal transducer and activator of transcription 5B (STAT5B), mRNA. | 2.77638225 | 54 | 56 | 53 | 54 | 82 | 121 | 84 | 193 | 175 | 234 | 172 | 184 | 243 |
| 12857400 | STAT6 | signal transducer and activator of transcription 6, interleukin-4 induced (STAT6), mRNA. | 2.103004092 | 87 | 93 | 107 | 79 | 141 | 152 | 132 | 226 | 283 | 224 | 255 | 217 | 220 |
| 12850240 | STEAP1 | six transmembrane epithelial antigen of the prostate 1 (STEAP1), mRNA. | 9.762893951 | 15 | 25 | 17 | 19 | 28 | 38 | 81 | 274 | 310 | 231 | 365 | 319 | 368 |
| 12849526 | STEAP2 | STEAP family member 2, metalloreductase (STEAP2), mRNA. | 3.777175606 | 34 | 46 | 48 | 37 | 82 | 82 | 86 | 190 | 209 | 196 | 224 | 218 | 305 |
| 12787808 | STK10 | serine/threonine kinase 10 (STK10), mRNA. | 2.471455179 | 49 | 44 | 57 | 58 | 105 | 102 | 95 | 189 | 204 | 177 | 175 | 151 | 185 |
| 12854087 | STK17A | Serine/Threonine Kinase 17a | 3.951363369 | 34 | 33 | 23 | 35 | 26 | 43 | 29 | 149 | 105 | 134 | 115 | 112 | 143 |
| 12781322 | STK17B | Serine/Threonine Kinase 17b | 2.316938643 | 73 | 90 | 106 | 87 | 63 | 60 | 63 | 152 | 163 | 161 | 199 | 175 | 225 |
| 12838532 | STK40 | serine/threonine kinase 40 (STK40), mRNA. | 2.198946389 | 154 | 151 | 151 | 118 | 207 | 188 | 208 | 342 | 372 | 331 | 388 | 421 | 365 |
| 12879916 | STX10 | syntaxin 10 (STX10), mRNA. | 2.25182319 | 100 | 97 | 88 | 82 | 158 | 158 | 118 | 273 | 237 | 229 | 301 | 275 | 230 |
| 12898561 | STX11 | syntaxin 11 (STX11), mRNA. | 8.692561206 | 55 | 64 | 44 | 44 | 39 | 45 | 46 | 216 | 252 | 199 | 405 | 772 | 670 |
| 12698638 | STXBP1 | Syntaxin Binding Protein 1 | 2.108072575 | 251 | 249 | 338 | 231 | 343 | 373 | 348 | 621 | 700 | 716 | 602 | 567 | 648 |
| 12815269 | SULT1A1 | Sulfotransferase Family, Cytosolic, 1A, Phenol-Preferring, Member 1 | 5.112698288 | 77 | 86 | 86 | 90 | 728 | 454 | 515 | 1602 | 1614 | 1740 | 1543 | 848 | 1576 |
| 12865176 | SUOX | sulfite oxidase (SUOX), nuclear gene encoding mitochondrial protein, mRNA. | 2.488064622 | 229 | 276 | 382 | 245 | 432 | 422 | 512 | 927 | 795 | 967 | 1017 | 630 | 993 |
| 12688887 | SUSD6 | Sushi Domain Containing 6 (SUSD6; KIAA0247) | 2.204743777 | 149 | 132 | 161 | 141 | 149 | 105 | 115 | 242 | 273 | 271 | 303 | 389 | 321 |
| 12895667 | SVEP1 | PREDICTED: sushi, von Willebrand factor type A, EGF and pentraxin domain containing 1 (SVEP1), mRNA. | 5.769606002 | 21 | 26 | 24 | 18 | 31 | 30 | 25 | 155 | 190 | 229 | 84 | 79 | 127 |
| 12732746 | SWAP70 | SWAP switching B-cell complex 70kDa subunit (SWAP70), mRNA. | 6.01393645 | 35 | 39 | 36 | 47 | 72 | 92 | 85 | 405 | 499 | 584 | 197 | 181 | 231 |
| 12906626 | SYN1 | synapsin I, mRNA (cDNA clone MGC:152080 IMAGE:8393130), complete cds. | 2.297754538 | 54 | 49 | 47 | 45 | 55 | 70 | 70 | 93 | 126 | 109 | 135 | 109 | 196 |
| 12833635 | TALDO1 | transaldolase 1 (TALDO1), mRNA. | 2.229479994 | 151 | 148 | 142 | 217 | 208 | 153 | 147 | 418 | 360 | 517 | 357 | 271 | 304 |
| 12868212 | TBC1D30 | PREDICTED: TBC1 domain family, member 30 (TBC1D30), mRNA. | 2.997458128 | 35 | 20 | 32 | 26 | 35 | 49 | 50 | 81 | 132 | 111 | 92 | 117 | 100 |
| 12906927 | TBC1D8B | PREDICTED: TBC1 domain family, member 8B (with GRAM domain) (TBC1D8B), partial mRNA. | 2.088630787 | 309 | 342 | 423 | 477 | 414 | 265 | 600 | 873 | 1003 | 665 | 846 | 745 | 934 |
| 12743968 | TBC1D9 | PREDICTED: TBC1 domain family, member 9 (with GRAM domain) (TBC1D9), mRNA. | 2.415610232 | 160 | 157 | 164 | 166 | 139 | 144 | 124 | 268 | 319 | 284 | 340 | 552 | 420 |
| 12744751 | TBX3 | T-Box 3 | 5.876345739 | 23 | 43 | 23 | 24 | 75 | 85 | 131 | 363 | 383 | 282 | 307 | 394 | 305 |
| 12793256 | TC2N | tandem C2 domains, nuclear (TC2N), mRNA. | 3.715781194 | 1315 | 1416 | 1489 | 1297 | 1412 | 1228 | 1354 | 5689 | 5296 | 5967 | 4772 | 3980 | 4587 |
| 12810271 | TCF4 | transcription factor 4 (TCF4), mRNA. | 4.259315722 | 54 | 52 | 95 | 61 | 233 | 374 | 252 | 773 | 820 | 590 | 604 | 727 | 582 |
| 12741946 | TCN2 | transcobalamin II (TCN2), mRNA. | 2.674445856 | 160 | 152 | 200 | 111 | 278 | 296 | 232 | 564 | 589 | 465 | 606 | 576 | 477 |
| 12796997 | TCTA | T-cell leukemia translocation altered gene (TCTA), mRNA. | 2.042214214 | 150 | 134 | 156 | 192 | 203 | 164 | 152 | 384 | 276 | 335 | 428 | 301 | 289 |
| 12683216 | TF | transferrin | 13.48564977 | 13 | 22 | 25 | 20 | 96 | 176 | 346 | 890 | 1318 | 859 | 1653 | 1653 | 1695 |
| 12853224 | TFPI2 | tissue factor pathway inhibitor 2 (TFPI2), mRNA. | 43.86206724 | 27 | 18 | 17 | 27 | 80 | 87 | 59 | 1464 | 2297 | 2231 | 1716 | 1627 | 2505 |
| 12702269 | TGFA | Transforming Growth Factor, Alpha | 4.488998962 | 49 | 50 | 52 | 49 | 59 | 61 | 55 | 305 | 285 | 393 | 162 | 101 | 190 |
| 12755803 | TGFB1 | Transforming Growth Factor, Beta 1 | 2.703265454 | 37 | 35 | 31 | 22 | 62 | 72 | 51 | 178 | 125 | 110 | 114 | 103 | 92 |
| 12796464 | TGFBR2 | transforming growth factor, beta receptor II (70/80kDa) (TGFBR2), mRNA. | 5.638454578 | 72 | 81 | 76 | 61 | 323 | 559 | 448 | 1953 | 2006 | 1316 | 950 | 955 | 656 |
| 12807824 | TGIF1 | TGFB-induced factor homeobox 1 (TGIF1), mRNA. | 2.409633582 | 157 | 163 | 122 | 178 | 153 | 114 | 127 | 331 | 332 | 339 | 380 | 436 | 274 |
| 12718026 | THBD | thrombomodulin (THBD), mRNA. | 6.222260286 | 29 | 26 | 25 | 27 | 103 | 73 | 57 | 327 | 422 | 447 | 180 | 243 | 197 |
| 12899536 | THBS2 | thrombospondin 2 (THBS2), mRNA. | 7.062849322 | 75 | 65 | 81 | 42 | 158 | 219 | 242 | 636 | 460 | 421 | 1291 | 1296 | 1234 |
| 12681636 | TIAM1 | T-cell lymphoma invasion and metastasis 1 | 7.691678875 | 30 | 31 | 25 | 31 | 28 | 30 | 47 | 190 | 262 | 180 | 251 | 238 | 334 |
| 12878519 | TICAM1 | Toll-Like Receptor Adaptor Molecule 1 | 2.683662733 | 49 | 43 | 59 | 41 | 63 | 55 | 47 | 157 | 128 | 132 | 131 | 153 | 122 |
| 12902909 | TIMP1 | TIMP metallopeptidase inhibitor 1 (TIMP1), mRNA. | 8.838403321 | 83 | 103 | 174 | 43 | 1090 | 1145 | 835 | 3838 | 4646 | 3637 | 4660 | 4530 | 4993 |
| 12781035 | TINAGL1 | PREDICTED: tubulointerstitial nephritis antigen-like 1 (TINAGL1), mRNA. | 4.931297409 | 62 | 40 | 42 | 36 | 136 | 200 | 181 | 790 | 649 | 680 | 283 | 315 | 228 |
| 12693241 | TINF2 | TERF1 (TRF1)-interacting nuclear factor 2 (TINF2), mRNA. | 2.066657699 | 203 | 242 | 205 | 208 | 168 | 175 | 166 | 419 | 356 | 443 | 466 | 369 | 366 |
| 12686567 | TIPARP | TCDD-inducible poly(ADP-ribose) polymerase (TIPARP), mRNA. | 7.591512348 | 200 | 195 | 184 | 179 | 168 | 166 | 242 | 1268 | 1211 | 1451 | 1662 | 1630 | 1463 |
| 12889280 | TJP2 | tight junction protein 2 (zona occludens 2) (TJP2), mRNA. | 3.700554075 | 78 | 69 | 73 | 56 | 69 | 98 | 84 | 276 | 288 | 267 | 258 | 291 | 292 |
| 12893523 | TLE1 | transducin-like enhancer of split 1 (E(sp1) homolog, Drosophila) (TLE1), mRNA. | 2.09263656 | 75 | 85 | 88 | 60 | 82 | 84 | 80 | 117 | 129 | 112 | 198 | 257 | 182 |
| 12889086 | TLR4 | Toll-Like Receptor 4 | 4.021728943 | 23 | 22 | 35 | 13 | 47 | 81 | 92 | 161 | 198 | 139 | 177 | 227 | 176 |
| 12681340 | TM4SF1 | transmembrane 4 L six family member 1 | 6.952072624 | 42 | 26 | 27 | 27 | 190 | 619 | 669 | 2130 | 2142 | 2256 | 980 | 1011 | 1016 |
| 12829453 | TM7SF2 | transmembrane 7 superfamily member 2 (TM7SF2), mRNA. | 5.625204645 | 154 | 138 | 135 | 64 | 751 | 323 | 279 | 1199 | 1480 | 1367 | 1769 | 1092 | 1978 |
| 12850265 | TMEM140 | transmembrane protein 140 (TMEM140), mRNA. | 2.252369292 | 48 | 56 | 50 | 43 | 88 | 97 | 102 | 156 | 146 | 145 | 149 | 170 | 169 |
| 12875089 | TMEM150C | transmembrane protein 150C (TMEM150C), mRNA. | 12.51408406 | 33 | 37 | 25 | 44 | 249 | 162 | 233 | 1424 | 1113 | 1323 | 1482 | 1735 | 1334 |
| 12849348 | TMEM176A | transmembrane protein 176A (TMEM176A), mRNA. | 3.411093248 | 1016 | 1016 | 1093 | 860 | 1181 | 1132 | 1538 | 3820 | 4053 | 2738 | 5340 | 3243 | 3716 |
| 12855804 | TMEM176B | transmembrane protein 176B (TMEM176B), mRNA. | 3.033179078 | 1063 | 1010 | 1125 | 808 | 750 | 1009 | 1219 | 3081 | 3364 | 1969 | 4349 | 2789 | 2605 |
| 12893870 | TMEM2 | transmembrane protein 2 (TMEM2), mRNA. | 2.590606145 | 53 | 59 | 71 | 50 | 88 | 89 | 134 | 255 | 294 | 184 | 153 | 178 | 142 |
| 12886445 | TMEM38A | transmembrane protein 38A (TMEM38A), mRNA. | 2.692280332 | 37 | 35 | 26 | 19 | 58 | 61 | 47 | 80 | 98 | 91 | 165 | 116 | 104 |
| 12798151 | TMEM43 | transmembrane protein 43 (TMEM43), mRNA. | 2.488641348 | 357 | 319 | 306 | 345 | 360 | 298 | 284 | 1004 | 832 | 842 | 796 | 655 | 710 |
| 12737563 | TMEM52 | transmembrane protein 52, mRNA (cDNA clone IMAGE:8011408), partial cds. | 2.149427527 | 107 | 100 | 102 | 86 | 247 | 162 | 172 | 322 | 292 | 313 | 350 | 218 | 303 |
| 12840924 | TMEM53 | transmembrane protein 53 (TMEM53), mRNA. | 2.057005003 | 55 | 53 | 64 | 45 | 57 | 56 | 57 | 140 | 116 | 166 | 92 | 74 | 95 |
| 12775974 | TMEM54 | transmembrane protein 54 (TMEM54), mRNA. | 3.703891134 | 55 | 51 | 55 | 44 | 52 | 50 | 46 | 146 | 166 | 166 | 282 | 170 | 193 |
| 12740309 | TMEM63A | transmembrane protein 63A (TMEM63A), mRNA. | 2.305044051 | 307 | 286 | 234 | 191 | 463 | 423 | 438 | 777 | 745 | 723 | 785 | 781 | 815 |
| 12825157 | TMEM66 | transmembrane protein 66 (TMEM66), mRNA. | 2.041309398 | 649 | 743 | 718 | 749 | 1028 | 1114 | 1077 | 1587 | 1587 | 1570 | 1882 | 1976 | 2032 |
| 12695970 | TMOD2 | tropomodulin 2 (neuronal) (TMOD2), mRNA. | 4.815946602 | 31 | 36 | 55 | 41 | 44 | 65 | 36 | 179 | 304 | 201 | 127 | 250 | 213 |
| 12856674 | TMTC2 | PREDICTED: transmembrane and tetratricopeptide repeat containing 2 (TMTC2), mRNA. | 2.451560216 | 80 | 80 | 102 | 89 | 252 | 296 | 217 | 399 | 386 | 361 | 392 | 487 | 321 |
| 12896970 | TNFAIP3 | tumor necrosis factor, alpha-induced protein 3 (TNFAIP3), mRNA. | 20.0993824 | 60 | 44 | 73 | 37 | 105 | 106 | 75 | 1466 | 1097 | 1386 | 1345 | 1285 | 2017 |
| 12886230 | TNFAIP8 | tumor necrosis factor, alpha-induced protein 8 (TNFAIP8), mRNA. | 3.559960757 | 24 | 19 | 15 | 16 | 51 | 48 | 61 | 83 | 111 | 71 | 130 | 178 | 138 |
| 12892054 | TNFRSF10A | PREDICTED: tumor necrosis factor receptor superfamily, member 10a (TNFRSF10A), mRNA. | 13.59543778 | 48 | 116 | 38 | 21 | 39 | 36 | 43 | 506 | 425 | 194 | 866 | 1634 | 343 |
| 12895762 | TNFRSF10D | tumor necrosis factor receptor superfamily, member 10d, decoy with truncated death domain (TNFRSF10D), mRNA. | 5.343975816 | 148 | 117 | 135 | 142 | 245 | 287 | 206 | 520 | 908 | 972 | 806 | 978 | 1679 |
| 12857267 | TNFRSF1A | tumor necrosis factor receptor superfamily, member 1A (TNFRSF1A), mRNA. | 2.580555302 | 250 | 304 | 272 | 196 | 792 | 600 | 541 | 1085 | 1055 | 861 | 1442 | 1131 | 958 |
| 12740702 | TNFSF4 | tumor necrosis factor (ligand) superfamily, member 4 (TNFSF4), mRNA. | 4.920368043 | 21 | 16 | 15 | 14 | 28 | 37 | 33 | 103 | 69 | 75 | 183 | 166 | 97 |
| 12885985 | TNIP1 | TNFAIP3 interacting protein 1 (TNIP1), mRNA. | 2.140584663 | 76 | 72 | 87 | 84 | 77 | 73 | 60 | 139 | 139 | 163 | 174 | 155 | 201 |
| 12735818 | TNN | tenascin N (TNN), mRNA. | 5.612164154 | 21 | 20 | 21 | 20 | 19 | 22 | 23 | 190 | 213 | 158 | 62 | 41 | 32 |
| 12723730 | TP53INP1 | tumor protein p53 inducible nuclear protein 1 (TP53INP1), mRNA. | 2.096342283 | 300 | 286 | 377 | 294 | 384 | 449 | 445 | 656 | 604 | 583 | 705 | 1074 | 935 |
| 12801716 | TPMT | thiopurine S-methyltransferase (TPMT), mRNA. | 3.058116297 | 104 | 142 | 139 | 122 | 164 | 145 | 180 | 451 | 407 | 520 | 456 | 361 | 414 |
| 12759184 | TPPP3 | tubulin polymerization-promoting protein family member 3 (TPPP3), mRNA. | 7.882246977 | 49 | 65 | 57 | 37 | 79 | 121 | 58 | 715 | 578 | 867 | 490 | 226 | 276 |
| 12812405 | TPST1 | tyrosylprotein sulfotransferase 1 (TPST1), mRNA. | 2.464596883 | 48 | 51 | 46 | 50 | 62 | 57 | 55 | 142 | 102 | 178 | 115 | 119 | 126 |
| 12898948 | TRAF3IP2 | TRAF3 interacting protein 2 (TRAF3IP2), mRNA. | 4.275671949 | 66 | 65 | 61 | 60 | 68 | 81 | 66 | 176 | 259 | 217 | 276 | 343 | 436 |
| 12725414 | TRIB1 | tribbles homolog 1 (Drosophila) (TRIB1), mRNA. | 19.14900971 | 41 | 35 | 52 | 39 | 67 | 56 | 64 | 1101 | 1003 | 1190 | 789 | 798 | 939 |
| 12747792 | TRIM2 | tripartite motif containing 2 (TRIM2), mRNA. | 2.130595748 | 331 | 298 | 314 | 370 | 420 | 421 | 307 | 587 | 713 | 643 | 717 | 1023 | 811 |
| 12769470 | TRIM25 | tripartite motif containing 25 (TRIM25), mRNA. | 2.280900363 | 129 | 132 | 168 | 154 | 221 | 241 | 263 | 319 | 429 | 414 | 390 | 445 | 560 |
| 12886534 | TRIP10 | Thyroid Hormone Receptor Interactor 10 | 2.779128539 | 121 | 128 | 115 | 135 | 375 | 431 | 368 | 593 | 704 | 660 | 533 | 783 | 713 |
| 12894284 | TRPM6 | Transient Receptor Potential Cation Channel, Subfamily M, Member 6 | 5.278310858 | 18 | 14 | 16 | 14 | 22 | 27 | 21 | 103 | 129 | 138 | 62 | 70 | 95 |
| 12902223 | TSC22D3 | TSC22 Domain Family, Member 3 | 3.629415592 | 118 | 93 | 174 | 72 | 323 | 381 | 390 | 831 | 800 | 1154 | 725 | 589 | 726 |
| 12855669 | TSPAN12 | tetraspanin 12 (TSPAN12), mRNA. | 17.98121597 | 31 | 33 | 36 | 29 | 60 | 120 | 128 | 891 | 1738 | 1017 | 1341 | 742 | 1009 |
| 12850708 | TSPAN13 | tetraspanin 13 (TSPAN13), mRNA. | 4.692474698 | 68 | 77 | 134 | 96 | 119 | 143 | 138 | 870 | 623 | 800 | 268 | 249 | 306 |
| 12826881 | TSPAN14 | tetraspanin 14 (TSPAN14), mRNA. | 2.638578941 | 89 | 97 | 138 | 54 | 101 | 110 | 120 | 293 | 291 | 296 | 244 | 311 | 169 |
| 12826384 | TSPAN15 | tetraspanin 15 (TSPAN15), mRNA. | 7.556618693 | 38 | 36 | 19 | 21 | 40 | 38 | 30 | 274 | 248 | 154 | 342 | 119 | 296 |
| 12730259 | TSPAN18 | Tetraspanin 18 | 4.905489892 | 43 | 42 | 51 | 30 | 78 | 110 | 71 | 250 | 232 | 168 | 440 | 399 | 294 |
| 12810182 | TTC39C | Tetratricopeptide Repeat Domain 39C | 2.200183137 | 137 | 147 | 249 | 274 | 97 | 134 | 120 | 294 | 408 | 282 | 389 | 414 | 396 |
| 12754384 | TUBB3 | tubulin, beta 3 class III (TUBB3), mRNA. | 2.105443296 | 62 | 68 | 58 | 55 | 76 | 49 | 69 | 108 | 120 | 159 | 150 | 117 | 136 |
| 12842719 | TUFT1 | Tuftelin 1 | 2.180354801 | 58 | 60 | 70 | 71 | 47 | 44 | 44 | 77 | 113 | 105 | 127 | 140 | 175 |
| 12864004 | TXN2 | thioredoxin 2 (TXN2), nuclear gene encoding mitochondrial protein, mRNA. | 2.510620188 | 360 | 346 | 330 | 195 | 517 | 440 | 427 | 1256 | 847 | 1177 | 1003 | 625 | 719 |
| 12742118 | UBC | ubiquitin C (UBC), mRNA. | 3.083746254 | 88 | 92 | 92 | 83 | 69 | 87 | 80 | 232 | 241 | 224 | 229 | 325 | 314 |
| 12766526 | ULBP11 | UL16-binding protein 11 (ULBP11), mRNA. | 25.07488941 | 5 | 45 | 6 | 5 | 4 | 5 | 4 | 32 | 295 | 580 | 41 | 277 | 369 |
| 12747034 | ULK1 | unc-51-like kinase 1 (C. elegans) (ULK1), mRNA. | 2.280636182 | 216 | 174 | 200 | 115 | 238 | 189 | 153 | 399 | 457 | 422 | 408 | 446 | 382 |
| 12678618 | UMPS | uridine monophosphate synthetase | 2.351809482 | 148 | 124 | 145 | 152 | 212 | 211 | 218 | 372 | 584 | 496 | 349 | 296 | 341 |
| 12826450 | UNC5B | unc-5 homolog B (C. elegans) (UNC5B), mRNA. | 5.668439216 | 56 | 38 | 58 | 51 | 63 | 75 | 68 | 553 | 505 | 351 | 207 | 212 | 164 |
| 12699139 | USP20 | ubiquitin specific peptidase 20 (USP20), mRNA. | 2.037951312 | 43 | 41 | 47 | 42 | 80 | 54 | 69 | 111 | 93 | 110 | 124 | 123 | 98 |
| 12859331 | VAMP1 | Vesicle-Associated Membrane Protein 1 (Synaptobrevin 1) | 2.135126489 | 62 | 91 | 76 | 67 | 85 | 76 | 60 | 136 | 149 | 107 | 192 | 215 | 148 |
| 12740649 | VASH2 | vasohibin 2 (VASH2), mRNA. | 2.501141157 | 78 | 66 | 63 | 67 | 78 | 76 | 64 | 129 | 132 | 170 | 226 | 167 | 229 |
| 12842734 | VCAM1 | vascular cell adhesion molecule 1 (VCAM1), mRNA. | 7.661972986 | 127 | 153 | 133 | 103 | 88 | 120 | 105 | 887 | 623 | 613 | 1315 | 1191 | 808 |
| 12791646 | WARS | Tryptophanyl-TRNA Synthetase | 2.123172541 | 305 | 310 | 338 | 276 | 464 | 401 | 508 | 882 | 901 | 940 | 628 | 559 | 825 |
| 12816646 | WBSCR22 | Williams Beuren Syndrome Chromosome Region 22 | 6.591990525 | 301 | 282 | 387 | 208 | 371 | 304 | 416 | 3440 | 2579 | 3639 | 913 | 692 | 1551 |
| 12720191 | WDR37 | WD repeat domain 37 (WDR37), mRNA. | 2.251617451 | 237 | 228 | 233 | 210 | 205 | 183 | 195 | 400 | 449 | 429 | 526 | 492 | 580 |
| 12703781 | XDH | xanthine dehydrogenase (XDH), mRNA. | 5.051060209 | 21 | 15 | 35 | 16 | 298 | 284 | 275 | 628 | 777 | 490 | 791 | 716 | 681 |
| 12740719 | YOD1 | YOD1 OTU deubiquinating enzyme 1 homolog (S. cerevisiae) (YOD1), mRNA. | 6.045744478 | 125 | 123 | 117 | 126 | 99 | 70 | 85 | 477 | 579 | 389 | 851 | 788 | 777 |
| 12814558 | YPEL3 | yippee-like 3 (Drosophila) (YPEL3), mRNA. | 2.2085507 | 267 | 242 | 234 | 180 | 318 | 236 | 148 | 497 | 490 | 422 | 574 | 643 | 452 |
| 12846451 | ZC3H12A | zinc finger CCCH-type containing 12A (ZC3H12A), mRNA. | 4.204353514 | 60 | 53 | 64 | 56 | 53 | 54 | 50 | 214 | 261 | 232 | 213 | 241 | 241 |
| 12813899 | ZFAND2A | zinc finger, AN1-type domain 2A (ZFAND2A), mRNA. | 10.65363773 | 83 | 107 | 84 | 111 | 92 | 85 | 94 | 877 | 1185 | 1060 | 725 | 1004 | 1147 |
| 12749424 | ZFP36 | zinc finger protein 36, C3H type, homolog (mouse) (ZFP36), mRNA. | 17.01590167 | 171 | 140 | 153 | 129 | 245 | 195 | 242 | 3094 | 2794 | 2886 | 3106 | 3533 | 3207 |
| 12697103 | ZFP36L1 | zinc finger protein 36, C3H type-like 1 (ZFP36L1), mRNA. | 4.655340677 | 155 | 158 | 136 | 97 | 662 | 718 | 681 | 1709 | 1534 | 1391 | 1964 | 2026 | 1777 |
| 12708387 | ZFP36L2 | zinc finger protein 36, C3H type-like 2 (ZFP36L2), mRNA. | 3.891701903 | 299 | 284 | 190 | 129 | 499 | 348 | 222 | 1041 | 1008 | 762 | 1218 | 1572 | 974 |
| 12725274 | ZHX2 | zinc fingers and homeoboxes 2 (ZHX2), mRNA. | 3.992752137 | 50 | 68 | 73 | 48 | 78 | 68 | 54 | 201 | 197 | 146 | 323 | 283 | 349 |
| 12827158 | ZMIZ1 | PREDICTED: zinc finger, MIZ-type containing 1 (ZMIZ1), mRNA. | 2.25100456 | 398 | 371 | 411 | 224 | 331 | 237 | 267 | 760 | 706 | 908 | 657 | 538 | 753 |
| 12772356 | ZMYND15 | PREDICTED: zinc finger, MYND-type containing 15 (ZMYND15), mRNA. | 3.218497794 | 32 | 29 | 25 | 29 | 38 | 44 | 34 | 88 | 78 | 69 | 141 | 141 | 119 |
| 12756497 | ZNF423 | zinc finger protein 423 (ZNF423), mRNA. | 2.923967288 | 71 | 44 | 52 | 38 | 86 | 123 | 95 | 231 | 316 | 210 | 157 | 186 | 174 |
| 12888194 | ZNF791 | Zinc Finger Protein 791 | 7.051672286 | 58 | 62 | 80 | 69 | 68 | 56 | 68 | 311 | 150 | 498 | 410 | 325 | 1096 |
| 12720706 | ZNFX1 | zinc finger, NFX1-type containing 1 (ZNFX1), mRNA. | 2.28808548 | 150 | 143 | 134 | 149 | 143 | 146 | 293 | 336 | 361 | 532 | 246 | 318 | 474 |
